# A Nickel-Catalyzed Cross-Electrophile Coupling Reaction of 1,3-Dimesylates for Alkylcyclopropane Synthesis: Investigation of Stereochemical Outcomes and Radical Lifetimes

**DOI:** 10.1021/acscatal.3c00905

**Published:** 2023-04-07

**Authors:** Pan-Pan Chen, Tristan M. McGinnis, Patricia C. Lin, Xin Hong, Elizabeth R. Jarvo

**Affiliations:** † Department of Chemistry, University of California, Irvine, California 92697, United States; § Center of Chemistry for Frontiers Technologies, Department of Chemistry, State Key Laboratory of Clean Energy Utilization, Zhejiang University, Hangzhou 310027, China; ∥ Beijing National Laboratory for Molecular Sciences, Zhongguancun North First Street No. 2, Beijing 100190, China; ⊥ Key Laboratory of Precise Synthesis of Functional Molecules of Zhejiang Province, School of Science, Westlake University, 18 Shilongshan Road, Hangzhou 310024, Zhejiang Province, China

**Keywords:** radical lifetime, radical clock, halogen-atom abstraction, cross-electrophile coupling, nickel, DFT calculations

## Abstract

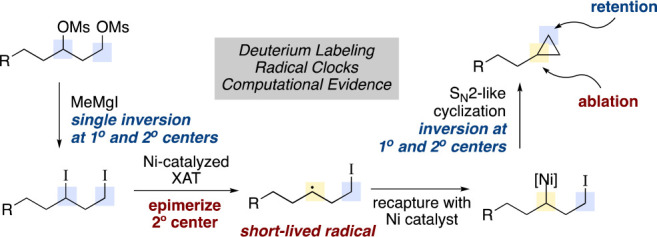

Understanding mechanistic details of the nickel-catalyzed coupling reactions of Csp^3^ alcohol derivatives is key to developing selective reactions of this widely prevalent functional group. In this manuscript, we utilize a combination of experimental data and DFT studies to define the key intermediates, stereochemical outcome, and competing pathways of a nickel-catalyzed cross-electrophile coupling reaction of 1,3-dimesylates. Stereospecific formation of a 1,3-diiodide intermediate is achieved in situ by the Grignard reagent. The overall stereoablative stereochemical outcome is due to a nickel-catalyzed halogen atom abstraction with a radical rebound that is slower than epimerization of the alkyl radical. Finally, lifetimes of this alkyl radical intermediate are compared to radical clocks to enhance the understanding of the lifetime of the secondary alkyl radical.

## Introduction

**Scheme 1:**
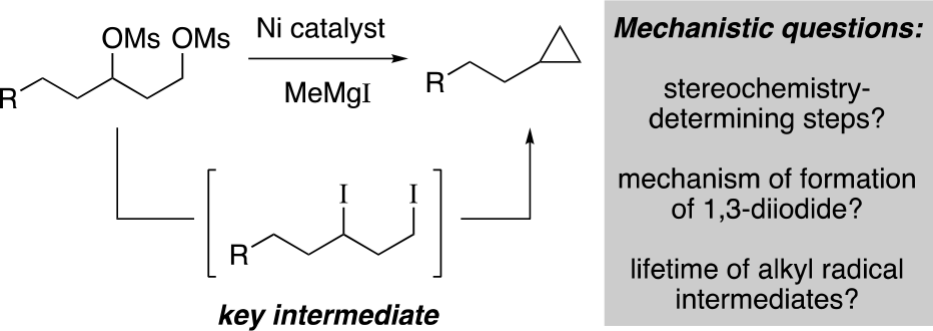
Ni-Catalyzed Cross-Electrophile Coupling Reaction of 1,3-Dimesylates

Engaging alkyl alcohol derivatives in nickel-catalyzed coupling reactions has been an exciting area of research, one that has relied on purely organometallic approaches as well as photocatalytic reactions.^1^ Building on these successes will require detailed analysis of the key mechanistic features that drive activation and selectivity in these transformations. In particular, we have developed intramolecular cross-electrophile coupling (XEC) reactions of 1,3-diols for formation of cyclopropanes.^2^ These transformations provide facile conversion of diols, functional groups that adorn natural products and synthetic intermediates, to cyclopropanes. As such, these deoxygenative reactions provide straightforward approaches toward structural remodeling of complex molecular architectures to incorporate the cyclopropropane structural element. Several mechanistic questions were raised in this reaction, each of which has important implications for the selectivity and stereochemical outcome of the reaction, and will be critical in further development of reactions that activate complex alcohols with low-valent catalysts (Scheme 1). In particular, we sought to establish the mechanism of formation of key intermediates, to identify the stereochemistry-determining steps of the transformation, and to define the lifetime of key organic radical intermediates. In this manuscript, we present a combination of labeling experiments, radical clocks, and DFT calculations that dovetail to illuminate the key mechanistic features. For example, we establish that the stereoablative reaction outcome is solely due to the nickel-catalyzed reaction and not a consequence of diiodide formation. The lessons learned from this reaction will have implications for other nickel-catalyzed reactions of alkyl electrophiles, by defining mechanisms for substrate activation and oxidative addition, as well as providing experimental data defining alkyl radical lifetimes.

## Computational Methods

All density functional theory (DFT) calculations were conducted with the Gaussian 09 software package.^3^ Geometry optimizations of all the intermediates and transition states were performed at the B3LYP^4^ level of theory with the def2-SVP^5^ basis set, including solvation energy corrections and Grimme’s D3 (BJ-damping) dispersion corrections.^6^ Based on the optimized structures, vibrational frequencies were calculated at the same level of theory to evaluate its zero-point vibrational energy (ZPVE) and thermal corrections at 298 K. The single-point energies were computed with M06^7^ functional and def2-TZVPP^5,8^ basis set, including solvation energy corrections. The solvation energies were evaluated by a self-consistent reaction field (SCRF) using SMD model.^9^ Extensive conformational searches for the intermediates and transition states have been conducted to ensure that the lowest energy conformers were located. The 3D diagrams of molecules were generated using CYLView.^10^

To correct the entropy change in solution, we applied an empirical approach^11^ because there is currently no widely accepted quantum mechanics-based approach to correct entropy in solution. For each component change in a reaction at 298 K and 1 atm, a correction of 4.3 kcal/mol is applied to the reaction free energy (i.e., a reaction from *m*- to *n*-components has an additional free energy correction for (*n* – *m*) × 4.3 kcal/mol). This approach has been validated through a number of computational and experimental studies. Yu and co-workers have found that the entropy corrections are overestimated by about half in several cycloaddition reactions.^12^ Wang and co-workers have discovered the improved description of free energy changes in a number of metal-catalyzed reactions using the same empirical approach.^13^

## Results and Discussion

**Scheme 2:**
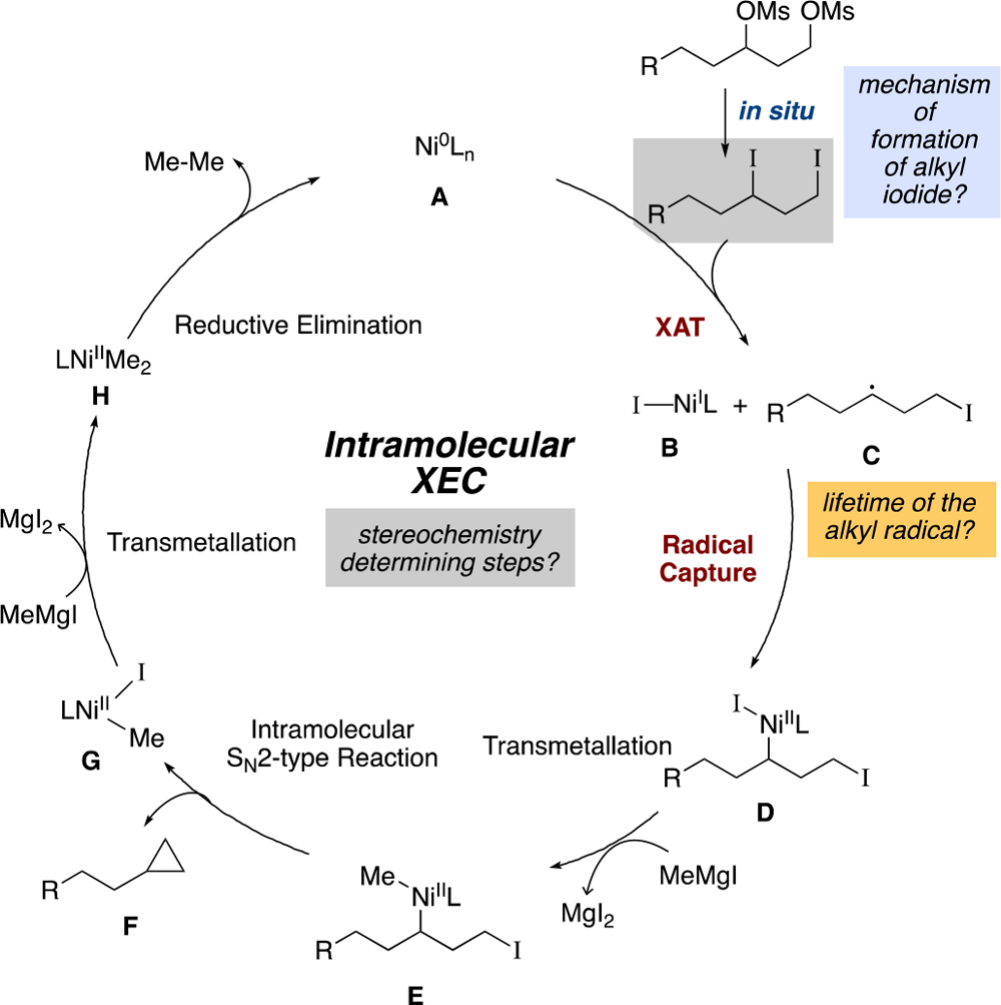
Plausible Catalytic Cycle

In broad strokes, the proposed mechanism of the XEC reaction is outlined in Scheme 2. The catalytic cycle can be divided into two parts: (1) formation of the 1,3-diiodide intermediate and (2) Ni-catalyzed XEC reaction. Our previous study provided evidence that 1,3-diiodides are formed in situ from 1,3-dimesylates before entering the nickel catalytic cycle.^2,14^ Therefore, the Ni-catalyzed XEC reaction begins with the active catalyst Ni^0^L_*n*_, **A**, and 1,3-diiodide. Halogen atom transfer (XAT) occurs to generate Ni(I) species **B** and secondary alkyl radical **C**. Then, radical rebound of **B** and **C** produces a Ni(II) species **D**, which undergoes transmetalation with Grignard reagent to deliver L_*n*_Ni(alkyl)(methyl) intermediate **E**. Subsequent intramolecular S_N_2 reaction generates cyclopropane product **F**, as well as methylNi(II) species **G**, which then undergoes transmetalation with another equivalent of Grignard reagent to produce **H**. The final step in the catalytic cycle would be the C–C reductive elimination to generate ethane and regenerate the active catalyst **A**, finishing the nickel-catalyzed cycle.

### Computational Study of Formation of the 1,3-Diiodide Intermediate

Based on the proposed catalytic cycle (Scheme 2), we studied the iodide displacement step using computational methods. The reaction with 1,3-dimesylate **1** was chosen as our computational model (eq 1).^2^ Key questions included the identity of the nucleophilic iodide source and stereochemical outcome of iodide formation.
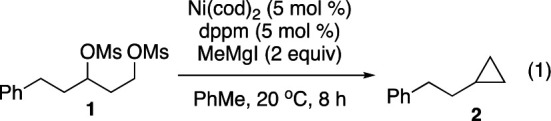
1

**Scheme 3:**
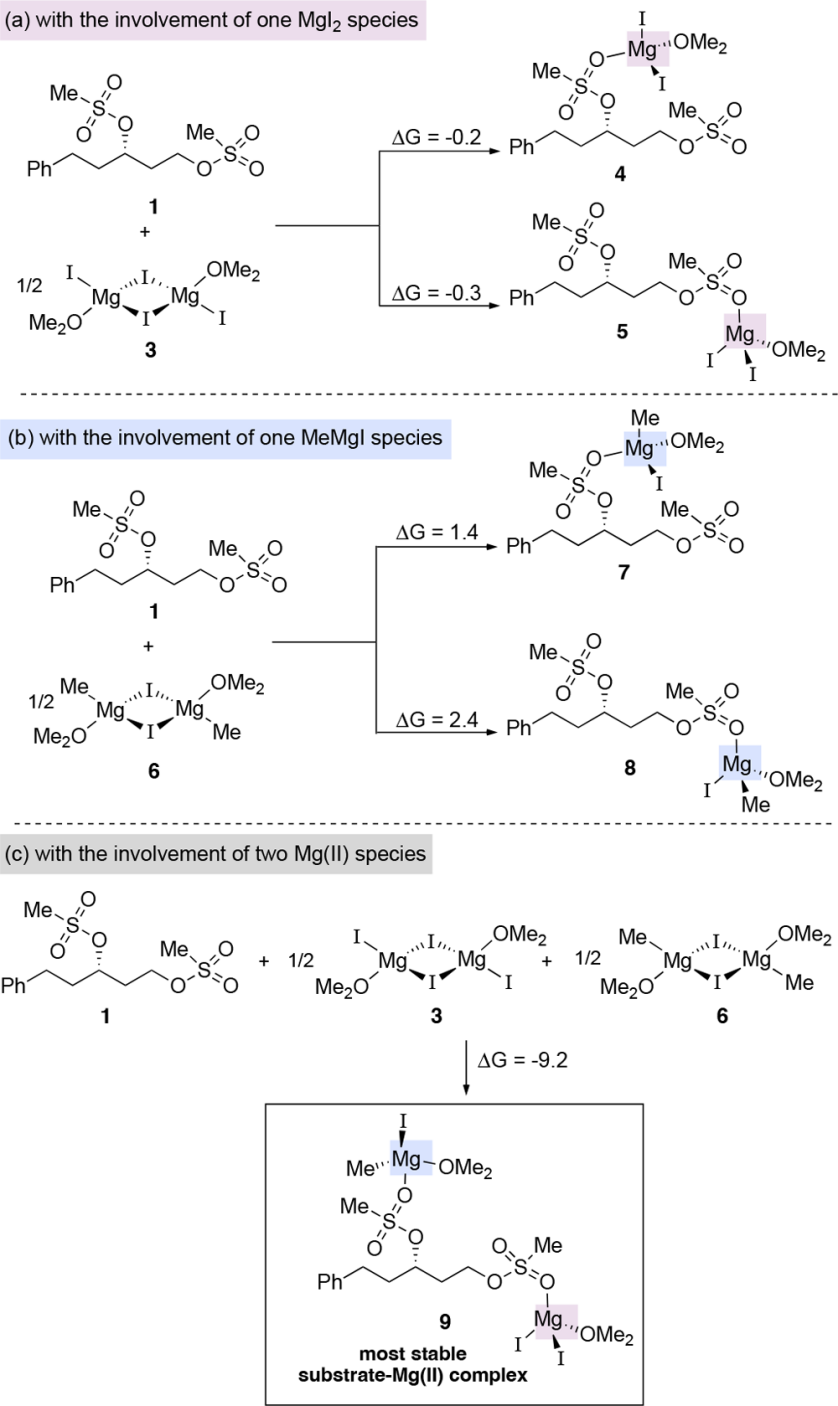
Free Energy Changes of Complexation between Substrate and Mg(II) Species Involved in the Dimesylate/MeMgI System Free energies in toluene are in kcal/mol.

Prior studies of nickel-catalyzed coupling reactions of ethers have found that magnesium salts play a key role as Lewis acid cocatalysts.^15^ To evaluate magnesium coordination to the 1,3-dimesylate **1**, we investigated the relative free energies of complexes of **1** with the Mg(II) species present in the reaction mixture based on experimental conditions (Scheme 3).^16^ MgI_2_ is present in the reaction mixture because of competitive Wurtz coupling that occurs during Grignard formation^17^ and the Schlenk equilibrium^18^ (i.e., formation of dialkyl magnesium compounds and magnesium halide salts). The μ-dihalide dimer form of Grignard reagent including two solvent molecules is well-known in the literature.^16^ Since, in our reaction system, the Grignard reagent (or MgI_2_) is a solution in diethyl ether, we employed dimethyl ether as the solvent molecule to model the magnesium dimer for MeMgI and MgI_2_, respectively. With one Mg(II) species involvement, complexation of **1** with MgI_2_ is slightly exothermic, with species **5** being marginally more favorable (Scheme 3a). The complexation between **1** and MeMgI is endothermic, where the substrate-MeMgI species are higher in energy than the starting materials by 1.4 or 2.4 kcal/mol (Scheme 3b). Bonding with MgI_2_ is stronger because of its higher Lewis acidity as compared to MeMgI (**4** vs **7**; **5** vs **8**). Since there is an additional mesylate moiety in either **4** or **5**, we hypothesized that the introduction of an equivalent of MeMgI would cause a change in the free energy of complexation. Indeed, complexation of **1** with both MgI_2_ and MeMgI is highly exothermic, generating a stable species **9** (Scheme 3c). The complexation of two Mg(II) species in **9** reduces the molecular polarity, making it lower in energy in a low polar toluene solvent, in comparison to other highly polar complexes with one Mg(II) species association (Scheme SI-8). Therefore, the MeMgI-dimesylate-MgI_2_ complex **9** was identified as the active species which forms before 1,3-diiodide intermediate formation.

**Figure 1:**
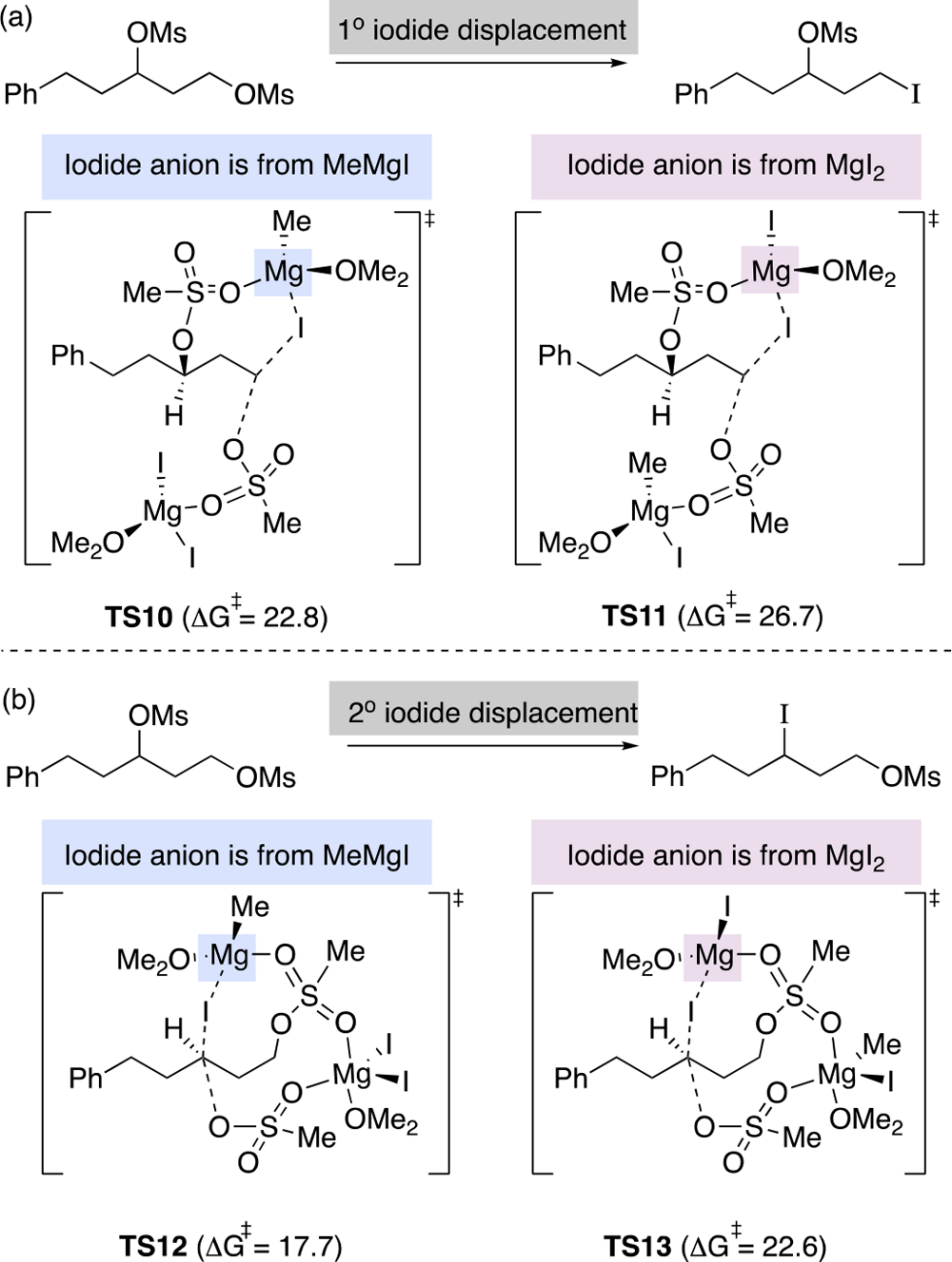
DFT-computed competitive transition states for iodide displacement involved in the dimesylate/MeMgI system. Free energies in kcal/mol are compared to those of intermediate **9**.

For the mechanism of 1,3-diiodide formation, a major question at the outset of these investigations was whether the reaction initiates with primary (1°) iodide displacement or secondary (2°) iodide displacement. To discriminate between possible reaction pathways, we began by evaluating the possible C–I bond formation transition states that could initiate the iodide displacement. Based on reaction conditions, we proposed four possible transition states according to the regioselectivity of the first C–I bond formation and the donor of iodide ion (Figure 1).

As shown in Figure 1a, **TS10** and **TS11** are the transition states of C–I bond formation at the 1° alkyl center. In **TS10** the iodide ion donor is MeMgI, while in **TS11** the iodide anion is from MgI_2_. In Figure 1b, both **TS12** and **TS13** lead to the C–I bond formation at the 2° alkyl center with MeMgI and MgI_2_ as the respective iodide sources. Among these possible transition states, **TS12** is the most favorable, where the first C–I bond is formed at the secondary alkyl center with MeMgI as the iodide source. Grignard reagent (MeMgI) is a better source of iodide because of its higher nucleophilicity compared to MgI_2_. Moreover, MgI_2_ is more Lewis acidic than Grignard reagent, thus assisting with the dissociation of the sulfonate group. The nucleophilicity of Grignard reagent and the Lewis acidity of magnesium iodide together determine that **TS10** and **TS12** are more favorable than their competing transition states (Figure 1, **TS10** vs **TS11**; **TS12** vs **TS13**). The competition between **TS10** and **TS12** determines the regioselectivity of the first C–I bond formation, in which the secondary alkyl iodide formation via **TS12** is favored. The rationale for the energy differences between **TS10** and **TS12** is discussed in the Supporting Information (Figure SI-1).

**Figure 2:**
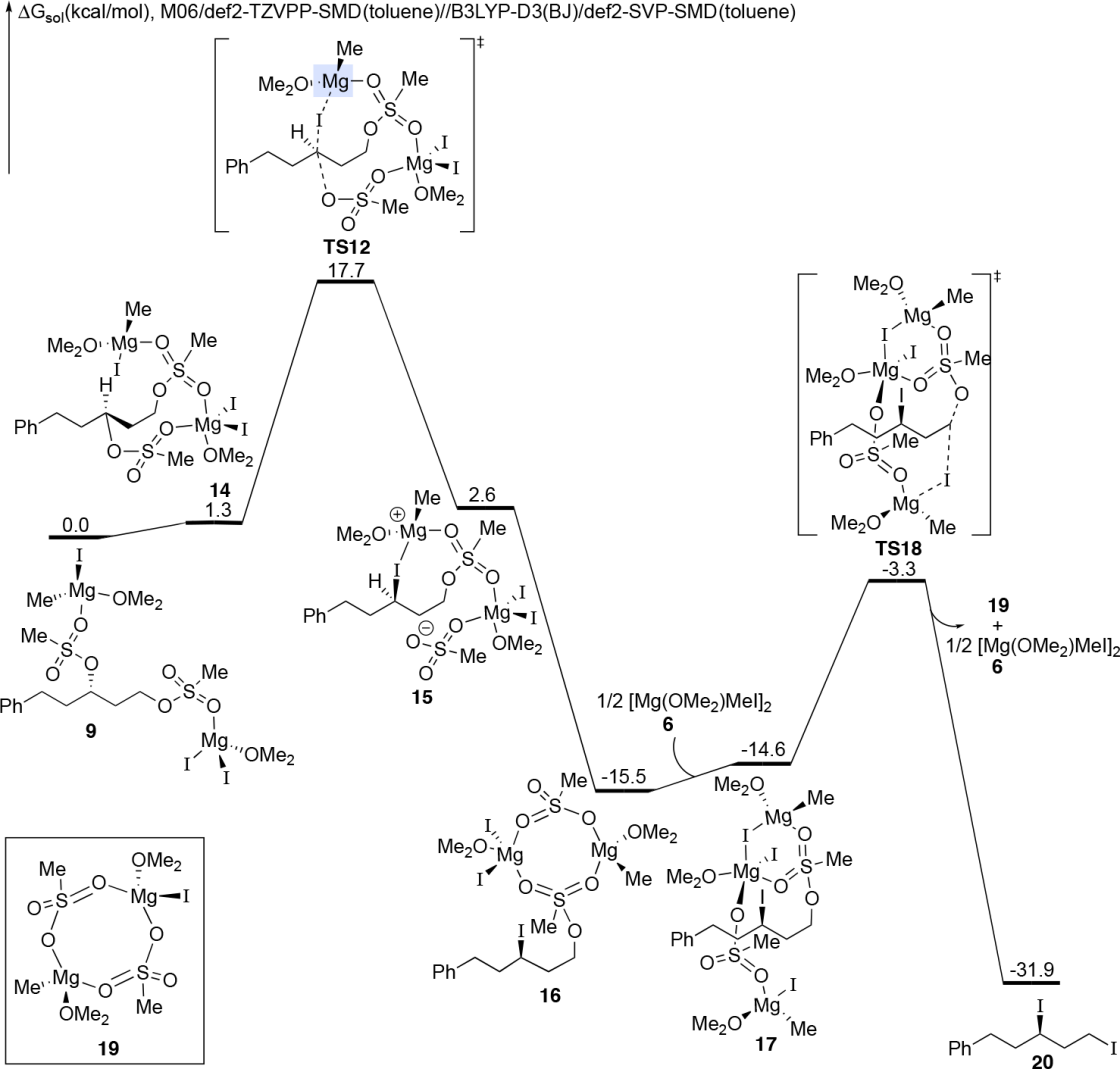
DFT-computed free energy changes of MeMgI-mediated iodide displacement of 1,3-dimesylate **1**.

The free energy changes of the most favorable pathway of MeMgI-mediated iodide displacement of dimesylate **1** are shown in Figure 2. Starting from complex **9**, conformational change occurs to give intermediate **14**, from which C–O bond displacement at the 2° alkyl center takes place through **TS12**, generating the zwitterionic species **15**. Then, the ions combine to form a stable intermediate **16**. After that, **16** complexes with another equivalent of Grignard reagent (**6**) to form species **17**, which undergoes a second C–O bond displacement at the 1° alkyl center via **TS18**. Along with the second C–I bond formation, 1,3-diiodide **20**, as well as Grignard reagent (**6**) and species **19**, is generated, finishing the transformation.

Figure 2 indicated that the reactive substrate in the 1,3-diiodide formation is the dimesylate-Mg(II) complex **9**, and the rate-limiting step for the iodide displacement is the first C–I bond formation via **TS12** with an overall barrier of 17.7 kcal/mol. Since the second C–I bond formation via **TS18** is facile with a lower barrier (**16** to **TS18**, 12.2 kcal/mol), the free energy is downhill along the reaction coordinate to provide the final product 1,3-diiodide **20**.

We also investigated the reaction pathway of 1,3-diiodide formation in the dimesylate/MgI_2_ system, in which MgI_2_ acts as both Lewis acid and nucleophile (Figure SI-2). The calculated results (Figure SI-2) are similar to those of the dimesylate/MeMgI system. The first C–I formation at the 2° alkyl center is the rate-determining step for diiodide formation, and the corresponding energy barrier is 18.3 kcal/mol. That barrier is 0.6 kcal/mol higher than the corresponding barrier of the dimesylate/MeMgI system (Figure 2), indicating that the formation of 1,3-diiodide regulated by dimesylate/MeMgI is more effective than that mediated by dimesylate/MgI_2_. These results highlight the importance of nucleophilicity of Mg(II) species in promoting such a process.

### Experimental Study of 1,3-Diiodide Formation

We sought experimental evidence to confirm that MeMgI provides facile conversion of mesylates to iodides. In addition, we were interested in investigating the stereochemical outcome of the 1,3-diiodide formation. We wished to determine whether a single displacement occurred, resulting in stereospecific formation of 1,3-diiodides, or whether multiple iodide displacements occur, resulting in stereoablative formation of the 1,3-diiodide. This understanding would be important in determining whether the overall stereoablative cyclopropane formation was due entirely to the nickel catalyst.

To begin, we sought to validate the computed mechanistic model that MeMgI mediates iodide displacement of dimesylate **21** (Table 1). We subjected 1,3-dimesylate **21** to MeMgI and MgI_2_ and compared the conversion to 1,3-diiodide **22**. To observe the reaction at partial conversion, we performed the experiment at 0 °C and stopped the reaction after 5 min. Under these conditions, using MeMgI, 45% of diiodide **22** was formed (entry 1). In contrast, the reaction employing MgI_2_ generated only 13% of diiodide **22** in the same time period (entry 2).^19^ Therefore, the reaction mixture containing MeMgI generated iodide faster than when employing only MgI_2_. This experimental data is consistent with the computed results and confirms that MeMgI is critical for rapid and clean formation of 1,3-diiodide in situ.

**Table 1:**
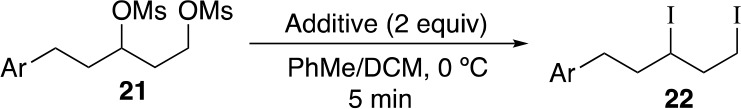

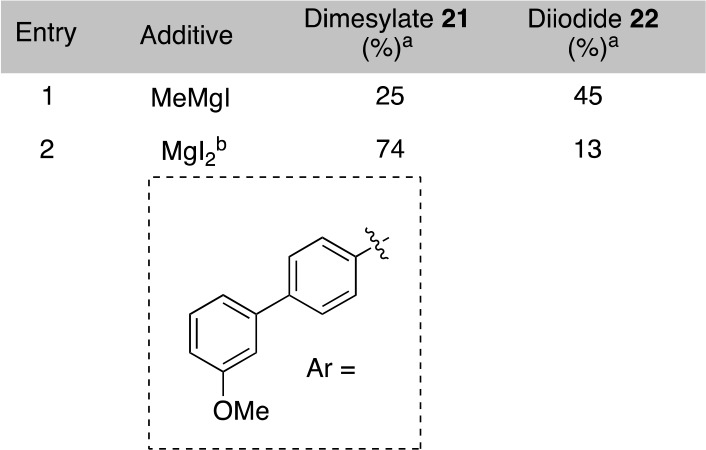
Comparison of MeMgI and MgI_2_ as Iodide Sources

a Yield determined by ^1^H NMR based on comparison to PhTMS as internal standard.

b 70 μL of Et_2_O added

**Scheme 4:**
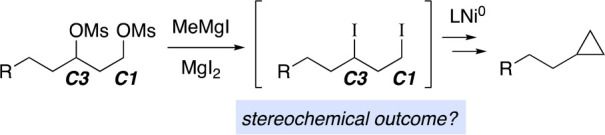
Stereochemical Outcome of Formation of 1,3-Diiodide

The mechanism of iodide formation has important implications for the stereochemical outcome of the reaction (Scheme 4). Since Grignard reagent is consumed upon formation of alkyl iodide, we hypothesized that subsequent epimerization of the alkyl iodide moieties by Walden-type inversion with MgI_2_ is unlikely.^20^ Therefore, formation of the alkyl iodide should occur with single inversion and be stereospecific. In particular, we sought to determine the stereochemical outcome of the primary center, carbon 1 (C1), since in the proposed catalytic cycle, all subsequent elementary steps involving this center are stereospecific.

**Scheme 5:**
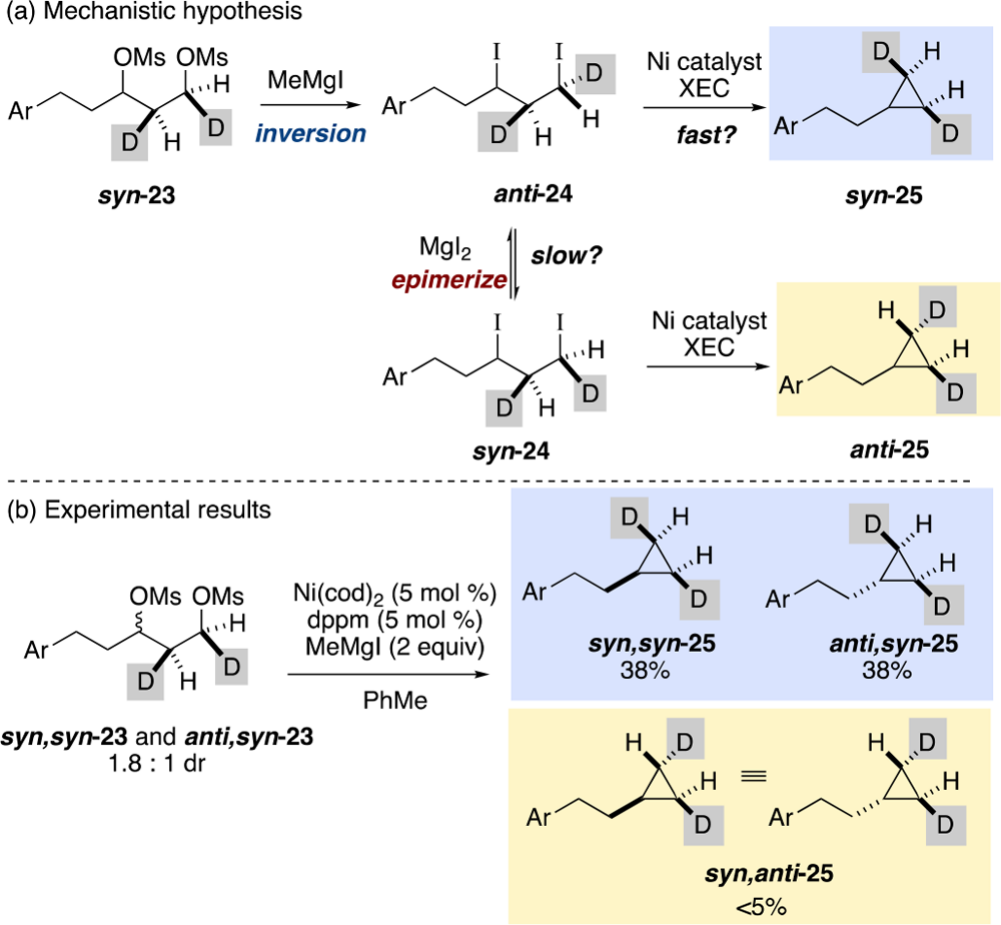
Synthesis of *Syn*-Dideuterated Cyclopropane **25**

To test this hypothesis, we designed an experiment to track the stereochemical outcome at the primary center (Scheme 5). A 1,3-dimesylate was labeled with *syn*-deuterium atoms. Assuming that Walden-type inversion with MgI_2_ is slow, MeMgI would convert *syn*-deuterium-labeled 1,3-dimesylate **23** into *anti*-1,3-diiodide **24** followed by the XEC reaction to generate only *syn*-deuterium-labeled cyclopropanes **25** (Scheme 5a). *Syn*-deuterium-labeled 1,3-dimesylate **23** was subjected to the standard reaction conditions to provide cyclopropane **25** with only *syn*-deuterium labels (Scheme 5b), supporting our hypothesis that epimerization by iodide displacement is slow. Importantly, this experiment also demonstrates that the reaction proceeds with net retention at the primary center, the result of double inversion (inversion in formation of the alkyl iodide, followed by inversion during cyclopropane formation).

In addition, we computationally investigated the possibility of racemization of formed 1,3-diiodide **20** facilitated by the attack of Mg(II) reagent. As shown in Scheme SI-9, this proposed pathway is less favorable than racemization facilitated by XAT at Ni(0). Therefore, the formed diiodide **20** directly enters into the nickel catalytic cycle to undergo the subsequent transformations.

Both experimental and computational results demonstrate that conversion of the alkyl mesylates to the alkyl iodides occurs via a single invertive S_N_2 displacement reaction. This mechanistic understanding is useful in development of a stereospecific synthesis of alkyl iodides.^21^ Furthermore, the overall stereoablative reaction at the secondary center is not due to epimerization reactions of the alkyl iodide but instead due to subsequent nickel-catalyzed steps.

### Experimental Study of the Lifetime of the Alkyl Radical

**Scheme 6:**
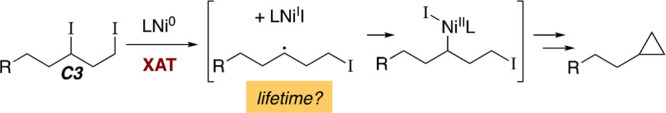
Formation and Capture of Alkyl Radical

Alkyl radicals, formed by XAT of alkyl halides with nickel complexes, are key intermediates in many XC and XEC reactions.^22^ The lifetimes of these radical intermediates have important implications for these reactions, including selectivity and overall mechanism, for example, whether the reaction proceeds through a radical chain versus sequential reduction mechanism.^23^ We thought that this reaction provided an opportunity to interrogate the recombination event. A series of substrates containing radical clocks were designed in order to provide boundary conditions for the lifetime of the secondary alkyl radical intermediate (Scheme 6).

**Scheme 7:**
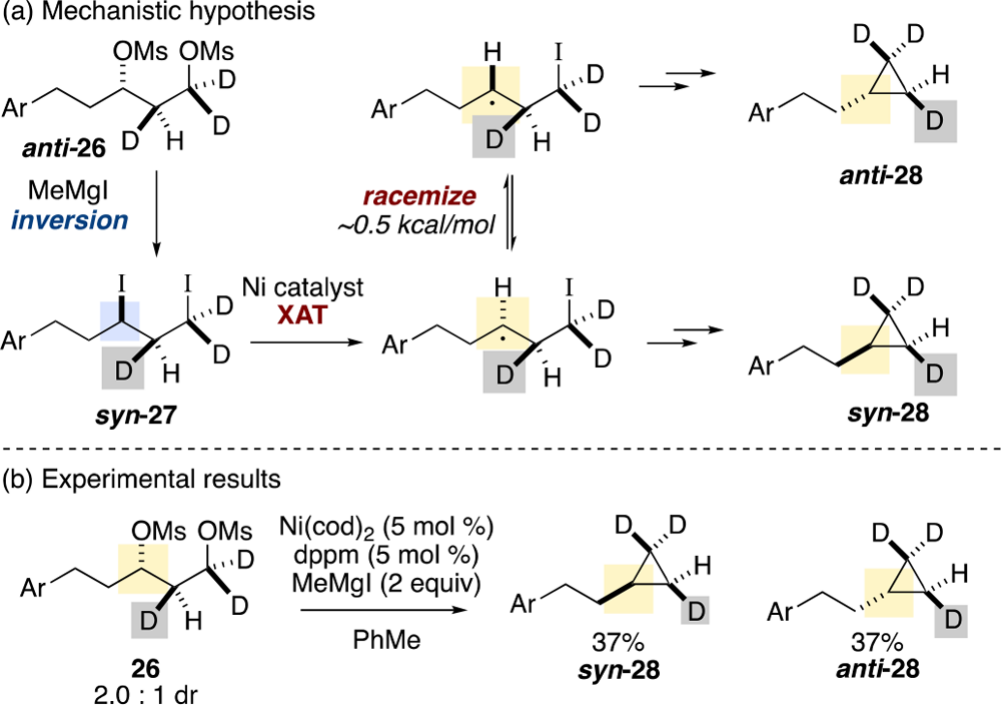
Synthesis of Trideuterated Cyclopropane **28**

To determine the lifetime of the alkyl radical at carbon 3 (C3), we began with trideuterated substrate **26**. This substrate was designed to establish that the reaction is stereoablative with respect to the secondary center, in the absence of a steric preference for formation of *trans*-cyclopropanes (Scheme 7a). Trideuterated 1,3-dimesylate **26** was synthesized as a 2.0:1 mixture of diastereomers; subjecting **26** to the standard conditions provided *syn*-**28** and *anti*-**28** in a 1:1 ratio (Scheme 7b). This result confirms that the reaction is stereoablative with respect to C3. Furthermore, this experimental evidence establishes that the rate of capture of the alkyl radical is sufficiently slower than the rate of epimerization of the alkyl radical (Δ*G*^‡^ = 0.5 kcal/mol).^24^

**Scheme 8:**
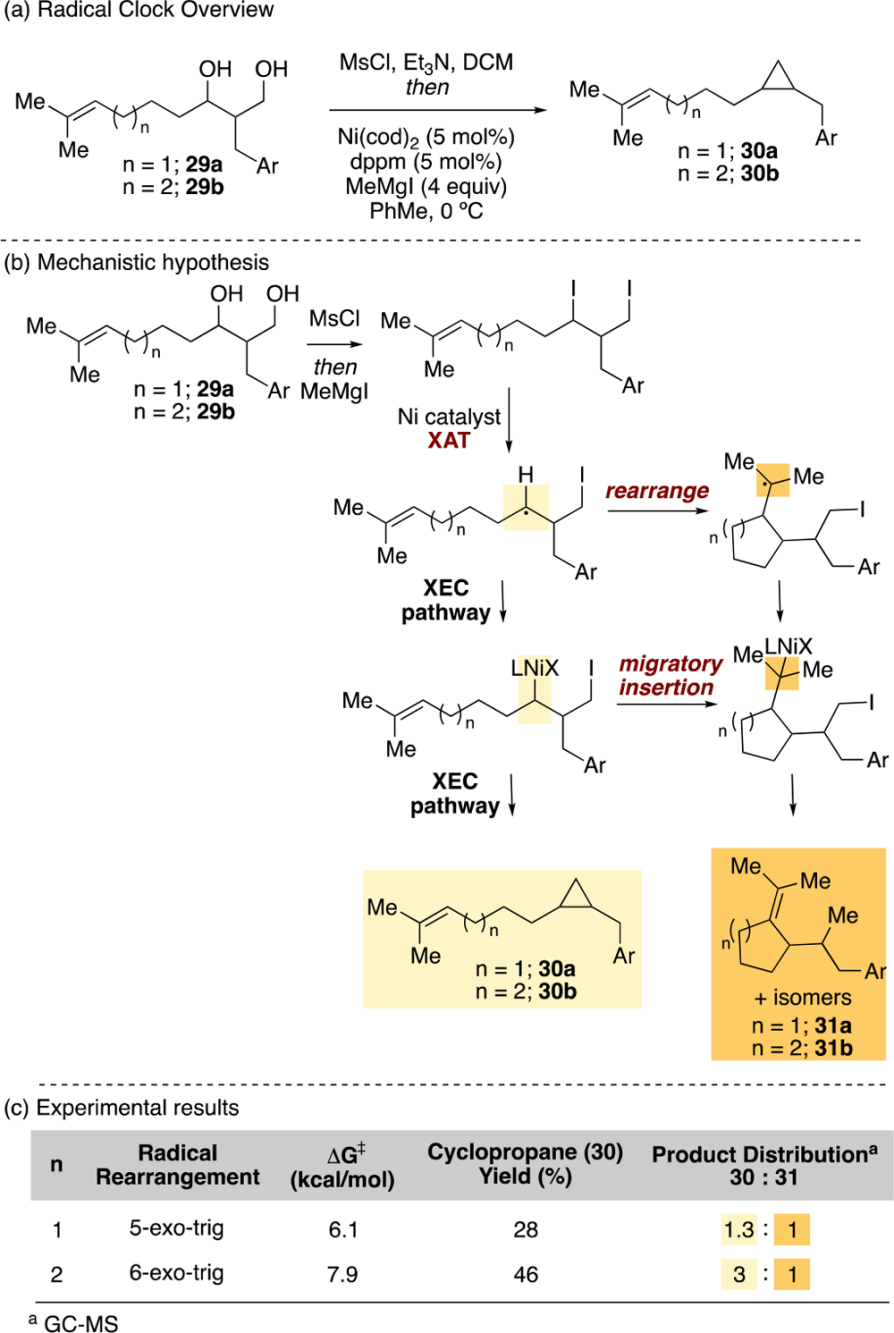
Radical Clock Experiments

Next, we designed a series of substrates containing radical clocks to further refine our understanding of the lifetime of the alkyl radical (Scheme 8).^25,26^ 1,3-Diols **29a** and **29b** compare the rate of capture of the alkyl radical along the XEC pathway with 5-exo-trig and 6-exo-trig cyclization (Scheme 8b). When subjected to our standard conditions, substrate **29a** provided a 1.3:1 mixture of cyclopropane **30a** to cyclopentyl products **31a** resulting from 5-exo-trig cyclization (Scheme 8c, entry 1). In contrast, substrate **29b**, which compares the rate of the XEC pathway with 6-exo-trig cyclization, provided a 3:1 mixture of cyclopropane **30b** to cyclohexyl products **31b** (Scheme 8c, entry 2). Based on these results, we can infer that consumption of the 2° radical intermediate along the XEC pathway is competitive with 5-exo-trig cyclization (1.0 × 10^5^ s^–1^) and faster than 6-exo-trig cyclization (5.4 × 10^3^ s^–1^). Notably, these experiments provide a *lower limit* for the rate of consumption of the 2° alkyl radical to generate the secondary organonickel intermediate, since cyclized products can also be formed by migratory insertion of the organonickel intermediate later in the pathway.^27,28^ Computational approaches were examined to further delineate these pathways.

### Computational Study of Ni-Catalyzed XEC Reaction

**Figure 3:**
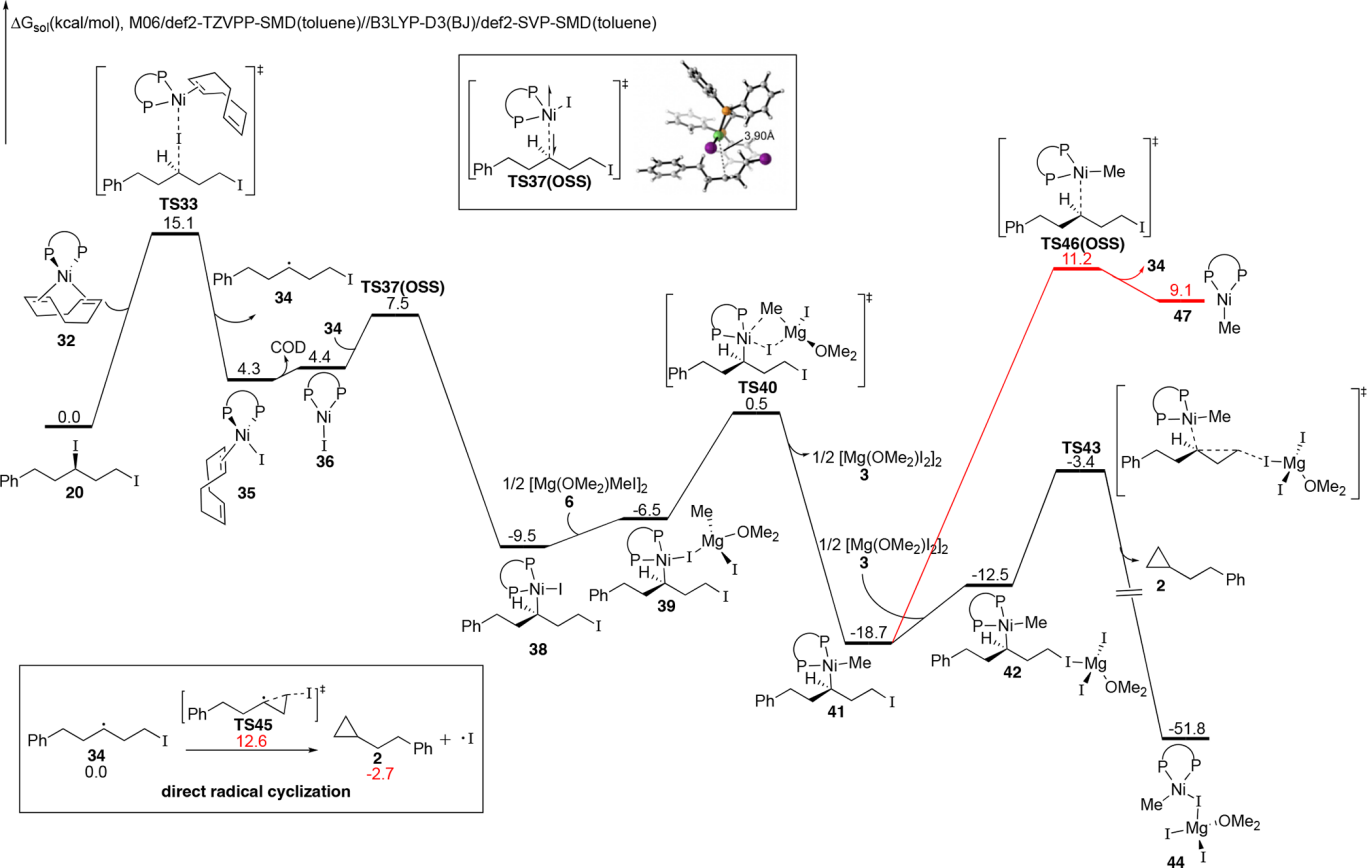
DFT-computed free energy changes of Ni/dppm-catalyzed XEC with 1,3-diiodide **20** (product formation).

Based on the proposed catalytic cycle (Scheme 2) and experimental results (Schemes 5–8), we computationally studied the reaction profile of the Ni/dppm-catalyzed XEC reaction with 1,3-diiodide **20**. The free energy changes of the most favorable pathway are shown in Figure 3. Starting from 1,3-diiodide **20**, XEC reaction begins with iodine atom abstraction at the 2° alkyl iodide moiety via an open shell singlet transition state, **TS33**, generating 2° alkyl radical **34** and Ni(I) species, **35**. From **35**, dissociation of 1,5-cyclooctadiene (COD) produces intermediate **36**, which recombines with alkyl radical **34** to form organonickel species **38** through **TS37**. **TS37** is also an open shell singlet transition state (Scheme SI-10), and the intrinsic barrier calculated from the energy difference between **TS37** and preceding intermediates (**34** and **36**) is 3.1 kcal/mol.

An alternative pathway for 2° alkyl radical **34** is, instead of capture to form alkylnickel complex **38**, direct intramolecular S_H_2 radical cyclization to generate cyclopropane **2** (Figure 3, inset).^26,29,30^ Indeed, our experimental evidence does not distinguish between a radical-mediated or organonickel-mediated formation of the cyclopropane ring. However, computationally we determined that the direct intramolecular S_H_2 radical cyclization of **34** via **TS45** is less favorable, with a barrier of 12.6 kcal/mol,^31^ as compared to the radical capture by LNi^I^I **36** (3.1 kcal/mol via **TS37**). We considered both the stereoinvertive and stereoretentive radical cyclization processes, and the stereoinvertive transition state **TS45** is more favorable (Figure SI-3). The proposed single electron transfer (SET) process between Ni(I) species (**35** or **36**) and the secondary alkyl radical (**34**) is not feasible in this studied reaction system (Scheme SI-11). Therefore, the intermediate 2° radical **34** recombines with Ni(I) intermediate **36**, generating the stable closed shell singlet species **38** as the product of formal oxidative addition.

Subsequent elementary steps transform secondary alkylnickel intermediate **38** to the observed product, cyclopropane **2**. Association of an equivalent Grignard reagent can form intermediate **39**, from which transmetalation occurs with an inner sphere mechanism via **TS40**, generating (alkyl)Ni(Me) species **41**. An alternative less favored transmetalation pathway is included in the Supporting Information (Figure SI-4). Subsequent complexation with MgI_2_ produces **42**, which undergoes intramolecular S_N_2-type reaction via **TS43** to generate cyclopropane product **2**, as well as Ni(II) species **44**. Starting from intermediate **41**, homolysis of the nickel–carbon bond can also occur through **TS46** (an open shell singlet transition state, Scheme SI-10) to regenerate secondary alkyl radical **34** and Ni(I) species **47**, but this hypothetical path is very unfavorable, with an energy barrier of 29.9 kcal/mol (from **41** to **TS46**). The rationalization of such a high energy barrier is provided in Figure SI-5 and Scheme SI-12. Therefore, organometallic intramolecular S_N_2-type reaction via **TS43** occurs effectively to generate the new C–C bond in product **2**.^15c^ Different computational methods (Figure SI-6 and Figure SI-7) were employed to investigate the reaction profile of product formation (Figure 3), and similar mechanistic scenery is obtained. The DFT-computed free energy diagram of catalyst regeneration is shown in Figure SI-8.

The reaction profile of product formation (Figure 3) indicates that after 1,3-diiodide **20** goes through XAT to generate secondary alkyl radical **34**, the energy barrier for Ni(I) (**36**) to bind with alkyl radical (**34**) via **TS37** is 3.1 kcal/mol. Generally, the energy barrier for epimerization of alkyl radicals is less than 0.5 kcal/mol;^24^ therefore, the generated alkyl radical **34** has a sufficient lifetime to racemize. The combination of Ni(I) complex **36** and alkyl radical **34** via **TS37** leads to the formation of a stable closed shell singlet species **38**, and the subsequent intramolecular S_N_2-type reaction is stereospecific. Thus, this mechanism explains the stereoablative reaction outcome at carbon 3, consistent with the experimentally observed formation of both epimers of trideuterated cyclopropane **28**.

**Scheme 9:**
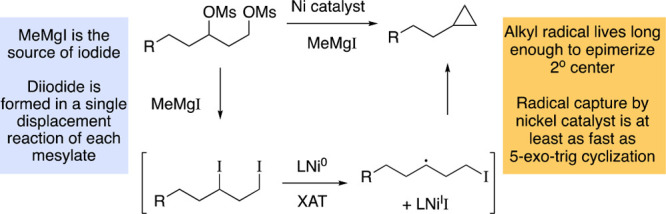
Proposed Mechanism and Conclusions

In conclusion, we present a combination of control experiments, labeling studies, radical clock experiments, and DFT calculations to determine the key mechanistic features of a nickel-catalyzed XEC reaction of alkyl mesylates (Scheme 9). In particular, we demonstrate that one role of the Grignard reagent is to convert alkyl mesylates to alkyl iodides in situ. This step occurs with clean inversion and does not suffer from racemization. The overall stereoablative reaction outcome at carbon 3 is due to subsequent nickel-catalyzed halogen atom abstraction, which provides an alkyl radical that rapidly epimerizes. Subsequent radical capture, to provide an organonickel intermediate that proceeds forward, is faster than S_H_2 cyclization of the alkyl radical and competitive with 5-exo-trig cyclization. Future directions include designing new nickel-catalyzed transformations that are informed by this improved mechanistic understanding.

## Supporting Information Available

Full experimental procedures and characterization data as well as copies of ^1^H NMR spectra for each compound and HRMS data of deuterium incorporation; full details of computational results (PDF)
