# Supplementary material for: A Nickel-Catalyzed Cross-Electrophile Coupling Reaction of 1,3-Dimesylates for Alkylcyclopropane Synthesis: Investigation of Stereochemical Outcomes and Radical Lifetimes
Source: ACS Catal. 2023 Apr 7;13(8):5472–81. doi: 10.1021/acscatal.3c00905 (PMC10127265; doi:10.1021/acscatal.3c00905)
Supplement: Supplementary file 1 — cs3c00905_si_001.pdf [file cs3c00905_si_001.pdf]

Supporting Information

A Nickel-Catalyzed Cross-Electrophile Coupling Reaction of 1,3-Dimesylates for Alkylcyclopropane Synthesis: Investigation of Stereochemical Outcomes and Radical Lifetimes

Pan-Pan Chen,[‡,‖] Tristan M. McGinnis,[†,‖] Patricia C. Lin,[†] Xin Hong,[*,‡] Elizabeth R. Jarvo[*,†]
[†]Department of Chemistry, University of California, Irvine, California 92697-2025, United States
[‡]Department of Chemistry, Zhejiang University, Hangzhou 310027, China
[‖]These authors contributed equally.

[*]Corresponding authors: hxchem@zju.edu.cn; erjarvo@uci.edu;

Supporting Information

**I.**	**General Procedures**	SI-2
**II.**	**Experimental Methods**	SI-4
	Method A: Reduction of Carboxylic Acid	SI-4
	Method B: Suzuki-Miyaura Cross-Coupling Reaction	SI-4
	1) Suzuki-Miyaura Cross-Coupling Reaction with $Pd(PPh_3)_4$	SI-4
	2) Suzuki-Miyaura Cross-Coupling Reaction with $Pd(OAc)_2$	SI-4
	Method C: DMP Oxidation of Primary Alcohol to Aldehyde	SI-5
	Method D: Grignard Addition into Aldehydes	SI-5
	Method E: Hydroboration/Oxidation	SI-5
	Method F: Mesylation of Alcohols	SI-5
	Method G: Cross-Electrophile Coupling (XEC) for Synthesis of Alkylcyclopropanes	SI-6
	1) Preparation of Methyl Grignard Reagent	SI-6
	2) XEC of 1,3-Dimesylate Generated In Situ from 1,3-Diols	SI-6
	Method H: Deprotonation of Alkyne and Quench with D_2O	SI-7
	Method I: $LiAlD_4$ Reduction of Alkyne and Quench with D_2O	SI-7
	Method J: Alkylation of Methyl Acetoacetate	SI-7
	Method K: Benzylation of β-Ketoester with Benzylic Bromide	SI-7
	Method L: $LiAlH_4$ Reduction	SI-8
**III.**	**Synthesis of 1,3-Dimesylate 21 and Experimental Data for Iodination Employing MgI_2 or MeMgI**	SI-9
	a. Synthesis of 1,3-Dimesylate **21**	SI-9
	b. Synthesis of Cyclopropane **SI-6**	SI-11
	c. Iodination Employing MgI_2 and MeMgI	SI-12
**IV.**	**Experimental Data and Synthesis for Deuterated Cyclopropanes**	SI-13
	a. Synthesis of Dideuterated 1,3-Dimesylate **23**	SI-13
	b. Nickel-catalyzed Intramolecular XEC to form Dideuterated Cyclopropane **25**	SI-19
	c. Synthesis of Trideuterated 1,3-Dimesylate **26**	SI-21
	d. Nickel-catalyzed Intramolecular XEC to form Trideuterated Cyclopropane **28**	SI-29

V. **Experimental Data and Synthesis for the 5-Exo-Trig and 6-Exo Trig Clock Substrates** SI-32
a. Synthesis of 5-Exo-Trig Clock Diol **29a** SI-32
b. Nickel-catalyzed intramolecular XEC of 5-Exo-Trig Clock Diol **29a** SI-35
c. Synthesis of 6-Exo-Trig Clock Diol **29b** SI-36
d. Nickel-catalyzed intramolecular XEC of 6-Exo-Trig Clock Diol **29b** SI-39
VI. **Computational Data** SI-40
a. Conformational Analysis SI-40
b. Complexation between Dimesylate and Mg(II) Species SI-40
c. Origins of the Regioselectivity for the First Iodide Displacement SI-42
d. Free Energy Diagram of MgI_2-mediated Iodide Displacement of Dimesylate **1** SI-43
e. Exploration of the Possibility of 1,3-Diiodide Racemization SI-44
f. Discussions on Different Spin States of Key Transition States and Intermediates SI-45
g. Alternative Transition States of Radical Cyclization SI-46
h. Explorations of the Possibility of Single Electron Transfer Process SI-46
i. Explorations of the Alternative Transmetalation Pathways SI-47
j. Discussions on the C–Ni Bond Homolysis Process SI-48
k. Computational Investigation of the Reaction Profile for Product Formation by Using Different Methods SI-49
l. Reaction Profile of Catalyst Regeneration SI-50
m. Table of Energies SI-51
VII. **References for Supporting Information** SI-55
VIII. **Cartesian Coordinates of Calculated Structures** SI-57
IX. **^{1}H, ^{13}C NMR Spectra** SI-117

I. General Procedures

All reactions were carried out under an atmosphere of N_2 when noted. All glassware was oven- or flame-dried prior to use. Tetrahydrofuran (THF), diethyl ether (Et_2O), dichloromethane (DCM), acetonitrile (MeCN), dimethylformamide (DMF), and toluene (PhMe) were degassed with Ar and then passed through two 4 x 36-inch columns of anhydrous neutral A-2 alumina (8 x 14 mesh; LaRoche Chemicals; activated under a flow of argon at 350 °C for 12 h) to remove H_2O. All other solvents utilized were purchased "anhydrous" commercially or purified as described. ^{1}H NMR spectra were recorded on Bruker DRX-400 (400 MHz ^{1}H, 100 MHz ^{13}C), GN-500 (500 MHz ^{1}H, 125.7 MHz ^{13}C), or CRYO-500 (500 MHz ^{1}H, 125.7 MHz ^{13}C) spectrometers. Proton chemical shifts are reported in ppm (δ) relative to internal tetramethylsilane (TMS, δ 0.00). Data are reported as follows: chemical shift (multiplicity [singlet (s), broad singlet (br s), doublet (d), doublet of doublets (dd), doublet of doublet of doublets (ddd), triplet (t), doublet of triplets (dt), doublet of quartets (dq), triplet of doublets (td), quartet (q), quintet (quint), multiplet (m), apparent singlet (ap s), apparent doublet (ad), apparent triplet (at), apparent quartet (aq), apparent quintet (aquint)], coupling constants [Hz], integration). Carbon chemical shifts are reported in ppm (δ) relative to TMS with the respective solvent resonance as the internal standard ($CDCl_3$, δ 77.16 ppm). NMR data were collected at 25 °C. Infrared (IR) spectra were obtained on a Thermo Scientific Nicolet iS5 spectrometer with an iD5 ATR tip (neat) and are reported in terms of frequency of absorption

(cm^{-1}). Analytical thin-layer chromatography (TLC) was performed using Silica Gel 60 F254 precoated plates (0.25 mm thickness). Visualization was accomplished by irradiation with a UV lamp. Flash chromatography was performed using SiliaFlash F60 (40-63 μm, 60 Å) from SiliCycle. Melting points (m.p.) were obtained using a MelTemp melting point apparatus and are uncorrected. High resolution mass spectrometry was performed by the University of California, Irvine Mass Spectrometry Center.

Bis(1,5-cyclooctadiene)nickel was purchased from Strem, stored in a glove box freezer (–20 °C) under an atmosphere of N_2 and used as received. All ligands were purchased from Strem or Sigma Aldrich and were stored under N_2 atmosphere and used as received. The methylmagnesium iodide was titrated with iodine prior to use. All other chemicals were purchased commercially and used as received, unless otherwise noted.

II. Experimental Methods

Method A: Reduction of Carboxylic Acid

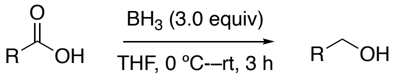

The target compound was prepared using a modified procedure reported by Cole.[1] A flame-dried round bottom flask equipped with stir bar was charged with carboxylic acid (1.0 equiv), and anhydrous THF (1.0 M) under N_2 atmosphere. The reaction mixture was cooled to 0 ºC and BH_3•THF (3.0 equiv) was added slowly via syringe. The reaction mixture was allowed to stir for at least 3 h before warming to rt. To quench, glacial acetic acid was added dropwise until reaction mixture stopped bubbling. The reaction mixture was diluted slowly with saturated aqueous $NaHCO_3$ (10 mL) and the aqueous layer was extracted with EtOAc (3 x 20 mL). The combined organic layers were washed with brine, dried over $MgSO_4$, and concentrated in vacuo.

Method B: Suzuki-Miyaura Cross-Coupling Reaction

1) Suzuki-Miyaura Cross-Coupling Reaction with $Pd(PPh_3)_4$

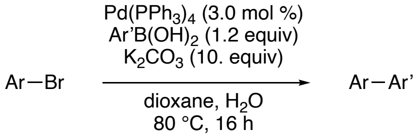

The target compound was prepared using a modified procedure reported by Nagano.[2] A two-neck round bottom flask was equipped with a reflux condenser and stir bar. Aryl bromide (1.0 equiv), $Pd(PPh_3)_4$ (3.0 mol %), Ar'–$B(OH)_2$ (1.2 equiv), K_2CO_3 (10. equiv), and dioxane/H_2O (4:1 ratio, 0.1 M) were added under N_2. The reaction mixture was allowed to stir at reflux overnight. Once complete, H_2O (10 mL) was added. The organic layer was separated, and the aqueous layer was then extracted with EtOAc (3 x 20 mL). The combined organic layers were washed with brine, dried over Na_2SO_4, and concentrated in vacuo.

2) Suzuki-Miyaura Cross-Coupling Reaction with $Pd(OAc)_2$

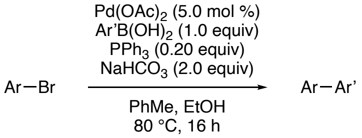

The target compound was prepared using a modified procedure reported by Walton.[3] To a flame-dried round-bottom flask equipped with a stir bar, aryl bromide (1.0 equiv), boronic acid (1.0 equiv), $Pd(OAc)_2$ (5.0 mol %), PPh_3 (0.20 equiv), $NaHCO_3$ (2.0 equiv), PhMe (0.20 M in substrate), and EtOH (13 mL) were added. The reaction was heated to 80 °C and allowed to stir for 16 h. H_2O was added to quench, and the reaction mixture was extracted with EtOAc (3 x 20 mL). The combined organic layers were washed with brine, dried over Na_2SO_4, and concentrated in vacuo.

Method C: DMP Oxidation of Primary Alcohol to Aldehyde

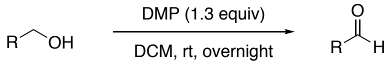

The target compound was prepared using a modified procedure reported by Fernandes.[4] A flame-dried round bottom flask equipped with stir bar was charged with alcohol (1.0 equiv), and DCM (0.2 M). To the reaction flask was added Dess-Martin periodinane (DMP; 1.3 equiv) in one portion. The reaction mixture was stirred overnight. To quench, saturated $NaHCO_3$ (10 mL) was added and the organic layer was separated. The aqueous layer was extracted with DCM (3 x 20 mL). The combined organic layers were washed with brine, dried over Na_2SO_4, and concentrated in vacuo.

Method D: Grignard Addition into Aldehydes

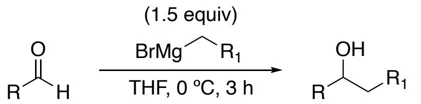

A flame-dried flask with a stir bar was charged with organomagnesium reagent (1.5 equiv) and cooled to 0 ºC. A solution of aldehyde (1.0 equiv) in anhydrous THF was added in a dropwise. The reaction mixture was stirred at room temperature for at least 3 h. The reaction was quenched with saturated aqueous NH_4Cl (10 mL) and the mixture was extracted with EtOAc (3 x 20 mL). The combined organic layers were washed with brine, dried over Na_2SO_4, and concentrated in vacuo.

Method E: Hydroboration/Oxidation

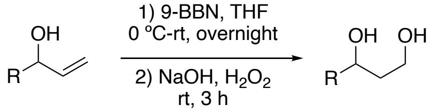

The target compound was prepared using a modified procedure reported by Hartwig.[5] A round bottom flask equipped with stir bar was charged with alkene (1.0 equiv) and THF (0.40 M). The flask was cooled to 0 ºC, and 9-BBN•THF (2.5 equiv) was added slowly. The reaction mixture was then warmed to rt and stirred overnight. Then, MeOH (3 mL/mmol), H_2O_2 (30% w/w, 1 mL/mmol) and NaOH (3.0 M, 1 mL/mmol) were added, and the reaction stirred for at least 3 h. Once complete, H_2O (10 mL) was added. The reaction mixture was then extracted with EtOAc (3 x 20 mL) and combined organic layers were washed with brine, dried over $MgSO_4$, and concentrated in vacuo.

Method F: Mesylation of Alcohols

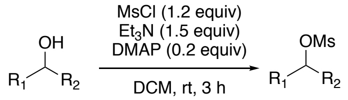

A round bottom flask equipped with a stir bar was charged with DMAP (0.2 equiv). Then, under N_2, alcohol (1.0 equiv), DCM (0.20 M), Et_3N (1.5 equiv), and MsCl (2.2 equiv) were added sequentially. The reaction mixture was then stirred at rt for at least 3 h. Once complete by TLC, sat. aqueous $NaHCO_3$ (5 mL) was added and the reaction mixture was extracted with DCM (3 x 10 mL). The combined organic layers were washed with brine, dried over Na_2SO_4, and concentrated in vacuo.

Method G: Cross-Electrophile Coupling (XEC) for Synthesis of Alkylcyclopropanes

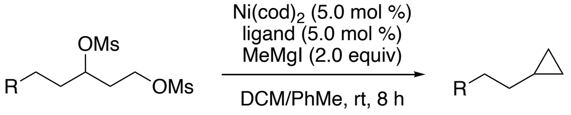

In a glovebox, a flame-dried 7 mL vial equipped with a stir bar was charged with substrate (1.0 equiv), $Ni(cod)_2$ (5.0 mol %), ligand (5.0 mol %), and PhMe (0.10 M in substrate). If substrate was still a precipitate once solvent was added, DCM (0.1 mL) was added and the reaction was stirred until substrate was dissolved, usually ~20 min. Once reaction mixture was homogenous, methylmagnesium iodide (2.0 equiv) was added added slowly over 15–20 seconds. The reaction stirred at rt for 8 h unless otherwise noted. Then the reaction was removed from the glovebox, quenched with MeOH (2 mL), filtered through a plug of silica gel (eluting with 100% Et_2O), and concentrated in vacuo.

1) Preparation of Methyl Grignard Reagent

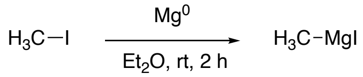

Under a N_2 atmosphere, a three-necked flask equipped with a stir bar, reflux condenser, and Schlenk filtration apparatus was charged with magnesium turnings (1.1 g, 45 mmol). The flask and magnesium turnings were then flame-dried under vacuum, and the flask was back-filled with N_2. Anhydrous Et_2O (7.0 mL) and a crystal of iodine (ca. 2.0 mg) were added to the flask. Freshly distilled iodomethane (1.9 mL, 31 mmol) was slowly added over 30 min to maintain a gentle reflux. The mixture was stirred for 2 h at room temperature then filtered through the fritted Schlenk filter into a Schlenk flask under N_2 atmosphere. The magnesium turnings were washed with Et_2O (2 x 1.0 mL) then the Schlenk flask was sealed, removed, and placed under an N_2 atmosphere. The resulting methylmagnesium iodide was typically between 2.4 and 3.0 M as titrated by Knochel's method[6] and could be stored, sealed under N_2 atmosphere or in a glovebox, for 8–12 weeks.

2) XEC of 1,3-Dimesylate Generated In Situ from 1,3-Diols

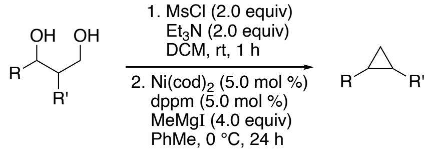

An oven-dried 7-mL vial equipped with a stir bar was charged with 1,3-diol (1.0 equiv), DCM (0.20 M in substrate), and anhydrous Et_3N (2.0 equiv) under N_2 at rt. The reaction was stirred for 5 min before MsCl (2.0 equiv) was added, and then allowed to stir for 1 h. In a glovebox, a separate oven-dried 7-mL vial was charged with $Ni(cod)_2$ (5.0 mol %), dppm (5.0 mol %), and PhMe (0.20 M in substrate). MeMgI (4.0 equiv) was drawn up into a syringe and put through the septum in a third 7-mL vial. Both the vial and MeMgI were removed from the glovebox and placed under N_2. The reaction mixture was transferred via syringe to the vial containing $Ni(cod)_2$ and dppm, and both the reaction mixture and MeMgI were cooled to 0 °C. After 15 min, MeMgI was added slowly to the stirring reaction mixture over 15–20 seconds. Upon addition of MeMgI, the reaction was vented. The reaction was allowed to stir at 0 °C for 24 h. The reaction was then quenched with methanol, filtered through a plug of silica gel (eluted with 100% Et_2O), and concentrated in vacuo.

Method H: Deprotonation of Alkyne and Quench with D_2O

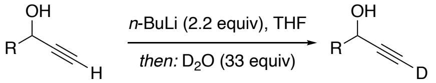

A round-bottom flask equipped with stir bar was charged with alkyne (1.0 equiv) and THF (0.20 M) under N_2. The reaction mixture was cooled to 0 ºC. Then, *n*-BuLi (2.2 equiv) was added, and the reaction was stirred for 1 h at 0 ºC. D_2O (33 equiv) was added dropwise and allowed to stir for 1 hour and warmed to rt. H_2O was then added and the reaction mixture was extracted with EtOAc (3 x 10 mL). The combined organic layers were washed with brine, dried over Na_2SO_4, and concentrated in vacuo.

Method I: $LiAlD_4$ Reduction of Alkyne and Quench with D_2O

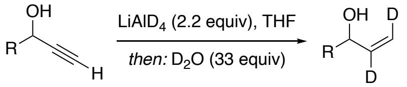

In a glovebox, to a round bottom flask equipped with a stir bar was added $LiAlD_4$ (2.2 equiv). The flask was capped with a septum and removed from the glovebox and diluted in THF (0.20 M in substrate). At rt, the alkyne (1.0 equiv) was added dropwise in THF and allowed to stir for 16 h. D_2O (33 equiv) was then added and allowed to stir for 1 h. The reaction was then diluted with H_2O and extracted with EtOAc (3 x 10 mL). The combined organic layers were washed with brine, dried over Na_2SO_4, and concentrated in vacuo.

Method J: Alkylation of Methyl Acetoacetate

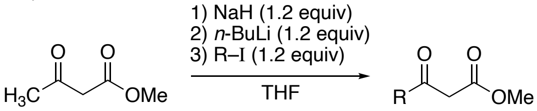

This method was adapted from a procedure reported by Fürstner.[7] In a glovebox, a flame-dried round-bottom flask was charged with NaH (1.2 equiv). The flask was sealed with a septum, removed from the glovebox, and placed under N_2. Anhydrous THF (0.20 M in substrate) and methyl acetoacetate (1.0 equiv) were added, and a vent needle was inserted to allow the H_2 gas to vent. The reaction mixture was allowed to stir at rt for 30 min. Following the 30 min, the reaction flask was cooled to –78 °C and allowed to stir for 5 min before *n*-BuLi (1.2 equiv, 2.5 M in hexanes) was added dropwise. After stirring at –78 °C for an hour, alkyl iodide (1.2 equiv) was added dropwise. The reaction mixture was warmed to 0 °C and allowed to stir for 30 min and at rt for another 30 min. To quench, saturated aqueous NH_4Cl (10 mL) was added. The reaction mixture was extracted with Et_2O (3 x 25 mL), and the combined organic layers were washed with brine, dried over Na_2SO_4, and concentrated in vacuo.

Method K: Benzylation of β-Ketoester with Benzylic Bromide

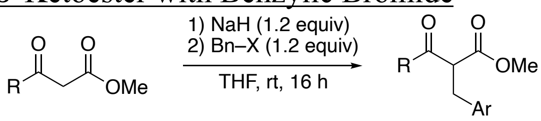

This method was adapted from a procedure reported by Maruoka.[8] In a glovebox, a flame-dried round-bottom flask equipped with a stir bar was charged with NaH (1.2 equiv). The flask was sealed with a septum, removed from the glovebox, and placed under N_2. Anhydrous THF (0.20 M in substrate) and β-ketoester (1.0 equiv, 0.10 M in THF) were added, and the reaction mixture was allowed to stir at rt for 1 h. Benzylic bromide (1.2 equiv, 0.10 M in THF) was added, and the

reaction mixture was allowed to stir at rt for 16 h. To quench, saturated aqueous NH_4Cl was added. The reaction mixture was extracted with Et_2O (3 x 10 mL), and the combined organic layers were washed with brine, dried over Na_2SO_4, and concentrated in vacuo.

Method L: $LiAlH_4$ Reduction

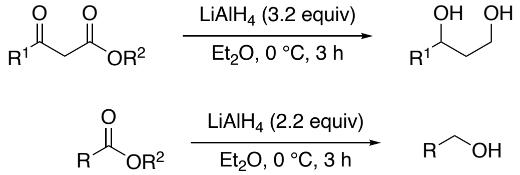

In a glovebox, a flame-dried round-bottom flask equipped with a stir bar was charged with $LiAlH_4$ (2.2–3.2 equiv). The flask was sealed with a septum, removed from the glovebox, and placed under N_2. Anhydrous THF (0.20 M in substrate) was added, and the reaction flask was cooled to 0 °C and allowed to stir for 5 min. Then, carbonyl substrate (1.0 equiv, 0.10 M in THF) was added, and the reaction mixture was allowed to stir at 0 °C for 2 h. To quench, saturated NH_4Cl was added at 0 °C, and the reaction mixture was allowed to warm to rt. 1 M HCl (2 mL) was added to the reaction mixture and extracted with Et_2O (3 x 20 mL). The combined layers were washed with brine, dried over Na_2SO_4, and concentrated in vacuo.

III. Synthesis of 1,3-Dimesylate 21 and Experimental Data for Iodination Employing MgI_2 or MeMgI

a) Synthesis of 1,3-Dimesylate **21**

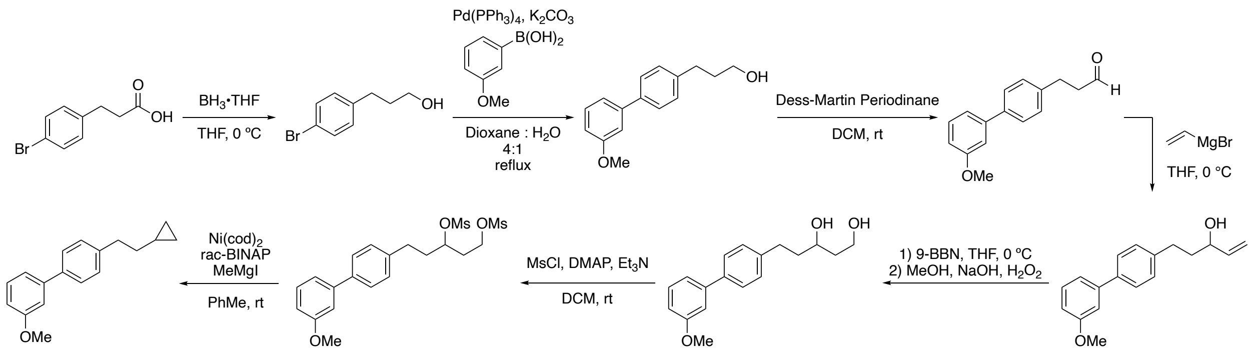

Scheme SI-1: Synthesis of 1,3 Dimesylate **21** and Cyclopropane **SI-6**

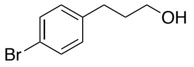

Alcohol SI-1 was prepared according to Method A. The following amounts of reagents were used: 3-(4-bromophenyl)propionic acid (2.3 g, 10. mmol, 1.0 equiv), BH_3•THF (30. mL, 30. mmol, 3.0 equiv, 1.0 M), and THF (20. mL, 0.50 M in substrate). The compound was purified by flash column chromatography (0–20% EtOAc/hexanes) to afford the title compound as a clear, colorless oil (2.1 g, 9.7 mmol, 97%). **^{1}H NMR** (400 MHz, $CDCl_3$) δ 7.39 (d, *J* =8.3 Hz, 2H), 7.07 (d, *J* =8.2 Hz, 2H), 3.66 (t, *J* =6.3 Hz, 2H), 2.66 (t, *J* =7.7 Hz, 2H), 1.86 (tt, *J* =7.8, 6.4 Hz, 2H), 1.42 (s, 1H). Analytical data is consistent with literature values.[9]

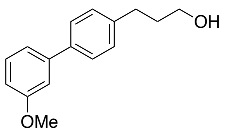

Alcohol SI-2 was prepared according to Method B1. The following amounts of reagents were used: alcohol **SI-1** (2.5 g, 12 mmol, 1.0 equiv), $Pd(PPh_3)_4$ (0.41 g, 0.35 mmol, 3.0 mol %), K_2CO_3 (16 g, 120 mmol, 10. equiv), 3-methoxyphenyl boronic acid (2.1 g, 14 mmol, 1.2 equiv), dioxane (60. mL, 0.20 M in substrate), and H_2O (15 mL). The compound was purified by flash column chromatography (0–30% EtOAc/hexanes) to afford the title compound as a clear yellow oil (2.8 g, 12 mmol, 77% yield).; **^{1}H NMR** (400 MHz, $CDCl_3$) δ 7.53 (d, *J* =8.2 Hz, 2H), 7.35 (t, *J* =7.8 Hz, 1H), 7.28 (d, *J* =8.4 Hz, 2H), 7.18 (d, *J* =7.9 Hz, 1H), 7.13 (s, 1H), 6.89 (d, *J* =6.8 Hz, 1H), 3.87 (s, 3H), 3.71 (br s, 2H), 2.77 (t, *J* =7.5 Hz, 2H), 1.95 (quint, *J* =7.5 Hz, 2H), 1.40 (s, 1H). Analytical data is consistent with literature values.[9]

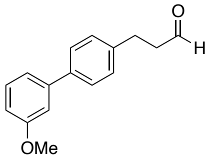

Alcohol SI-3 was prepared according to Method C. The following amounts of reagents were used: **SI-2** (2.8 g, 12 mmol, 1.0 equiv), DMP (5.5 g, 13 mmol, 1.1 equiv), and DCM (50 mL). The compound was purified by flash column chromatography (0–20% EtOAc/hexanes) to afford the title compound as a yellow oil (1.6 g, 6.8 mmol, 53% yield, contains 15% EtOAc by ^{1}H NMR); **^{1}H NMR** (400 MHz, $CDCl_3$) δ 9.86 (s, 1H), 7.53 (d, *J* =8.1 Hz, 2H), 7.35 (t, *J* =7.9 Hz, 1H), 7.28 (d, *J* =7.7 Hz, 2H), 7.17 (d, *J* =7.6 Hz, 1H), 7.11 (s, 1H), 6.90 (d, *J* =8.1 Hz, 1H), 3.87 (s, 3H), 3.02 (t, *J* =7.3 Hz, 2H), 2.83 (t, *J* =7.4 Hz, 2H). Analytical data is consistent with literature values.[9]

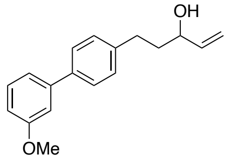

Alcohol SI-4 was prepared according to Method D. The following amounts of reagents were used: **SI-3** (0.79 g, 3.3 mmol, 1.0 equiv), vinyl magnesium bromide (5.0 mL, 5.0 mmol, 1.5 equiv, 1.0 M in THF), and THF (10 mL, 0.33 M in substrate). The compound was purified by flash column chromatography (0–20% EtOAc/hexanes) to afford the title compound as a yellow oil (0.88 g, 3.3 mmol, 99% yield). **^{1}H NMR** (400 MHz, $CDCl_3$) δ 7.51 (d, *J* = 8.1 Hz, 2H), 7.34 (t, *J* =7.9 Hz, 1H), 7.27 (d, *J* = 8.8 Hz, 2H), 7.17 (d, *J* = 8.0 Hz, 1H), 7.11 (s, 1H), 6.88 (dd, *J* =2.5, 8.3 Hz, 1H), 5.93 (ddd, *J* = 17.2, 10.5, 6.2 Hz, 1H), 5.26 (dt, *J* = 17.2, 1.3 Hz, 1H), 5.16 (dt, *J* = 10.5, 1.3 Hz, 1H), 4.17 (q, *J* = 6.3 Hz, 1H), 3.86 (s, 3H), 2.84–2.64 (m, 2H), 1.93–1.85 (m, 2H).

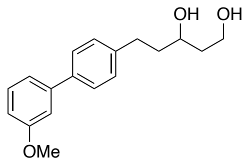

Diol SI-5 was prepared according to Method E. The following amounts of reagents were used: **SI-4** (0.84 g, 3.3 mmol, 1.0 equiv), 9-BBN (17 mL, 8.2 mmol, 2.5 equiv, 0.50 M in THF), THF (5.0 mL, 0.67 M in substrate), MeOH (10 mL), NaOH (5.0 mL, 3.0 M), and H_2O_2 (5.0 mL, 30% w/w). The compound was purified by flash column chromatography (0–60% EtOAc/hexanes) to afford the title compound as a white solid (0.91 g, 2.4 mmol, 74% yield). **^{1}H NMR** (500 MHz, C_6D_6) δ 7.52 (d, *J* = 8.2 Hz, 2H), 7.24 (s, 1H), 7.22–7.13 (m, 4H), 6.82–6.76 (m, 1H), 3.68 (s, 1H), 3.62 (s, 1H), 3.51 (s, 1H), 3.37 (s, 3H), 2.80–2.70 (m, 1H), 2.67–2.59 (m, 1H), 2.43–2.08 (br s, 2H), 1.78–1.67 (m, 1H), 1.64–1.56 (m, 1H), 1.54–1.45 (m, 1H), 1.39–1.31 (m, 1H); **^{13}C NMR** (125.7 MHz, C_6D_6) δ 160.7, 143.2, 141.9, 139.3, 130.1, 129.3 (2C), 127.6 (2C), 119.9, 113.2, 113.0, 71.1, 61.7, 54.8, 39.9, 38.9, 31.9. Analytical data is consistent with literature values.[9]

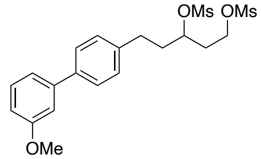

1,3-Dimesylate 21 was prepared by Method F. The following amounts of reagents were used: **SI-5** (0.16 g, 0.58 mmol, 1.0 equiv), Et_3N (0.24 mL, 1.7 mmol, 3.0 equiv), DMAP (14 mg, 0.12 mmol, 0.20 equiv), MsCl (0.10 mL, 1.3 mmol, 2.2 equiv), and DCM (6.0 mL, 0.10 M in substrate). The compound was purified by flash column chromatography (0–50% EtOAc/hexanes) to afford the title compound as a white solid (0.24 g, 0.55 mmol, 94%). **m.p.** = 71–72 ºC; **TLC** $\mathbf{R_f}$ = 0.7 (50% EtOAc/hexanes); ^{1}**H NMR** (400 MHz, $CDCl_3$) δ 7.53 (d, *J* = 8.1 Hz, 2H), 7.34 (t, *J* = 8.0 Hz, 1H), 7.27 (d, *J* = 8.1 Hz, 2H), 7.16 (d, *J* = 7.3 Hz, 1H), 7.10 (s, 1H), 6.89 (dd, *J* = 8.1, 2.5 Hz, 1H), 5.00–4.92 (m, 1H), 4.41–4.33 (m, 2H), 3.86 (s, 3H), 3.05 (s, 3H), 3.03 (s, 3H), 2.79 (t, *J* = 7.0 Hz, 2H), 2.22–2.07 (m, 4H); Analytical data is consistent with literature values.[9]

b) Synthesis of Cyclopropane **SI-6**

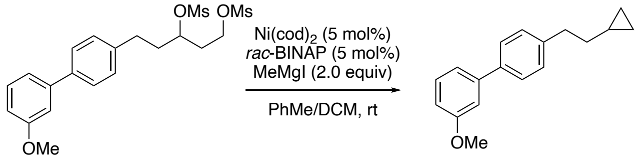

Cyclopropane SI-6 was prepared according to Method G. The following amounts of reagents were used: 1,3-dimesylate **21** (45 mg, 0.10 mmol, 1.0 equiv), $Ni(cod)_2$ (1.4 mg, 5.0 μmol, 5.0 mol %), *rac*-BINAP (3.7 mg, 6.0 μmol, 6.0 mol %), methylmagnesium iodide (0.070 mL, 0.20 mmol, 2.0 equiv, 2.9 M in Et_2O), DCM (0.20 mL, 0.50 M in substrate), and PhMe (1.0 mL, 0.10 M in substrate). A 73% yield was recorded by ^{1}H NMR in comparison to PhTMS as internal standard. **TLC** $\mathbf{R_f}$ = 0.7 (5% EtOAc/hexanes). ^{1}**H NMR** (500 MHz, $CDCl_3$) δ 7.52 (d, *J* =8.2 Hz, 2H), 7.25 (s, 1H), 7.20 (d, *J* =6.9 Hz, 2H), 7.13 (d, *J* =8.0 Hz, 2H), 6.81–6.78 (m, 1H), 3.36 (s, 3H), 2.64 (t, *J* =7.7 Hz, 2H), 1.43 (q, *J* =7.5 Hz, 2H), 0.64–0.57 (m, 1H), 0.42 (dt, *J* =8.0, 4.4 Hz, 2H), 0.05 (dt, *J* =5.1, 4.1 Hz, 2H); ^{13}**C NMR** (125.7 MHz, $CDCl_3$) δ 160.7, 143.3, 142.1, 139.3, 130.1, 129.3 (2C), 127.5 (2C), 119.9, 113.2, 113.0, 54.8, 37.0, 36.0, 11.1, 4.9 (2C); Analytical data is consistent with literature values.[5]

c) Iodination Employing MgI_2 or MeMgI

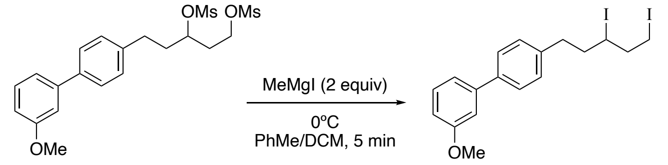

1,3-Diiodide 22 synthesized from MeMgI: 1,3-Dimesylate **21** (38 mg, 85 μmol, 1.0 equiv) was dissolved in PhMe (1.0 mL, 0.085 M in substrate) and DCM (0.1 mL) in an oven-dried 7-mL vial with a stir bar in the glovebox, capped with septum, and moved into an ice bath outside the glovebox. MeMgI (0.06 mL, 0.17 mmol, 2.0 equiv, 2.9 M in Et_2O) was added over 10 seconds and allowed to stir at 0 ºC for 5 minutes. This reaction was quenched with MeOH (1 mL) and filtered through a short plug of silica (eluting with 100% Et_2O). Phenyltrimethylsilane (PhTMS; 8.6 μL, 50. μmol) was added to determine the yield by ^{1}H NMR based on comparison to PhTMS as internal standard. A 45% yield of 1,3-diiodide **22** was observed by ^{1}H NMR. 25% 1,3-Dimesylate **21** was also observed by ^{1}H NMR.

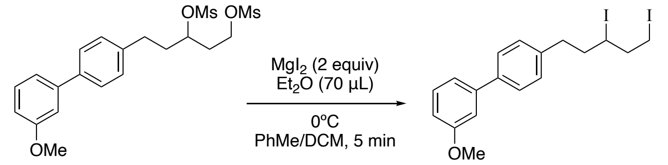

1,3 Diiodide 22 synthesized from MgI_2: MgI_2 (55 mg, 0.20 mmol, 2.0 equiv) was weighed into a 7mL vial equipped with stir bar and wrapped in aluminum foil in a glovebox. 1,3-Dimesylate **21** (43 mg, 98 μmol, 1.0 equiv) was dissolved in PhMe (1.0 mL, 0.098 M in substrate), DCM (0.1 mL), and Et_2O (70 μL) in a separate 7mL scintillation vial in a glovebox. Both vials were capped with septa before moving to an ice bath outside the glovebox. The solution of 1,3-dimesylate **21** was added quickly to the vial with MgI_2 and allowed to stir at 0 ºC for 5 minutes. This reaction was then filtered through a short plug of silica (eluting with 100% Et_2O). Phenyltrimethylsilane (PhTMS; 8.6 μL, 50. μmol) was added to determine the yield by ^{1}H NMR based on comparison to PhTMS as internal standard. A 13% yield of 1,3-diiodide **22** was observed by ^{1}H NMR. 74% 1,3-Dimesylate **21** was also observed by ^{1}H NMR.

IV. Experimental Data and Synthesis for Deuterated Cyclopropanes

a) Synthesis of Dideuterated 1,3-Dimesylate **23**

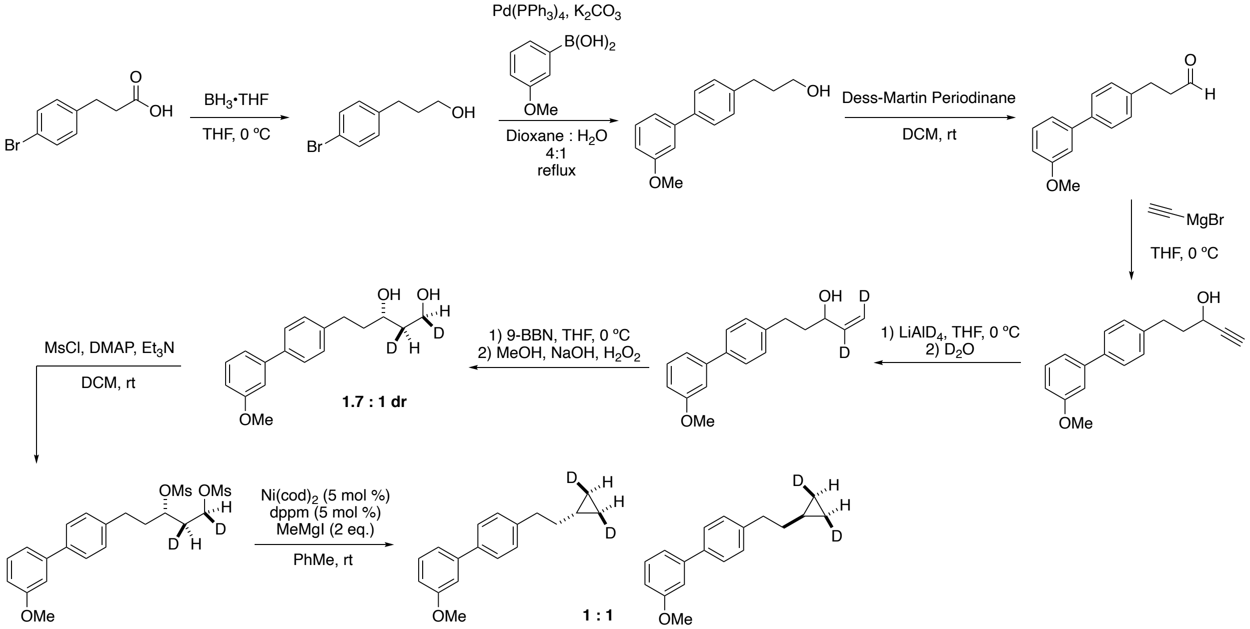

Scheme SI-2: Synthesis of 1,3-Dimesylate **23** and Cyclopropane **25**

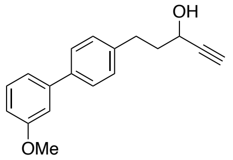

Alkyne SI-7 was prepared according to Method D. The following amounts of reagents were used: **SI-3** (1.5 g, 6.3 mmol, 1.0 equiv), ethynyl magnesium bromide (19 mL, 9.4 mmol, 1.5 equiv, 0.50 M in THF), THF (15 mL, 0.42 M in substrate). The compound was purified by flash column chromatography (0–20% EtOAc/hexanes) to afford the title compound as a colorless oil (1.5 g, 5.6 mmol, 90% yield). **^{1}H NMR** (500 MHz, $CDCl_3$) δ 7.52 (d, *J* =8.0 Hz, 2H), 7.34 (t, *J* =7.7 Hz, 1H), 7.28 (d, *J* =8.0 Hz, 2H), 7.16 (d, *J* =7.7 Hz, 1H), 7.11 (s, 1H), 6.88 (d, *J* =8.2 Hz, 1H), 4.41 (s, 1H), 3.86 (s, 3H), 2.85 (t, *J* =7.7 Hz, 2H), 2.52 (s, 1H), 2.12–2.03 (m, 2H), 1.86 (s, 1H); **^{13}C NMR** (127.5 Hz, $CDCl_3$) δ 160.1, 142.7, 140.5, 139.1, 129.9, 129.0 (2C), 127.4 (2C), 119.7, 112.9, 112.7, 84.8, 73.4, 61.8, 55.4, 39.2, 31.0.

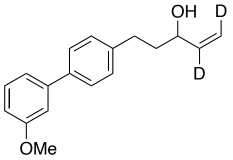

Dideuterated Alkene SI-8 was prepared according to Method I. The following amounts of reagents were used: **SI-7** (0.31 g, 1.2 mmol, 1.0 equiv), lithium aluminum deuteride (0.97 g, 2.3 mmol, 2.0 equiv), D_2O (0.69 mL, 38 mmol, 33 equiv), and THF (10 mL, 0.12 M in substrate). The compound was purified by flash column chromatography (0–20% EtOAc/hexanes) to afford the

title compound as a yellow oil (0.30 g, 1.2 mmol, 96% yield). Deuterium incorporation was measured to be 94% dideuterated and 6% monodeuterated by ^{1}H NMR; **^{1}H NMR** (400 MHz, $CDCl_3$) δ 7.51 (d, *J* =8.2 Hz, 2H), 7.34 (t, *J* =7.9 Hz, 1H), 7.27 (d, *J* = 8.6 Hz, 2H), 7.16 (d, *J* = 7.5 Hz, 1H), 7.11 (s, 1H), 6.88 (dd, *J* = 8.0, 2.5 Hz, 1H), 5.13 (s, 1H), 4.20–4.14 (m, 1H), 3.86 (s, 3H), 2.88–2.69 (m, 2H), 1.94–1.83 (m, 2H).

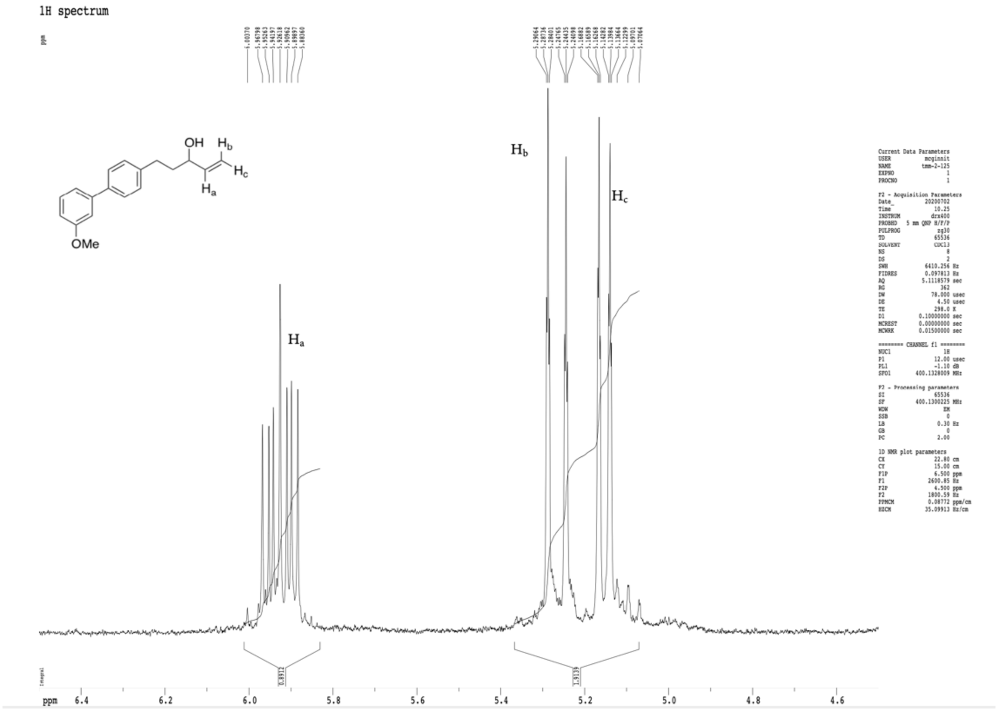

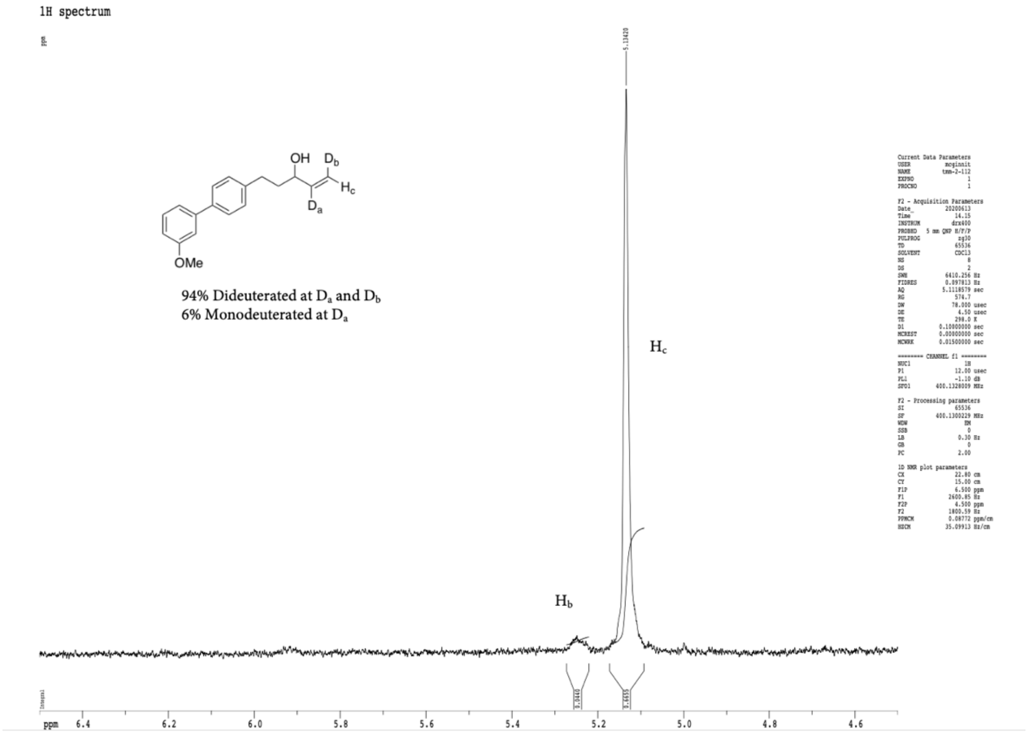

Trans-deuterated alkene is only configuration from the reaction. A small amount (10%) of monodeuterated alkene was carried forward in this synthesis.

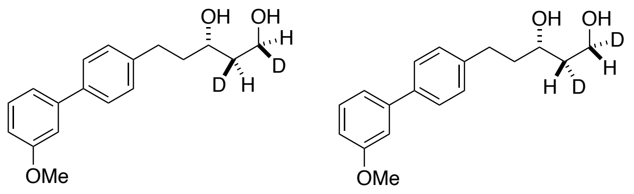

1.7:1 dr

Dideuterated Diol SI-9 was prepared according to Method E. The following amounts of reagents were used: **SI-8** (0.39 g, 1.4 mmol, 1.0 equiv), 9-BBN (7.2 mL, 3.6 mmol, 2.5 equiv, 0.50 M in THF), THF (15 mL, 0.093 M in substrate), MeOH (4.0 mL), NaOH (2.0 mL, 3.0 M), and H_2O_2 (2.0 mL, 30% w/w). The compound was purified by flash column chromatography (0–60% EtOAc/hexanes) to afford the title compound as a white solid (0.14 g, 0.49 mmol, 35% yield). Deuterium incorporation was measured to be 85% dideuterated and 15% monodeuterated by HRMS. Diastereomer ratio was determined to be 1.7:1 by ^{1}H NMR (vide infra); **^{1}H NMR** (500 MHz, C_6D_6) δ 7.52 (d, *J* =8.2 Hz, 2H), 7.24 (s, 1H), 7.21–7.17 (m, 2H), 7.17–7.12 (m, 2H), 6.82–6.76 (m, 1H), 3.63 (br s, 1H), 3.51 (br s, 0.6 H), 3.42 (br s, 0.4 H), 3.37 (s, 3H), 2.77–2.67 (m, 1H), 2.66–2.57 (m, 1H), 2.35 (br s, 1H), 1.74–1.62 (m, 1H), 1.61–1.51 (m, 1H), 1.46–1.39 (m, 0.6 H), 1.32–1.26 (m, 0.4 H), 0.62 (s, 1H).; **^{13}C NMR** (125.7 MHz, C_6D_6) δ 160.7, 143.3, 141.9, 139.4, 130.1, 129.3 (2C), 127.6 (2C), 119.9, 113.2, 113.0, 71.1, 61.3, 54.8, 39.8, 38.4, 31.9; **HRMS** (TOF MS ES+) *m/z*: $[M + Na]^+$ cald for $C_{18}H_{20}O_3D_2Na$, 311.1592; found 311.1520.

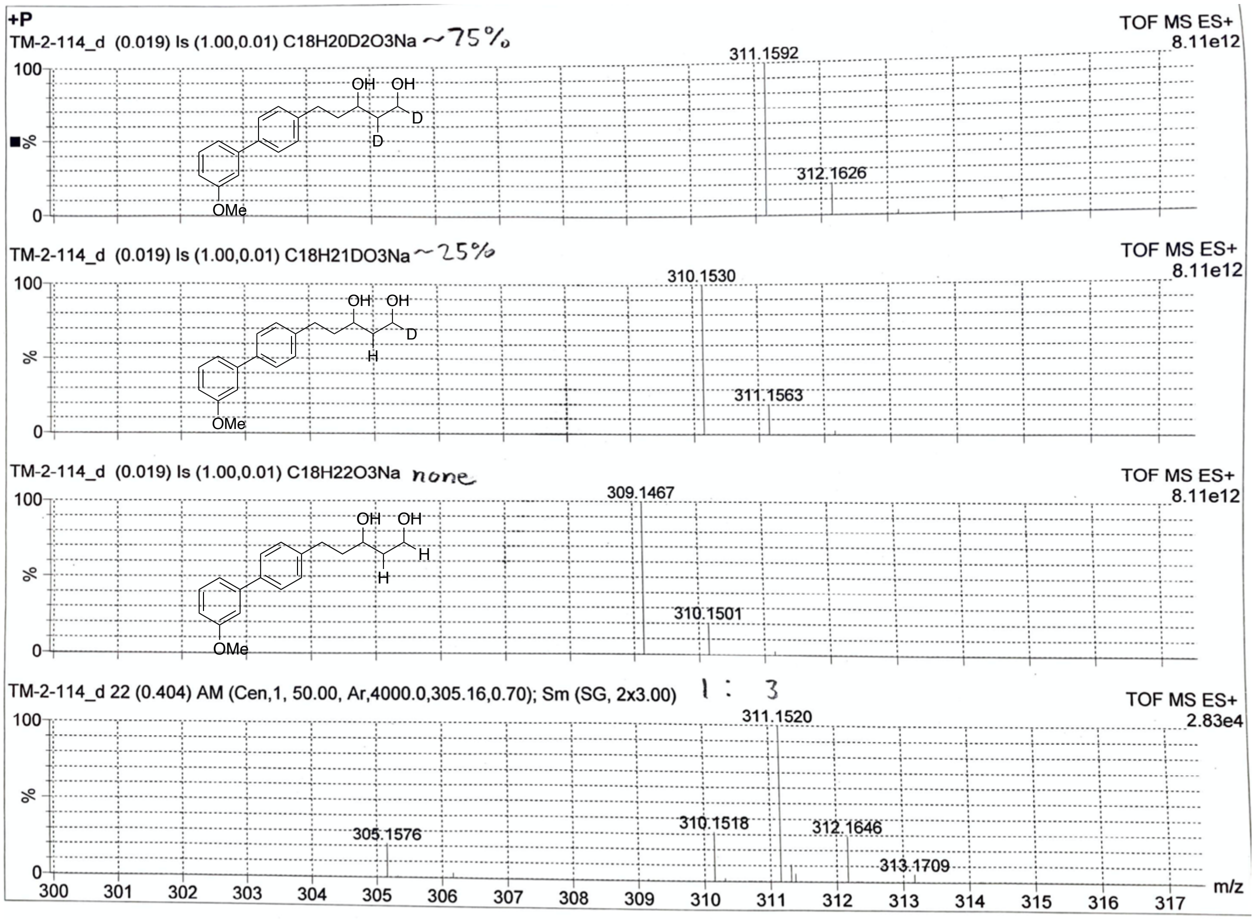

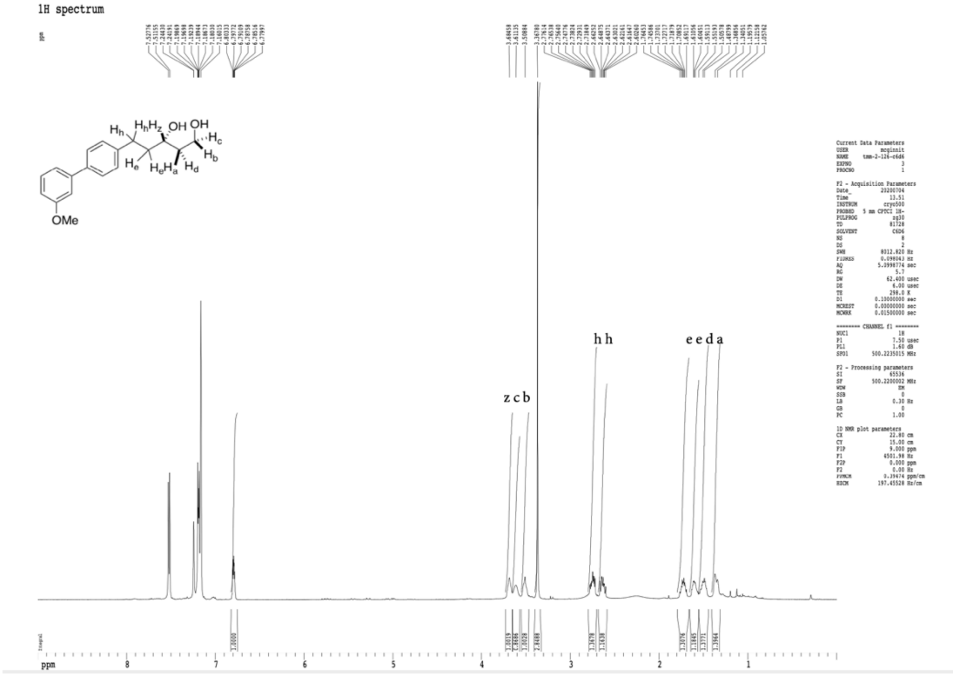

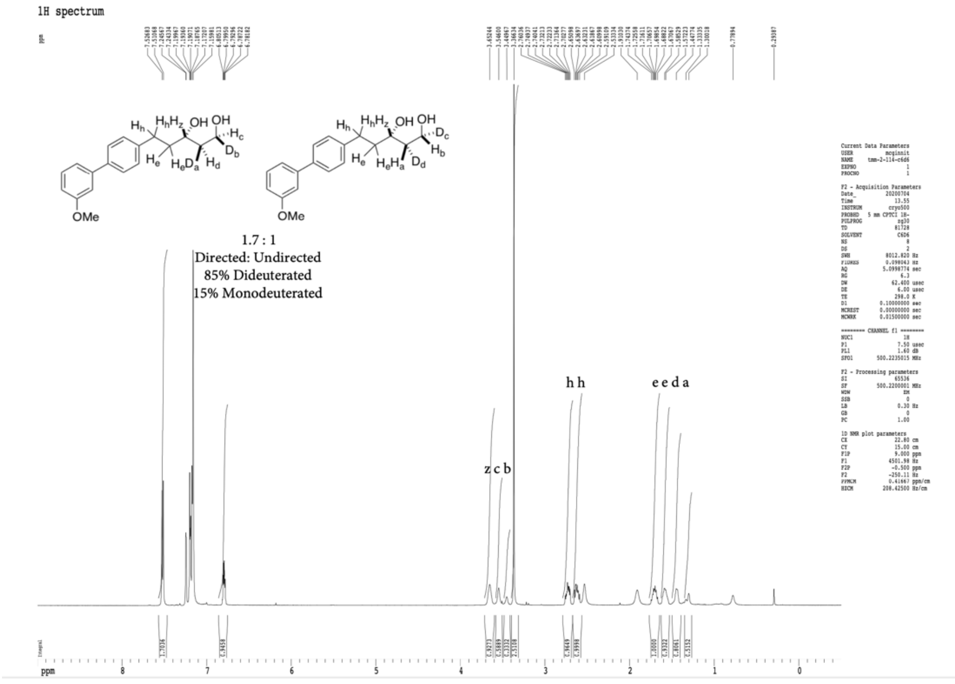

The configuration of H_a/D_a and H_d/D_d was determined by ^1H NMR spectroscopy. A 1.7:1 ratio of was formed. Due to the stereochemical outcome of a hydroboration and oxidation, only syn-deuterated alcohols were formed.

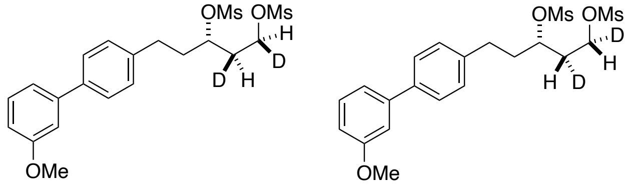

1.7:1 dr

Dideuterated 1,3-Dimesylate *syn*-23 was prepared according to Method F. The following amounts of reagents were used: **SI-9** (0.14 g, 0.49 mmol, 1.0 equiv), Et_3N (0.21 mL, 1.5 mmol, 3.0 equiv), DMAP (12 mg, 0.10 mmol, 0.20 equiv), MsCl (0.090 mL, 1.1 mmol, 2.2 equiv), and DCM (5.0 mL, 0.10 M in substrate). The compound was purified by flash column chromatography (0–50% EtOAc/hexanes) to afford the title compound as a white solid (0.21 g, 0.46 mmol, 94%, 1.7:1 dr). Deuterium incorporation was measured to be 85% dideuterated and 15% monodeuterated by HRMS; **^{1}H NMR** (400 MHz, $CDCl_3$) δ 7.52 (d, *J* =8.0 Hz, 2H), 7.33 (t, *J* =7.9 Hz, 1H), 7.26 (d, *J* =7.9 Hz, 2H), 7.15 (d, *J* =7.9 Hz, 1H), 7.11 (s, 1H), 6.88 (d, *J* =8.2 Hz, 1H), 4.97–4.89 (m, 1H), 4.36–4.30 (m, 1H), 3.84 (s, 3H), 3.03 (s, 3H), 3.01 (s, 3H), 2.81–2.74 (m, 2H), 2.19–2.04 (m, 3H); **^{13}C NMR** (125.8 MHz, $CDCl_3$) δ 160.0, 142.4, 139.7, 139.2, 129.9, 128.8 (2C), 127.4 (2C), 119.6, 112.8, 112.7, 78.3, 65.3 (t, *J* =23.3 Hz), 55.4, 38.7, 37.5, 36.5, 33.7 (t, *J* =19.5 Hz), 30.8; **HRMS** (TOF MS ES+) *m/z*: $[M + Na]^+$ calcd for $C_{20}H_{24}O_7D_2S_2Na$, 467.1112; found 467.1153.

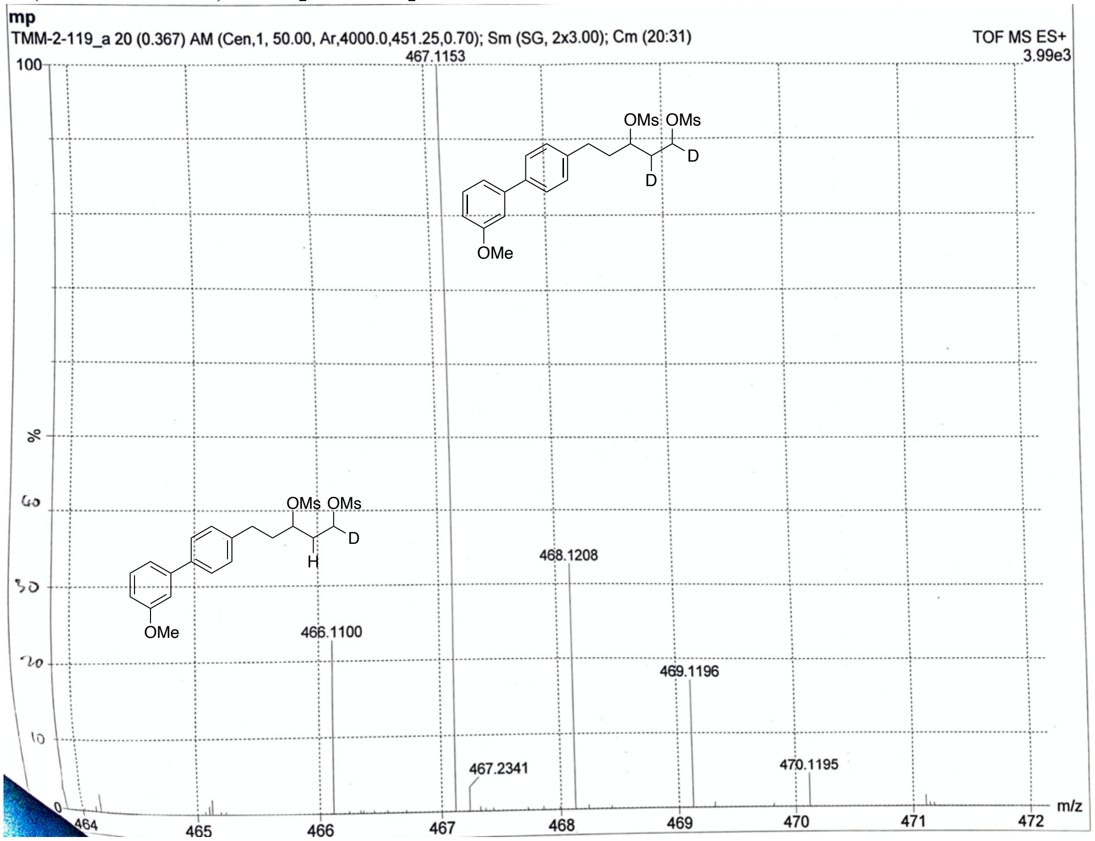

b) Nickel-catalyzed Intramolecular XEC to form Dideuterated Cyclopropane **25**

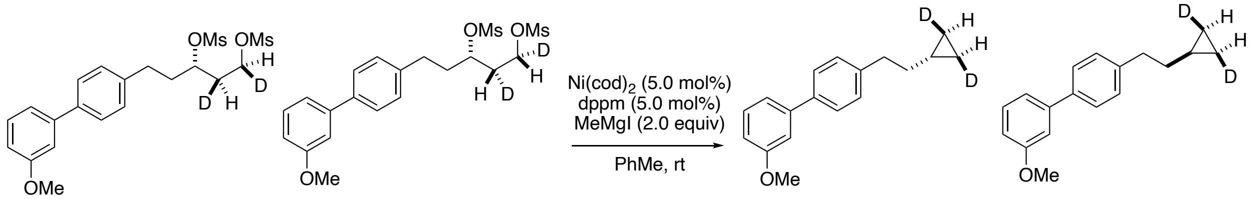

1.7: 1 dr
85% Dideuterated
15% Monodeuterated

1:1 dr
90% Dideuterated
10% Monodeuterated

Syn-Dideuterated Cyclopropane 25 was prepared according to Method G. The following amounts of reagents were used: 1,3-dimesylate ***syn*-23** (44 mg, 0.10 mmol, 1.0 equiv), Ni(cod)$_2$ (1.4 mg, 5.0 μmol, 5.0 mol %), dppm (1.9 mg, 5.0 μmol, 5.0 mol %), methylmagnesium iodide (0.87 mL, 0.20 mmol, 2.0 equiv, 2.3 M in Et$_2$O), and PhMe (1.0 mL, 0.10 M in substrate). A 75% yield was recorded by ^{1}H NMR in comparison to PhTMS as internal standard. The compound was purified by flash column chromatography (100% hexanes) to afford the title compound as a clear, colorless oil (12 mg, 0.049 mmol, 49%). Deuterium incorporation was measured to be 90% dideuterated and 10% monodeuterated by HRMS; **^{1}H NMR** (500 MHz, CDCl$_3$) δ 7.52 (d, J =8.2 Hz, 2H), 7.25 (s, 1H), 7.20 (d, J =6.6 Hz, 2H), 7.13 (d, J =8.0 Hz, 2H), 6.82–6.77 (m, 1H), 3.37 (s, 3H), 2.64 (t, J =7.6 Hz, 2H), 1.43 (q, J =7.0 Hz, 2H), 0.63–0.55 (m, 1H), 0.33 (ad, J =8.0 Hz, 1H), –0.03 (ad, J =4.9 Hz, 1H); **^{13}C NMR** (127.5 MHz, CDCl$_3$) δ 160.4, 143.0, 141.7, 138.9, 129.7, 128.9 (2C), 127.1 (2C), 119.5, 112.9, 112.6, 54.4, 36.6, 35.6, 10.5, 4.1 (t, J =24.2 Hz, 2C); **HRMS** (TOF MS CI+) m/z: [M]$^+$ cald for $C_{18}H_{18}OD_2$, 254.1640; found 254.1652.

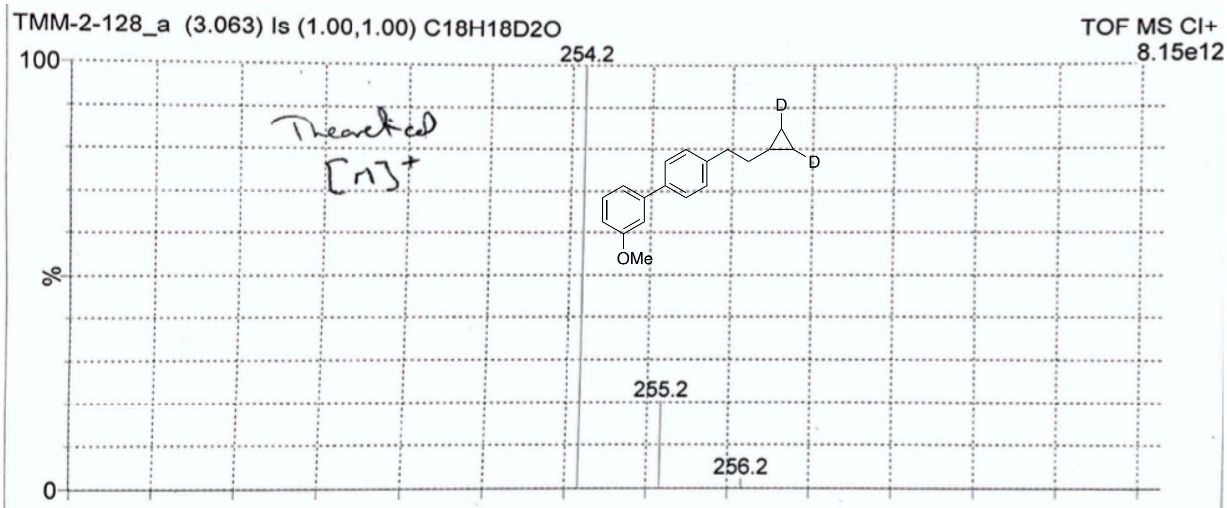

TMM-2-128_a (3.063) Is (1.00,1.00) C18H18D2O
TOF MS CI+
8.15e12
254.2
Theoretical
[M]+
255.2
256.2
OMe

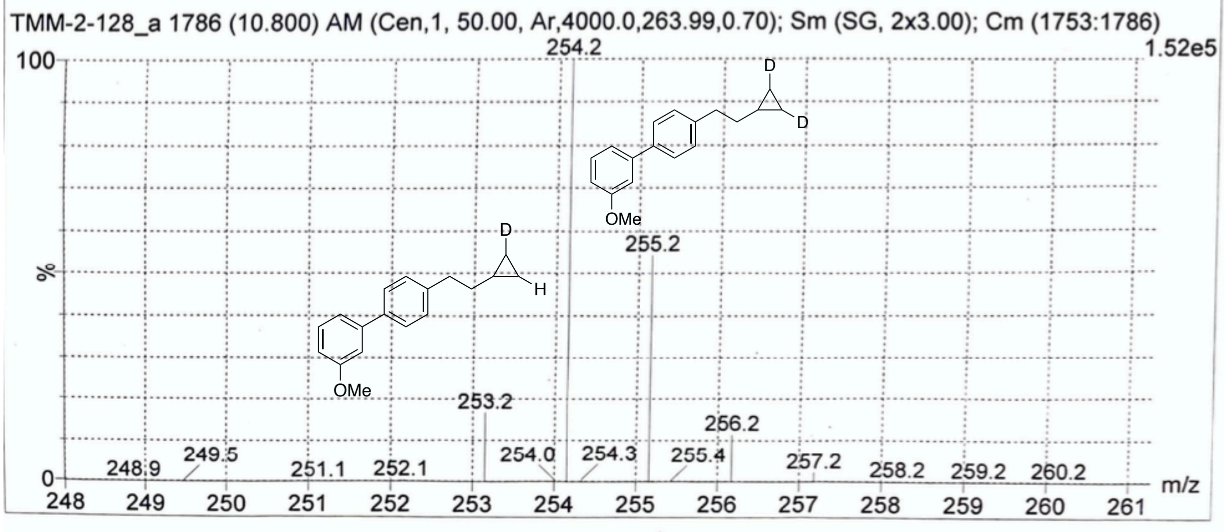

TMM-2-128_a 1786 (10.800) AM (Cen,1, 50.00, Ar,4000.0,263.99,0.70); Sm (SG, 2x3.00); Cm (1753:1786)
1.52e5
254.2
255.2
253.2
256.2
248.9
249.5
251.1
252.1
254.0
254.3
255.4
257.2
258.2
259.2
260.2
m/z
OMe

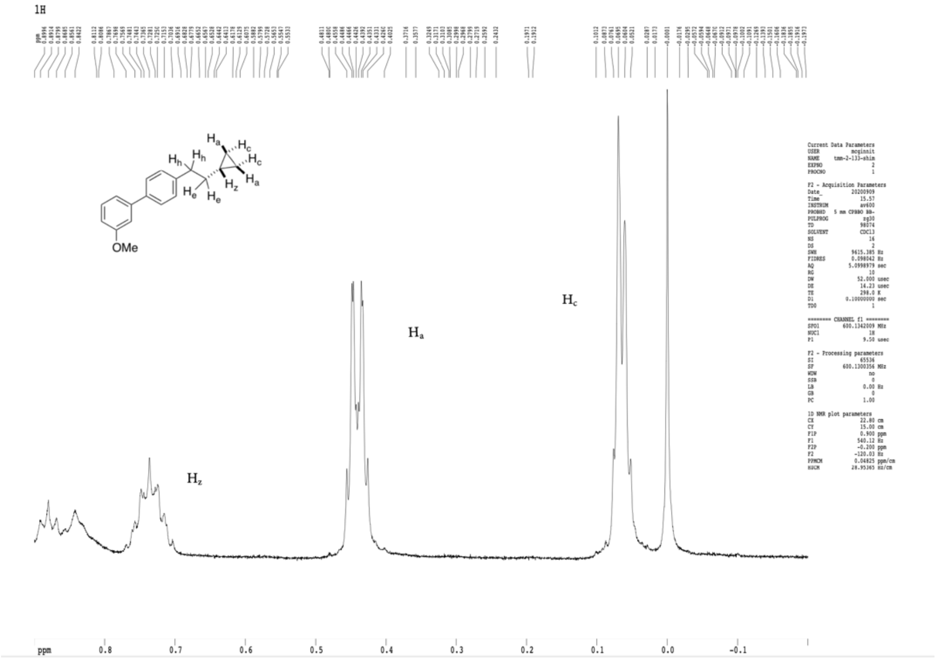

1H
Ha
Hc
Hz
OMe
ppm

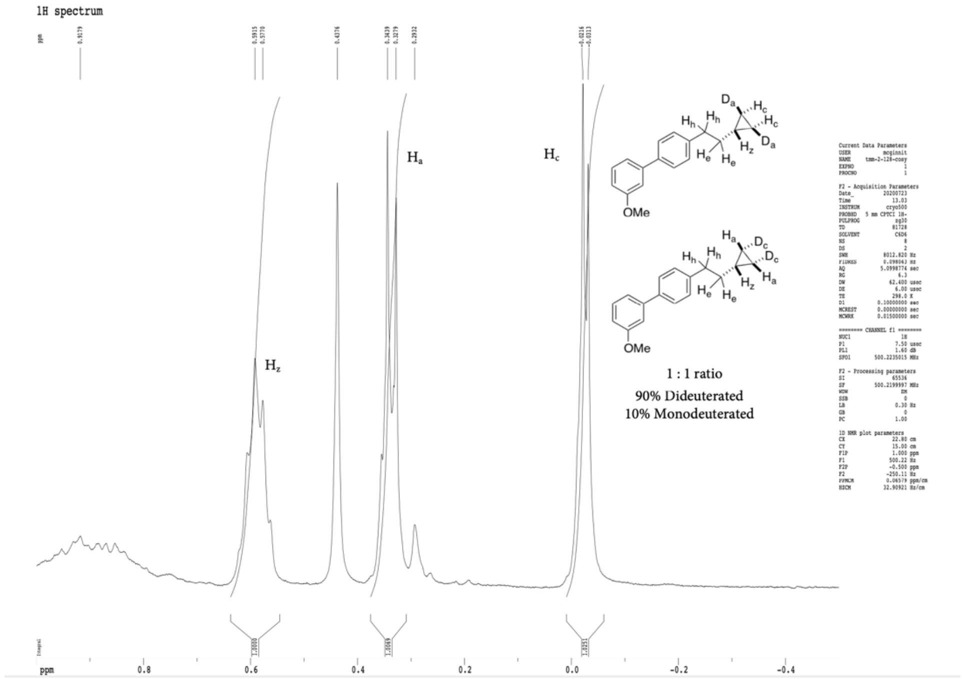

*Only cis-dideuterated cyclopropanes were produced in the XEC reaction. Fully protonated cyclopropane **SI-6** has two triplet of doublets for H_a and H_c. Syn-di-deteuterated cyclopropane **25** has two apparent doublets that signify protons H_a (cis,trans-**25**) and H_c (for cis,cis-**25**) proof of just one other spin state (H_z). A small amount of monodeuterated cyclopropane was noticed by the small apparent triplets next to H_a and H_c.*

c) Synthesis of Trideuterated 1,3-Dimesylate **26**

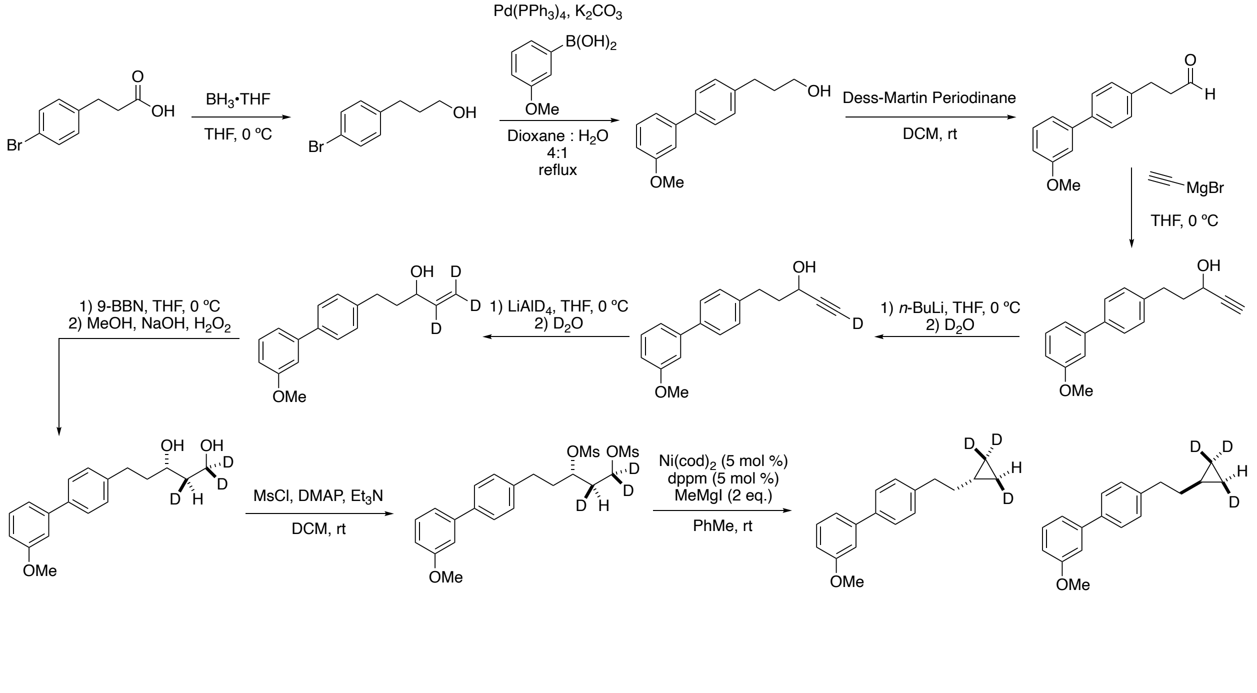

Scheme SI-3: Synthesis of 1,3-Dimesylate **26** and Cyclopropane **28**

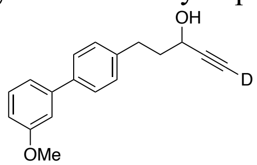

Deuterated Alkyne SI-10 was prepared according to Method H. The following amounts of reagents were used: **SI-7** (1.5 g, 5.6 mmol, 1.0 equiv), *n*-BuLi (5.0 mL, 12 mmol, 2.2 equiv, 2.5 M in hexanes), D_2O (3.4 mL, 170 mmol, 30 equiv), THF (25 mL, 0.22 M in substrate). The compound was purified by flash column chromatography (0–20% EtOAc/hexanes) to afford the title compound as a colorless oil (1.3 g, 4.9 mmol, 88% yield). Deuterium incorporation was measured to be 85% by HRMS. **^{1}H NMR** (500 MHz, $CDCl_3$) δ 7.52 (d, *J* =8.2 Hz, 2H), 7.34 (t, *J* =7.9 Hz, 1H), 7.28 (d, *J* =8.1 Hz, 2H), 7.17 (d, *J* =7.6 Hz, 1H), 7.11 (s, 1H), 6.88 (dd, *J* = 8.2, 2.7 Hz, 1H), 4.40 (t, *J* =6.6, 1H), 3.86 (s, 3H), 2.85 (t, *J* =7.9 Hz, 2H), 2.11–2.05 (m, 2H), 1.86 (s, 1H); **HRMS** (TOF MS ES+) *m/z*: $[M + Na]^+$ cald for $C_{18}H_{17}O_2DNa$, 290.1267; found 290.1250.

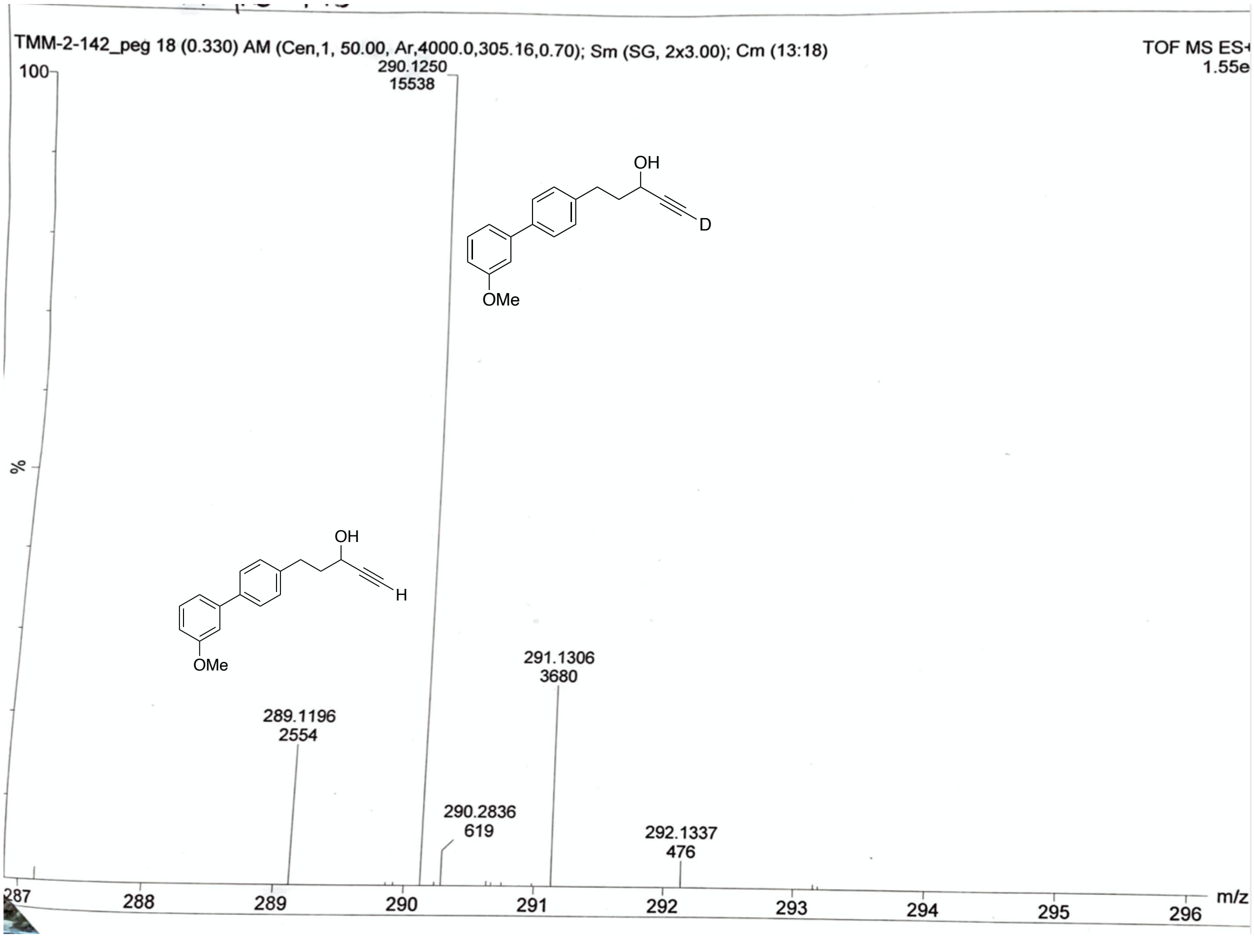

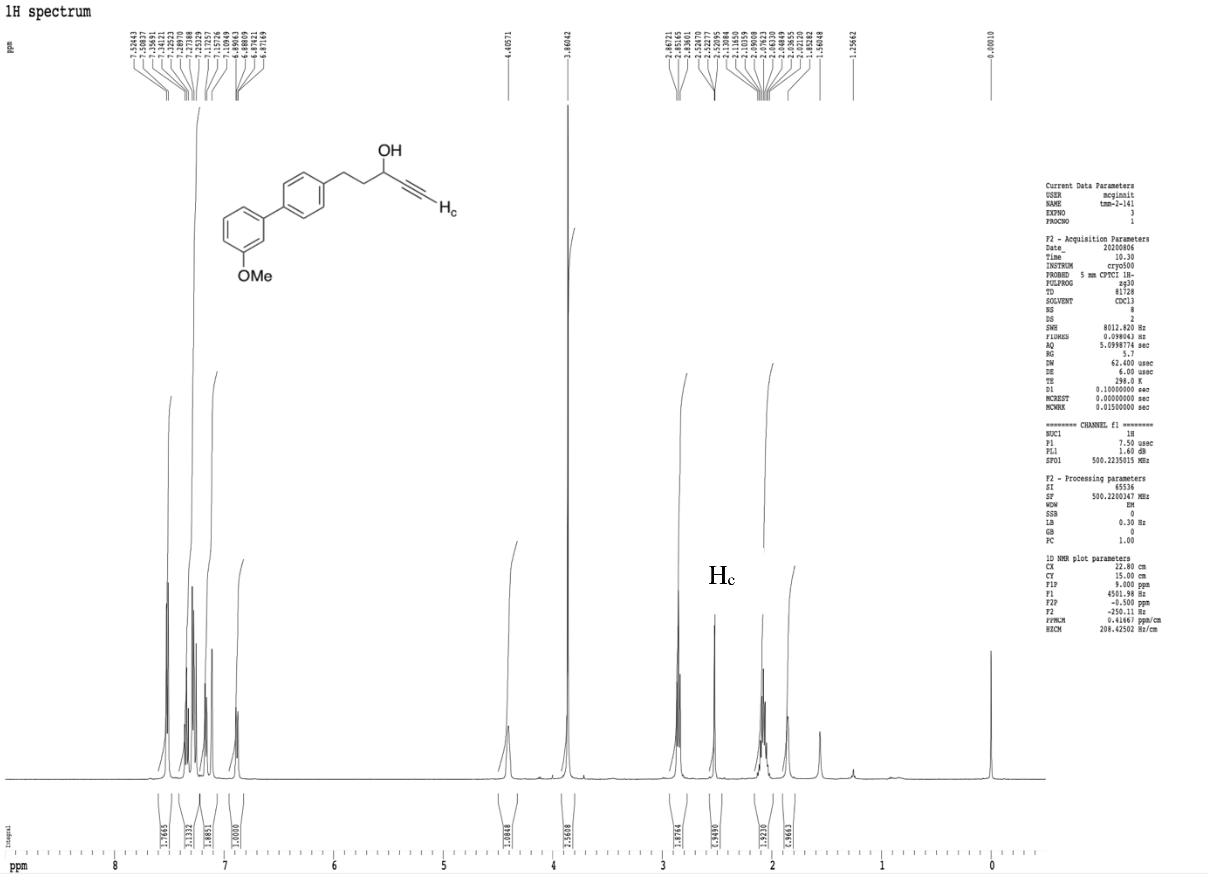
1H spectrum
OH
Hc
OMe
Hc
ppm

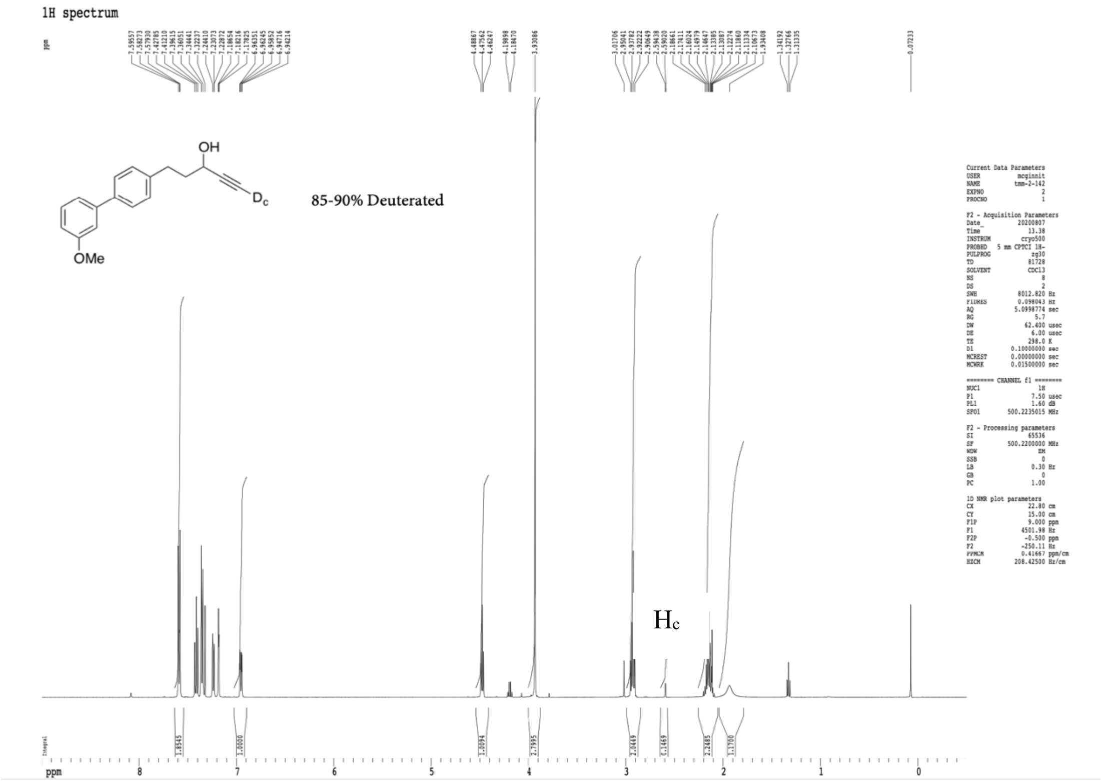
1H spectrum
OH
Dc
OMe
85-90% Deuterated
Hc
ppm

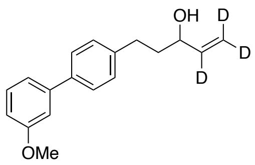

Trideuterated Alkene SI-11 was prepared according to Method I. The following amounts of reagents were used: **SI-10** (1.3 g, 5.0 mmol, 1.0 equiv), lithium aluminum deuteride (0.42 g, 10. mmol, 2.0 equiv), D_2O (3.3 mL, 170 mmol, 33 equiv), and THF (25 mL, 0.20 M in substrate). The compound was purified by flash column chromatography (0–20% EtOAc/hexanes) to afford the title compound as a yellow oil (0.74 g, 2.7 mmol, 54% yield). Deuterium incorporation was measured to be 90% trideuterated and 10% dideuterated by HRMS. **^{1}H NMR** (500 MHz, $CDCl_3$) δ 7.51 (d, *J* =8.2 Hz, 2H), 7.34 (t, *J* =7.9 Hz, 1H), 7.29–7.24 (m, 2H), 7.17 (d, *J* =8.0 Hz, 1H), 7.11 (s, 1H), 6.88 (d, *J* =8.3 Hz, 1H), 4.19–4.13 (m, 1H), 3.85 (s, 3H), 2.88–2.69 (m, 2H), 1.93–1.82 (m, 2H), 1.63–1.57 (m, 1H); **HRMS** (TOF MS ES+) *m/z*: $[M + Na]^+$ cald for $C_{18}H_{17}O_2D_3Na$, 294.1549; found 294.1535.

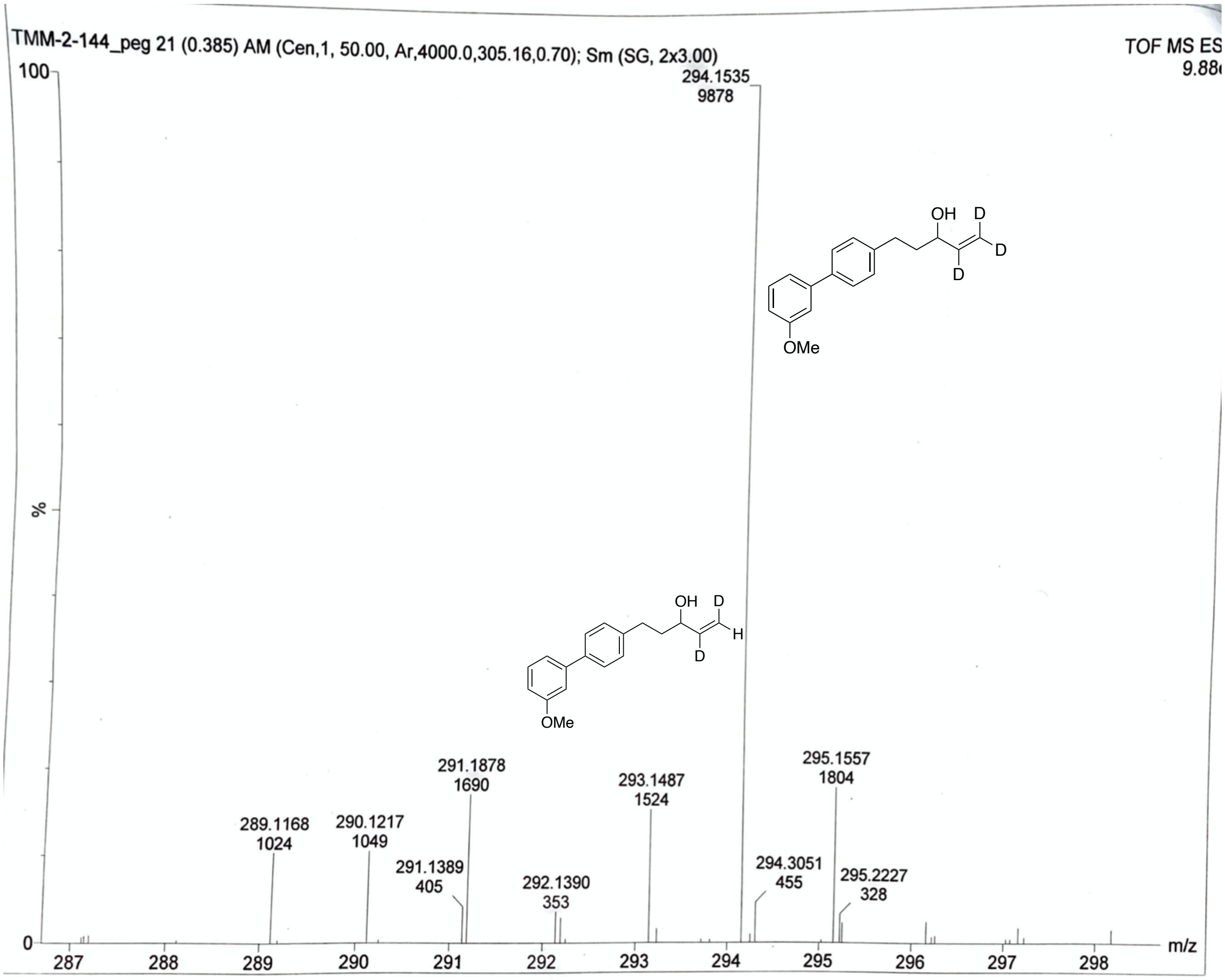

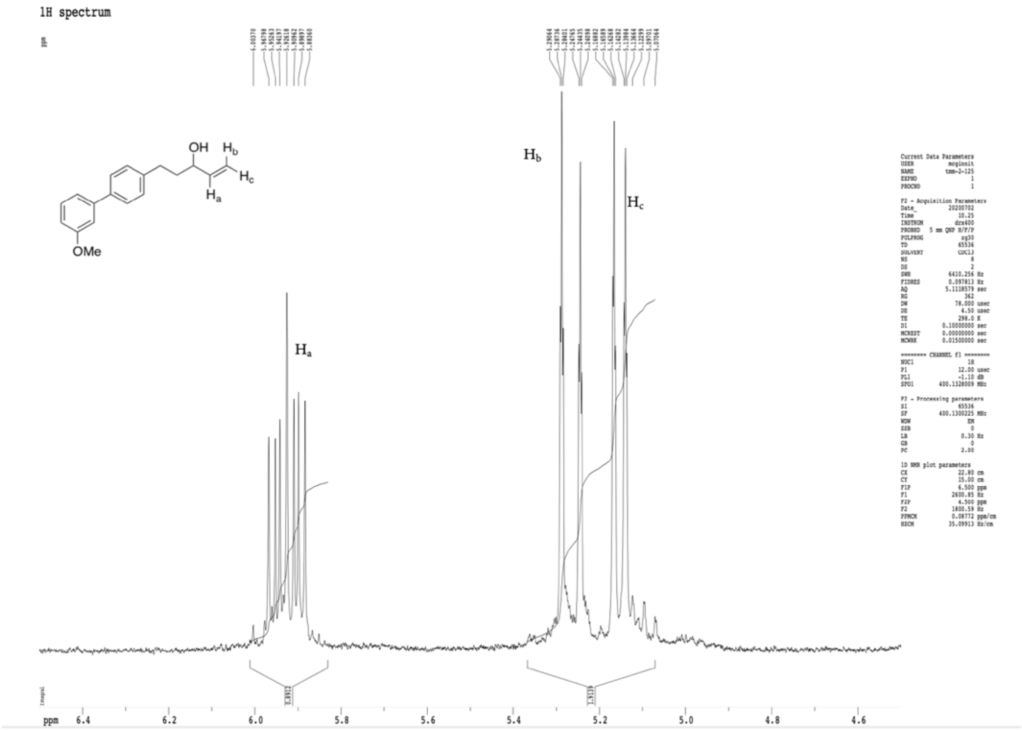
1H spectrum
OH
OMe
Ha
Hb
Hc
ppm 6.4 6.2 6.0 5.8 5.6 5.4 5.2 5.0 4.8 4.6

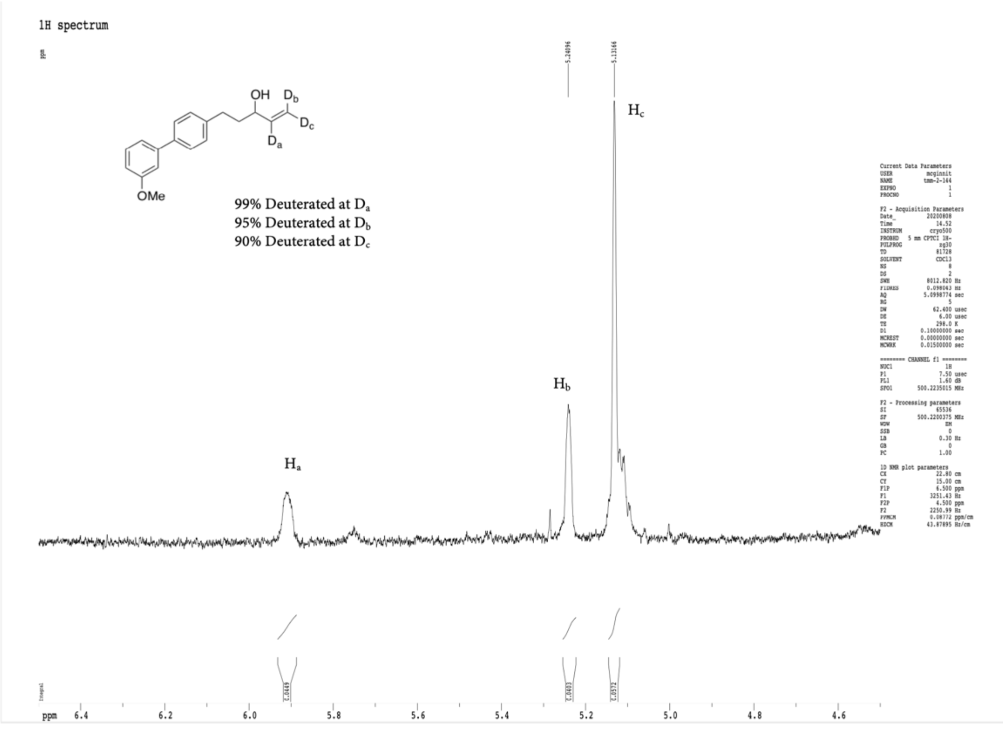
1H spectrum
OH
OMe
Da
Db
Dc
99% Deuterated at Da
95% Deuterated at Db
90% Deuterated at Dc
Ha
Hb
Hc
ppm 6.4 6.2 6.0 5.8 5.6 5.4 5.2 5.0 4.8 4.6

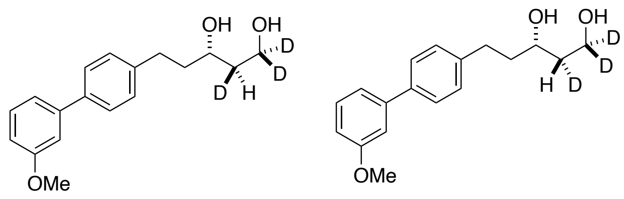

1.8:1 dr

Trideuterated Diol SI-12 was prepared according to Method E. The following amounts of reagents were used: **SI-11** (0.32 g, 1.2 mmol, 1.0 equiv), 9-BBN (6.0 mL, 3.0 mmol, 2.5 equiv, 0.5 M in THF), THF (5.0 mL, 0.24 M in substrate), MeOH (5.0 mL), NaOH (2.5 mL, 3.0 M), and H_2O_2 (2.5 mL, 30% w/w). The compound was purified by flash column chromatography (0–60% EtOAc/hexanes) to afford the title compound as a white solid (0.35 g, 1.2 mmol, 99% yield). Deuterium incorporation was measured to be 90% trideuterated and 10% dideuterated by HRMS. Diastereomer ratio was determined to be 1.8:1 by ^{1}H NMR (vide infra). **^{1}H NMR** (500 MHz, C_6D_6) δ 7.52 (d, J =8.2 Hz, 2H), 7.25 (s, 1H), 7.22–7.13 (m, 4H), 6.82–6.77 (m, 1H), 3.63–3.56 (m, 1H), 3.37 (s, 3H), 2.75–2.67 (m, 1H), 2.65–2.56 (m, 1H), 2.08 (br s, 1H), 1.72–1.62 (m, 1H), 1.59–1.51 (m, 1H), 1.42–1.36 (m, 0.6 H), 1.29 (br s, 1H), 1.26–1.23 (m, 0.4 H); **^{13}C NMR** (125.7 MHz, C_6D_6) δ 160.0, 142.6, 141.3, 138.8, 129.8, 128.9 (2C), 127.3 (2C), 119.6, 112.8, 112.5, 71.5, 61.1 (t, J =22.0 Hz), 55.3, 39.4, 37.9 (t, J =18.5 Hz), 31.6; **HRMS** (TOF MS ES+) m/z: $[M + Na]^+$ cald for $C_{18}H_{19}O_3D_3Na$, 312.1655; found, 312.1658.

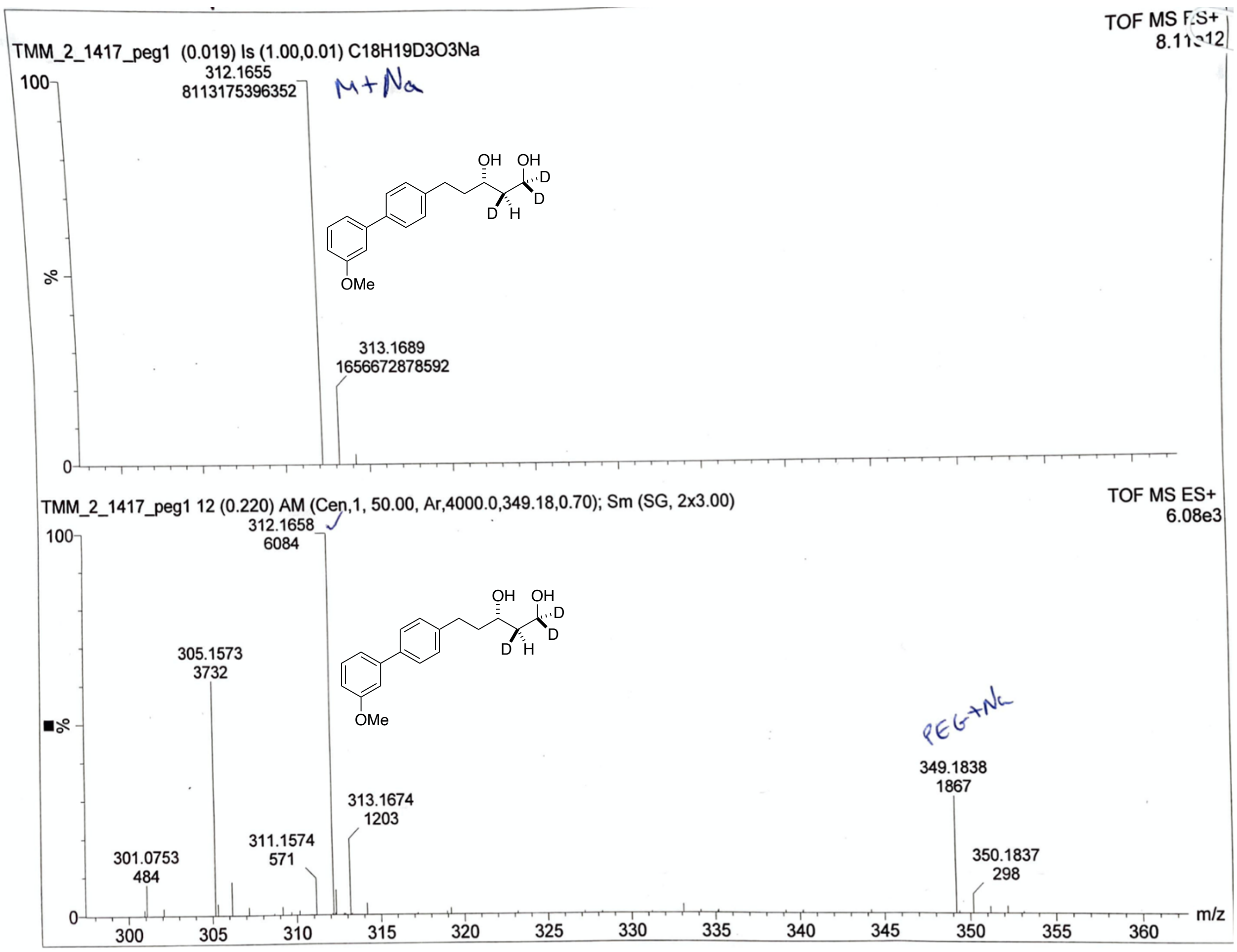

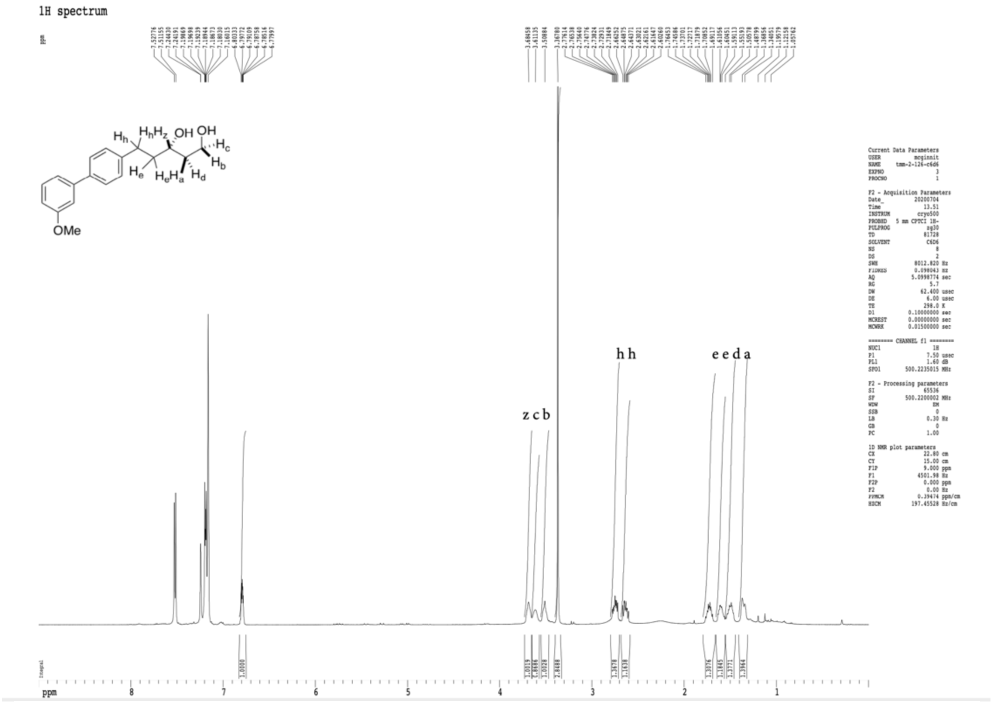

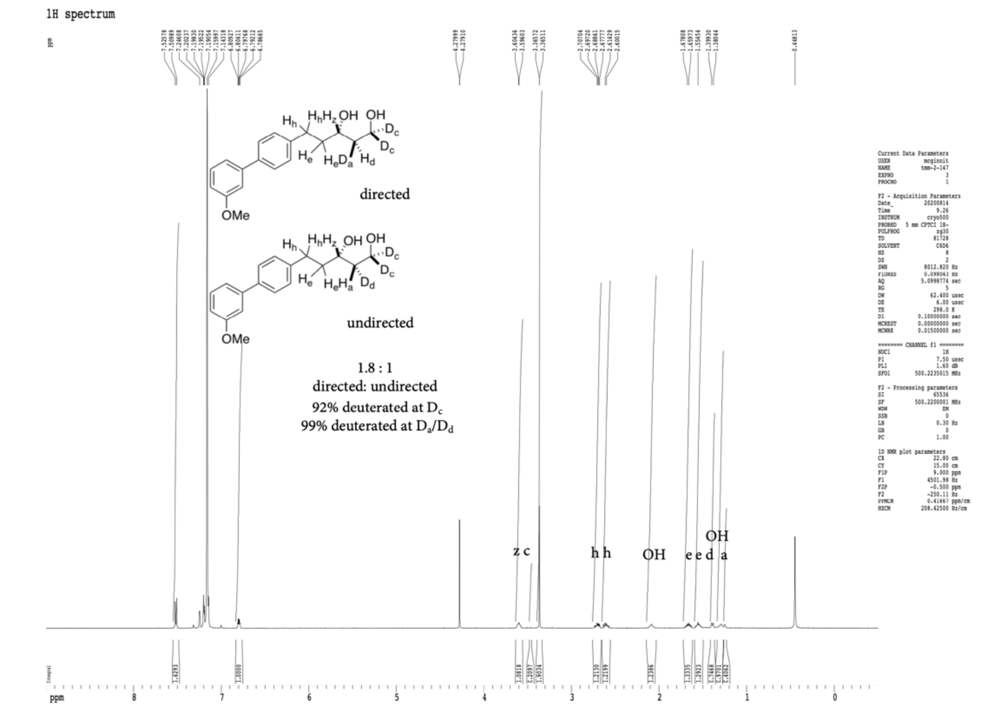

The configuration of H_a/D_a and H_d/D_d was determined by ^1H NMR spectroscopy. A 1.8:1 ratio of epimers at the secondary position was formed.

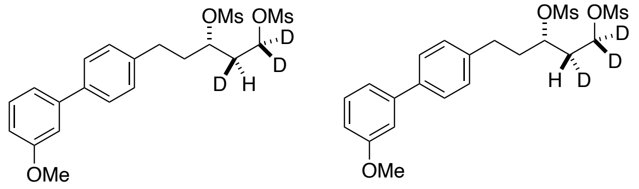

1.8:1 dr

Trideuterated 1,3-Dimesylate 26 was prepared according to Method F. The following amounts of reagents were used: **SI-12** (0.39 g, 1.4 mmol, 1.0 equiv), Et_3N (0.57 mL, 4.1 mmol, 3.0 equiv), DMAP (33 mg, 0.27 mmol, 0.20 equiv), MsCl (0.23 mL, 3.0 mmol, 2.2 equiv), and DCM (7.0 mL, 0.20 M in substrate). The compound was purified by flash column chromatography (0–50% EtOAc/hexanes) to afford the title compound as a white solid (0.14 g, 0.31 mmol, 23%, 1.8:1 dr). Deuterium incorporation was measured to be 90% trideuterated and 10% dideuterated by HRMS; **^{1}H NMR** (600 MHz, C_6D_6) δ 7.50 (d, *J* =8.1 Hz, 2H), 7.23 (s, 1H), 7.20–7.17 (m, 2H), 7.09 (d, *J* =8.0 Hz, 2H), 6.79 (d, *J* =7.3 Hz, 1H), 4.71–4.66 (m, 1H), 3.37 (s, 3H), 2.49 (t, *J* =8.1 Hz, 2H), 2.28 (s, 3H), 2.25 (s, 3H), 1.79–1.71 (m, 1H), 1.68–1.60 (m, 1H), 1.57–1.54 (m, 0.4 H), 1.52–1.49 (m, 0.84 H); **^{13}C NMR** (150.9 MHz, C_6D_6) δ 160.4, 142.6, 139.8, 139.4, 129.8, 128.8 (2C), 127.4 (2C), 119.5, 112.9, 112.7, 77.6, 77.1 (t, *J* =31.7 Hz), 54.5, 37.6, 36.4, 36.3, 33.3 (t, *J* =19.3 Hz), 30.5; **HRMS** (TOF MS ES+) *m/z*: $[M + Na]^+$ cald for $C_{20}H_{23}O_7D_3S_2Na$, 468.1206; found 468.1211.

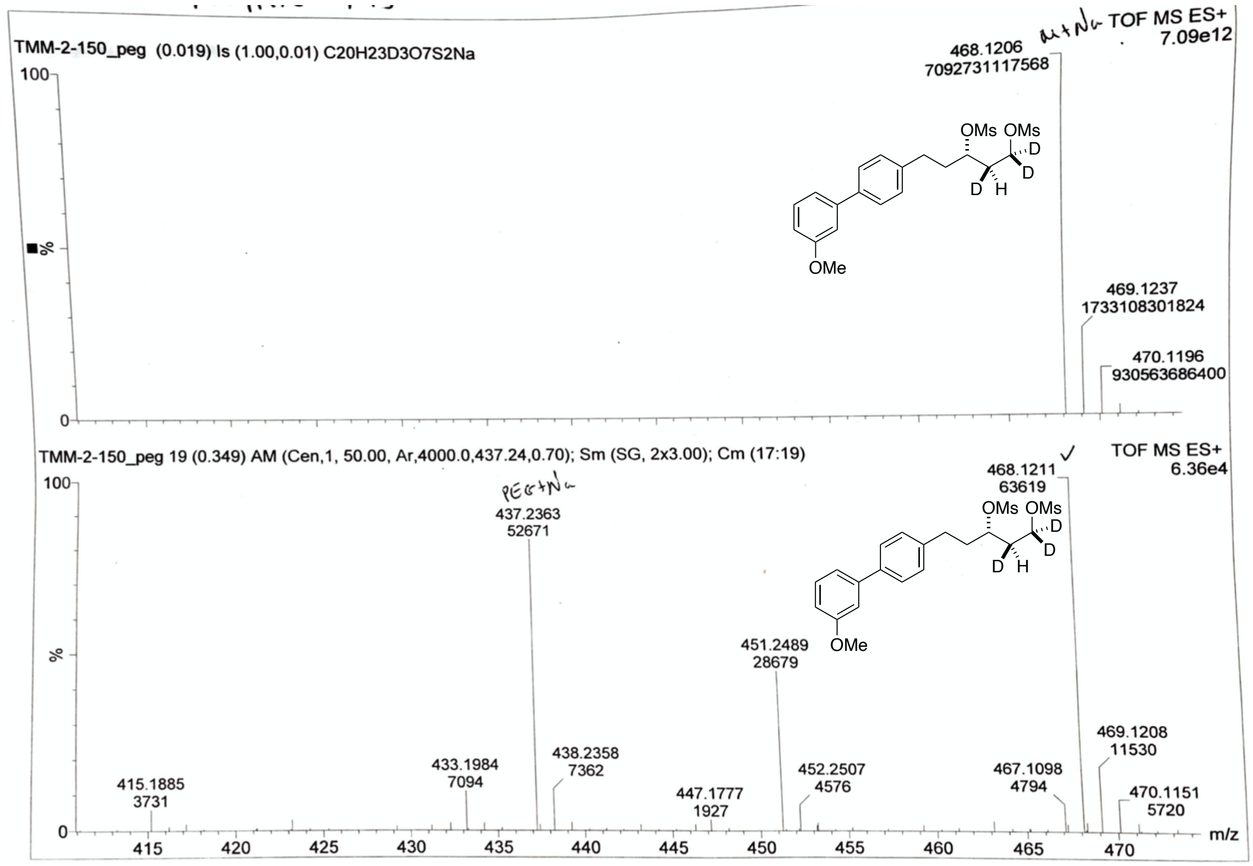

d) Nickel-catalyzed Intramolecular XEC to form Trideuterated Cyclopropane **28**

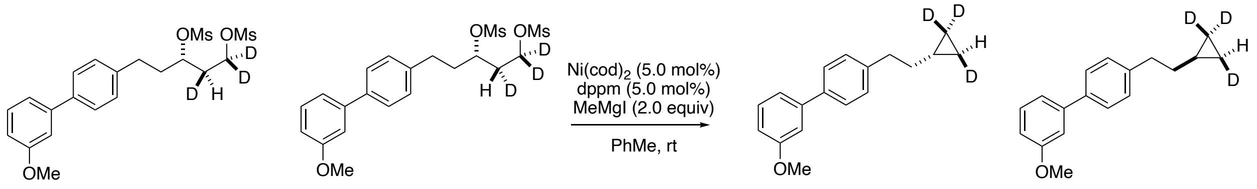

1.8:1 dr **1:1 dr**

Trideuterated Cyclopropane 28 was prepared according to Method G. The following amounts of reagents were used: 1,3-dimesylate **26** (89 mg, 0.20 mmol, 1.0 equiv), $Ni(cod)_2$ (2.8 mg, 0.010 mmol, 5.0 mol %), dppm (3.8 mg, 0.010 mmol, 5.0 mol %), methylmagnesium iodide (0.17 mL, 0.40 mmol, 2.0 equiv, 2.3 M in Et_2O), and PhMe (2.0 mL, 0.10 M in substrate). A 73% yield was recorded by ^{1}H NMR in comparison to PhTMS as internal standard. The compound was purified by flash column chromatography (100% hexanes) to afford the title compound as a clear, colorless oil (33 mg, 0.13 mmol, 65%). Deuterium incorporation was measured to be 95% trideuterated and 5% dideuterated by HRMS. Diastereomer ratio was determined to be 1.8:1 by ^{1}H NMR.; **^{1}H NMR** (600 MHz, $CDCl_3$) δ 7.49 (d, *J* =8.1 Hz, 2H), 7.33 (t, *J* =7.9 Hz, 1H), 7.25 (d, *J* =8.1 Hz, 2H), 7.17 (d, *J* =8.1 Hz, 1H), 7.11 (s, 1H), 6.87 (d, *J* =8.2 Hz, 1H), 3.85 (s, 3H), 2.74 (t, *J* =7.8 Hz, 2H), 1.54 (q, *J* =7.5 Hz, 2H), 0.71 (q, *J* =7.0 Hz, 1H), 0.41 (ad, *J* =8.1 Hz, 0.5 H), 0.03 (ad, *J* =5.0 Hz, 0.5 H); **^{13}C NMR** (150.9 MHz, $CDCl_3$) δ 160.0, 142.9, 142.2, 138.6, 129.8, 129.0 (2C), 127.1 (2C), 119.7, 112.9, 112.5, 55.4, 36.8, 35.8, 10.6, 4.1 (t, *J* =23.8 Hz, 2C); **HRMS** (TOF MS CI+) *m/z*: $[M]^+$ calcd for $C_{18}H_{17}OD_3$, 255.1702; found 255.1691.

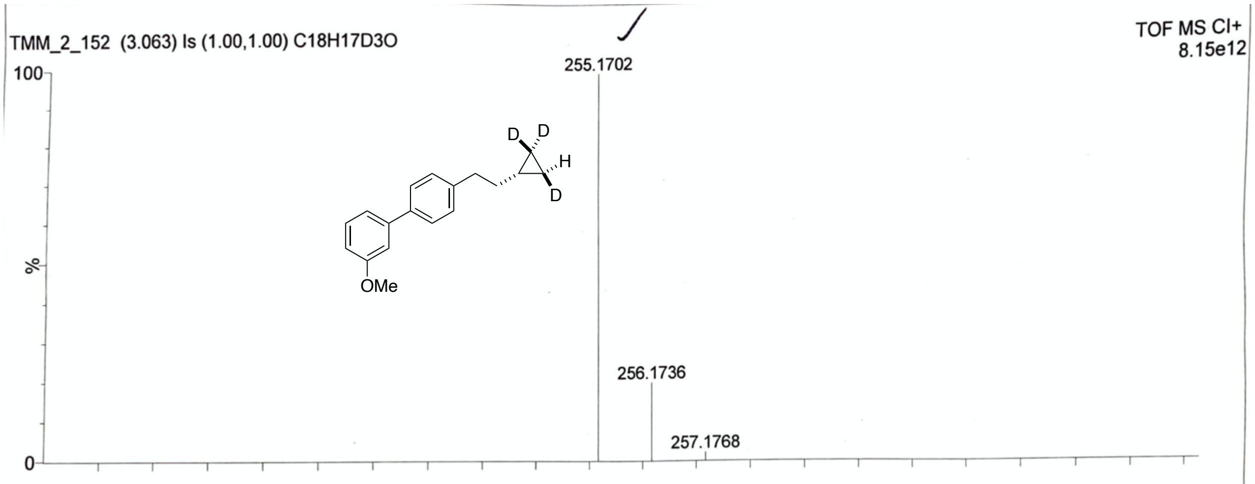
TMM_2_152 (3.063) Is (1.00,1.00) C18H17D3O
TOF MS CI+
8.15e12
255.1702
256.1736
257.1768
OMe
D
H
%
100
0

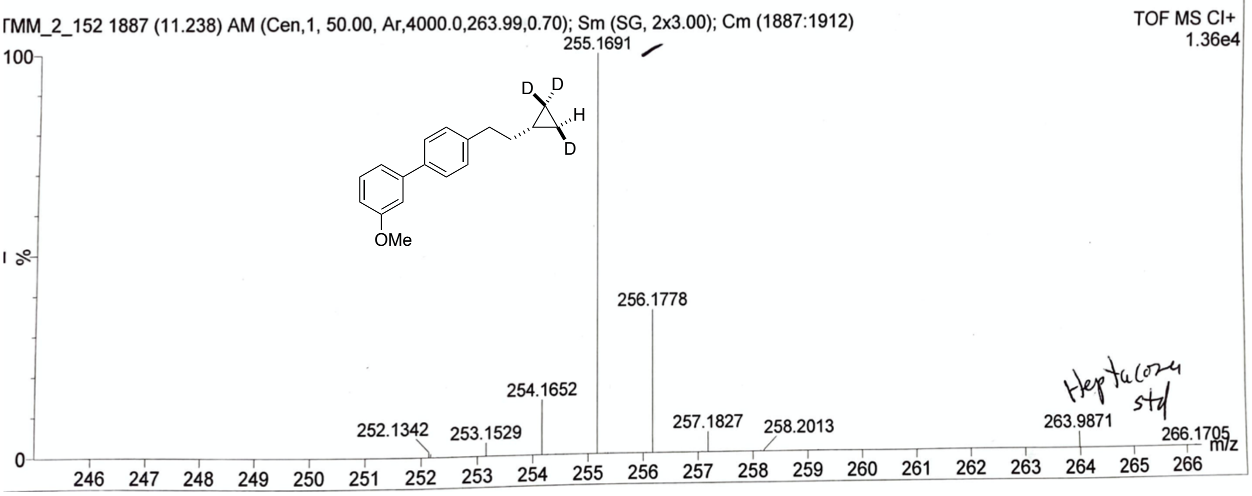
TMM_2_152 1887 (11.238) AM (Cen,1, 50.00, Ar,4000.0,263.99,0.70); Sm (SG, 2x3.00); Cm (1887:1912)
TOF MS CI+
1.36e4
255.1691
256.1778
254.1652
252.1342
253.1529
257.1827
258.2013
263.9871
266.1705
m/z
OMe
D
H
%
100
0

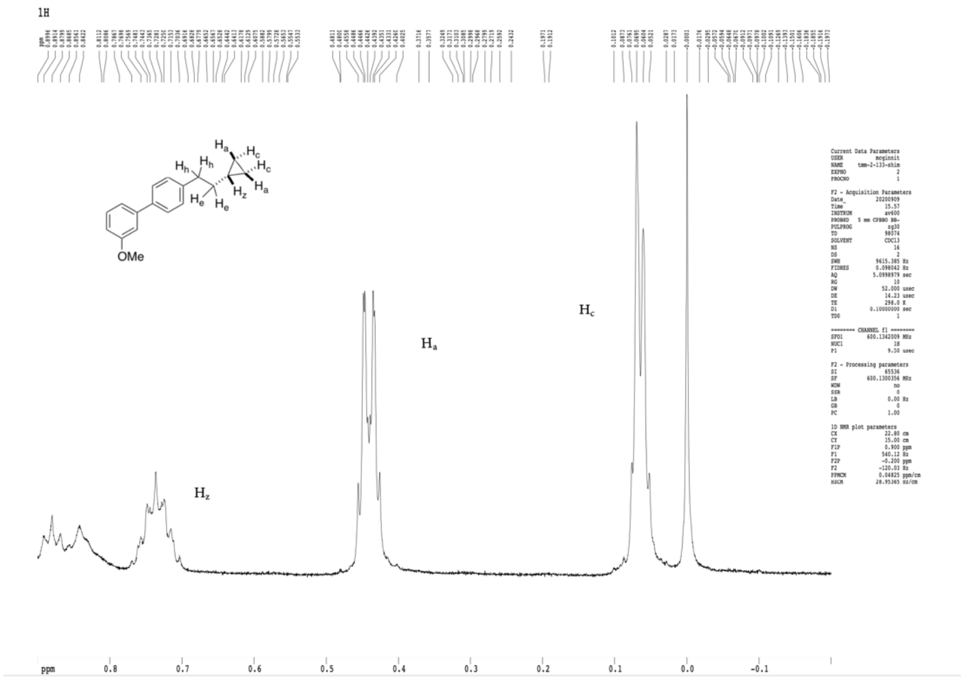
1H
OMe
Ha
Hc
Hz
ppm

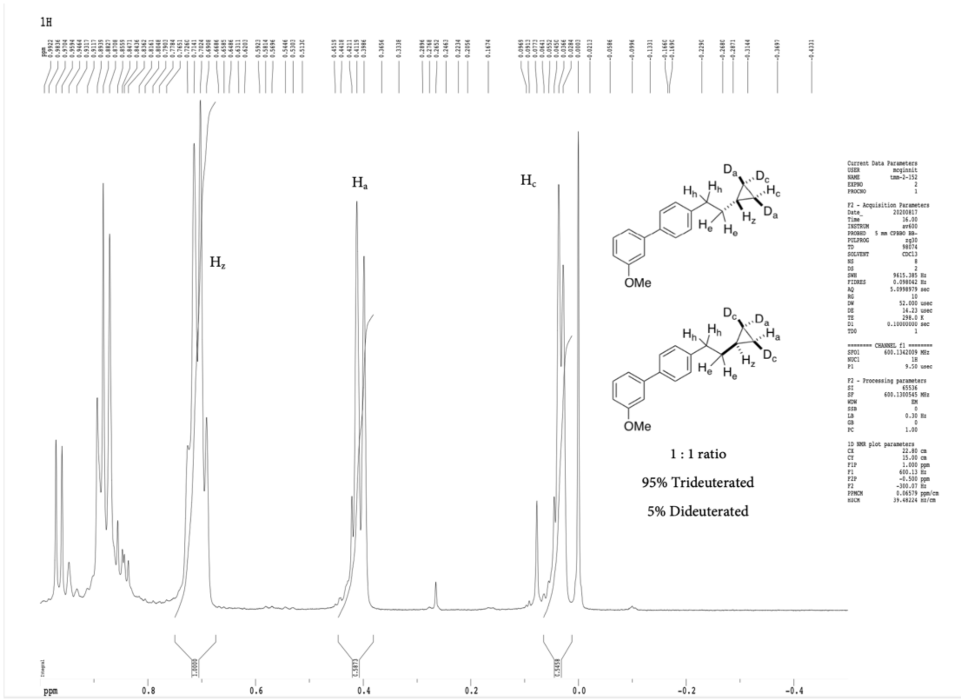

*Trideuterated cyclopropane **28** was formed in a 1:1 ratio of diasteriomers signified by the 1:1 ratio of H_a to H_c. A small amount of dideuterated cyclopropane was noticed by the small apparent doublet next to H_a and H_c.*

V. Experimental Data and Synthesis for the 5-Exo-Trig and 6-Exo Trig Clock Substrates

a) Synthesis of 5-Exo-Trig Clock Diol **29a**

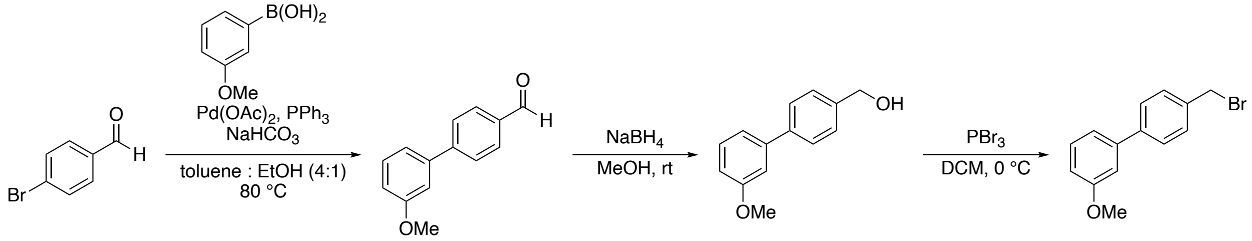

Scheme SI-4. Synthesis of Benzylic Bromide **SI-15**.

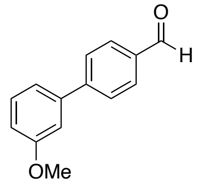

Aldehyde SI-13 was prepared following Method B2. The following amounts of reagents were used: 4-bromobenzaldehyde (1.9 g, 10. mmol, 1.0 equiv), (3-methoxyphenyl)boronic acid (1.5 g, 10. mmol, 1.0 equiv), $Pd(OAc)_2$ (110 mg, 0.50 mmol, 5.0 mol %), PPh_3 (530 mg, 2.0 mmol, 0.20 equiv), $NaHCO_3$ (1.7 g, 20. mmol, 2.0 equiv, 0.20 M in H_2O), PhMe (50. mL, 0.20 M in substrate), and EtOH (13 mL). The residue was purified by flash column chromatography (0–10% EtOAc/hexanes) to afford the title compound as a clear, colorless oil (1.7 g, 8.2 mmol, 82%). **TLC** $\mathbf{R_f}$ = 0.4 (10% EtOAc/hexanes); **^{1}H NMR** (400 MHz, $CDCl_3$) δ 10.06 (s, 1H), 7.94 (d, J = 8.2 Hz, 2H), 7.74 (d, J = 8.2 Hz, 2H), 7.40 (t, J = 8.0 Hz, 1H), 7.22 (d, J = 7.7 Hz, 1H), 7.16 (at, J = 2.0 Hz, 1H), 6.98 (dd, J = 8.2, 2.5 Hz, 1H), 3.88 (s, 3H). Analytical data is consistent with literature values.[10]

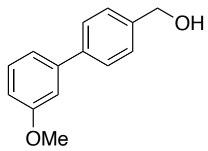

Alcohol SI-14 was prepared following a procedure reported by Bao.[11] **SI-13** (1.6 g, 7.4 mmol, 1.0 equiv), $NaBH_4$ (0.56 g, 15 mmol, 2.0 equiv), and MeOH (37 mL, 0.20 M in substrate) were added to a round-bottom flask equipped with a stir bar. The reaction mixture was allowed to stir open to air for 30 mins. The reaction mixture was then concentrated in vacuo, then diluted with DCM and extracted with H_2O (3 x 20 mL). The combined organic layers were washed with brine, dried over Na_2SO_4, and concentrated in vacuo. The residue was used directly in the next step without further purification. **TLC** $\mathbf{R_f}$ = 0.2 (20% EtOAc/hexanes); **^{1}H NMR** (400 MHz, $CDCl_3$) δ 7.58 (d, J =8.2 Hz, 2H), 7.43 (d, J = 8.2 Hz, 2H), 7.35 (t, J = 7.9 Hz, 1H), 7.18 (ad, J = 7.7 Hz, 1H), 7.12 (at, J = 8.2 Hz, 1H), 6.91 (ddd, J = 8.2, 2.5, 0.8 Hz, 1H), 4.74 (d, J = 5.9 Hz, 2H), 3.87 (s, 3H), 1.68 (t, J = 6.0 Hz, 1H). Analytical data is consistent with literature values.[12]

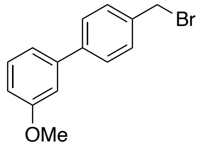

Benzylic Bromide SI-15 was prepared following a procedure reported by Bao.[11] To a flame-dried round-bottom flask, **SI-14** (1.6 g, 7.4 mmol, 1.0 equiv) and DCM (74 mL, 0.10 M in substrate) were added and cooled to 0 °C. PBr_3 (0.77 mL, 8.1 mmol, 1.1 equiv) was added neat dropwise via syringe, and the reaction mixture was allowed to stir at 0 °C for 30 mins. The reaction mixture was the diluted with cold DI H_2O and extracted with DCM (3 x 50 mL). The combined organic layers were washed with brine, dried over Na_2SO_4, and concentrated in vacuo. The residue was purified by flash column chromatography (0–10% EtOAc/hexanes) to afford the title compound as a white solid (0.91 g, 3.3 mmol, 44%). **m.p.** 45–48°C; **TLC R_f** = 0.8 (20% EtOAc/hexanes); **^{1}H NMR** (400 MHz, $CDCl_3$) δ 7.56 (d, J = 8.4 Hz, 2H), 7.46 (d, J = 8.4 Hz, 2H), 7.36 (t, J = 7.9 Hz, 1H), 7.16 (dq, J = 7.4, 1.0 Hz, 1H), 7.12 (at, J = 2.1 Hz, 1H), 6.92 (ddd, J = 8.2, 2.5, 0.8 Hz, 1H), 4.55 (s, 2H), 3.86 (s, 3H); **^{13}C NMR** (100 MHz, $CDCl_3$) δ 160.1, 142.1, 141.4, 137.1, 130.0, 129.6 (2C), 127.7 (2C), 119.8, 113.1, 113.0, 55.5, 33.5; **HRMS** (TOF MS CI+) *m/z*: $[M]^+$ calculated for $C_{14}H_{13}BrO$ 276.0150, found 276.0158.

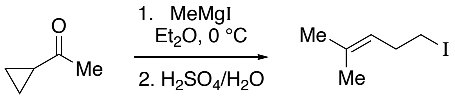

Iodide SI-16 was prepared following a procedure reported by Gibson.[13] To a flame-dried round-bottom flask with stir bar, anhydrous Et_2O (3.0 mL, 1.7 M in substrate) and methylmagnesium iodide (3.9 mL, 6.0 mmol, 1.2 equiv, 1.5 M in Et_2O) were added at 0 °C. Cyclopropyl methyl ketone (0.50 mL, 45 mmol, 1.0 equiv) was added dropwise at 0 °C, then the reaction mixture was allowed to warm to rt and stir for 1 h. In a separate round-bottom flask cooled to 0 °C, H_2O (10. mL) and H_2SO_4 (5.0 mL, 18 M) were added. The Grignard reaction mixture was added to the acid-water mixture at 0 °C and allowed to stir at rt for 1 h. The reaction mixture was extracted with Et_2O (3 x 20 mL). The combined organic layers were washed with brine, dried over Na_2SO_4, and concentrated in vacuo. The compound was used in the next step without further purification. Due to the volatility of compound, residual solvent was not completely removed. **^{1}H NMR** (400 MHz, $CDCl_3$) δ 5.10 (at, J = 7.2 Hz, 1H), 3.11 (t, J = 7.4 Hz, 2H), 2.57 (q, J = 7.3 Hz, 2H), 1.70 (s, 3H), 1.62 (s, 3H). Analytical data is consistent with literature values.[14]

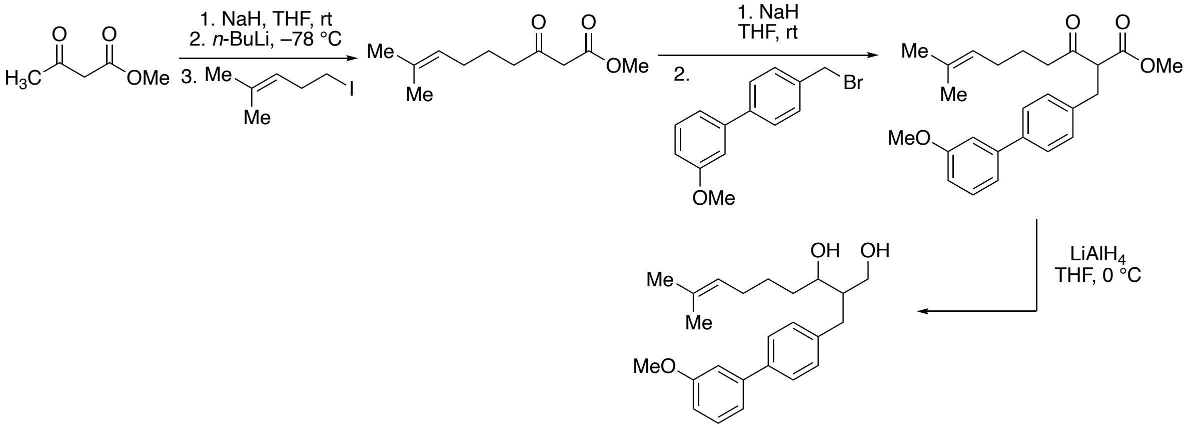

Scheme SI-5. Synthesis of 5-Exo-Trig 1,3-Diol **29a**.

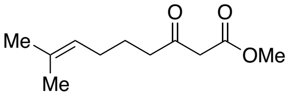

Alkene SI-17 was prepared according to Method J. The following amounts of reagents were used: methyl acetoacetate (0.54 mL, 5.0 mmol, 1.0 equiv), NaH (140 mg, 6.0 mmol, 1.2 equiv), *n*-BuLi (2.4 mL, 6.0 mmol, 1.2 equiv, 2.5 M in hexanes), alkyl iodide **SI-16** (1.4 g, 6.0 mmol, 1.2 equiv), and anhydrous THF (25 mL, 0.20 M in substrate) The residue was purified by flash column chromatography (0–10% EtOAc/hexanes) to afford the title compound as a yellow oil (0.60 g, 3.0 mmol, 61%). **TLC R_f** = 0.5 (10% EtOAc/hexanes); **^{1}H NMR** (400 MHz, $CDCl_3$) δ 5.09–5.05 (m, 1H), 3.74 (s, 3H), 3.44 (s, 2H), 2.52 (t, *J* = 7.4 Hz, 2H), 1.99 (q, *J* =7.3 Hz, 2H), 1.69 (s, 3H), 1.64 (t, *J* = 7.3 Hz, 2H), 1.59 (s, 3H). Analytical data is consistent with literature values.[13]

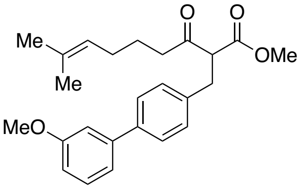

Alkene SI-18 was prepared according to Method K. The following amounts of reagents were used: β-ketoester **SI-17** (0.60 g, 3.0 mmol, 1.0 equiv), NaH (86 mg, 3.6 mmol, 1.2 equiv), benzylic bromide **SI-15** (1.0 g, 3.6 mmol, 1.2 equiv), and anhydrous THF (15 mL, 0.20 M in substrate). The residue was purified by flash column chromatography (0–10% EtOAc/hexanes) to afford the title compound as a clear colorless oil (0.95 g, 2.4 mmol, 81%). **TLC R_f** = 0.6 (10% EtOAc/hexanes); **^{1}H NMR** (400 MHz, $CDCl_3$) δ 7.49 (d, *J* = 8.2 Hz, 2H), 7.33 (t, *J* = 7.9 Hz, 1H), 7.23 (d, *J* = 8.1 Hz, 2H), 7.14 (d, *J* = 7.7 Hz, 1H), 7.09 (at, *J* = 2.0 Hz, 1H), 6.87 (dd, *J* = 8.2, 2.5 Hz, 1H), 5.01 (at, *J* = 7.2 Hz, 1H), 3.85 (s, 3H), 3.82 (t, *J* = 7.6 Hz, 1H), 3.70 (s, 3H), 3.19 (d, *J* = 7.5 Hz, 2H), 2.57–2.49 (m, 1H), 2.40–2.32 (m, 1H), 1.91 (q, *J* = 7.3 Hz, 2H), 1.66 (s, 3H), 1.62–1.58 (m, 2H), 1.55 (s, 3H); **^{13}C NMR** (125.7 MHz, $CDCl_3$) δ 204.8, 169.8, 160.1, 142.4, 139.6, 137.7, 130.6, 129.9, 129.4 (2C), 127.5 (2C), 127.2, 123.6, 119.7, 112.9, 60.5, 55.5, 52.6, 42.4, 33.9, 27.3, 25.8, 23.6, 17.8; **IR** (neat) 2934, 1746, 1715, 1601, 1585, 1220 cm^{-1}; **HRMS** (TOF MS ES+) *m/z*: $[M + Na]^+$ calculated for $C_{25}H_{30}O_4Na$, 417.2042; found 417.2030.

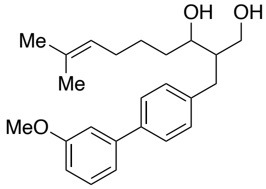

Diol 29a was prepared according to Method L. The following amounts of reagents were used: β-ketoester **SI-18** (0.94 g, 2.4 mmol, 1.0 equiv), $LiAlH_4$ (0.29 g, 7.6 mmol, 3.2 equiv), and anhydrous THF (12 mL, 0.20 M in substrate). The residue was purified by flash column chromatography (0–50% EtOAc/hexanes) to afford the title compound as a mixture of diastereomers as a clear, colorless oil (0.40 g, 1.1 mmol, 46%, 2:1 dr). **TLC R_f** = 0.5 (50% EtOAc/hexanes); **^{1}H NMR** (400 MHz, $CDCl_3$) δ 7.50 (d, *J* = 8.1 Hz, 4H, both), 7.33 (t, *J* = 7.9 Hz, 2H, both), 7.26 (t, *J* = 7.1 Hz, 4H, both), 7.16 (d, *J* = 7.7 Hz, 2H, both), 7.11 (s, 2H, both), 6.87 (dd, *J* =8.2, 2.4 Hz, 2H, both), 5.16–5.08 (m, 2H, both), 3.95–3.92 (m, 2H, both), 3.85 (s, 6H, both), 3.72–3.60 (m, 4H, both), 2.90–2.76 (m, 2H, both), 2.76–2.63 (m, 4H, both), 2.52 (at, *J* =4.6, 2H, both), 2.06–2.01 (m, 4H, both), 1.78 (s, 2H, both), 1.70 (s, 3H, major), 1.68 (s, 3H, minor), 1.65–1.60 (m, 4H, both), 1.61 (s, 3H, major), 1.58 (s, 3H, minor), 1.54–1.45 (m, 2H, both), 1.43–1.33 (m, 2H, both); **^{13}C NMR**

(100 MHz, $CDCl_3$) δ 160.1 (both diastereomers, 2C), 142.6 (both, 2C), 140.1 (both, 2C), 140.0 (both, 2C), 139.0 (both, 2C), 132.0 (both, 2C), 129.8 (both, 2C), 129.6 (both, 2C), 127.3 (both, 4C), 124.4 (both, 2C), 119.6 (both, 4C), 112.9 (major), 112.6 (minor), 75.0 (both, 2C), 64.4 (minor), 62.9 (major), 55.4 (both, 2C), 46.4 (major), 46.0 (minor), 35.5 (major), 35.0 (minor), 33.6 (major), 31.0 (minor), 28.1 (major), 28.0 (minor), 26.6 (major), 26.2 (minor), 25.8 (both, 2C), 17.9 (major), 17.8 (minor) ; **IR** (neat) 3346, 2929, 2857, 1269, 995, 776 cm^{-1}; **HRMS** (TOF MS ES+) *m/z*: $[M + Na]^+$ calculated for $C_{24}H_{32}O_3Na$, 391.2249; found 391.2246.

b) Nickel-catalyzed intramolecular XEC of 5-Exo-Trig Clock Diol **29a**

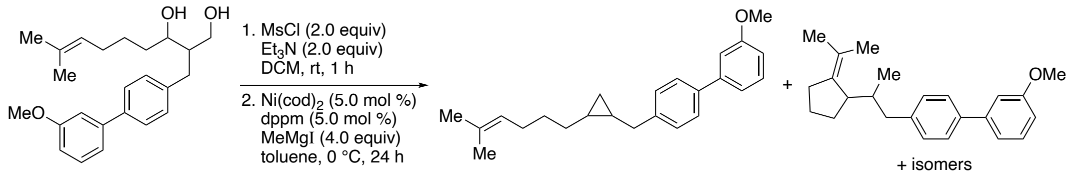

Cyclopropane 30a was prepared following Method G-2. The following amounts of reagents were used: 1,3-diol **29a** (74 mg, 0.20 mmol, 1.0 equiv), anhydrous Et_3N (60. µL, 0.40 mmol, 2.0 equiv), MsCl (30. µL, 0.40 mmol, 2.0 equiv), DCM (1.0 mL, 0.20 M in substrate), $Ni(cod)_2$ (2.8 mg, 10. µmol, 5.0 mol %), dppm (3.8 mg, 10. µmol, 5.0 mol %), PhMe (1.0 mL, 0.20 M in substrate), and MeMgI (0.33 mL, 0.80 mmol, 4.0 equiv). The residue was then purified by flash column chromatography (0–10% EtOAc/hexanes) to afford the title compound as a mixture of cyclopropane diastereomers and clock rearranged products as a clear, colorless oil (18 mg, 54 µmol, 28%, 4.2:1 trans:cis dr cyclopropane, 1.3:1 cyclopropane: rearranged products as determined by GC-MS). **TLC** $\mathbf{R_f}$ = 0.7 (10% EtOAc/hexanes); **^{1}H NMR** (500 MHz, $CDCl_3$) *δ* 7.51 (d, *J* = 8.0 Hz, 4H, both diastereomers), 7.34 (t, *J* = 7.9 Hz, 2H, both diastereomers), 7.30 (d, *J* = 7.9 Hz, 4H, both diastereomers), 7.17 (d, *J* = 7.6 Hz, 2H, both diastereomers), 7.12 (s, 2H, both diastereomers), 6.88 (ad, *J* = 8.1 Hz, 2H, both diastereomers), 5.10–5.07 (m, 2H, both diastereomers), 3.86 (s, 6H, both diastereomers), 2.65–2.53 (m, 4H, both diastereomers), 1.96 (aq, *J* = 7.4 Hz, 4H, both diastereomers), 1.69–1.54 (m, 12H, both diastereomers), 1.39 (aquint, *J* = 7.5 Hz, 4H, both diastereomers), 1.33–1.21 (m, 6H, both diastereomers), 0.78–0.72 (m, 2H, both diastereomers), 0.65–0.59 (m, 1H, trans diastereomer), 0.40–0.36 (m, 1H, trans diastereomer), 0.33–0.29 (m, 1H, trans diastereomer), -0.03 to -0.06 (m, 1H, cis diastereomer); **^{13}C NMR** (125.7 MHz, $CDCl_3$) δ 160.1 (2C, both), 142.9 (2C, both), 141.8 (2C, both), 138.7 (2C, both), 131.3 (2C, both), 129.8 (2C, both), 128.8 (4C, both), 127.1 (4C, both), 125.0 (2C, both), 119.7 (2C, both), 112.9 (2C, both), 112.5 (2C, both), 55.4 (2C, both), 39.8 (2C, both), 33.9 (2C, both), 29.9 (2C, both), 28.0 (2C, both), 25.9 (2C, both), 19.7 (2C, both), 19.1 (2C, both), 17.8 (2C, both), 12.1 (2C, both); **HRMS** (TOF MS ES+) *m/z*: $[M + H]^+$ calculated for $C_{24}H_{30}OH$, 335.2375; found 335.2389.

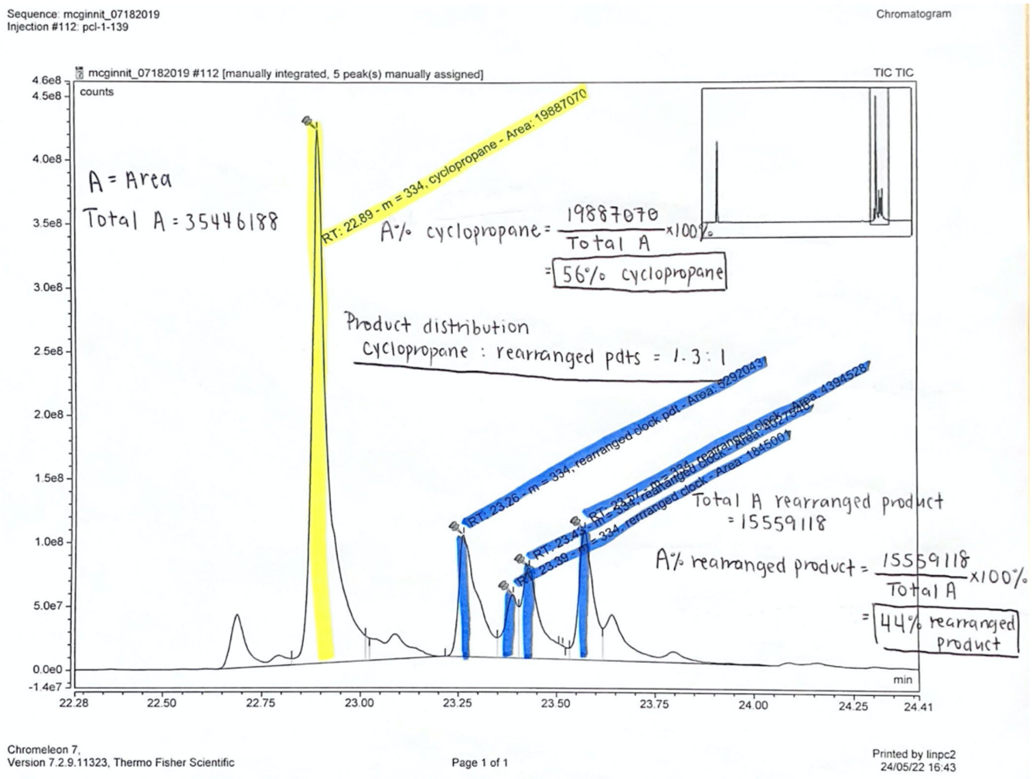

c) Synthesis of 6-Exo-Trig Clock Diol **29b**

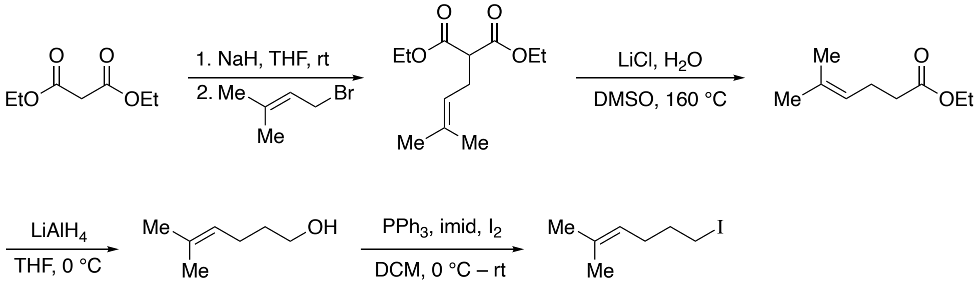

Scheme SI-6. Synthesis of Alkyl Iodide **SI-22.**

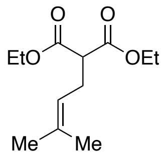

Alkene SI-19 was prepared according to a procedure reported by Kawasaki.[15] To a flame-dried round bottom flask with a stir bar was added NaH (0.26 g, 11 mmol, 1.1 equiv) in a glovebox. The flask was sealed with a septum and brought out of the glovebox. Anhydrous THF (50 mL, 0.20 M in substrate) and diethyl malonate (1.5 mL, 10. mmol, 1.0 equiv) was added dropwise via syringe. The reaction mixture was allowed to stir at rt for 30 min. 1-bromo-3-methylbut-2-ene (1.4 mL, 12 mmol, 1.2 equiv) was added to the reaction mixture at rt and was allowed to stir for 16 h. To quench, saturated aqueous NH_4Cl (50 mL) was added. The reaction mixture was extracted with Et_2O (3 x 50 mL), and the combined organic layers were washed with brine, dried over Na_2SO_4, and concentrated in vacuo. The residue was purified by flash column chromatography (0–5%

EtOAc/hexanes) to afford the title compound as a colorless oil (1.7 g, 7.5 mmol, 75%). **TLC R$_f$** = 0.3 (5% EtOAc/hexanes); **^{1}H NMR** (400 MHz, $CDCl_3$) δ 5.07 (at, *J* = 7.3 Hz, 1H), 4.18 (q, *J* = 7.1 Hz, 4H), 3.32 (t, *J* = 7.7 Hz, 1H), 2.58 (at, *J* = 7.5 Hz, 2H), 1.68 (s, 3H), 1.63 (s, 3H), 1.26 (t, *J* = 7.1 Hz, 6H). Analytical data is consistent with literature values.[15]

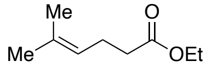

Ester SI-20 was prepared according to a method reported by Fuess.[16] To a round bottom flask with a stir bar was added **SI-19** (1.8 g, 7.8 mmol, 1.0 equiv), DMSO (13 mL, 0.60 M in substrate), DI H_2O (0.14 mL, 7.8 mmol, 1.0 equiv), and LiCl (0.99 g, 23 mmol, 3.0 equiv). The flask was equipped with a reflux condenser, and the reaction mixture was heated to 160 °C and stirred for 3 h. The reaction mixture was allowed to cool to rt, and then poured onto a mixture crushed ice and brine. The mixture was concentrated in vacuo, diluted with Et_2O, and washed with H_2O. The aqueous layer was extracted with Et_2O (x 50 mL). The combined organic layers were dried over Na_2SO_4 and concentrated in vacuo. The residue was purified by flash column chromatography (0–10% EtOAc/Hex) to afford the title compound as a colorless oil (0.74 g, 4.8 mmol, 61%). **TLC R$_f$** = 0.6 (10% EtOAc/hexanes); **^{1}H NMR** (400 MHz, $CDCl_3$) δ 5.09 (br s, 1H), 4.13 (q, *J* = 7.1 Hz, 2H), 2.31–2.30 (m, 4H), 1.68 (s, 3H), 1.62 (s, 3H), 1.25 (t, *J* = 7.1 Hz, 3H). Analytical data is consistent with literature values.[16]

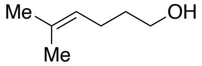

Alcohol SI-21 was prepared according to Method K. The following amounts of reagents were used: ester **SI-20** (0.74 g, 4.8 mmol, 1.0 equiv), $LiAlH_4$ (0.40 g, 10.5 mmol, 2.2 equiv), and anhydrous THF (24 mL, 0.20 M in substrate). The residue was purified by flash column chromatography (0–20% EtOAc/hexanes) to afford the title compound as a mixture of diastereomers as a clear, colorless oil (0.40 g, 3.5 mmol, 75%). **TLC R$_f$** = 0.3 (20% EtOAc/hexanes); **^{1}H NMR** (400 MHz, $CDCl_3$) δ 5.13 (tt, *J* = 10.8, 1.4 Hz, 1H), 3.65 (br s, 2H), 2.10–2.04 (m, 2H), 1.69 (s, 3H), 1.62 (s, 3H), 1.61 (aquint, *J* = 6.9 Hz, 2H), 1.29 (br s, 1H). Analytical data is consistent with literature values.[17]

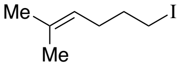

Iodide SI-22 To a flame-dried round bottom flask with a stir bar was added PPh_3 (1.0 g, 3.9 mmol, 1.1 equiv) and imidazole (0.29 g, 4.3 mmol, 1.2 equiv). DCM (18 mL, 0.20 M in substrate) was added, followed by alcohol **SI-21** (0.40 g, 3.5 mmol, 1.0 equiv). The reaction was allowed to stir at rt for 30 min before cooling to 0 °C. While at 0 °C, the septum was opened slightly to add I_2 (1.2 g, 4.8 mmol, 1.4 equiv) in small portions while keeping it under an atmosphere of N_2. Upon complete addition of I_2, the reaction mixture was allowed to stir at 0 °C for 2 h. To quench, saturated aqueous $Na_2S_2O_3$ (20 mL) was added at 0 °C. The reaction mixture was extracted with DCM (3 x 20 mL), and the combined organic layers were washed with brine, dried over Na_2SO_4, and concentrated in vacuo. The residue was purified by flash column chromatography (100% hexanes) to afford the title compound as a colorless oil (0.65 g, 2.9 mmol, 82%). **TLC R$_f$** = 0.7 (100% hexanes); **^{1}H NMR** (400 MHz, $CDCl_3$) δ 5.06 (tt, *J* = 10.8, 1.4 Hz, 1H), 3.18 (t, *J* = 7.0 Hz, 2H), 2.09 (q, *J* = 7.1 Hz, 2H), 1.89–1.82 (m, 2H), 1.69 (s, 3H), 1.64 (s, 3H). Analytical data is consistent with literature values.[18]

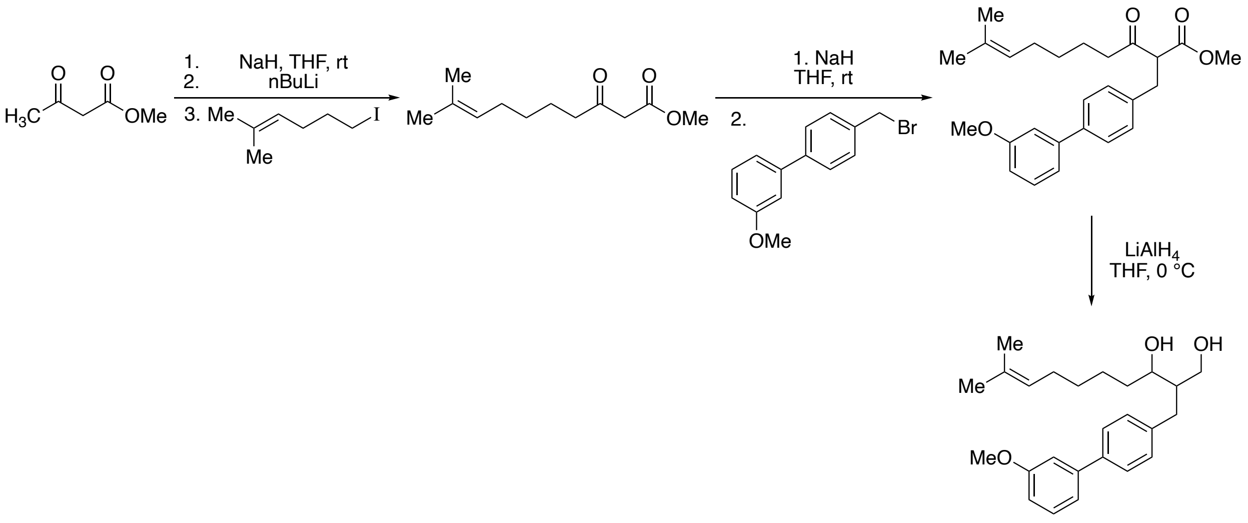

Scheme SI-7. Synthesis of 6-Exo-Trig 1,3-Diol **29b**.

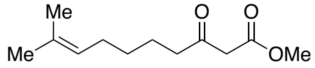

Alkene SI-23 was prepared according to Method J. The following amounts of reagents were used: methyl acetoacetate (0.24 mL, 2.3 mmol, 1.0 equiv), NaH (65 mg, 2.7 mmol, 1.2 equiv), *n*-BuLi, (1.1 mL, 2.7 mmol, 1.2 equiv, 2.5 M in hexanes), alkyl iodide **SI-22** (0.61 g, 2.7 mmol, 1.2 equiv), and anhydrous THF (11 mL, 0.20 M in substrate). The residue was purified by flash column chromatography (0–10% EtOAc/hexanes) to afford the title compound as a pale-yellow oil (0.34 g, 1.6 mmol, 71%). **TLC R$_f$** = 0.4 (10% EtOAc/hexanes); **^{1}H NMR** (500 MHz, $CDCl_3$) δ 5.09 (at, J = 7.0 Hz, 1H), 3.73 (s, 3H), 3.44 (s, 2H), 2.53 (t, J = 7.4 Hz, 2H), 1.98 (aq, J = 7.2 Hz, 2H), 1.68 (s, 3H), 1.63–1.58 (m, 5H), 1.36–1.30 (m, 2H); **^{13}C NMR** (125.7 MHz, $CDCl_3$) δ 202.9, 167.8, 132.0, 124.2, 52.5, 49.2, 43.2, 29.3, 27.8, 25.8, 23.3, 17.8; **HRMS** (TOF MS ES+) *m/z*: [M + Na]$^+$ calculated for $C_{12}H_{20}O_3Na$, 235.1310; found 235.1309.

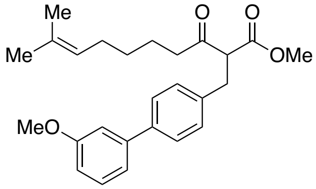

Alkene SI-24 was prepared according to Method K. The following amounts of reagents were used: β-ketoester **SI-23** (0.34 g, 1.6 mmol, 1.0 equiv), NaH (46 mg, 1.9 mmol, 1.2 equiv), benzylic bromide **SI-15** (0.53 g, 1.9 mmol, 1.2 equiv), and anhydrous THF (8.0 mL, 0.20 M in substrate). The residue was purified by flash column chromatography (0–10% EtOAc/hexanes) to afford the title compound as a clear colorless oil (0.23 g, 0.57 mmol, 34%). **TLC R$_f$** = 0.3 (10% EtOAc/hexanes); **^{1}H NMR** (500 MHz, $CDCl_3$) δ 7.49 (d, J = 8.0 Hz, 2H), 7.34 (t, J = 7.9 Hz, 1H), 7.24–7.14 (m, 3H), 7.09 (s, 1H), 6.87 (d, J = 8.1 Hz, 1H), 5.05 (at, J = 7.0 Hz, 1H), 3.86 (s, 3H), 3.83 (t, J = 7.6 Hz, 1H), 3.70 (s, 3H), 3.19 (d, J = 7.5 Hz, 2H), 2.54 (tt, J = 17.5, 7.3 Hz, 1H), 2.36 (tt, J = 17.3, 7.3 Hz, 1H), 1.92 (q, J = 7.2 Hz, 2H), 1.66 (s, 3H), 1.59–1.49 (m, 5H), 1.27–1.21 (m, 2H); **^{13}C NMR** (125.7 MHz, $CDCl_3$) δ 204.7, 169.7, 160.1, 142.4, 139.6, 137.6, 130.6, 129.9, 129.3 (2C), 127.5 (2C), 127.2, 124.3, 119.6, 112.9, 60.5, 55.4, 52.6, 43.0, 33.9, 29.2, 27.8,

25.8, 23.1, 17.8; **HRMS** (TOF MS ES+) *m/z*: $[M + Na]^+$ calculated for $C_{26}H_{32}O_4Na$, 431.2198; found 431.2199.

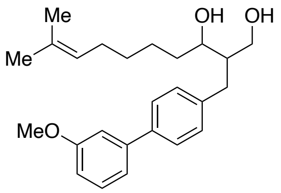

1,3-Diol 29b was prepared according to Method L. The following amounts of reagents were used: *β*-ketoester **SI-24** (0.23 g, 0.55 mmol, 1.0 equiv), $LiAlH_4$ (67 mg, 1.8 mmol, 3.2 equiv), and anhydrous THF (2.8 mL, 0.20 M in substrate). The residue was purified by flash column chromatography (0–50% EtOAc/hexanes) to afford the title compound as a mixture of diastereomers as a clear, colorless oil (150 mg, 0.39 mmol, 70.%, 1:1 dr). **TLC** $\mathbf{R_f}$ = 0.6 (50% EtOAc/hexanes); **^{1}H NMR** (500 MHz, $CDCl_3$) *δ* 7.52 (d, *J* = 8.0 Hz, 4H, both diastereomers), 7.34 (t, *J* = 7.9 Hz, 2H, both), 7.28 (d, *J* = 7.9 Hz, 4H, both), 7.17 (d, *J* = 7.6 Hz, 2H, both), 7.11 (s, 2H, both), 6.89 (d, *J* = 8.2 Hz, 2H, both), 5.15–5.09 (m, 2H, both), 3.96 (dd, *J* = 11.0, 2.8 Hz, 2H, both), 3.86 (s, 6H, both), 3.75–3.72 (m, 2H, both), 3.65–3.62 (m, 2H, both), 2.91–2.72 (m, 4H, both), 2.38 (br s, 4H, both), 2.02–1.96 (m, 4H, both), 1.80 (br s, 2H, both), 1.70–1.58 (m, 16H, both), 1.49–1.31 (m, 8H, both); **^{13}C NMR** (125.7 MHz, $CDCl_3$) δ 160.1 (both diastereomers, 2C), 142.7 (both, 2C), 139.8 (both, 2C), 139.0 (both, 2C), 131.7 (both, 2C), 129.9 (both, 2C), 129.7 (both, 4C), 129.6 (both, 2C), 127.3 (both, 4C), 124.6 (both, 2C), 119.7 (both, 2C), 112.9 (both, 2C), 75.2 (both, 2C), 63.0 (both, 2C), 55.4 (both, 2C), 46.1 (both, 2C), 35.9 (both, 2C), 35.0 (both, 2C), 30.0 (both, 2C), 28.1 (both, 2C), 25.9 (both, 2C), 25.7 (both, 2C), 17.8 (both, 2C); **HRMS** (TOF MS ES+) *m/z*: $[M + Na]^+$ calculated for $C_{25}H_{34}O_3Na$, 405.2406; found 405.2409.

d) Nickel-catalyzed intramolecular XEC of 6-Exo-Trig Clock Diol **29b**

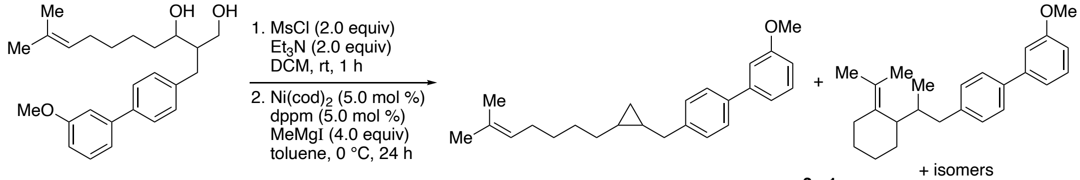

3 : 1

Cyclopropane 30b was prepared following Method G-2. The following amounts of reagents were used: 1,3-diol **29b** (75 mg, 0.20 mmol, 1.0 equiv), anhydrous Et_3N (54. µL, 0.39 mmol, 2.0 equiv), MsCl (30. µL, 0.39 mmol, 2.0 equiv) DCM (0.98 mL, 0.20 M in substrate), $Ni(cod)_2$ (2.7 mg, 9.8 µmol, 5.0 mol %), dppm (3.7 mg, 9.8 µmol, 5.0 mol %), PhMe (0.98 mL, 0.20 M in substrate), and MeMgI (0.28 mL, 0.78 mmol, 4.0 equiv). The residue was then purified by flash column chromatography (0–10% EtOAc/hexanes) to afford the title compound as a mixture of cyclopropane diastereomers and clock rearranged products as a clear, colorless oil (34 mg, 98 µmol, 46%, 8:1 trans:cis dr, 3:1 cyclopropane: rearranged products as determined by GC-MS). **TLC** $\mathbf{R_f}$ = 0.5 (10% EtOAc/hexanes); **^{1}H NMR** (500 MHz, $CDCl_3$) *δ* 7.51 (d, *J* = 8.2 Hz, 4H, both diastereomers), 7.33 (t, *J* = 7.9 Hz, 2H, both diastereomers), 7.30 (d, *J* = 8.1 Hz, 4H, both diastereomers), 7.17 (d, *J* = 8.1 Hz, 2H, both diastereomers), 7.12 (s, 2H, both diastereomers), 6.87 (ad, *J* = 8.2 Hz, 2H, both diastereomers), 5.09 (br s, 2H, both diastereomers), 3.85 (s, 6H, both diastereomers), 2.59–2.57 (m, 4H, both diastereomers), 2.02–1.91 (m, 4H, both diastereomers), 1.70 (s, 3H, cis diastereomer), 1.67 (s, 3H, trans diastereomer), 1.60 (s, 3H, cis

diastereomer), 1.58 (s, 3H, trans diastereomer), 1.45–1.21 (m, 12H, both diastereomers), 0.91–0.76 (m, 2H, cis diastereomer), 0.75–0.71 (m, 2H, both diastereomers), 0.64–0.58 (m, 1H, trans diastereomer), 0.37 (tt, J = 8.7, 4.4 Hz, 1H, trans diastereomer), 0.30 (tt, J = 8.2, 4.7 Hz, 1H, trans diastereomer), -0.04 to -0.08 (m, 1H, cis diastereomer); **^{13}C NMR** (125.7 MHz, $CDCl_3$) δ 160.1 (2C, both), 142.9 (2C, both), 141.8 (2C, both), 138.7 (2C, both), 131.3 (2C, both), 129.8 (2C, both), 128.8 (4C, both), 127.2 (2C, both), 127.1 (4C, both), 125.0 (2C, both), 119.8 (2C, both), 112.9 (trans diastereomer), 112.5 (cis diastereomer), 55.4 (2C, both), 39.8 (2C, both), 34.2 (2C, both), 29.8 (2C, both), 29.4 (2C, both), 28.2 (2C, both), 25.9 (2C, both), 19.7 (2C, both), 19.1 (2C, both), 17.8 (2C, both), 12.1 (2C, both); **HRMS** (TOF MS CI+) *m/z*: $[M]^+$ calculated for $C_{25}H_{32}O$, 348.2453; found 348.2446.

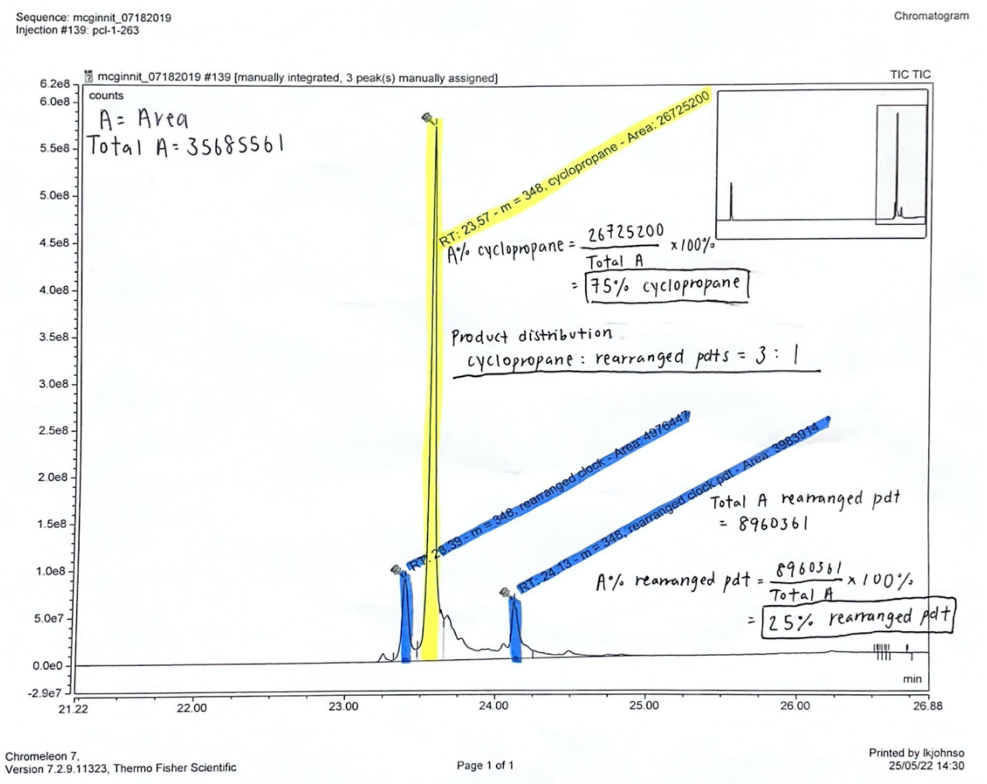

VI. Computational Data

a) Conformational Analysis

Regarding the conformational analysis, for each structure, an extensive conformational search was performed with CREST (which is abbreviated from Conformer–Rotamer Ensemble Sampling Tool)[19] to locate the lowest-energy conformer of intermediates and transition states.

b) Complexation between Dimesylate and Mg(II) Species

The complexation of two Mg(II) species in **9** reduced molecular polarity, as can be seen from its lower dipole moment (μ = 5.49 D), and thus it is stabilized in a low polar toluene solvent, in

comparison to other highly polar complexes with one Mg(II) species association (Scheme SI-8a to SI-8e, **9** v.s. **4**, **5**, **7**, **8**).

With the involvement of two Mg(II) species, in addtion to complex **9**, we also investigated a related complex with two MgI_2 species coordinated, namely **S1**. As shown in Scheme SI-8f, the energy of complex **S1** is slightly lower than that of complex **9** by 0.9 kcal/mol, which is due to the stronger Lewis acidity of magnesium iodide, and thus its stronger complexation with mesylate moiety. However, in the actual reaction system, magnesium iodide is present in a catalytic amount. Therefore, in the exploration of reaction mechanism, we adopt one magnesium iodide and one Grignard reagent to complex with the substrate, and then the MeMgI-dimesylate-MgI_2 complex **9** was identified as the active species which enters into the catalytic cycle for the 1,3-diiodide intermediate formation.

(1) with the involvement of one Mg(II) species

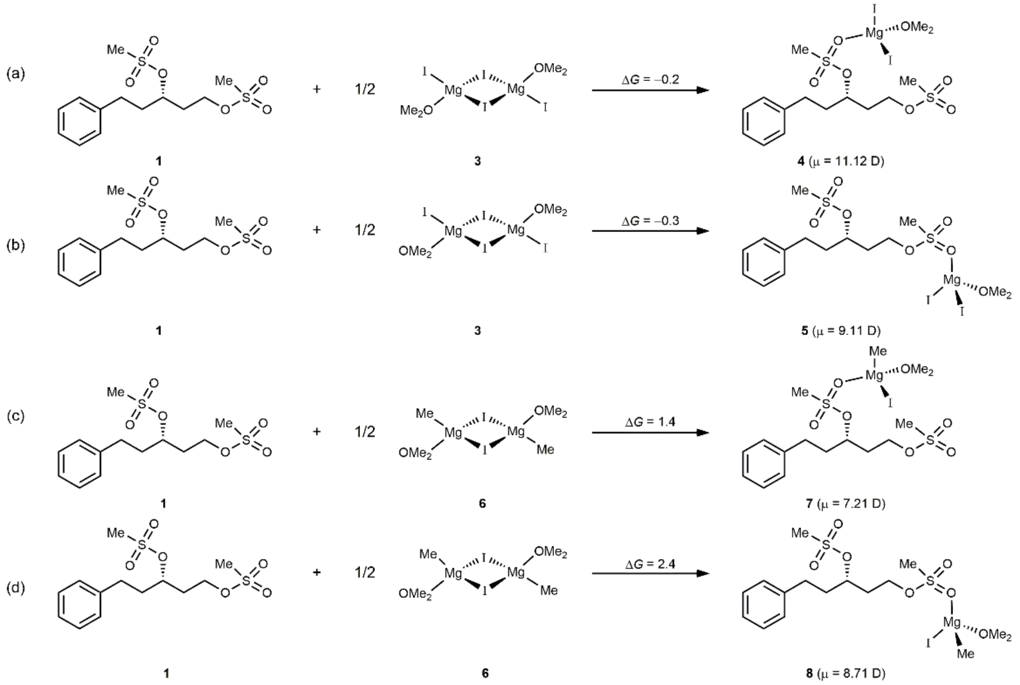

(2) with the involvement of two Mg(II) species

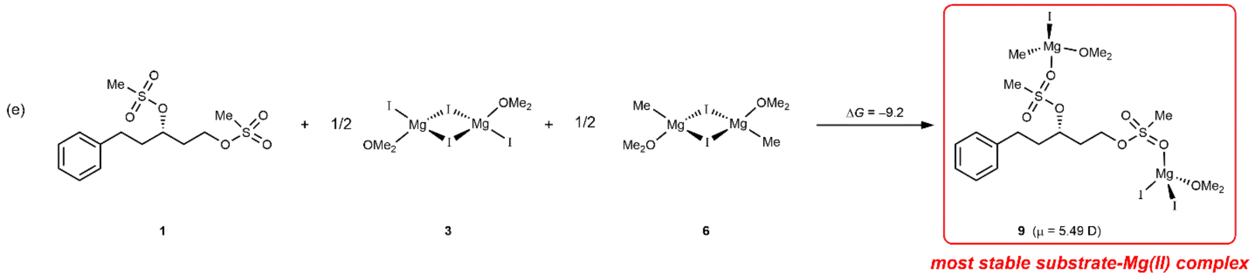

(3) transformation of complex 9 to S1

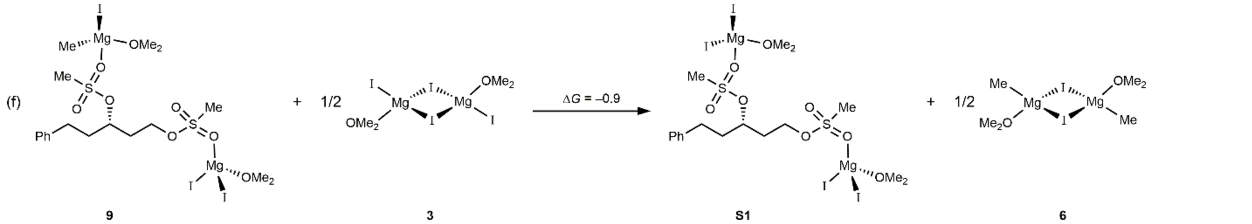

<u>Scheme SI-8.</u> Free energy changes of complexation between substrate and Mg(II) species involved in dimesylate/MeMgI system. Free energies in toluene are in kcal/mol.

<u>c). Origins of the Regioselectivity for the First Iodide Displacement</u>

For the 1,3-diiodide formation, the competition between **TS10** and **TS12** (Figure 1) determines the regioselectivity of the first C−I bond formation, in which the secondary alkyl iodide formation is favored (via **TS12**). To explore the origins of regioselectivity, we constructed model reactions (Figure SI-1a and SI-1b) to explore whether chelation has an effect on selectivity. As shown in Figure SI-1a and SI-1b, secondary C−I bond formation via **TS-S5** is more favorable than the primary C−I bond formation through **TS-S3**, this selectivity is consistent with the observation in the dimesylate/MeMgI system, indicating that chelation had little effect on selectivity and other factors were crucial.

Based on **TS-S3** and **TS-S5**, we performed distortion/interaction analysis (Figure SI-1c and SI-1d).[2021] Each transition state was separated into two fragments: MeMgI$(OMe_2)_2$ catalyst fragment and the monomesylate−MgI_2 substrate fragment. The distortion energy (ΔE_{dist}) is the energy required for the geometric change during the C−I bond formation, and the interaction energy (ΔE_{int}) reflects the strength of the interaction between the Mg(II) and substrate fragment in the transition state. The reference points are the optimized MeMg$(OMe)_2$I and substrate (**S2** or **S4**).

Comparing the two C–I bond formation transition states, **TS-S3** and **TS-S5**. $\Delta E_{dist\text{-}sub}$ is the leading causes for the regioselectivity. Substrate in **TS-S3** has greater distortion than that of in **TS-S5**, thus, the stability of carbocation determines the selectivity (S_N1 nature); It is because that iodine derived from Mg(II) species is not a stronger nucleophile, thus, nucleophilic attack to break the C–O bond ($\rightarrow S_N2$) is very weak and ionization ($\rightarrow S_N1$) may occur, making **TS-S3** and **TS-S5** “loose” transition states, which have bond forming lagging behind bond breaking, so that negative charge is localized on the leaving group and positive charge is localized on the carbon being attacked.[22] Overall, the stability of carbocation determines the selectivity of C–I bond formation, eventually leading to the regioselectivity in the first iodide displacement.

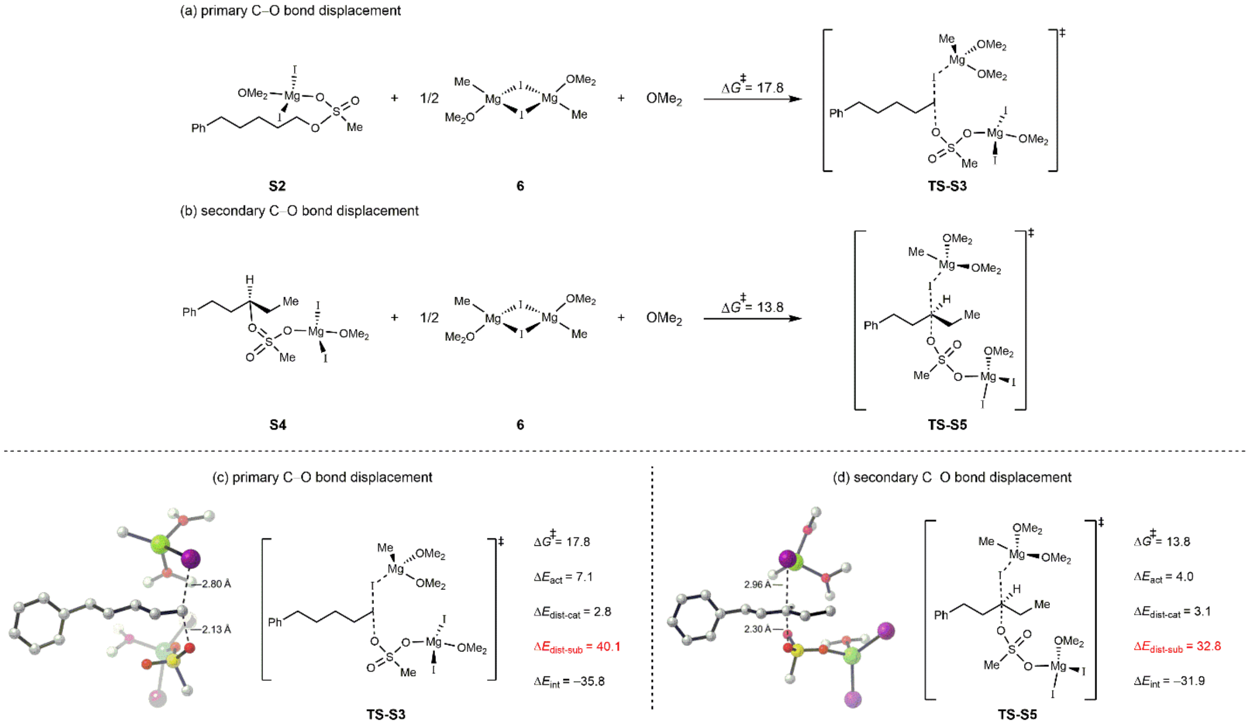

Figure SI-1. Origins of the regioselectivity for the first iodide displacement. Energies are in kcal/mol.

d). Free Energy Diagram of MgI_2-Mediated Iodide Displacement of Dimesylate **1**.

The free energy changes of the most favorable pathway of MgI_2-mediated iodide displacement with dimesylate **1** are shown in Figure SI-2. Figure SI-2 indicated that the on-cycle resting state is the dimesylate-Mg(II) complex **S1**, and the rate-limiting step for the iodide displacement is the first C–I bond formation via **TS-S7** with an overall barrier of 18.3 kcal/mol. Consistent with the experimental observations, displacement of the dimesylate by MgI_2 has a higher barrier (18.3 kcal/mol) than that of by Grignard reagent (17.7 kcal/mol, Figure 2), highlighting the importance of nucleophilicity of Mg(II) species in promoting such a process.

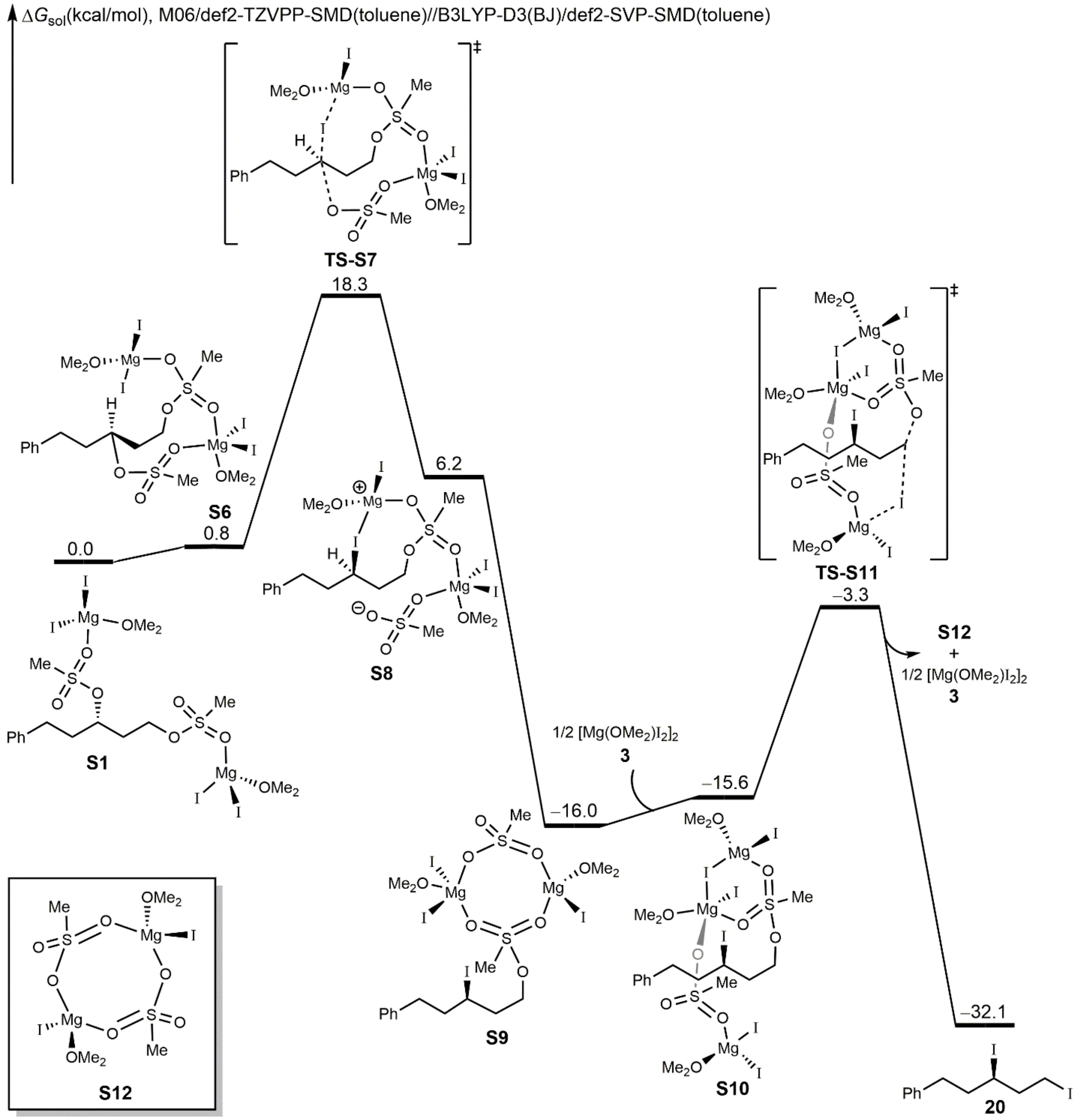

Figure SI-2. DFT-computed free energy changes of MgI_2-mediated iodide displacement of dimesylate **1**.

e). Exploration of the Possibility of 1,3-Diiodide Racemization

We explored different ways of C–I bond displacement. **TS-S13** and **TS-S15** are the transition states of C–I bond replacement at the secondary alkyl center, while **TS-S14** and **TS-S16** lead to C–I bond replacement at the primary alkyl center. In these competitive transition states, Grignard reagent acts as iodine donor, and magnesium iodide acts as Lewis acid to assist the departure of iodide ions. Different from **TS-S13** and **TS-S14**, both **TS-S15** and **TS-S16** form unique iodine bridges. As shown in Scheme SI-9, **TS-S13** is the most favorable transition state to promote the

C−I bond replacement with 18.9 kcal/mol energy barrier. However, it is still higher in energy than iodine atom abstraction catalyzed by nickel via **TS33** (the corresponding energy barrier is 15.1 kcal/mol, Figure 3). Therefore, 1,3-diiodide racemization facilitated by the attack of Mg(II) reagent is less favorable.

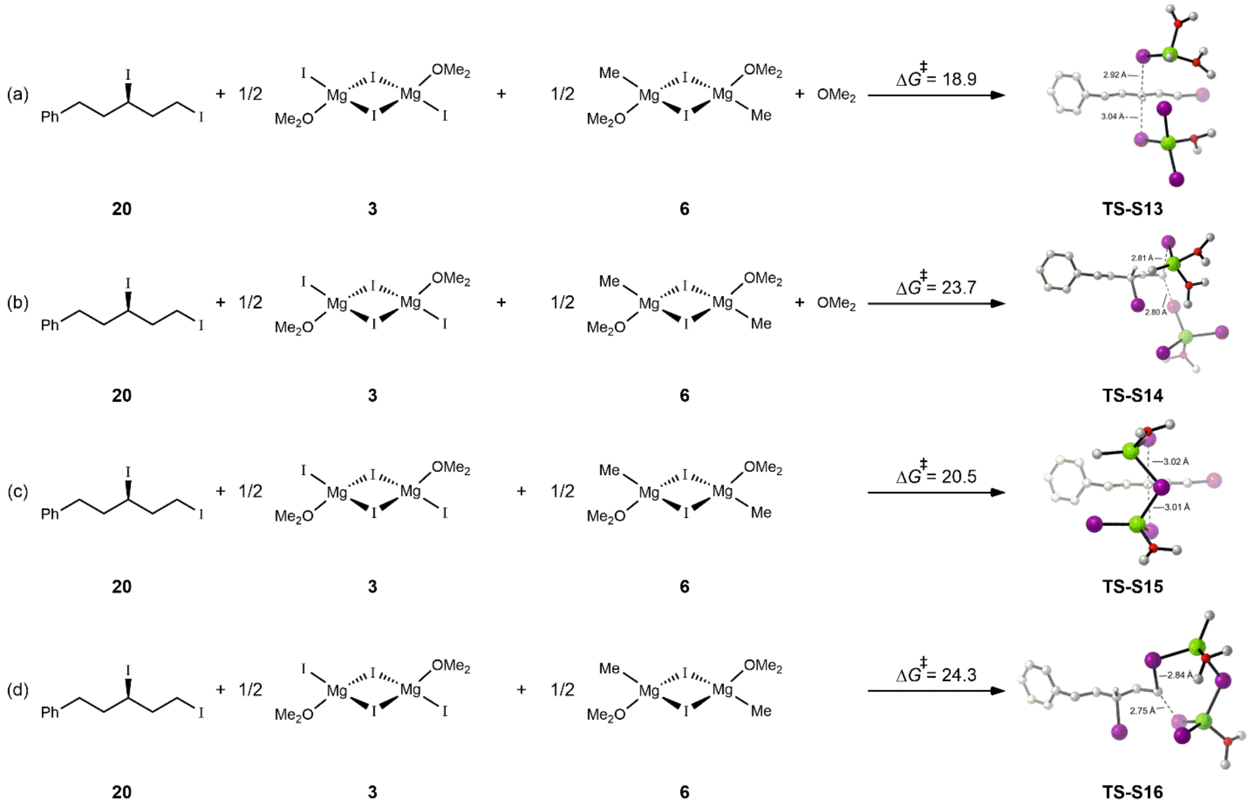

Scheme SI-9. Iodide displacement occurs on 1,3-diiodide. Free energies in toluene are in kcal/mol.

f). Discussions on Different Spin States of Key Transition States and Intermediates

In the reaction mechanism shown in Figure 3, for the iodine atom abstraction step via **TS33**, this process is believed to occur with an open-shell singlet transition state.[23–25] Subsequently, the recombination between secondary alkyl radical and Ni(I) species takes place also via an open-shell singlet transition state (**TS37**), this is because the triplet state of **38** is significantly higher in energy compared to the corresponding singlet structure. Similarly, for intermediate **41**, the triplet structure is also more energetic than the corresponding singlet state (Scheme SI-10). Therefore, the homolysis cleavage of the Ni–C bond of **41** occurs with an open-shell singlet transition state **TS46**.

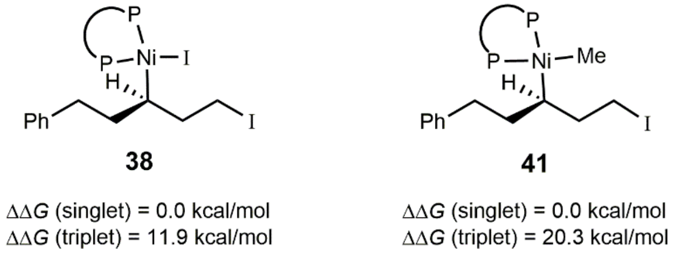

Scheme SI-10. Energy difference of the singlet and triplet structures of intermediates **38** and **41**.

g). Alternative Transition States of Radical Cyclization

In addition to **TS45**, we also calcualted an alternative transition state **TS-S17**, which is higher in energy than **TS45** by 15.4 kcal/mol. Therefore, **TS45** is the most favorable transition state leading to the radical cyclization.

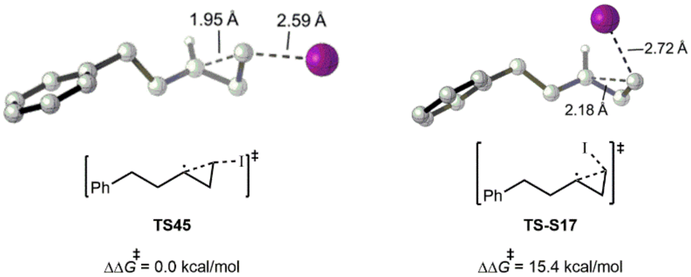

Figure SI-3. Optimized structures and free energies of alternative transition states of radical cyclization.

h). Explorations of the Possibility of Single Electron Transfer Process

We computational investigated the single electron transfer (SET) process occurs between Ni(I) species (**35** or **36**) and the secondary alkyl radical (**34**). We assume that this process will generate Ni(II) (**35*** or **36***) and alkyl anionic species. We successfully have located complexes **35*** and **36***. However, during the geometry optimization, we couldn't obtain the alkyl anionic species deriving from secondary alkyl radical, instead, the cyclopropane product (**34***) is formed directly. This may be because the corresponding alkyl anionic species are less stable and thus spontaneous cyclization occurs, generating **34***.
Both Scheme SI-11a and SI-11b show that the hypothetical SET process is endothermic, and the corresponding reaction energy is higher than the energy barrier of radical recombination (Figure 3, via **TS37**). Therefore, single electron transfer is not feasible in this studied reaction system.

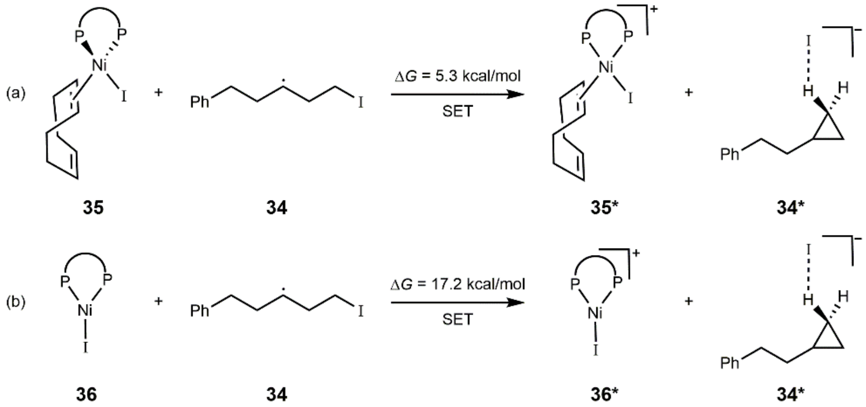

Scheme SI-11. Single electron transfer process occurs between Ni(I) species and the secondary alkyl radical.

i). Explorations of the Alternative Transmetalation Pathways

Grignard reagent is consumed one equivalent upon formation of alkyl iodide (Figure 2), and since two equivalent Grignard reagents were used in the experiment, one equivalent of MeMgI should still be available for the subsequent steps. In the Ni-catalyzed cycle (Figure 3 and Figure SI-8), transmetalation needs to occur twice (Figure 3, **38** to **41**; Figure SI-8, **44** to **S20**), thus, the transmetalation reagents could be MeMgI and MeMgOMs. The latter originates from complex **19**, which is a by-product of the diiodide formation process.

For the first transmetalation step (Figure 3, **38** to **41**), we investigated two possible transition states, **TS40** and **TS40***, corresponding to using MeMgI (black path) and MeMgOMs (red path) as transmetalation reagents, respectively. As shown in Figure SI-4, **TS40*** is higher in energy than **TS40** by 10.7 kcal/mol. Because the transmetalation via **TS40** is facile and irreversible (Figure 3), this process occurs readily with MeMgI as transmetalation reagent (Figure SI-4, black path). Therefore, in the second transmetalation step (Figure SI-8, **44** to **S20**), the available transmetalation reagent should be MeMgOMs (Figure SI-8).

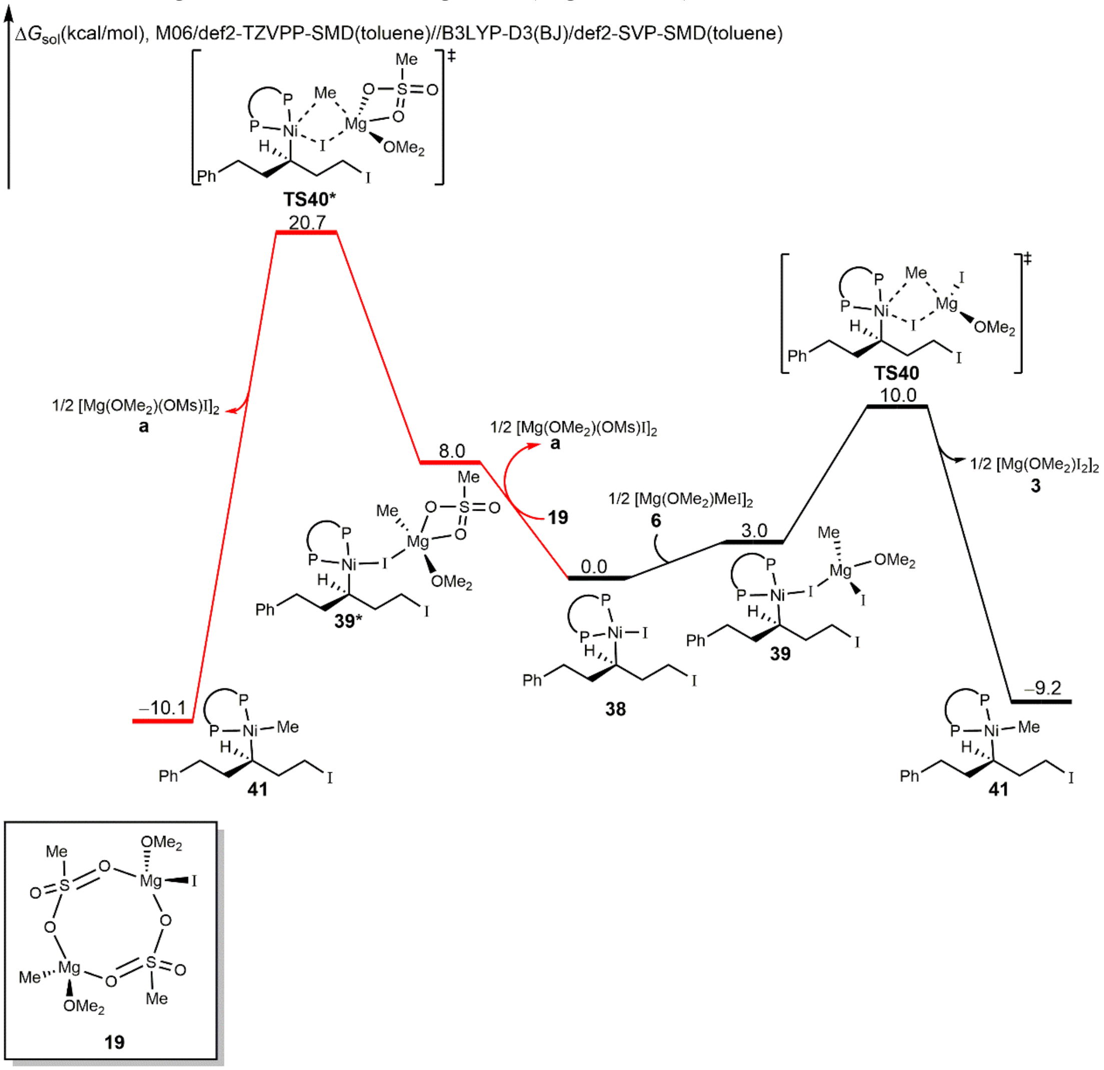

Figure SI-4. Competing transmetalation pathways starting from intermediate **38**.

j). Discussions on the C–Ni Bond Homolysis Process

As shown in Figure 3, the computed free energy changes of C–Ni bond homolysis (via **TS37**) from intermediate **38** (i.e. the reverse reaction to intermediate **36**) is 17.0 kcal/mol. However, similar C–Ni homolysis (via **TS46**) from **41** is 29.9 kcal/mol. We performed distortion/interaction analysis[20,21] to investigate the origins of energy difference for the C–Ni bond homolysis starting from different intermediates (**38** or **41**).

Based on **TS37** and **TS46**, each transition state was separated into two fragments: catalyst fragment (for **TS37**, the catalyst fragment is (dppm)NiI; and for **TS46**, the catalyst fragment is (dppm)Ni(Me)) and the secondary alkyl radical substrate fragment. The distortion energy (ΔE_{dist}) is the energy required for the geometric change during the Ni−C bond cleavage, and the interaction energy (ΔE_{int}) reflects the strength of the interaction between the nickel catalyst and substrate fragment in the transition state. For **TS37** and **TS46,** the reference points are intermediates **38** and **41**, respectively.

As shown in Figure SI-5, both distortion and interaction favor the formation of **TS37**. Comparing **TS37** and **TS46**, the difference in distortion energy is mainly due to the catalyst part. In **TS46**, the steric repulsion between the catalyst and the substrate (highlighted H−H bond distance is 2.12 Å) causes the catalyst to show a large distortion. The interaction energy is unfavorable for either **TS37** or **TS46**, where in **TS46** the interaction energy is even more disfavored. This is because, compared to the reference point (**38** or **41**), the bond homolysis destroys the originally stable C–Ni bond in **38** or **41**. As shown in Scheme SI-12, the C–Ni bond in intermediate **41** is stronger than that of in **38**, and thus in **TS46**, corresponds to a more unfavorable interaction energy. Overall, both catalyst distortion and unfavorable interaction contribute to the energy difference of C–Ni bond homolysis based on different intermediates.

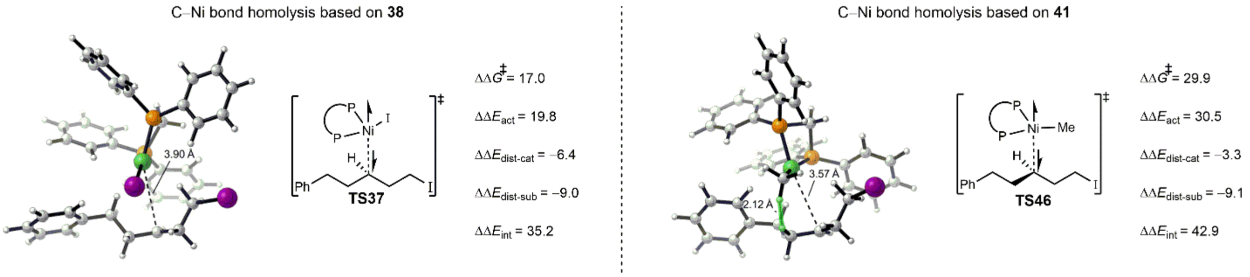

Figure SI-5. Distortion/interaction analysis of C–Ni bond homolysis transition states. For **TS37** and **TS46**, the reference points are **38** and **41**, respectively. Energies are in kcal/mol.

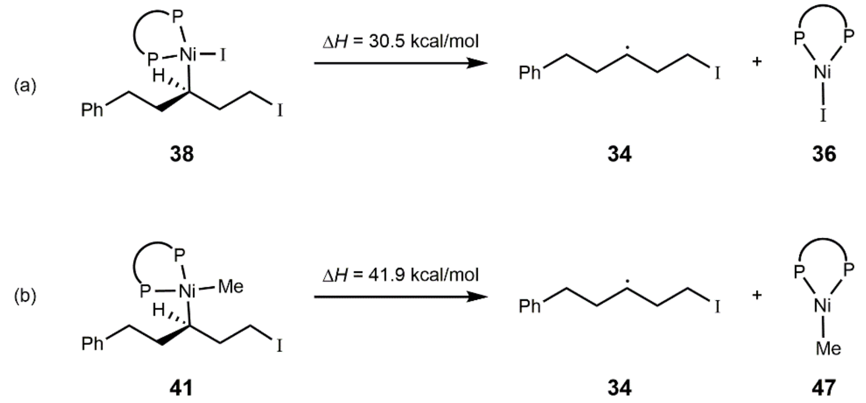

Scheme SI-12. Bond-dissociation energies of intermediates **38** and **41**.

k). Computational Investigation of the Reaction Profile for Product Formation by Using Different Methods

In addition to the current computational method (Method I) used in the manuscript, we employ two other methods (Methods II and III) to study the reaction profile shown in Figure 3. The free energy diagrams given by all computational methods are very similar (Figures 3, Figure SI-6 and Figure SI-7), and we choose M06 as the final method for single-point energy calculation because it is robust in describing the energetics of similar types of structure.[26,27]

Method I: M06/def2-TZVPP-SMD(toluene)//B3LYP-D3(BJ)/def2-SVP-SMD(toluene)

Method II: ωB97X-D[28]/def2-TZVPP-SMD(toluene)//B3LYP-D3(BJ)/def2-SVP-SMD(toluene)

Method III: PBE0-D3(BJ)[29]/def2-TZVPP-SMD(toluene)//B3LYP-D3(BJ)/def2-SVP-SMD(toluene)

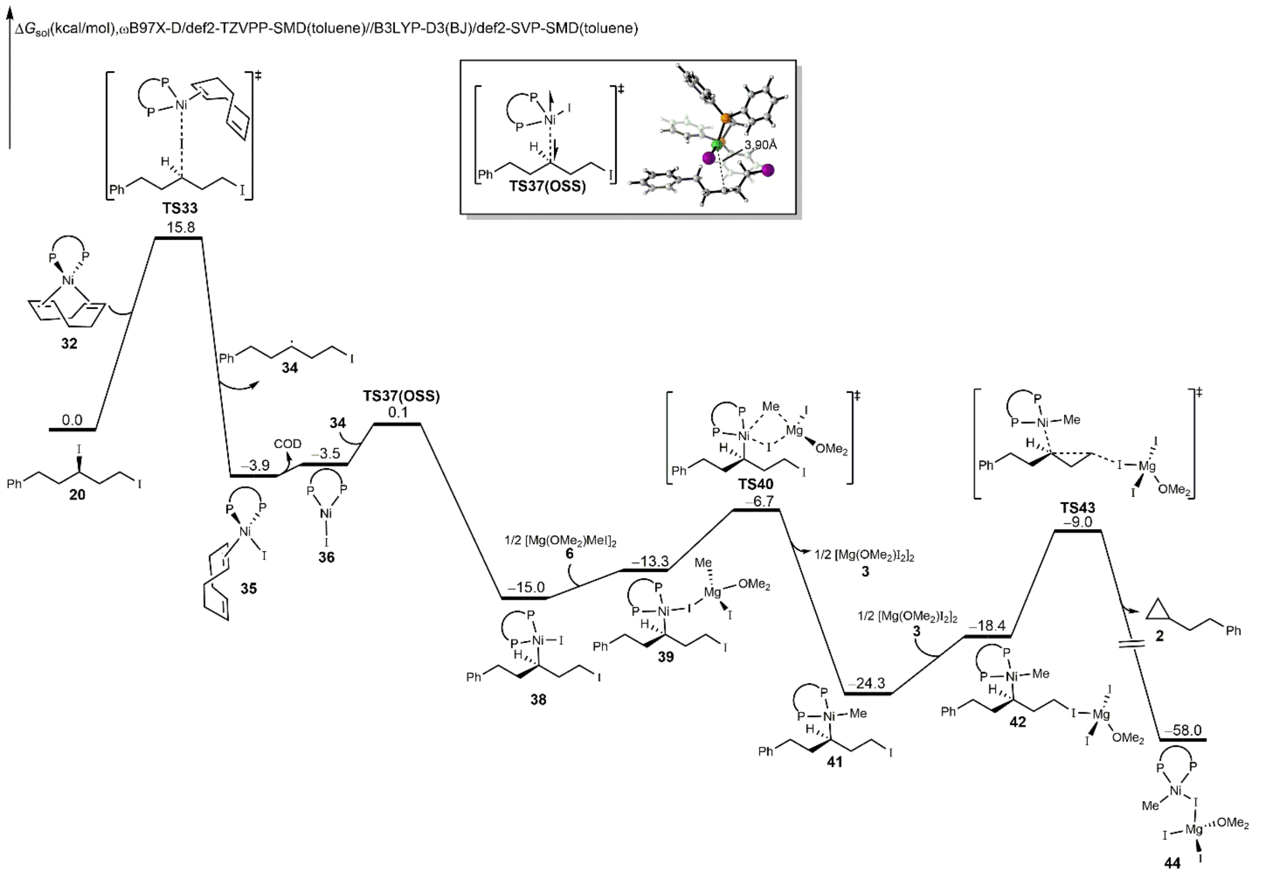

Figure SI-6. DFT-computed free energy changes of Ni/dppm-catalyzed XEC with 1,3-diiodide **20** (product formation) by using computational Method II.

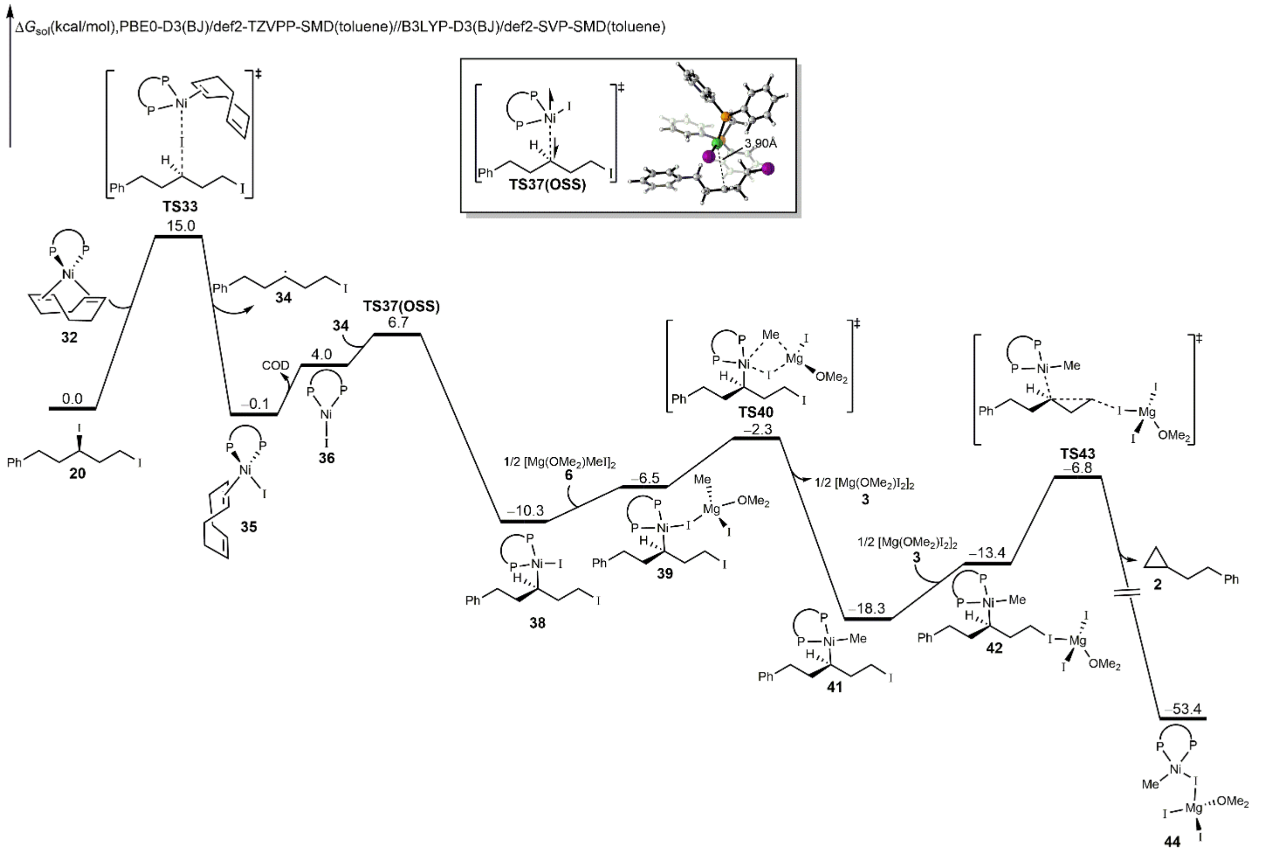

Figure SI-7. DFT-computed free energy changes of Ni/dppm-catalyzed XEC with 1,3-diiodide **20** (product formation) by using computational Method III.

l). Reaction Profile of Catalyst Regeneration

Completion of the catalytic cycle requires reduction of (dppm)Ni(Me)I **44** to regenerate the nickel(0) catalyst. The free energy changes of the catalyst regeneration are shown in Figure SI-8. From **44**, transmetallation with MeMgOMs occurs via **TS-S19** to generate (dppm)NiMe$_2$ (**S20**). Subsequent reductive elimination via **TS-S21** produces (dppm)Ni(ethane) (**S22**), which then releases ethane to regenerate the active nickel(0) catalyst (**32**) for the next catalytic cycle.

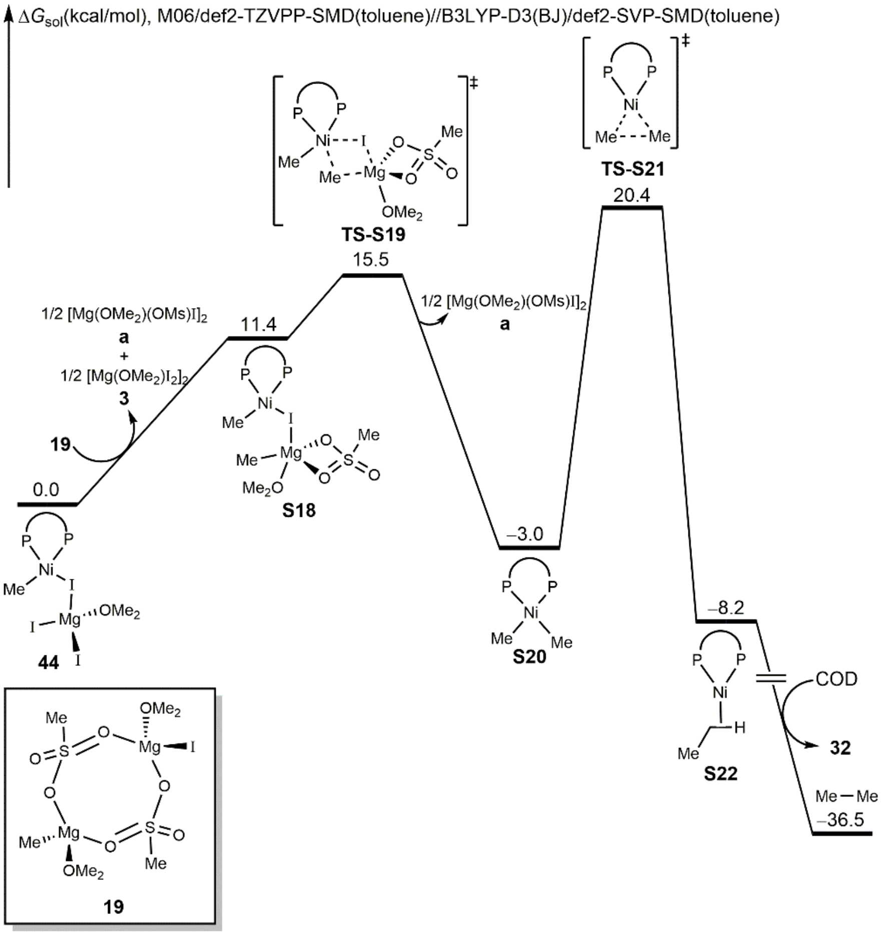

Figure SI-8. DFT-computed free energy changes of catalyst regeneration.

m). Table of Energies

Table SI-1. Energies in Scheme 3, Figure 1, Figure 2, Figure 3, Scheme SI-8, Figure SI-1, Figure SI-2, Scheme SI-9, Scheme SI-10, Figure SI-3, Scheme SI-11, Figure SI-4 and Figure SI-8. Zero-point vibrational energy (*ZPVE*), thermal correction to enthalpy (*TCH*), thermal correction to Gibbs free energy (*TCG*), energies (*E*), enthalpies (*H*), and Gibbs free energies (*G*) (in Hartree) of the structures calculated at the M06/def2-TZVPP-SMD(toluene)//B3LYP-D3(BJ)/def2-SVP-SMD(toluene) level of theory.

Structures	*ZPVE*	*TCH*	*TCG*	*E*	*H*	*G*	Imaginary Frequency
1	0.328519	0.351754	0.272931	-1754.933987	-1754.582233	-1754.661056	
3	0.168089	0.191203	0.107179	-1901.603920	-1901.412717	-1901.496741	
4	0.414458	0.448817	0.344395	-2705.750753	-2705.301936	-2705.406358	
5	0.414139	0.448790	0.341578	-2705.748117	-2705.299327	-2705.406539	
6	0.234696	0.259206	0.177132	-1385.682050	-1385.422844	-1385.504918	
7	0.447621	0.482774	0.376988	-2447.784808	-2447.302034	-2447.407820	
8	0.447705	0.482802	0.377263	-2447.783506	-2447.300704	-2447.406243	
9	0.531839	0.579208	0.442272	-3398.612044	-3398.032836	-3398.169772	

**TS10**	0.529554	0.576459	0.441742	-3398.575168	-3397.998709	-3398.133426	326.5*i*
**TS11**	0.530401	0.576815	0.444456	-3398.571601	-3397.994786	-3398.127145	326.6*i*
**TS12**	0.529592	0.575975	0.445637	-3398.587271	-3398.011296	-3398.141634	248.7*i*
**TS13**	0.529489	0.575843	0.444669	-3398.578346	-3398.002503	-3398.133677	216.5*i*
**14**	0.532822	0.579305	0.447512	-3398.615255	-3398.035950	-3398.167743	
**15**	0.530996	0.578090	0.444864	-3398.610569	-3398.032479	-3398.165705	
**16**	0.530781	0.578092	0.442279	-3398.636764	-3398.058672	-3398.194485	
**17**	0.649674	0.709130	0.546716	-4091.488724	-4090.779594	-4090.942008	
**TS18**	0.646416	0.705788	0.543913	-4091.468009	-4090.762221	-4090.924096	294.5*i*
**19**	0.305483	0.337373	0.239651	-2375.667758	-2375.330385	-2375.428107	
**20**	0.221978	0.236968	0.174751	-1022.974067	-1022.737099	-1022.799316	
**32**	0.577021	0.611425	0.509451	-3468.471709	-3467.860284	-3467.962258	
**TS33**	0.797662	0.848847	0.701312	-4491.432039	-4490.583192	-4490.730727	272.5*i*
**34**	0.217483	0.231370	0.172275	-725.138494	-724.907124	-724.966219	
**35**	0.578066	0.615835	0.502108	-3766.290647	-3765.674812	-3765.788539	
**COD**	0.179883	0.188225	0.148454	-311.898055	-311.709830	-311.749601	
**36**	0.396718	0.425696	0.329536	-3454.375201	-3453.949505	-3454.045665	
**TS37**	0.616059	0.658721	0.530982	-4179.530995	-4178.872274	-4179.000013	41.2*i*
**38**	0.619635	0.661743	0.535843	-4179.563021	-4178.901278	-4179.027178	
**39**	0.739150	0.793248	0.642721	-4872.414056	-4871.620808	-4871.771335	
**TS40**	0.739150	0.792436	0.642651	-4872.402896	-4871.610460	-4871.760245	55.5*i*
**41**	0.653217	0.695931	0.568152	-3921.613952	-3920.918021	-3921.045800	
**42**	0.738317	0.792665	0.639585	-4872.420581	-4871.627916	-4871.780996	
**TS43**	0.737270	0.791443	0.638463	-4872.404848	-4871.613405	-4871.766385	228.3*i*
**2**	0.219231	0.230320	0.182166	-427.399902	-427.169582	-427.217736	
**44**	0.518167	0.561099	0.433000	-4445.065691	-4444.504592	-4444.632691	
**TS45**	0.217620	0.230828	0.173980	-725.120128	-724.889300	-724.946148	526.1*i*
**I•**	0.000000	0.002360	-0.017503	-297.742134	-297.739774	-297.759637	
**TS46**	0.649373	0.692452	0.566832	-3921.565008	-3920.872556	-3920.998176	45.5*i*
**47**	0.429726	0.459300	0.365090	-3196.407302	-3195.948002	-3196.042212	
**S1**	0.498399	0.545164	0.407884	-3656.575078	-3656.029914	-3656.167194	
**S2**	0.371233	0.400354	0.305410	-2042.596109	-2042.195755	-2042.290699	
**Me_2O**	0.079149	0.084403	0.053781	-154.987373	-154.902970	-154.933592	
**TS-S3**	0.568908	0.615909	0.479931	-2890.418114	-2889.802205	-2889.938183	317.9*i*
**S4**	0.369499	0.399218	0.301083	-2042.599779	-2042.200561	-2042.298696	
**TS-S5**	0.566582	0.614621	0.474820	-2890.427237	-2889.812616	-2889.952417	205.4*i*
**S6**	0.499111	0.545128	0.410976	-3656.576921	-3656.031793	-3656.165945	
**TS-S7**	0.496169	0.541972	0.409146	-3656.547192	-3656.005220	-3656.138046	229.9*i*
**S8**	0.497770	0.544081	0.410153	-3656.567515	-3655.247614	-3655.381542	
**S9**	0.497151	0.544034	0.405331	-3656.597955	-3656.053921	-3656.192624	
**S10**	0.582794	0.641013	0.476595	-4607.413580	-4606.772567	-4606.936985	

**TS-S11**	0.580028	0.638009	0.475153	-4607.392555	-4606.754546	-4606.917402	286.9*i*
**S12**	0.272291	0.303502	0.203931	-2633.629759	-2633.326257	-2633.425828	
**TS-S13**	0.504445	0.549714	0.412739	-2821.602669	-2821.052955	-2821.189930	181.2*i*
**TS-S14**	0.505027	0.550171	0.413717	-2821.595918	-2821.045747	-2821.182201	248.2*i*
**TS-S15**	0.422094	0.461926	0.337269	-2666.597824	-2666.135898	-2666.260555	186.0*i*
**TS-S16**	0.422939	0.462514	0.337213	-2666.591737	-2666.129223	-2666.254524	248.2*i*
**38 (triplet)**	0.617672	0.660435	0.532307	-4179.540455	-4178.880020	-4179.008148	
**41 (triplet)**	0.651448	0.694490	0.567012	-3921.580460	-3920.885970	-3921.013448	
**TS-S17**	0.216638	0.230032	0.172966	-725.094514	-724.864482	-724.921548	479.4*i*
**34**[*]	0.219571	0.233233	0.174463	-725.309934	-725.076701	-725.135471	
**35**[*]	0.581629	0.618362	0.509583	-3766.120475	-3765.502113	-3765.610892	
**36**[*]	0.398914	0.427126	0.336008	-3454.185018	-3453.757892	-3453.849010	
**a**	0.272319	0.303509	0.204211	-2633.629751	-2633.326242	-2633.425540	
**39**[*]	0.789989	0.848609	0.687117	-5238.413402	-5237.564793	-5237.726285	
**TS40**[*]	0.791367	0.848466	0.692262	-5238.398429	-5237.549963	-5237.706167	53.3*i*
**S18**	0.603089	0.650771	0.516063	-4553.097508	-4552.446737	-4552.581445	
**TS-S19**	0.603588	0.650158	0.519854	-4553.094847	-4552.444689	-4552.574993	45.7*i*
**S20**	0.467516	0.498735	0.403052	-3236.298143	-3235.799408	-3235.895091	
**TS-S21**	0.466354	0.496961	0.402885	-3236.260763	-3235.763802	-3235.857878	560.5*i*
**S22**	0.470185	0.501239	0.403400	-3236.306772	-3235.805533	-3235.903372	
**Me-Me**	0.074130	0.078546	0.052716	-79.788576	-79.710030	-79.735860	

Table SI-2. Energies in Figure SI-6. Zero-point vibrational energy (*ZPVE*), thermal correction to enthalpy (*TCH*), thermal correction to Gibbs free energy (*TCG*), energies (*E*), enthalpies (*H*), and Gibbs free energies (*G*) (in Hartree) of the structures calculated at the ωB97X-D/def2-TZVPP-SMD(toluene)//B3LYP-D3(BJ)/def2-SVP-SMD(toluene) level of theory.

Structures	*ZPVE*	*TCH*	*TCG*	*E*	*H*	*G*	Imaginary Frequency
**20**	0.221978	0.236968	0.174751	-1023.215345	-1022.978377	-1023.040594	
**32**	0.577021	0.611425	0.509451	-3469.179377	-3468.567952	-3468.669926	
**TS33**	0.797662	0.848847	0.701312	-4492.379761	-4491.530914	-4491.678449	272.5*i*
**34**	0.217483	0.231370	0.172275	-725.362889	-725.131519	-725.190614	
**35**	0.578066	0.615835	0.502108	-3767.028201	-3766.412366	-3766.526093	
**COD**	0.179883	0.188225	0.148454	-312.056419	-311.868194	-311.907965	
**36**	0.396718	0.425696	0.329536	-3454.953884	-3454.528188	-3454.624348	
**TS37**	0.616059	0.658721	0.530982	-4180.333379	-4179.674658	-4179.802397	41.2*i*
**38**	0.619635	0.661743	0.535843	-4180.362339	-4179.700596	-4179.826496	
**6**	0.234696	0.259206	0.177132	-1385.889646	-1385.630440	-1385.712514	
**39**	0.739150	0.793248	0.642721	-4873.319236	-4872.525988	-4872.676515	
**TS40**	0.739150	0.792436	0.642651	-4873.308637	-4872.516201	-4872.665986	55.5*i*
**3**	0.168089	0.191203	0.107179	-1901.809718	-1901.618515	-1901.702539	
**41**	0.653217	0.695931	0.568152	-3922.414372	-3921.718441	-3921.846220	

**42**	0.738317	0.792665	0.639585	-4873.324222	-4872.531557	-4872.684637	
**TS43**	0.737270	0.791443	0.638463	-4873.308250	-4872.516807	-4872.669787	228.3*i*
**2**	0.219231	0.230320	0.182166	-427.604247	-427.373927	-427.422081	
**44**	0.518167	0.561099	0.433000	-4445.765648	-4445.204549	-4445.332648	

Table SI-3. Energies in Figure SI-7. Zero-point vibrational energy (*ZPVE*), thermal correction to enthalpy (*TCH*), thermal correction to Gibbs free energy (*TCG*), energies (*E*), enthalpies (*H*), and Gibbs free energies (*G*) (in Hartree) of the structures calculated at the PBE0-D3(BJ)/def2-TZVPP-SMD(toluene)//B3LYP-D3(BJ)/def2-SVP-SMD(toluene) level of theory.

Structures	*ZPVE*	*TCH*	*TCG*	*E*	*H*	*G*	Imaginary Frequency
**20**	0.221978	0.236968	0.174751	-1022.733298	-1022.496330	-1022.558547	
**32**	0.577021	0.611425	0.509451	-3467.632662	-3467.021237	-3467.123211	
**TS33**	0.797662	0.848847	0.701312	-4490.352295	-4489.503448	-4489.650983	272.5*i*
**34**	0.217483	0.231370	0.172275	-724.942206	-724.710836	-724.769931	
**35**	0.578066	0.615835	0.502108	-3765.414170	-3764.798335	-3764.912062	
**COD**	0.179883	0.188225	0.148454	-311.784010	-311.595785	-311.635556	
**36**	0.396718	0.425696	0.329536	-3453.606225	-3453.180529	-3453.276689	
**TS37**	0.616059	0.658721	0.530982	-4178.566566	-4177.907845	-4178.035584	41.2*i*
**38**	0.619635	0.661743	0.535843	-4178.598381	-4177.936638	-4178.062538	
**6**	0.234696	0.259206	0.177132	-1385.227625	-1384.968419	-1385.050493	
**39**	0.739150	0.793248	0.642721	-4871.221149	-4870.427901	-4870.578428	
**TS40**	0.739150	0.792436	0.642651	-4871.214415	-4870.421979	-4870.571764	55.5*i*
**3**	0.168089	0.191203	0.107179	-1901.106825	-1900.915622	-1900.999646	
**41**	0.653217	0.695931	0.568152	-3920.668893	-3919.972962	-3920.100741	
**42**	0.738317	0.792665	0.639585	-4871.229024	-4870.436359	-4870.589439	
**TS43**	0.737270	0.791443	0.638463	-4871.217376	-4870.425933	-4870.578913	228.3*i*
**2**	0.219231	0.230320	0.182166	-427.242989	-427.012669	-427.060823	
**44**	0.518167	0.561099	0.433000	-4444.032183	-4443.471084	-4443.599183	

Table SI-4. Energies in Figure SI-1 and Figure SI-5. Gas-phase energies (*E*) (in Hartree) of the structures calculated at M06/def2-TZVPP//B3LYP-D3(BJ)/def2-SVP-SMD(toluene) level of theory.

Structures	*E*
**MeMg(OMe)₂I**	-847.828347
**S2**	-2042.572593
**TS-S3**	-2890.389580
**MeMg(OMe)₂I fragment in TS-S3**	-847.823926
**S2 fragment in TS-S3**	-2042.508651
**S4**	-2042.576744
**TS-S5**	-2890.398656
**MeMg(OMe)₂I fragment in TS-S5**	-847.823408
**S4 fragment in TS-S5**	-2042.524444

**38**	-4179.526198
**(dppm)NiI fragment in 38**	-3454.333638
**2º alkyl radical fragment in 38**	-725.110180
**TS37**	-4179.49467
**(dppm)NiI fragment in TS37**	-3454.343907
**2º alkyl radical fragment in TS37**	-725.124494
**41**	-3921.57882
**(dppm)Ni(Me) fragment in 41**	-3196.372262
**2º alkyl radical fragment in 41**	-725.109203
**TS46**	-3921.530275
**(dppm)Ni(Me) fragment in TS46**	-3196.377582
**2º alkyl radical fragment in TS46**	-725.123729

Table SI-5. Energies in Scheme SI-12. Energies (*E*) (in Hartree) of the structures calculated at M06/def2-TZVPP//B3LYP-D3(BJ)/def2-SVP level of theory.

Structures	*ZPVE*	*TCH*	*TCG*	*E*	*H*	*G*	Imaginary Frequency
**38**	0.619224	0.661468	0.534241	-4179.526162	-4178.864694	-4178.991921	
**34**	0.217568	0.231440	0.172320	-725.126325	-724.894885	-724.954005	
**36**	0.396601	0.425476	0.329789	-3454.346709	-3453.921233	-3454.016920	
**41**	0.654232	0.696420	0.572258	-3921.578492	-3920.882072	-3921.006234	
**47**	0.429901	0.459410	0.365375	-3196.379816	-3195.920406	-3196.014441	

VII. References for Supporting Information

[1] Zheng, W.; Cole, P. A. Novel Bisubstrate Analog Inhibitors of Serotonin *N*-Acetyltransferase: The Importance of Being Neutral. *Bioorg. Chem.* **2003**, *31*, 398–411.
[2] Terai, T.; Kohno, M.; Boncompain, G.; Sugiyama, S.; Saito, N.; Fujikake, R.; Ueno, T.; Komatsu, T.; Hanaoka, K.; Okabe, T.; Urano, Y.; Perez, F.; Nagano, T. Artificial Ligands of Streptavidin (ALiS): Discovery, Characterization, and Application for Reversible Control of Intracellular Protein Transport. *J. Am. Chem. Soc.* **2015**, *137*, 33, 10464–10467.
[3] Walton, J. G. A.; Chankeshwara, S. V.; Bradley, M. Preparation of Imidazolidine and Thiazolidine Derivatives as Ubiquitination Modulators. WO 2011/135303, 2011.
[4] Halle, M. B.; Fernandes, R. A. A Relay Ring-Opening/Double Ring-Closing Metathesis Strategy for the Bicyclic Macrolide-Butenolide Core Structures. *RSC. Adv.* **2014**, *4*, 63342–63348.
[5] Stanley, L. M.; Hartwig, J. F. Regio- and Enantioselective *N*-Allylations of Imidazole, Benzimidazole, and Purine Heterocycles Catalyzed by Single-Component Metallacyclic Iridium Complexes. *J. Am. Chem. Soc.* **2009**, *131*, 8971–8983.
[6] Krasovskiy, A.; Knochel, P. Convenient Titration Method for Organometallic Zinc, Magnesium, and Lanthanide-Reagents. *Synthesis* **2006**, *5*, 890–891.
[7] Arlt, A.; Benson, S.; Schulthoff, S.; Gabor, B.; Fürstner, A. A Total Synthesis of Spirastrellolide A Methyl Ester. *Chem. Eur. J.* **2013**, *19*, 3596–3608.
[8] Hashimoto, T.; Naganawa, Y.; Maruoka, K. Stereoselective Construction of Seven-Membered Rings with an All-Carbon Quaternary Center by Direct Tiffeneau−Demjanov-type Ring Expansion. *J. Am. Chem. Soc.* **2009**, *131*, 6614–6617.

[9] Sanford, A. B.; Thane, T. A.; McGinnis, T. M.; Chen, P.–P.; Hong, X.; Jarvo, E. R. Nickel-Catalyzed Alkyl-Alkyl Cross-Electrophile Coupling Reaction of 1,3-Dimesylates for the Synthesis of Alkylcyclopropanes. *J. Am. Chem. Soc.* **2020**, *142*, 11, 5017–5023

[10] Azua, A.; Mata, J. A.; Heymes, P.; Peris, E.; Lamaty, F.; Martinez, J.; Colacino, E. Palladium N-Heterocyclic Carbene Catalysts for the Ultrasound-Promoted Suzuki–Miyaura Reaction in Glycerol. *Adv. Synth. Catal*. **2013**, *355*, 1107–1116.

[11] Mao, J.; Zhang, S.-Q.; Shi, B.-F.; Bao, W. Palladium(0)-Catalyzed Cyclopropanation of Benzyl Bromides via C(sp3)–H Bond Activation. *Chem. Commun*. **2014**, *50*, 3692–3694.

[12] Rao, M. L. N.; Meka, S. Pd-Catalyzed Atom-Efficient Cross-Coupling of Triarylbismuth Reagents with Protecting Group-Free Iodophenylmethanols: Synthesis of Biarylmethanols. *Tetrahedron Lett*. **2020**, *61*, 151676.

[13] Abecassis, K.; Gibson, S. E. Synthesis of (+)- and (–)-Gossonorol and Cyclisation to Boivinianin B. *Eur. J. Org. Chem*. **2010**, 2938–2944.

[14] Neufeld, K.; Henssen, B.; Pietruszka, J. Enantioselective Allylic Hydroxylation of ω-Alkenoic Acids and Esters by P450 BM3 Monooxygenase. *Angew. Chem. Int. Ed*. **2014**, *53*, 13253–13257.

[15] Kawasaki, M.; Shimizu, M.; Kuroyanagi, S.; Shishido, Y.; Komiyama, T.; Toyooka, N. Syntheses and odor properties of optically active dimethyl octenone and its analogs. *Tetrahedron Asymmetry* **2016**, *27*, 285–293.

[16] Brücher, O.; Bergsträßer, U.; Kelm, H.; Hartung, J.; Grab, M.; Svoboda, I.; Fuess, H. Controlling 6-endo-selectivity in oxidation/bromocyclization cascades for synthesis of aplysiapyranoids and other 2,2,6,6-substituted tetrahydropyrans. *Tetrahedron* **2012**, *68*, 6968–6980.

[17] Hu, G.; Xu, J.; Li, P. Sulfur Mediated Allylic C–H Alkylation of Tri- and Disubstituted Olefins. *Org. Lett*. **2014**, *16*, 6036–6039.

[18] Wallace, G. A.; Heathcock, C. H. Further Studies of the Daphniphyllum Alkaloid Polycyclization Cascade. *J. Org. Chem*. **2001**, *66*, 450–454.

[19] Pracht, P.; Bohle, F.; Grimme, S. Automated Exploration of the Low-energy Chemical Space with Fast Quantum Chemical Methods. *Phys. Chem. Chem. Phys*. **2020**, *22*, 7169−7192.

[20] For early studies relate to distortion/interaction analysis, see: (a) Kitaura, K.; Morokuma, K. A New Energy Decomposition Scheme for Molecular Interactions within the Hartree-Fock Approximation. *Int. J. Quantum Chem*. **1976**, *10*, 325−340. (b) Ziegler, T.; Rauk, A. A Theoretical Study of the Ethylene-Metal Bond in Complexes between Copper(1+), Silver(1+), Gold(1+), Platinum(0) or Platinum(2+) and Ethylene, Based on the Hartree-Fock-Slater Transition-State Method. *Inorg. Chem*. **1979**, *18*, 1558−1565.

[21] For reviews of distortion/interaction analysis, see: (a) van Leeuwen, P. W. N. M.; Kamer, P. C. J.; Reek, J. N. H.; Dierkes, P. Ligand Bite Angle Effects in Metal-catalyzed C−C Bond Formation. *Chem. Rev*. **2000**, *100*, 2741−2770. (b) Van Zeist, W.-J.; Bickelhaupt, F. M. The Activation Strain Model of Chemical Reactivity. *Org. Biomol. Chem*. **2010**, *8*, 3118−3127. (c) Fernandez, I.; Bickelhaupt, F. M. The Activation Strain Model and Molecular Orbital Theory: Understanding and Designing Chemical Reactions. *Chem. Soc. Rev*. **2014**, *43*, 4953−4967. (d) Bickelhaupt, F. M.; Houk, K. N. Analyzing Reaction Rates with the Distortion/Interaction-Activation Strain Model. *Angew. Chem., Int. Ed*. **2017**, *56*, 10070−10086.

[22] For discussions regarding S_N1–S_N2 mechanisms, see: (a) Phan, T. B.; Nolte, C.; Kobayashi, S.; Ofial, A. R.; Mayr, H. Can One Predict Changes from S_N1 to S_N2 Mechanisms? *J. Am. Chem. Soc*. **2009**, *131*, 11392–11401. (b) Vitullo, V. P.; Grabowski, J.; Sridharan, S. -Deuterium Isotope Effects in Benzyl Halides. 2. Reaction of Nucleophiles with Substituted Benzyl Bromides. Evidence for a Change in Transition-State Structure with Electron-Donating Substituents. *J. Am. Chem. Soc*. **1980**, *102*, 6463–6465. (c) Ko, E. C. F.; Parker, A. J. Solvation of Ions. XV. Solvation of "Tight" and "Loose" Transition States for S_N2, E_2C, and E_2Hal Reactions of Thiophenoxide Ion in Ethanol, Methanol, and Dimethylformamide. *J. Am. Chem. Soc*. **1968**, *90*, 6447–6453.

[23] Kehoe, R.; Mahadevan, M.; Manzoor, A.; McMurray, G.; Wienefeld, P.; Baird, M. C.; Budzelaar, P. H. M. Reactions of the Ni(0) Compound $Ni(PPh_3)_4$ with Unactivated Alkyl Halides: Oxidative Addition Reactions Involving Radical Processes and Nickel(I) Intermediates. *Organometallics*. **2018**, *37*, 2450−2467.

[24] For related mechanisms for oxidative addition of aryl halides, see: (a) Tsou, T. T.; Kochi, J. K. Mechanism of Oxidative Addition. Reaction of Nickel(0) Complexes with Aromatic Halides. *J. Am. Chem. Soc*. **1979**, *101*, 6319−6332. (b) Funes-Ardoiz, I.; Nelson, D. J.; Maseras, F. Halide Abstraction Competes with Oxidative Addition in the Reactions of Aryl Halides with $[Ni(PMe_nPh_{(3-n)})_4]$. *Chem. - Eur. J*. **2017**, *23*, 16728−16733.

[25] Sanford, A. B.; Thane, T. A.; McGinnis, T. M.; Chen, P.-P.; Hong, X.; Jarvo, E. R. Nickel-Catalyzed Alkyl–Alkyl Cross-Electrophile Coupling Reaction of 1,3-Dimesylates for the Synthesis of Alkylcyclopropanes. *J. Am. Chem. Soc*. **2020**, *142*, 5017−5023.

[26] (a) Zhu, C.; Yue, H.; Maity, B.; Atodiresei, I.; Cavallo, L.; Rueping, M. A Multicomponent Synthesis of Stereodefined Olefins via Nickel Catalysis and Single Electron/Triplet Energy Transfer. *Nature Catalysis*. **2019**, *2*,

678–687. (b) Maity, B.; Zhu, C.; Yue, H.; Huang, L.; Harb, M.; Minenkov, Y.; Rueping, M.; Cavallo, L. Mechanistic Insight into the Photoredox-Nickel-HAT Triple Catalyzed Arylation and Alkylation of α-Amino C_{sp3}–H Bonds. *J. Am. Chem. Soc*. **2020**, *142*, 16942−16952.
[27] Qi, Z.-H.; Ma, J. Dual Role of a Photocatalyst: Generation of Ni(0) Catalyst and Promotion of Catalytic C–N Bond Formation. *ACS Catal*. **2018**, *8*, 1456−1463.
[28] Chai, J.-D.; Head-Gordon, M. Long-Range Corrected Hybrid Density Functionals with Damped Atom−Atom Dispersion Corrections. *Phys. Chem. Chem. Phys*. **2008**, *10*, 6615−6620.
[29] Adamo, C.; Barone, V. Toward Reliable Density Functional Methods without Adjustable Parameters: The PBE0 Model. *J. Chem. Phys*. **1999**, *110*, 6158.

VIII. Cartesian Coordinates of Calculated Structures

1

C	6.27225400	-1.37149500	-0.87133600
C	5.00260900	-0.84748900	-1.12799300
C	4.26231900	-0.22216300	-0.11319100
C	4.82695200	-0.13722000	1.16893700
C	6.09567700	-0.66034400	1.43049900
C	6.82297400	-1.28012700	0.41010200
H	6.83523200	-1.85062500	-1.67644400
H	4.57913200	-0.92015800	-2.13400200
H	4.26430700	0.34899100	1.97132700
H	6.51992200	-0.58092000	2.43472700
H	7.81680900	-1.68712600	0.61207400
C	2.87084500	0.30036100	-0.37832500
C	1.78484300	-0.74414800	-0.08660000
H	2.78647900	0.62252400	-1.42983200
H	2.68364200	1.18770300	0.24530800
C	0.37678800	-0.26115700	-0.40116000
H	1.96890100	-1.64742200	-0.69054500
H	1.83630400	-1.05583300	0.97014500
C	-0.70580800	-1.28305500	-0.06960000
H	0.31738300	0.04366900	-1.45803000
H	-0.36819800	-2.28457300	-0.37863800
H	-0.83315600	-1.30636000	1.02418900
C	-2.04662200	-0.97909800	-0.71981400
H	-2.03621900	-1.20332500	-1.79789700
H	-2.31972000	0.07906900	-0.61870900
O	-3.04368900	-1.80134600	-0.08262700
S	-4.54164300	-1.15771600	0.10116400
O	-5.38307900	-2.28262100	0.47998300
O	-4.85281700	-0.34123100	-1.07064100
C	-4.30359700	-0.08650000	1.51260400
H	-3.54789500	0.67565700	1.27524400
H	-5.27477300	0.38939200	1.70840600
H	-3.99989500	-0.71027900	2.36304900
O	0.16310100	0.93552300	0.41334300
S	-0.74915600	2.17170900	-0.13727400
O	-1.96183300	2.23470300	0.67909100
O	-0.86167300	2.07521900	-1.59310400
C	0.31262300	3.52848000	0.30872400
H	0.50724400	3.47589700	1.38754100
H	1.23950000	3.45306600	-0.27348300
H	-0.23343500	4.44849900	0.05787500

3

Mg	1.71757800	-0.01194700	-0.29244300
I	-0.00755200	2.11704400	-0.76073500
I	0.19925300	-1.54605400	1.53621200
O	1.37278600	-1.27830300	-1.86909500
C	0.12203400	-1.39061900	-2.55850700
H	0.30370800	-1.66320500	-3.61016600
H	-0.51443100	-2.15250400	-2.08056300
H	-0.37298000	-0.41344700	-2.52137800
C	2.16791500	-2.46876700	-1.95351200
H	2.38584500	-2.69270000	-3.00977000
H	3.10889500	-2.28099700	-1.41951100
H	1.63517100	-3.31667300	-1.49283400
Mg	-1.65205200	0.18346700	0.44112600
I	-3.67836100	-0.70178700	-1.03478500
I	4.30915300	0.26800200	0.13841900
O	-2.47472700	1.22114500	1.99137700
C	-3.31719800	2.33391600	1.66574800

H	-3.25381700	3.09072800	2.46338000
H	-2.94974200	2.76639000	0.72571700
H	-4.35890800	1.99948100	1.53728600
C	-2.79669800	0.60245300	3.24216000
H	-3.80928000	0.16836200	3.20456300
H	-2.05714800	-0.18854900	3.42221900
H	-2.73656700	1.34838800	4.05040900
**4**			
C	-5.49264600	-0.22515500	1.24032400
C	-4.56921300	0.80682100	1.05299800
C	-4.30979000	1.31779200	-0.22865300
C	-5.00620400	0.76805100	-1.31576400
C	-5.93428700	-0.26013700	-1.13252300
C	-6.17816400	-0.76468800	0.14800100
H	-5.67185900	-0.61367600	2.24600400
H	-4.03465700	1.20327400	1.91906300
H	-4.80204200	1.14122500	-2.32259300
H	-6.46071000	-0.67626300	-1.99534000
H	-6.89687600	-1.57476700	0.29427700
C	-3.27650100	2.39856400	-0.45903200
C	-2.00577700	2.21717800	0.38151000
H	-3.71234200	3.39127600	-0.24535500
H	-3.01484500	2.40070000	-1.52587800
C	-0.79177200	2.95847800	-0.15329700
H	-2.16511400	2.57518100	1.40992000
H	-1.75648600	1.15159200	0.48914300
C	0.43205200	2.87177000	0.74790300
H	-1.04793100	4.00937900	-0.35735900
H	0.14743500	3.24807200	1.74232700
H	0.69500900	1.81616600	0.88232400
C	1.62535200	3.64988900	0.20856500
H	1.54394000	4.72444100	0.42827300
H	1.71095000	3.52983900	-0.87888600
O	2.85667300	3.23772700	0.83167500
S	3.72032700	2.01415600	0.14543800
O	5.10744500	2.45126300	0.12043100
O	3.05007600	1.62789100	-1.10215000
C	3.53671300	0.72812600	1.36076400
H	2.47597300	0.48219100	1.49311600
H	4.08697500	-0.14379000	0.98067000
H	3.97474600	1.09292200	2.29868200
O	-0.43629800	2.50865600	-1.53151800
S	-0.31354800	0.96608000	-1.94952400
O	-1.58419300	0.45275600	-2.44948900
O	0.29062800	0.22681900	-0.80596200
C	0.88523100	1.08178100	-3.24326500
H	0.47813000	1.74159400	-4.02029600
H	1.81885800	1.45331200	-2.80138000
H	1.01232400	0.05644700	-3.61909500
Mg	0.33543700	-1.52239600	0.18511100
I	2.50299700	-2.76755800	-0.76719300
I	-0.18152100	-0.77108100	2.71651100
O	-1.34802900	-2.31627900	-0.64869700
C	-2.66149500	-1.95726800	-0.19347700
H	-3.08680400	-1.18461300	-0.84903100
H	-3.30753500	-2.84931300	-0.18878400
H	-2.56270500	-1.57314200	0.82850700
C	-1.33808600	-2.87676700	-1.96542400
H	-1.73637600	-2.14644300	-2.68712500
H	-0.29698400	-3.12417300	-2.21318900
H	-1.94592100	-3.79586900	-1.98389200
**5**			
C	-8.68790100	-0.33792900	0.16402700
C	-7.45757900	-0.67544800	0.73675500
C	-6.30143200	0.06110000	0.44295100
C	-6.40311000	1.14612300	-0.44412800
C	-7.63003300	1.48426600	-1.01844000
C	-8.77756600	0.74298600	-0.71650700
H	-9.57987100	-0.92044000	0.40926000
H	-7.39445100	-1.52097100	1.42804900
H	-5.50558400	1.72513100	-0.67738200
H	-7.69247200	2.33444400	-1.70281800
H	-9.73850400	1.01001400	-1.16349300
C	-4.95931400	-0.31345900	1.02427300
C	-4.08716500	-1.08562500	0.02501300

H	-5.09623800	-0.91944100	1.93480400
H	-4.42363400	0.59962800	1.31914300
C	-2.66866800	-1.34108400	0.51991400
H	-4.54545800	-2.06392300	-0.19454800
H	-4.04537600	-0.53955900	-0.93017500
C	-1.83100600	-2.15750600	-0.46680400
H	-2.70423200	-1.86596500	1.48729200
H	-2.31065200	-3.14208300	-0.58053900
H	-1.84954200	-1.67121800	-1.45117300
C	-0.39766700	-2.32109500	0.00385000
H	-0.31076100	-3.01853700	0.84734800
H	0.03972300	-1.36801700	0.31471400
O	0.43445600	-2.90765500	-1.04457100
S	1.44307400	-2.01381000	-1.91575300
O	2.48673100	-2.90761600	-2.38747800
O	1.84730300	-0.83878500	-1.10219400
C	0.47120400	-1.39255400	-3.26035700
H	-0.26717400	-0.69393800	-2.84520100
H	1.16983400	-0.86631300	-3.92607300
H	0.01521600	-2.25199700	-3.76871600
O	-1.99509300	-0.10071400	0.90886200
S	-1.70548200	1.11325300	-0.15206200
O	-2.95143700	1.80209500	-0.48783900
O	-0.88529200	0.59726100	-1.25646200
C	-0.72054300	2.11926200	0.92798200
H	-1.35810600	2.45528900	1.75530600
H	0.12967200	1.52862900	1.29360600
H	-0.37453500	2.96735700	0.32160800
Mg	2.95201700	0.17423700	0.22251300
I	2.00187200	-0.53463200	2.63654800
I	3.12084700	2.69008200	-0.65883300
O	4.63824900	-0.90266700	-0.21474900
C	5.26969200	-0.70200000	-1.48456700
H	6.35172100	-0.89034000	-1.39620300
H	4.83250200	-1.37787900	-2.23765400
H	5.10281800	0.34404200	-1.77504800
C	4.79984400	-2.23117800	0.29695500
H	5.87223700	-2.46022200	0.40358200
H	4.31592000	-2.26626900	1.28175000
H	4.32529500	-2.95874000	-0.38146000
**6**			
Mg	-1.66846800	0.09157700	0.88003200
I	-0.00000800	-2.05845000	-0.00006400
I	0.00001900	2.22184900	0.00001500
Mg	1.66848100	0.09156800	-0.88001200
C	2.65547600	0.05652100	-2.74225500
H	3.47040300	0.80382300	-2.80254000
H	3.10988400	-0.92682800	-2.97017800
H	1.97140600	0.28069500	-3.58280800
O	2.98821000	0.00061700	0.71360200
C	4.22503700	-0.68290600	0.50203400
H	5.03355500	-0.17835300	1.05604300
H	4.14694500	-1.73236300	0.83309700
H	4.44300400	-0.64975200	-0.57332300
C	2.60431200	0.07143400	2.08738000
H	1.67690100	0.65476300	2.14330100
H	2.43508800	-0.93903300	2.49453600
H	3.38971400	0.58235200	2.66809900
C	-2.65538600	0.05637300	2.74231100
H	-3.47030400	0.80367500	2.80270300
H	-3.10979300	-0.92699500	2.97014900
H	-1.97128000	0.28045200	3.58286100
O	-2.98825700	0.00068600	-0.71352800
C	-4.22505500	-0.68289300	-0.50195600
H	-4.44297500	-0.64984600	0.57341400
H	-5.03361600	-0.17831500	-1.05588000
H	-4.14694500	-1.73231500	-0.83312100
C	-2.60442800	0.07163400	-2.08731900
H	-1.67701900	0.65496700	-2.14323800
H	-2.43522500	-0.93879600	-2.49457700
H	-3.38986400	0.58260100	-2.66794700
**7**			
C	-6.45610700	-0.64973900	-1.48899700
C	-5.40830700	-1.54366400	-1.24626000
C	-4.41048300	-1.24214700	-0.30918300

C	-4.48223900	-0.02059300	0.38067100
C	-5.52447400	0.87568300	0.13727400
C	-6.51607700	0.56362800	-0.79894200
H	-7.22775500	-0.90297400	-2.22072600
H	-5.36622600	-2.49158800	-1.79079900
H	-3.71267400	0.22773300	1.11568100
H	-5.55926900	1.82215600	0.68223800
H	-7.33309500	1.26421700	-0.98884500
C	-3.24795100	-2.17382800	-0.06477900
C	-1.97128900	-1.70154900	-0.77825300
H	-3.49844700	-3.19216900	-0.40363500
H	-3.05526600	-2.23224500	1.01596300
C	-0.72291200	-2.47672400	-0.38821100
H	-2.09381700	-1.81053100	-1.86764200
H	-1.81068400	-0.62642400	-0.60869700
C	0.53828700	-2.09031600	-1.14749500
H	-0.91570100	-3.55545500	-0.49557600
H	0.31693500	-2.12899100	-2.22506100
H	0.80578900	-1.05133800	-0.91612800
C	1.70501500	-3.01026300	-0.82191100
H	1.60701200	-3.98858600	-1.31471700
H	1.77732500	-3.18316800	0.26021700
O	2.94721900	-2.46363000	-1.31440400
S	3.89623900	-1.62622800	-0.27421700
O	5.08503000	-2.41906200	0.00859100
O	3.06418100	-1.17561700	0.85481100
C	4.30967000	-0.24834400	-1.31195100
H	3.39227500	0.31384200	-1.54201600
H	5.01776400	0.36908200	-0.74192400
H	4.78537000	-0.64213000	-2.21954900
O	-0.46139200	-2.40963400	1.07727700
S	-0.43580300	-1.04123600	1.91722200
O	-1.70434100	-0.82758400	2.59961800
O	0.04405900	0.04473800	1.02017500
C	0.85168000	-1.41580800	3.07466100
H	0.54616300	-2.30778600	3.63713700
H	1.78233400	-1.56679500	2.51154300
H	0.92237800	-0.54279100	3.73886500
Mg	-0.24005300	2.01370900	0.54950700
I	1.09453600	2.06970500	-1.86060900
O	1.26200000	2.54668400	1.88789300
C	1.21614000	3.82630300	2.51472600
H	1.62902500	3.76346000	3.53568900
H	1.79002600	4.56629600	1.93091700
H	0.16283300	4.13155600	2.56477900
C	2.57280600	1.98809000	1.80538400
H	2.99494500	1.87357500	2.81863100
H	2.49662400	1.00410700	1.32841300
H	3.23139800	2.63772700	1.20470200
C	-2.07532700	2.90064300	1.10958500
H	-2.89270100	2.60741400	0.42485500
H	-2.39517700	2.59913400	2.12639800
H	-2.05877000	4.00782900	1.09975400
**8**			
C	-8.40674300	-0.36260200	0.66099700
C	-7.14551500	-0.41435200	1.26291600
C	-6.03697900	0.19940400	0.66303700
C	-6.21792100	0.86621500	-0.56060800
C	-7.47601800	0.91845000	-1.16390000
C	-8.57564200	0.30388600	-0.55516700
H	-9.26073400	-0.84252200	1.14621500
H	-7.02065100	-0.93454600	2.21717000
H	-5.35804700	1.34911100	-1.03232100
H	-7.60058300	1.44613000	-2.11316000
H	-9.56098500	0.34784300	-1.02597800
C	-4.66122100	0.11856700	1.27994800
C	-3.78671400	-0.95033400	0.60796400
H	-4.74135200	-0.10287100	2.35668000
H	-4.16190300	1.09298600	1.18392700
C	-2.33357900	-0.92732800	1.06318600
H	-4.19097300	-1.95247900	0.82510800
H	-3.82532700	-0.83924700	-0.48749400
C	-1.49823900	-2.08540000	0.51511100
H	-2.29478800	-0.93725800	2.16395100
H	-1.95146300	-3.02920400	0.85461200

H	-1.54593700	-2.07459800	-0.58117100
C	-0.05396900	-1.99275000	0.97905700
H	0.08272300	-2.35549600	2.00579400
H	0.31118600	-0.96104900	0.93990600
O	0.81639200	-2.83243200	0.16566300
S	1.80388000	-2.18838700	-0.92954600
O	2.90844000	-3.11903700	-1.08549000
O	2.10943200	-0.78498000	-0.52558500
C	0.83673300	-2.09132100	-2.41028200
H	0.02263000	-1.36886900	-2.25128000
H	1.51671400	-1.74569500	-3.20170100
H	0.46363600	-3.10043500	-2.62902800
O	-1.68606900	0.35631600	0.77418300
S	-1.70876400	1.03067500	-0.71858200
O	-2.88719800	1.88354800	-0.85266600
O	-1.49233100	-0.01466100	-1.73157600
C	-0.24670900	2.02385800	-0.55026600
H	-0.36346700	2.67783500	0.32259900
H	0.62277300	1.36516100	-0.44408900
H	-0.16919900	2.61727700	-1.47140000
Mg	3.36242500	-0.04584400	0.97686100
I	3.53603600	2.54972300	0.15369000
O	5.03455400	-0.96198700	0.16709200
C	5.44619600	-0.74196100	-1.18058900
H	6.54681000	-0.70689200	-1.23873100
H	5.06538900	-1.54635100	-1.83125300
H	5.03781300	0.22748200	-1.49586900
C	5.55379500	-2.16215200	0.73994700
H	6.65622200	-2.13148300	0.74309200
H	5.18477800	-2.22101900	1.77145800
H	5.20244100	-3.03913600	0.17335700
C	2.83955700	-1.01384900	2.78916000
H	1.91577700	-0.61268000	3.24922500
H	2.66948300	-2.09932300	2.64433800
H	3.61821100	-0.92858300	3.57213100
**9**			
C	6.91420700	1.06233300	-0.60521700
C	5.52036300	1.13274600	-0.56944400
C	4.87198000	2.26554000	-0.05093900
C	5.65498800	3.32292700	0.43315100
C	7.05116400	3.25606600	0.39784900
C	7.68514200	2.12469600	-0.12163500
H	7.40100100	0.17286400	-1.01321700
H	4.92463800	0.29564100	-0.94408100
H	5.16395700	4.21094300	0.84187500
H	7.64503600	4.09164400	0.77737500
H	8.77629800	2.07020200	-0.15058600
C	3.36433200	2.31092200	0.02598100
C	2.82232200	1.47039800	1.18771100
H	2.93507500	1.93085600	-0.91618900
H	3.03290500	3.35417200	0.13979600
C	1.30956300	1.35923600	1.22630400
H	3.21056100	0.44363000	1.10028300
H	3.18414300	1.86884400	2.14989300
C	0.82668100	0.39525800	2.29911600
H	0.92922900	1.03882100	0.24384400
H	1.33672600	-0.55904700	2.09010900
H	1.17373100	0.72681600	3.29030200
C	-0.67530500	0.16053800	2.30945800
H	-1.08820400	0.02494800	1.30465400
H	-1.23229200	0.96842300	2.79700100
O	-1.00456400	-1.01481900	3.10957600
S	-0.91128900	-2.48266100	2.45696500
O	0.05755200	-3.28396900	3.17885800
O	-0.71687400	-2.30886300	0.98598700
C	-2.55953800	-3.07745000	2.70251200
H	-3.24410700	-2.41126600	2.15733800
H	-2.58938100	-4.10023100	2.30216400
H	-2.75033700	-3.07436700	3.78375200
O	0.77206300	2.71650800	1.49728500
S	-0.41240300	3.34039500	0.62166100
O	-0.87732100	4.51706200	1.33140200
O	-1.39514200	2.24545200	0.38065100
C	0.30776500	3.77872300	-0.94084700
H	1.07930700	4.53551500	-0.75296600

H	0.71025500	2.87508200	-1.41614000
H	-0.51281300	4.18871900	-1.54645000
Mg	-2.86862200	1.80520700	-0.97303700
I	-3.98588100	-0.56177000	-0.00684300
O	-4.23842700	3.01911400	0.10332800
C	-5.51782500	3.12414700	-0.51614200
H	-5.99767900	4.07698300	-0.23594400
H	-5.36056200	3.10487900	-1.60286800
H	-6.16424200	2.27995800	-0.22145500
C	-4.29336900	3.07884100	1.52517400
H	-4.81219500	2.19548200	1.93458900
H	-3.26249400	3.10865000	1.89451600
H	-4.81497300	3.99750700	1.84219600
Mg	0.45765400	-2.21765600	-0.62529500
I	3.02286600	-2.55003300	0.05541000
I	-0.14376000	-0.09426700	-2.19174500
O	-0.40079000	-3.80348100	-1.55170500
C	-1.78096800	-3.72215200	-1.92703500
H	-2.29106600	-4.66483900	-1.66958500
H	-2.23838800	-2.89103300	-1.37290900
H	-1.87090800	-3.52834800	-3.00789400
C	0.33024100	-4.84251900	-2.20592500
H	1.35715200	-4.81941500	-1.81708800
H	-0.12710500	-5.82007300	-1.98211800
H	0.33996800	-4.67620900	-3.29578500
C	-2.74295700	2.84340000	-2.81974600
H	-3.48451300	2.49426700	-3.56498900
H	-2.93243200	3.92911600	-2.69334900
H	-1.76122500	2.75026400	-3.32066700
**TS10**			
C	-4.67691800	4.84800800	-1.16040900
C	-3.92554400	3.68131600	-0.99064400
C	-2.66271600	3.72229500	-0.38195200
C	-2.16876400	4.96446000	0.04848200
C	-2.91581000	6.13228700	-0.11869100
C	-4.17491300	6.07717900	-0.72526600
H	-5.66042900	4.79501400	-1.63439400
H	-4.32147700	2.72039100	-1.33006700
H	-1.18224700	5.02130900	0.51899100
H	-2.51474300	7.08904800	0.22557300
H	-4.76180400	6.98958700	-0.85701600
C	-1.84318800	2.46499400	-0.22383400
C	-0.62004100	2.43763400	-1.14992900
H	-2.47010000	1.58960900	-0.44345400
H	-1.52806200	2.34822400	0.82405600
C	0.22902000	1.18931500	-0.98745500
H	-0.96807100	2.44642400	-2.19518600
H	-0.00010200	3.33845300	-1.02691300
C	1.29848300	1.00613800	-2.05671600
H	-0.41841100	0.30580900	-0.98702800
H	0.80002700	0.99688000	-3.03737200
H	1.97688900	1.87061600	-2.05921400
C	2.08156700	-0.26216800	-1.91790700
H	2.20946200	-0.75591000	-0.95803900
H	2.52991900	-0.73164600	-2.78679300
O	0.39581500	-1.45069900	-2.30602000
S	0.31087700	-2.78085900	-1.59634900
O	-0.41922600	-2.62682600	-0.26520700
O	0.83455600	1.07040500	0.35990300
S	1.76023800	2.17668300	1.05677700
O	2.20265600	3.20308600	0.12418200
O	2.80303900	1.37705900	1.76306000
C	0.73896900	2.84903500	2.33892100
H	-0.02880100	3.47924500	1.87501600
H	0.28862400	2.01186200	2.89007200
H	1.40431800	3.44656200	2.97757200
I	4.69280800	0.61424000	-1.45502500
Mg	4.34425200	0.13852900	1.31026800
O	3.17967400	-1.56319800	1.06150500
C	2.04525300	-1.83708500	1.89543500
H	2.37384700	-2.21852000	2.87698800
H	1.49493300	-0.90040100	2.02675200
H	1.39172800	-2.56915200	1.40233800
C	3.92223700	-2.73774400	0.70537000
H	4.72841500	-2.42342200	0.02818800

H	4.35636300	-3.20135900	1.60691900
H	3.26278500	-3.44120300	0.17877300
Mg	-2.01019000	-1.80575100	0.52908600
I	-3.62255200	-1.02379500	-1.51430900
O	-2.99952100	-3.51001200	1.12321800
C	-4.30099900	-3.34233600	1.69621400
H	-4.28307900	-2.41763600	2.28827300
H	-5.06306400	-3.26345500	0.90396400
H	-4.52647700	-4.19364100	2.35832800
C	-2.85171800	-4.71484500	0.37294100
H	-1.79066200	-4.80801600	0.11242800
H	-3.15135900	-5.57751000	0.98952000
H	-3.46630400	-4.68005200	-0.54257800
I	-1.64108300	-0.43202000	2.81720100
C	5.90267200	-0.16301500	2.69310600
H	5.53383700	-0.63868600	3.62308300
H	6.69002500	-0.83097900	2.29352400
H	6.41518100	0.76695100	3.00381800
C	-0.76091800	-3.76694500	-2.62544500
H	-0.86829600	-4.75377000	-2.15628600
H	-1.72788500	-3.25152400	-2.71074400
H	-0.27334400	-3.85709400	-3.60507700
O	1.59745700	-3.46434000	-1.42606900
**TS11**			
C	5.84080500	4.36028300	0.24402800
C	4.93728900	3.29866100	0.14882800
C	3.64768800	3.49883300	-0.36725400
C	3.28626100	4.78916800	-0.78359300
C	4.18643700	5.85345600	-0.68978100
C	5.46827300	5.64131900	-0.17388400
H	6.84300000	4.18479200	0.64337400
H	5.23210400	2.29564600	0.46987500
H	2.28671100	4.96523000	-1.19272600
H	3.88763000	6.85058100	-1.02334900
H	6.17548300	6.47133400	-0.10148400
C	2.66393000	2.35626800	-0.42387400
C	1.76230100	2.30504600	0.81932100
H	3.20949400	1.40423700	-0.49675800
H	2.05436100	2.42973300	-1.33597400
C	0.81087000	1.12366200	0.82403000
H	2.40239200	2.18938900	1.70849100
H	1.21259700	3.24926000	0.96000400
C	0.09354700	0.87512600	2.14432900
H	1.36269900	0.21876000	0.54898400
H	0.86180500	0.75356900	2.92248100
H	-0.50059500	1.75453700	2.42984000
C	-0.75242600	-0.36199400	2.14299900
H	-1.10103700	-0.81726200	1.22117300
H	-0.94321200	-0.89971000	3.06548400
O	0.98723700	-1.60066600	1.98174900
S	0.80505700	-2.94896000	1.32619800
O	1.18715000	-2.88196900	-0.14611900
O	-0.17836000	1.17124400	-0.28592600
S	-1.19073900	2.37264600	-0.57691400
O	-1.31872400	3.29105400	0.54271000
O	-2.42236800	1.67024300	-1.05389000
C	-0.53526700	3.18085900	-2.01197600
H	0.36506100	3.73125000	-1.71411300
H	-0.31144100	2.41891800	-2.76993200
H	-1.31911100	3.87022500	-2.35575400
I	-3.32116800	0.52838700	2.40406600
Mg	-3.66484500	0.28283300	-0.32855100
O	-2.57791100	-1.41907200	-0.59908200
C	-1.65727600	-1.61428100	-1.68456800
H	-2.18609100	-2.02756800	-2.55873500
H	-1.22798100	-0.63943300	-1.93615600
H	-0.85471900	-2.29298800	-1.36427800
C	-3.17314800	-2.64310400	-0.13026600
H	-3.81017300	-2.38977400	0.72802500
H	-3.78870500	-3.08814000	-0.92797300
H	-2.37959200	-3.32795800	0.19867800
Mg	2.42971700	-1.79412900	-1.26982000
I	4.57772200	-1.22559100	0.40963000
O	3.28799700	-3.41720000	-2.25321700
C	4.25299700	-3.08589900	-3.25178500

H	3.90775600	-2.17172000	-3.75273600
H	5.23975500	-2.90509900	-2.79363200
H	4.32612300	-3.90141300	-3.99039700
C	3.58955500	-4.61745800	-1.54560200
H	2.75310600	-4.81186000	-0.86357900
H	3.68658800	-5.45708100	-2.25394400
H	4.52329700	-4.50577700	-0.96901700
C	2.02578300	-4.00317600	2.08762800
H	1.91641300	-5.00738100	1.65705200
H	3.02032300	-3.58150500	1.88516100
H	1.81609100	-4.02146800	3.16521300
O	-0.53071200	-3.53174100	1.52000900
I	-5.93873200	0.01120200	-1.65266000
C	1.64494100	-0.44573300	-2.71426000
H	1.08894000	-0.97601700	-3.51318600
H	0.94202500	0.27714900	-2.26662000
H	2.42696400	0.15088600	-3.22226000

TS12

C	-7.41011100	-1.80556900	-1.06799400
C	-6.26088600	-1.04720400	-0.82605700
C	-4.99167500	-1.64388100	-0.83967100
C	-4.89492000	-3.02092300	-1.09877900
C	-6.04265200	-3.77954800	-1.34133900
C	-7.30395100	-3.17505700	-1.32608900
H	-8.39143100	-1.32403800	-1.05836600
H	-6.34746700	0.02531200	-0.62868500
H	-3.90533400	-3.48476900	-1.11665100
H	-5.95195700	-4.84918600	-1.54849100
H	-8.20105500	-3.76890600	-1.51887700
C	-3.75219000	-0.83719900	-0.53658400
C	-3.37432600	-0.91932100	0.94587300
H	-3.90995800	0.21693700	-0.80667800
H	-2.91131900	-1.22228400	-1.12983100
C	-2.06594000	-0.32612100	1.32313900
H	-4.16351800	-0.49733700	1.59064300
H	-3.29760300	-1.97862900	1.24967000
C	-1.67967700	-0.22891300	2.76336600
H	-1.36081500	-0.00975500	0.56053400
H	-2.32530000	0.51606600	3.25445100
H	-1.93313400	-1.19577100	3.23052800
C	-0.21993300	0.04022600	3.06761700
H	0.43427500	-0.67982300	2.55983000
H	-0.03698100	-0.00973000	4.14800700
O	0.16140700	1.41187800	2.70019800
S	1.09815200	1.69405000	1.44153600
O	0.66831700	2.99934300	0.90226900
O	1.00020600	0.54047700	0.52934800
C	2.72077800	1.89759400	2.10127700
H	3.00920300	0.95268300	2.58422500
H	3.37273900	2.10992300	1.24008100
H	2.67596000	2.74132100	2.80272100
O	-0.89330900	-2.22039400	1.11785400
S	-0.44553200	-2.75331200	-0.21970900
O	0.50228600	-1.76997300	-0.89195600
O	-1.52555000	-3.14960200	-1.13472400
C	0.52160900	-4.20473800	0.16023000
H	1.35534400	-3.91600700	0.81467000
H	-0.14773100	-4.91673100	0.66092100
H	0.88588500	-4.62013000	-0.78867300
I	-2.92956500	2.47196400	0.86605300
Mg	-0.75523800	3.78612700	-0.36022500
Mg	2.23700300	-0.92483800	-0.45592500
I	3.23725900	-1.89511300	1.95475600
O	3.17574400	-2.36970800	-1.66621400
C	4.59773100	-2.50028800	-1.65053800
H	4.95416600	-2.18804300	-0.66115200
H	4.87596600	-3.55378400	-1.82122900
H	5.05086900	-1.86101300	-2.42561100
C	2.60036500	-2.77107700	-2.90836900
H	1.51409500	-2.65328400	-2.82538000
H	2.98186700	-2.14515400	-3.73330900
H	2.84510700	-3.82756700	-3.11156400
I	3.51988100	1.16001700	-1.76690900
O	-0.40519400	2.64504300	-2.03552500
C	0.30510900	3.24507400	-3.12631100

H	0.48617400	4.29585000	-2.86560100
H	1.26549200	2.72855000	-3.27643100
H	-0.30578200	3.19390300	-4.04284900
C	-0.69589200	1.26203300	-2.24288300
H	-1.27744000	0.91436400	-1.38394200
H	-1.30009200	1.13651400	-3.15642000
H	0.23606700	0.68475000	-2.32744100
C	-0.57464800	5.85391800	-0.71526900
H	-0.95542000	6.45724700	0.13080500
H	0.47931900	6.16063600	-0.86137800
H	-1.12877400	6.19606300	-1.61036000

TS13

C	-1.95180000	7.29811800	0.33703000
C	-2.04708800	5.90887400	0.21103400
C	-0.95309200	5.08553200	0.51274500
C	0.24379500	5.68087000	0.94425200
C	0.34007900	7.06867900	1.07015900
C	-0.75666900	7.88196300	0.76583400
H	-2.81486500	7.92612600	0.10114600
H	-2.98493200	5.45547700	-0.12318400
H	1.09349000	5.03701100	1.18678100
H	1.27593600	7.51892100	1.41163400
H	-0.68085500	8.96766700	0.86602200
C	-1.02680000	3.58906600	0.32326200
C	-0.48821000	3.18393500	-1.05069300
H	-2.06576600	3.24152200	0.42258400
H	-0.41774100	3.09434500	1.09212500
C	-0.25058800	1.74225200	-1.30100600
H	-1.09763000	3.60341400	-1.86890800
H	0.51306800	3.63207400	-1.19005000
C	0.12896900	1.31497300	-2.68193000
H	-0.22808000	1.02778100	-0.48405900
H	-0.74806000	1.41936600	-3.34005400
H	0.86082400	2.05464900	-3.05003600
C	0.76924400	-0.04622900	-2.84065800
H	1.64978200	-0.14664800	-2.19335900
H	1.06857200	-0.21313000	-3.88261300
O	-0.19318800	-1.11974100	-2.55434500
S	-0.01434000	-2.02236200	-1.25165400
O	-1.39249000	-2.42859800	-0.87202300
O	0.73636100	-1.26674000	-0.24344800
C	0.84309400	-3.46361300	-1.80017100
H	1.83760400	-3.13909400	-2.14450600
H	0.93630100	-4.12727000	-0.92932900
H	0.24004600	-3.91618800	-2.59833000
O	1.95227400	1.76399000	-0.68600100
S	2.42827300	1.72417200	0.74133600
O	2.21331800	0.35310800	1.36213100
O	1.87318000	2.77746900	1.61014600
C	4.19778800	1.94367600	0.63731700
H	4.61280500	1.13992400	0.01388000
H	4.37811600	2.92718400	0.18359500
H	4.60392600	1.90644000	1.65645400
I	-3.05081500	0.89345100	-1.24175600
Mg	-2.81532800	-1.43748200	0.20038700
Mg	2.66236000	-1.54897600	0.91507900
O	4.36688000	-1.29352800	2.22131800
C	5.34946900	-2.32284700	2.20941000
H	5.43904100	-2.68320600	1.17669600
H	6.32368700	-1.92191900	2.53880400
H	5.05389800	-3.15588900	2.87046200
C	4.17548500	-0.71855600	3.50650800
H	3.41142500	0.06189200	3.40933500
H	3.83473200	-1.47934600	4.23149300
H	5.11711800	-0.27242000	3.87160100
O	-1.64679800	-0.95562800	1.79635700
C	-1.12343700	-2.00707400	2.61983800
H	-1.51042700	-2.96024500	2.23685900
H	-0.02451600	-2.00720000	2.57978400
H	-1.46961900	-1.87191800	3.65698500
C	-1.20912900	0.34798100	2.20049900
H	-1.73723200	1.07943300	1.57952900
H	-1.47385500	0.51693900	3.25678300
H	-0.12094700	0.44351500	2.06688900
I	-4.74994800	-3.08061000	0.93526600

I	4.20438000	-1.54414600	-1.47507800
C	2.05434000	-3.46109500	1.67591800
H	2.09657700	-3.53817700	2.78162100
H	1.04278500	-3.81819200	1.39990500
H	2.75673900	-4.23684600	1.30690200

14

C	7.21436700	-1.38917700	0.95780500
C	5.89880500	-0.97411000	0.73429500
C	4.82788600	-1.86610500	0.89940700
C	5.10640500	-3.18484800	1.29118800
C	6.42082300	-3.60279000	1.51469500
C	7.47994500	-2.70524300	1.34800200
H	8.03609300	-0.67997400	0.82842800
H	5.69336200	0.05614800	0.43047300
H	4.27884000	-3.88758400	1.42471500
H	6.61939700	-4.63260500	1.82346700
H	8.50863600	-3.02978500	1.52431400
C	3.41165000	-1.43447900	0.60831400
C	3.03845400	-1.67390100	-0.86276300
H	3.30110800	-0.36037400	0.81962500
H	2.72070800	-1.98056600	1.26234600
C	1.60890600	-1.29263000	-1.23928200
H	3.71912600	-1.07930500	-1.48999600
H	3.19906200	-2.72854800	-1.13914500
C	1.46457700	-0.89981100	-2.70384500
H	1.22182800	-0.49606700	-0.59166200
H	2.20432900	-0.11316500	-2.91486800
H	1.72918200	-1.77230000	-3.32407400
C	0.09940200	-0.41168800	-3.14803900
H	-0.73115400	-0.98462700	-2.71157200
H	0.00978400	-0.44354600	-4.24078100
O	-0.06906000	1.02121500	-2.84162600
S	-0.93993800	1.53011600	-1.61410100
O	-0.34016700	2.80380300	-1.18921300
O	-0.99310900	0.44613100	-0.61045200
C	-2.53860900	1.88146700	-2.27369200
H	-2.95956300	0.94501100	-2.66516400
H	-3.13912100	2.25349000	-1.42933100
H	-2.40401900	2.64677000	-3.04949900
O	0.69941500	-2.46402000	-1.09086100
S	0.09640600	-2.93742800	0.31414300
O	-0.66971800	-1.79567600	0.89897400
O	1.09219900	-3.51902500	1.20149200
C	-1.01171300	-4.18953100	-0.26623200
H	-1.74787400	-3.73546000	-0.94500600
H	-0.40129800	-4.95663300	-0.76003600
H	-1.50164700	-4.59474700	0.62961500
I	3.33375300	2.24210200	-0.83723200
Mg	1.13742500	3.66214900	0.00532600
Mg	-2.35578200	-0.69982900	0.52923300
I	-3.59272500	-1.76558900	-1.71820400
O	-3.26140200	-1.93831300	1.97947200
C	-4.69115100	-1.98913400	2.03835400
H	-5.06978100	-1.94265500	1.00984000
H	-5.00618200	-2.93738900	2.50406900
H	-5.08267600	-1.13656200	2.61592000
C	-2.66082900	-2.00978200	3.27459400
H	-1.57339600	-2.04628700	3.13787200
H	-2.93339700	-1.12711800	3.87709900
H	-2.99350300	-2.92559600	3.79038700
I	-3.37379000	1.59312600	1.67235500
O	0.60271000	2.69534400	1.76037000
C	0.10823700	3.46183300	2.86175800
H	-0.00468600	4.49764900	2.51864000
H	-0.86993800	3.07077000	3.18260900
H	0.82481700	3.42528600	3.69991100
C	0.76197200	1.31172300	2.06214900
H	1.10910500	0.80627800	1.15578000
H	1.51383000	1.17631800	2.85787100
H	-0.20297500	0.88790100	2.38137500
C	0.78767500	5.73991200	0.16321900
H	1.05915100	6.27318800	-0.76850300
H	-0.28065100	5.96926700	0.34622100
H	1.35894700	6.23521600	0.97213600

15

C	-7.49999000	-2.38275800	-0.59888100
C	-6.57152100	-1.35739700	-0.39898200
C	-5.21382600	-1.54832900	-0.69677500
C	-4.80435200	-2.79227300	-1.20369600
C	-5.73138400	-3.81826900	-1.40497700
C	-7.08176000	-3.61839700	-1.10152600
H	-8.55464700	-2.21538200	-0.36463100
H	-6.90562800	-0.39096700	-0.00950300
H	-3.74882700	-2.93488400	-1.44852100
H	-5.39835600	-4.77938700	-1.80600500
H	-7.80671500	-4.42073600	-1.26070000
C	-4.19676900	-0.46343900	-0.43255700
C	-3.69065700	-0.50497500	1.01402100
H	-4.63856700	0.52279400	-0.64475500
H	-3.33669000	-0.60394300	-1.10262600
C	-2.46210200	0.32889000	1.31628200
H	-4.50235100	-0.27501600	1.72489800
H	-3.35751000	-1.53536900	1.22909300
C	-1.97277900	0.20960800	2.75984300
H	-1.66606700	0.08663200	0.60773300
H	-2.43476800	0.96342500	3.41465300
H	-2.31426400	-0.77124000	3.13333400
C	-0.47022200	0.18181900	2.93093900
H	-0.01991100	-0.59802100	2.30385000
H	-0.18415800	0.02176700	3.97804300
O	0.11549900	1.49172300	2.57858700
S	1.12025700	1.65283200	1.35945800
O	0.73612600	2.92942400	0.70497500
O	1.05581800	0.45274900	0.51281700
C	2.70127600	1.89828300	2.09823800
H	2.96536500	0.96636200	2.62086600
H	3.39636900	2.09435500	1.26794500
H	2.60812800	2.75827700	2.77451200
O	-0.82375100	-2.08499700	0.69549200
S	-0.52520400	-2.42607800	-0.71550600
O	0.86186300	-1.93315800	-1.13902500
O	-1.53766900	-2.01044300	-1.71026700
C	-0.37459000	-4.20611000	-0.79939000
H	0.43575500	-4.51499300	-0.12551300
H	-1.33117500	-4.63941600	-0.47723500
H	-0.15304900	-4.48892500	-1.83686200
I	-2.88860400	2.49418400	0.82249300
Mg	-0.54213200	3.59934300	-0.69757200
Mg	2.39976500	-1.07171600	-0.30088800
I	3.02625600	-1.89917000	2.27795200
O	3.70862800	-2.41937300	-1.26394600
C	5.10252100	-2.39027300	-0.96498800
H	5.21005200	-2.10103000	0.08802800
H	5.53727700	-3.39319700	-1.11236300
H	5.61923000	-1.65804600	-1.60692400
C	3.43441800	-2.82940700	-2.60147800
H	2.34533200	-2.83678900	-2.72392400
H	3.88577200	-2.12894300	-3.32486300
H	3.83657600	-3.84255600	-2.77212300
I	3.64583900	1.07341900	-1.64601700
O	-0.30822600	2.31128700	-2.24253300
C	0.36023400	2.75711300	-3.42912100
H	0.50190200	3.84350900	-3.34453800
H	1.33837400	2.25923100	-3.51431300
H	-0.26217900	2.53757000	-4.31174300
C	-0.60271400	0.90638800	-2.24098400
H	-1.08882100	0.67354600	-1.29084300
H	-1.29041800	0.66666100	-3.06590700
H	0.32223200	0.32296200	-2.33257400
C	-0.80235900	5.63117800	-1.15115500
H	-1.22244400	6.20866100	-0.30647500
H	0.15396500	6.12135800	-1.41567400
H	-1.48409300	5.78754800	-2.00841900
**16**			
I	2.50736700	-2.42491300	0.98891400
Mg	3.23835600	3.08502100	0.91477400
C	3.25018500	5.17884600	1.14957900
O	4.55165500	2.11039000	2.15763800
O	1.60848700	2.05406100	1.62943200
H	2.86620600	5.50147700	2.13717500

H	4.25640000	5.63013300	1.04729100
H	2.61231400	5.68309500	0.39810600
C	5.04038600	2.72614000	3.34620200
C	4.66131200	0.68501000	2.14586400
S	0.47026800	1.18373400	1.24498600
H	6.10775100	2.48766600	3.48749000
H	4.46851100	2.38111500	4.22495800
H	4.92004600	3.81180600	3.23219900
H	4.05674600	0.24372400	2.95491100
H	5.71527000	0.38586300	2.26856900
H	4.29166600	0.32703000	1.17853400
C	0.02216000	0.22984100	2.66076900
O	-0.81448400	2.08719700	1.02107600
O	0.67241000	0.32853900	0.07141600
H	-0.82261200	-0.41669900	2.39164900
H	0.90535500	-0.38183000	2.89435600
H	-0.22744200	0.92664300	3.47132900
C	-1.29672200	2.43431100	-0.33856200
Mg	1.43381100	-1.11593700	-1.27096600
C	-2.81190400	2.53378300	-0.29162800
H	-0.83540900	3.39759100	-0.58580900
H	-0.93978700	1.67278400	-1.04346400
O	2.39389700	-2.51385600	-2.52234300
O	2.53591700	0.33292200	-2.11109400
I	-1.06868100	-1.32494300	-2.42413000
C	-3.54147200	1.19120000	-0.36906400
H	-3.10739400	3.15692400	-1.14992800
H	-3.10513400	3.08795200	0.61432600
C	1.88431400	-3.84687700	-2.57588800
C	3.81363900	-2.46073900	-2.64616300
S	2.74118900	1.80647000	-1.86193400
C	-3.14358300	0.20517300	0.71078000
H	-3.41578400	0.73968000	-1.36167300
H	2.26597900	-4.43921000	-1.72769600
H	2.17844400	-4.31934400	-3.52773000
H	0.78984300	-3.78487100	-2.52536000
H	4.12269900	-2.93037000	-3.59498700
H	4.29480400	-2.97704600	-1.79873800
H	4.10470300	-1.40397500	-2.64683400
O	3.69675300	1.99599300	-0.69493400
C	-3.81192800	-1.17334000	0.71569500
H	-2.07210900	0.02236400	0.54304800
H	-3.24025800	0.67358600	1.70432100
C	-3.13612800	-2.04608700	1.74782600
H	-4.88475700	-1.07287700	0.93844200
H	-3.71812300	-1.62161500	-0.28557500
C	-1.93270800	-2.70583200	1.44529100
C	-3.62849900	-2.12331800	3.05967600
C	-1.24009800	-3.41975100	2.42659300
H	-1.53125800	-2.65008800	0.42962400
C	-2.93960900	-2.83918700	4.04367100
H	-4.56290200	-1.61337100	3.31190100
C	-1.74079400	-3.48717900	3.73070500
H	-0.29928500	-3.91141600	2.16915400
H	-3.34167200	-2.89096500	5.05905100
H	-1.19984400	-4.04528600	4.49913800
I	-5.72153500	1.61163000	-0.31394200
O	1.50741700	2.59224300	-1.68816600
C	3.61062500	2.38417200	-3.30052400
H	2.95746700	2.22274200	-4.16839600
H	3.81849700	3.45325700	-3.16051800
H	4.54129900	1.81036000	-3.39714000
**17**			
I	-2.99157300	0.80531600	1.40443200
Mg	-3.81414100	1.68120900	-1.17713200
C	-3.77753400	3.69483600	-1.76814700
O	-5.63350300	0.74563200	-1.15679300
O	-2.87340900	0.21734400	-2.29095100
H	-4.55196600	3.94390200	-2.51909100
H	-3.92232800	4.38504300	-0.91637700
H	-2.80546800	3.96704100	-2.21994500
C	-6.71070700	1.48007700	-0.57047100
C	-5.80237300	-0.67411000	-1.10051900
S	-1.45776300	-0.22124500	-2.31187000
H	-6.79820600	1.24366700	0.50324800

H	-7.65517600	1.23813300	-1.08402800
H	-6.49163000	2.54947600	-0.69789800
H	-6.73274000	-0.95780600	-1.61881200
H	-5.83767400	-1.01867500	-0.05441700
H	-4.94495200	-1.13610200	-1.60236300
C	-1.26171300	-1.45500900	-3.55735800
O	-0.64413000	1.03635300	-2.80108300
O	-0.88433100	-0.70171500	-1.04150800
H	-0.18825100	-1.68004700	-3.62973800
H	-1.83322100	-2.33180300	-3.21520600
H	-1.65363100	-1.04684300	-4.49842800
C	0.79506200	1.22181400	-2.44898200
Mg	-1.52473800	-1.57481300	0.79668200
C	0.91389300	2.51825500	-1.67665200
H	1.14046600	0.34261200	-1.89001100
H	1.31341100	1.26707200	-3.41271400
O	-1.88960600	-2.32617000	2.71156200
O	0.40244200	-1.79436300	1.09467900
I	-2.98095700	-3.48934600	-0.54837800
C	0.41468000	2.40844700	-0.23499600
H	0.36653000	3.29099100	-2.23454600
H	1.97675200	2.80619100	-1.68485800
C	-3.22560500	-2.44798800	3.19915300
C	-0.90168700	-2.62985900	3.69951100
S	1.65247000	-2.41245000	0.49904200
C	1.44607600	1.82000700	0.71605000
H	-0.53189500	1.85630800	-0.17808500
H	-3.40177500	-1.73327000	4.02005000
H	-3.40202400	-3.47673100	3.55357800
H	-3.91077300	-2.23257200	2.37088000
H	-1.13780400	-3.59570500	4.17534300
H	-0.87256900	-1.83809300	4.46691600
H	0.06621000	-2.69772500	3.19415700
C	1.09951100	-3.78786600	-0.49376500
O	2.56401700	-2.90739600	1.53605600
O	2.28165400	-1.42849700	-0.46504700
C	0.96874700	1.58142000	2.15466800
H	1.76439200	0.85383700	0.28769900
H	2.33983700	2.46508500	0.71497700
H	0.41557800	-3.41701000	-1.26644700
H	1.99088100	-4.24445200	-0.94334300
H	0.58114200	-4.49558100	0.16588000
C	2.09302200	1.17771600	3.07739500
H	0.49836100	2.50205600	2.53471900
H	0.19049000	0.80560700	2.15533300
C	2.25710400	-0.15287100	3.48552300
C	3.03100900	2.12990700	3.51140900
C	3.32880200	-0.52651700	4.30259200
H	1.55496200	-0.90713500	3.13387300
C	4.10350600	1.76087200	4.32599900
H	2.91655000	3.17335500	3.20368200
C	4.25671000	0.42828000	4.72532000
H	3.44048800	-1.57218300	4.59987300
H	4.82272600	2.51629500	4.65301900
H	5.09664900	0.13774100	5.36133400
I	-0.23665600	4.41695900	0.44509000
I	4.36336300	0.56359600	-2.91967400
Mg	4.16639900	-1.61556800	-1.23401800
O	5.23128500	-0.98406400	0.41961900
C	5.68844900	-1.95501900	1.36162300
H	6.64439700	-1.62490000	1.80184400
H	4.93810000	-2.10567300	2.15213900
H	5.84090800	-2.89554800	0.81707500
C	5.02889400	0.31143300	0.98979000
H	4.77489800	0.99498900	0.16955700
H	4.21553300	0.27995300	1.72657100
H	5.95673200	0.65539500	1.47513000
C	4.60087800	-3.64267400	-1.70539900
H	4.38380900	-4.34569900	-0.87698800
H	4.04023200	-4.01686300	-2.58542400
H	5.66943400	-3.79487800	-1.95599400
**TS18**			
I	-2.78205400	0.83969900	1.54731400
Mg	-3.71940900	1.88675100	-0.94030400
C	-3.75406400	3.93626300	-1.42042600

O	-5.58402500	1.03216100	-0.80313100
O	-2.96740700	0.44026100	-2.12623700
H	-4.57795700	4.19984900	-2.11244000
H	-3.86441900	4.58568000	-0.53155200
H	-2.81892300	4.25643100	-1.91718200
C	-6.62414700	1.78956900	-0.18713700
C	-5.79654400	-0.38100600	-0.76857200
S	-1.57226200	-0.05234000	-2.38522600
H	-6.70812200	1.53237200	0.88254200
H	-7.58496100	1.59347800	-0.69117100
H	-6.36803000	2.85268600	-0.29258100
H	-6.73122500	-0.63098400	-1.29731500
H	-5.85272700	-0.73787200	0.27272800
H	-4.94913800	-0.86225200	-1.26938400
C	-1.71280500	-1.28439900	-3.65181100
O	-0.70447900	1.05320400	-2.91560900
O	-0.95379000	-0.69325200	-1.18192500
H	-0.69863400	-1.63391900	-3.88742700
H	-2.33540700	-2.10005100	-3.25681900
H	-2.17443500	-0.80664800	-4.52598600
C	1.26379100	1.31005400	-2.41052800
Mg	-1.56197600	-1.56688400	0.60479300
C	1.07157700	2.55212000	-1.61102900
H	1.32558000	0.32378100	-1.94698300
H	1.45033200	1.38830200	-3.47757700
O	-1.86607900	-2.43069400	2.51777000
O	0.37642600	-1.90232400	0.87272800
I	-3.17613700	-3.44676900	-0.64471700
C	0.61224100	2.30973000	-0.17455300
H	0.34452200	3.17002800	-2.15982900
H	2.01133700	3.12596000	-1.62428500
C	-3.18111300	-2.50086300	3.06502700
C	-0.85574400	-2.81213600	3.45069700
S	1.54752200	-2.48714500	0.11855600
C	1.67650200	1.71918500	0.73679500
H	-0.29668600	1.69666700	-0.15735600
H	-3.29245200	-1.78439700	3.89593200
H	-3.38298200	-3.52382000	3.42359200
H	-3.89403000	-2.25787100	2.26850600
H	-1.12387700	-3.77491600	3.91637100
H	-0.74574900	-2.04411000	4.23534600
H	0.08279300	-2.92261000	2.90002300
C	0.86811700	-3.65459500	-1.04571000
O	2.49384500	-3.18081100	1.00028000
O	2.20088200	-1.40065200	-0.72019200
C	1.19786000	1.38683900	2.15500400
H	2.01876000	0.78490800	0.26088500
H	2.54812500	2.39311900	0.76985300
H	0.15605800	-3.13027600	-1.69401600
H	1.70567900	-4.06545800	-1.62478200
H	0.35776100	-4.44097400	-0.47471600
C	2.28811900	0.83013400	3.03753000
H	0.78614200	2.29762200	2.61800500
H	0.37001400	0.66695600	2.09263500
C	2.32258300	-0.53085300	3.37275600
C	3.30619400	1.66381500	3.52994500
C	3.33603100	-1.04561000	4.18750400
H	1.55770000	-1.19400800	2.97101700
C	4.32365800	1.15305700	4.33920400
H	3.29492700	2.72891500	3.28135500
C	4.34023000	-0.20541000	4.67507800
H	3.34383600	-2.11120100	4.43054200
H	5.10455400	1.81916000	4.71537200
H	5.13435500	-0.60505800	5.31068800
I	-0.13837600	4.24153800	0.60154100
I	4.17719900	1.04396200	-2.52203900
Mg	4.06366600	-1.56714100	-1.48713000
O	5.25222800	-1.37425000	0.17520400
C	5.71890300	-2.56628600	0.81146500
H	6.73625800	-2.40377300	1.20457000
H	5.03791400	-2.85637700	1.62602000
H	5.74167300	-3.35890700	0.05267300
C	5.20004800	-0.24784300	1.05678000
H	4.90093700	0.62422300	0.46330900
H	4.47301800	-0.43014100	1.85810100

H	6.19737800	-0.06828800	1.49001600
C	4.40599100	-3.38050300	-2.52427200
H	4.20457300	-4.27277300	-1.89898700
H	3.77503900	-3.48867200	-3.42858800
H	5.45005100	-3.48923000	-2.87751100
**19**			
Mg	3.24596100	-0.06616100	-0.30087500
O	2.42373300	-1.78071200	-1.12843800
C	1.07235100	-1.89116800	-1.58319600
H	1.02176100	-2.56213900	-2.45674100
H	0.43041600	-2.28332900	-0.78046600
H	0.74027300	-0.89007000	-1.88018400
C	2.98536700	-3.02750400	-0.71590600
H	2.96537100	-3.74562600	-1.55272700
H	4.02844800	-2.83921300	-0.42664100
H	2.42416700	-3.43024100	0.14188900
Mg	-1.37810000	0.43707900	0.15869400
O	-2.49261100	1.94733100	0.93992900
C	-3.88495700	1.83697400	1.24475700
H	-4.40464000	2.76584800	0.95835000
H	-4.28588100	0.99429900	0.66633400
H	-4.02720800	1.64888500	2.32221800
C	-1.83124100	3.02447900	1.61357800
H	-1.81943400	2.84333000	2.70116500
H	-0.80381900	3.08522400	1.23140800
H	-2.35610800	3.97044700	1.40191100
O	2.21678600	-0.03799500	1.41432900
S	1.01103500	-0.69804000	2.03655900
O	-0.23373300	0.11381600	1.71194800
O	0.87471000	-2.12459400	1.72349700
C	1.22124700	-0.48752900	3.78955600
H	2.13106700	-1.02897900	4.08092900
H	0.33928100	-0.91409500	4.28535900
H	1.31828200	0.58486200	4.00214400
S	1.02261800	2.15004100	-1.16056800
O	-0.25543900	1.32355600	-1.18545700
O	2.20671400	1.24367400	-1.39704300
C	0.92549800	3.15821700	-2.62206300
H	0.05148500	3.81465100	-2.51842800
H	1.85069400	3.74681900	-2.67965900
H	0.82271100	2.49915700	-3.49350000
O	1.13862600	3.00895000	0.02370800
C	5.35647000	-0.15982800	-0.25222100
H	5.78766100	-0.60396100	-1.17106600
H	5.83167700	0.83482400	-0.14556100
H	5.73823000	-0.76752400	0.59196300
I	-2.92143200	-1.50340100	-0.81613600
**20**			
C	5.54004500	-2.40974300	0.46256800
C	4.39493300	-1.77067400	0.94520600
C	3.93177200	-0.58236200	0.35996000
C	4.64532900	-0.05082800	-0.72539000
C	5.79092600	-0.68603200	-1.21113400
C	6.24218700	-1.86903000	-0.61845800
H	5.88778500	-3.33206500	0.93506800
H	3.85298900	-2.19711700	1.79447300
H	4.29792400	0.87577000	-1.19134800
H	6.33579200	-0.25343900	-2.05420600
H	7.13966800	-2.36576700	-0.99531500
C	2.66774500	0.08466900	0.84703500
C	1.41933700	-0.44914800	0.13187100
H	2.55399000	-0.07360100	1.93252500
H	2.74056900	1.17172300	0.68953800
C	0.10139700	0.17604700	0.56659300
H	1.33586600	-1.53302100	0.33336100
H	1.53592500	-0.35100000	-0.95978800
C	-1.11677200	-0.54478000	-0.00357000
H	0.03890000	0.24144200	1.66213900
H	-1.00520200	-1.60733000	0.27563200
H	-1.09705200	-0.50448600	-1.10396500
C	-2.44210300	-0.02831200	0.52511600
H	-2.50508300	-0.07467100	1.61973100
H	-2.66964300	0.98917900	0.18837400
I	0.05544700	2.32612800	-0.02090900
I	-4.12131900	-1.23564600	-0.18295900

32

C	0.71592000	-0.48221900	2.95667000
C	-0.66436800	-0.30504600	2.90143500
C	-1.70502100	-1.32745500	3.31028000
H	-1.87064900	-1.31348200	4.40700200
H	-2.66473400	-1.02382800	2.86010200
C	-1.37969300	-2.76549400	2.84912400
H	-2.31696600	-3.34291500	2.79751300
H	-0.76055100	-3.27406100	3.60330800
C	-0.68795100	-2.81296200	1.49496300
C	0.69144000	-2.84650200	1.30550500
C	1.73251200	-2.76881000	2.40264600
H	1.88922100	-3.76145800	2.87228200
H	2.69234300	-2.51070900	1.92744300
C	1.41902000	-1.71973000	3.49523800
H	0.81332100	-2.17494300	4.29317200
H	2.36318300	-1.41854200	3.97761100
H	-1.31359800	-3.14365500	0.66076100
H	1.06095900	-3.22914700	0.34734000
H	-1.03320100	0.72490600	2.83418900
H	1.33098400	0.42084300	2.89372100
Ni	0.01463800	-0.89875500	1.01970100
P	-1.34390100	-0.05968600	-0.50859900
P	1.40260400	0.10179400	-0.37760200
C	0.07940400	0.21879700	-1.68598900
C	-2.13316500	1.57694700	-0.24930400
C	-2.58680300	-0.96167700	-1.52976700
C	1.82705200	1.87504600	-0.07757400
C	2.91135700	-0.51616900	-1.22187600
H	0.13163300	-0.67754300	-2.32101300
H	0.07334600	1.10858700	-2.33386200
C	-1.85479300	2.72064400	-1.01134000
C	-3.01384300	1.68867000	0.84226500
C	-2.25231100	-2.26746900	-1.93219100
C	-3.84173000	-0.44409300	-1.88244500
C	2.84762200	2.55660800	-0.75871500
C	1.05048200	2.57264900	0.86163100
C	3.01548000	-0.68846200	-2.61027600
C	4.00278800	-0.87146300	-0.40960100
C	-2.44080900	3.94830500	-0.68928100
H	-1.16944800	2.66642600	-1.85799200
C	-3.61105400	2.91072400	1.15433900
H	-3.22870100	0.80689100	1.45136000
C	-3.14489700	-3.03075200	-2.68567100
H	-1.28224700	-2.68658800	-1.65029100
C	-4.73989500	-1.21467400	-2.62770500
H	-4.11981900	0.56670600	-1.57861600
C	3.07828000	3.91237000	-0.51032400
H	3.46654400	2.02739200	-1.48630800
C	1.27583200	3.92952700	1.10254700
H	0.25878000	2.04205700	1.39252200
C	4.18656300	-1.20526400	-3.17432700
H	2.18409600	-0.41264900	-3.26267100
C	5.17427400	-1.37774400	-0.97334600
H	3.92903900	-0.74538300	0.67416700
C	-3.32038600	4.04720400	0.39148000
H	-2.20482200	4.83238000	-1.28686900
H	-4.29680500	2.97935900	2.00254900
C	-4.39484300	-2.50650900	-3.03293900
H	-2.86823900	-4.04168100	-2.99572700
H	-5.71435500	-0.79874200	-2.89675900
C	2.29188600	4.60253000	0.41790000
H	3.87678000	4.43299200	-1.04554100
H	0.65560500	4.45994000	1.82928200
C	5.26769100	-1.54942800	-2.35910800
H	4.25456900	-1.33471100	-4.25757600
H	6.01582200	-1.64607200	-0.32966900
H	-3.77639600	5.00808100	0.64243300
H	-5.09849600	-3.10565200	-3.61614400
H	2.47433800	5.66296100	0.60971800
H	6.18182400	-1.95284900	-2.80153400

TS33

Ni	1.43913100	-0.11630900	0.96061100
I	-1.26524400	0.28776700	0.58165400
H	-3.39784700	0.51948900	-1.48859800

C	-3.68539100	0.60831600	-0.43234700
C	-4.07927100	1.99405900	-0.00410600
H	-5.15460800	2.16031200	-0.23011800
H	-3.99983200	2.07807200	1.09369200
C	-4.50466400	-0.54381300	0.08727300
H	-5.55279700	-0.42208000	-0.25705700
H	-4.54296300	-0.51737500	1.18938200
C	-3.97305100	-1.88478600	-0.38675800
H	-3.94563300	-1.96832200	-1.48000500
H	-2.98293900	-2.11465400	0.01957700
C	-3.25104900	3.09939500	-0.67798700
H	-2.18851400	2.81542100	-0.62350300
H	-3.50737500	3.14923400	-1.74866200
C	-3.41576300	4.45894800	-0.04768400
C	-2.91257900	4.69773200	1.24329700
C	-4.04737000	5.51471800	-0.71892000
C	-3.03507500	5.95291000	1.84118500
H	-2.40586500	3.88726000	1.77503300
C	-4.17103500	6.77560100	-0.12501800
H	-4.44118600	5.34771900	-1.72555500
C	-3.66493100	6.99943900	1.15732500
H	-2.63324900	6.11887200	2.84432800
H	-4.66497800	7.58583600	-0.66796400
H	-3.75959400	7.98348200	1.62338900
I	-5.23382200	-3.55873800	0.25426000
C	4.34564000	-0.18404800	4.66509000
C	3.64344300	-1.20005600	5.18898500
C	2.69968300	-2.17819900	4.54683500
H	3.14318900	-3.18911400	4.63881800
H	1.78248600	-2.21943800	5.16406900
C	2.30227200	-1.94341300	3.08339900
H	1.72658100	-2.82191800	2.74901200
H	3.20220100	-1.93937800	2.45605800
C	1.43287800	-0.72112300	2.85606400
C	1.81123300	0.63805700	2.77652100
C	3.18354100	1.27519800	2.93464800
H	3.42900500	1.85663900	2.02936000
H	3.11593900	2.02288200	3.74679800
C	4.37114000	0.35081700	3.25249600
H	4.43387300	-0.45418300	2.50697400
H	5.29360500	0.93974900	3.12690400
H	0.38605000	-0.89277200	3.13713100
H	0.99620400	1.34671700	2.97036500
H	3.77448500	-1.36909800	6.26592400
H	4.95984100	0.38656400	5.37312400
P	1.94372700	1.14082200	-0.89754400
P	1.37644600	-1.60455900	-0.78143100
C	1.35228800	-0.21339500	-2.03202700
C	3.69604800	1.44939000	-1.34932400
C	1.11381800	2.67876600	-1.43978200
C	2.96629200	-2.46283900	-1.10190800
C	0.08116700	-2.78068700	-1.30219300
H	0.29838800	0.01093500	-2.25026800
H	1.89471900	-0.36926700	-2.97530300
C	4.45226200	0.59435100	-2.16378300
C	4.32722500	2.56213700	-0.76244800
C	0.61226800	3.54616500	-0.45905300
C	0.96597000	3.00615600	-2.79609700
C	3.13230400	-3.46388800	-2.07068800
C	4.06973200	-2.06194400	-0.33154400
C	-0.45123900	-2.81789700	-2.59902100
C	-0.43751700	-3.64202100	-0.32113600
C	5.81018600	0.84508900	-2.38543900
H	3.99608100	-0.28230500	-2.62215100
C	5.68008000	2.81306500	-0.98898400
H	3.75022600	3.24179700	-0.12981900
C	-0.01547400	4.73865300	-0.82919200
H	0.69673100	3.27069200	0.59422900
C	0.31933000	4.18762100	-3.16414400
H	1.35759100	2.33648700	-3.56624600
C	4.38788100	-4.04304900	-2.27133700
H	2.27803400	-3.79358900	-2.66637600
C	5.32475900	-2.63736600	-0.53582200
H	3.93689400	-1.28150700	0.42165800
C	-1.48295200	-3.70892800	-2.91067100

H	-0.06903500	-2.14644900	-3.37110200
C	-1.46263400	-4.53436600	-0.63552600
H	-0.04977500	-3.58976300	0.69943600
C	6.42791600	1.95192300	-1.80049300
H	6.38502000	0.16489300	-3.01869400
H	6.15398400	3.68410000	-0.52970400
C	-0.16779700	5.05637900	-2.18102400
H	-0.41129800	5.40657900	-0.06237100
H	0.19833300	4.43456500	-4.22195300
C	5.48503300	-3.62955800	-1.50736700
H	4.51063300	-4.82359300	-3.02647100
H	6.17669600	-2.30918900	0.06424600
C	-1.98940300	-4.56711100	-1.93109500
H	-1.89596500	-3.72800200	-3.92233600
H	-1.87263800	-5.18812300	0.13715000
H	7.48899300	2.14568600	-1.97520300
H	-0.67824700	5.97873500	-2.46815000
H	6.46492400	-4.08632300	-1.66680600
H	-2.80727400	-5.25050200	-2.16939600
**34**			
C	-5.78093200	0.21357100	-0.89830200
C	-4.66219000	0.90887100	-0.43242400
C	-3.72166300	0.28634300	0.40316600
C	-3.93217200	-1.05488900	0.75867300
C	-5.04940600	-1.75460600	0.29541100
C	-5.97809600	-1.12218800	-0.53613200
H	-6.50408900	0.71797600	-1.54447200
H	-4.51642000	1.95495900	-0.71737400
H	-3.21105400	-1.55552000	1.41142200
H	-5.19701400	-2.79758900	0.58743700
H	-6.85385600	-1.66687300	-0.89757900
C	-2.48517200	1.02226300	0.85919800
C	-1.31829100	0.89676100	-0.13515500
H	-2.71762800	2.09097300	1.00048100
H	-2.16068200	0.63648400	1.83955700
C	-0.08344100	1.62169600	0.27680500
H	-1.65834800	1.26290800	-1.12846100
H	-1.09254800	-0.17375500	-0.30540200
C	1.25528800	1.27482700	-0.28636400
H	-0.14911600	2.40112800	1.04369800
H	1.90103400	2.16533100	-0.35952900
H	1.15470200	0.85534400	-1.30167500
C	1.92832600	0.24220300	0.61904300
H	2.13507500	0.63307200	1.62265700
H	1.35230400	-0.68797200	0.69704200
I	3.89592700	-0.39708600	-0.14134600
**35**			
Ni	1.02994800	0.06463200	0.33902200
P	-0.50417200	-1.37419800	-0.78762800
P	-0.88491600	1.36915500	-0.18486300
C	-1.24741900	0.14843200	-1.54543700
C	-1.82951600	-2.10866100	0.23837100
C	-0.27436700	-2.56465900	-2.16197500
C	-2.42600300	1.28425200	0.81396100
C	-0.96123500	3.03252800	-0.94462200
H	-0.59956700	0.38988100	-2.40025700
H	-2.29052300	0.09719200	-1.89056200
C	-3.19676500	-1.87077400	0.03441200
C	-1.43265900	-2.92704700	1.30935000
C	0.86485400	-2.41739600	-2.97221400
C	-1.17172000	-3.61389100	-2.41598300
C	-3.67038300	1.65067400	0.27334200
C	-2.37189400	0.76673400	2.11414000
C	-0.44014900	3.26720400	-2.22742500
C	-1.44135700	4.11972100	-0.19362300
C	-4.14665800	-2.42715700	0.89332200
H	-3.52977700	-1.23866900	-0.78905800
C	-2.38344700	-3.49078600	2.16263100
H	-0.37043500	-3.12146300	1.47730600
C	1.08708600	-3.29600700	-4.03426900
H	1.57744900	-1.61354400	-2.76768300
C	-0.93961200	-4.49424700	-3.47580400
H	-2.05301900	-3.74607300	-1.78573900
C	-4.83787200	1.49023800	1.01972600
H	-3.72594100	2.06133000	-0.73781100

C	-3.54199800	0.60385300	2.86140400
H	-1.41207000	0.47279100	2.53660200
C	-0.42515300	4.56008200	-2.75598000
H	-0.02559100	2.44613100	-2.81307400
C	-1.42118100	5.41047100	-0.72466300
H	-1.83998900	3.95653200	0.81001600
C	-3.74288400	-3.23580200	1.95944000
H	-5.20711200	-2.22118600	0.73139200
H	-2.06068200	-4.12349700	2.99293600
C	0.18687500	-4.33568600	-4.28806200
H	1.97390300	-3.17141200	-4.66045600
H	-1.64339700	-5.30834500	-3.66676400
C	-4.77553200	0.96242800	2.31497400
H	-5.80146700	1.77511400	0.58988300
H	-3.48710100	0.18858100	3.87028800
C	-0.91678200	5.63396000	-2.00938500
H	-0.01905100	4.72676700	-3.75658100
H	-1.80215400	6.24537100	-0.13122100
H	-4.48772000	-3.66558400	2.63362600
H	0.36628700	-5.02617400	-5.11596100
H	-5.69152300	0.83161400	2.89637400
H	-0.90228400	6.64428700	-2.42541200
I	2.63625500	0.93335700	-1.55769900
H	5.45844800	-1.17320400	2.75626300
C	4.38637500	-1.44467000	2.81465100
H	4.19542700	-1.97477300	4.93660000
H	3.35323700	1.36692400	3.22140900
C	3.89415400	-1.20114200	4.21888400
H	4.36479900	-2.53595500	2.64223600
C	3.16139500	-0.18801100	4.70441000
H	2.47674000	1.83332600	4.66358100
C	2.62179100	1.00242500	3.95392300
H	2.90073300	-0.23319200	5.76916800
H	3.79762900	0.32709000	1.66851200
C	3.63913400	-0.75710300	1.65612000
H	4.08534800	-1.09990600	0.71063300
C	1.26414900	0.75530300	3.26033700
H	0.99302700	1.67801300	2.71819000
C	2.16830000	-1.09445400	1.64345300
C	1.15125100	-0.46365000	2.35256200
H	0.49316800	0.64354300	4.04148200
H	1.95771800	-2.11919800	1.31266500
H	0.24710700	-1.05722500	2.51441100

COD

H	2.72830200	-0.30927200	0.67559200
C	1.91946200	0.00254700	-0.01361800
H	1.80997700	-1.83461700	-1.19431300
H	-0.64781000	-0.71245500	1.60151200
C	1.20796400	-1.23076000	-0.50314800
H	2.44474000	0.44788100	-0.87822300
C	-0.01923200	-1.69578000	-0.22527400
H	-1.77045000	-1.90088800	0.97267800
C	-1.08021100	-1.09773300	0.66848200
H	-0.31404500	-2.62093500	-0.73592300
H	0.64779200	0.71244500	1.60152900
C	1.08020300	1.09772700	0.66850500
H	1.77044000	1.90088000	0.97271400
C	-1.91946200	-0.00254900	-0.01364600
H	-2.72831500	0.30926200	0.67555200
C	0.01923400	1.69578300	-0.22525700
C	-1.20795800	1.23076500	-0.50315100
H	-2.44472300	-0.44787700	-0.87826500
H	0.31405100	2.62094400	-0.73589100
H	-1.80996300	1.83463100	-1.19431500

36

Ni	0.32054600	-1.24135400	-0.54298500
P	1.10631400	0.51185000	0.64093300
P	-1.52694200	-0.28780900	0.36374200
C	-0.46485200	0.55689800	1.64057000
C	1.20512700	2.11942900	-0.22023300
C	2.49059200	0.48496900	1.83059700
C	-2.13296500	1.10815800	-0.66113700
C	-3.01094400	-0.90681100	1.22949000
H	-0.31945100	-0.14449400	2.47705600
H	-0.80569100	1.52510300	2.03523900

C	0.50645700	3.26791800	0.17726800
C	1.96954200	2.15280000	-1.39897000
C	3.10322000	-0.75640700	2.06166500
C	2.94547900	1.62524900	2.51030200
C	-3.10813200	2.01128200	-0.21290600
C	-1.51598800	1.31381400	-1.90395000
C	-3.38449900	-0.48061200	2.51330000
C	-3.79945500	-1.86069000	0.56458900
C	0.56993400	4.43182100	-0.59247800
H	-0.10977800	3.25722800	1.07732100
C	2.04113200	3.32015900	-2.15972700
H	2.49179500	1.25043700	-1.72879500
C	4.15095900	-0.85936600	2.98035600
H	2.76100500	-1.63374200	1.50532400
C	3.99370200	1.51810900	3.42587300
H	2.48535800	2.59733100	2.31779800
C	-3.44769000	3.11689400	-0.99501300
H	-3.60219500	1.85259700	0.74875700
C	-1.85356800	2.42274300	-2.68205900
H	-0.75619400	0.60610900	-2.24473600
C	-4.53346200	-0.99699800	3.11919000
H	-2.78071500	0.25644700	3.04713300
C	-4.94963400	-2.36933400	1.16920500
H	-3.50473600	-2.20902200	-0.42934900
C	1.33662200	4.46050500	-1.76023000
H	0.00961500	5.31686600	-0.28202400
H	2.63631100	3.33518100	-3.07594700
C	4.59480100	0.27650000	3.66317100
H	4.62733400	-1.82705900	3.15498800
H	4.34740600	2.40711800	3.95401700
C	-2.81732900	3.32611900	-2.22698600
H	-4.20687600	3.81957900	-0.64242000
H	-1.35775500	2.58258600	-3.64205600
C	-5.31809000	-1.93865200	2.44806400
H	-4.81623200	-0.66112500	4.11993500
H	-5.55631500	-3.11101000	0.64410800
H	1.37941900	5.37003800	-2.36434100
H	5.41811500	0.19744300	4.37747800
H	-3.08180600	4.19529100	-2.83431300
H	-6.21518200	-2.34199700	2.92394600
I	1.45028200	-3.00586000	-1.92689300
**TS37**			
C	-1.75335400	-2.56238900	0.46160800
C	-0.95198500	-3.64421600	-0.25257600
H	-1.40315600	-1.54959300	0.20970800
C	0.46452000	-3.61543600	0.22215900
H	-1.42178300	-4.62537900	-0.04239300
H	-0.99969500	-3.48404300	-1.34011800
C	1.65710500	-3.44302600	-0.65761100
H	0.63913000	-3.71845600	1.30021500
C	2.50089000	-2.20626300	-0.27160500
H	1.35031100	-3.35317300	-1.71146700
H	2.31280300	-4.33488600	-0.59158100
H	1.85156300	-1.32555000	-0.38392900
H	2.76226400	-2.27248100	0.79591800
H	-1.76222200	-2.69319300	1.54938300
C	3.74843900	-2.00871900	-1.09139000
C	4.99441300	-2.46626200	-0.63699700
C	3.68634800	-1.36107000	-2.33643500
C	6.15005200	-2.28197800	-1.40124600
H	5.05909700	-2.96701200	0.33360900
C	4.83973200	-1.17330200	-3.10160800
H	2.72107500	-1.00317600	-2.70464500
C	6.07642800	-1.63292500	-2.63697400
H	7.11232400	-2.64294100	-1.02837500
H	4.77145600	-0.66450600	-4.06677000
H	6.97909100	-1.48497200	-3.23530800
Ni	-0.10182300	0.16393000	-0.55277400
P	0.65854300	0.30088900	1.58105400
P	-0.95036700	2.11584400	0.21699000
C	-0.55766700	1.65369600	1.98208300
C	2.28088700	1.13992700	1.60114600
C	0.67694700	-0.85912800	2.99452900
C	-0.15522600	3.72573100	-0.10735600
C	-2.74387400	2.43855700	0.15614200

H	-1.47216600	1.20329400	2.39540000
H	-0.21640300	2.44638600	2.66426500
C	2.83843600	1.69581300	2.76447400
C	2.93259600	1.30831800	0.37077300
C	-0.53427000	-1.26237400	3.58263000
C	1.87156900	-1.46839700	3.41307600
C	0.89254900	4.22981400	0.67822600
C	-0.53878800	4.42460600	-1.26661900
C	-3.34233600	3.56744400	0.73942500
C	-3.54043700	1.46703800	-0.46718500
C	4.03534600	2.40954700	2.69098600
H	2.33529100	1.57091100	3.72661200
C	4.13409000	2.01711600	0.30076500
H	2.48875100	0.88400600	-0.53238500
C	-0.54859300	-2.24858600	4.57058700
H	-1.47921700	-0.81514700	3.26608300
C	1.85344300	-2.45869100	4.39833500
H	2.82207600	-1.16825600	2.96861400
C	1.53767900	5.41554000	0.31631600
H	1.22534000	3.69784100	1.56912200
C	0.10410900	5.61066600	-1.62053500
H	-1.34370400	4.03586500	-1.89570600
C	-4.72908700	3.71478400	0.70106400
H	-2.72273600	4.33029100	1.21732400
C	-4.92941300	1.61765900	-0.49998800
H	-3.06576600	0.59783600	-0.92843300
C	4.68341300	2.57013000	1.45938700
H	4.46697600	2.84293500	3.59658400
H	4.63665400	2.13376800	-0.66175900
C	0.64483200	-2.85220000	4.97913400
H	-1.49796100	-2.54918000	5.02022100
H	2.79074700	-2.92369300	4.71334600
C	1.14519900	6.10986900	-0.83006600
H	2.35434600	5.79302100	0.93632100
H	-0.20609000	6.14552400	-2.52147100
C	-5.52276000	2.73959400	0.08308400
H	-5.19577900	4.59314500	1.15393600
H	-5.54300900	0.85482900	-0.98445800
H	5.62227600	3.12685200	1.40706700
H	0.63197500	-3.62655300	5.74974000
H	1.65137000	7.03647000	-1.11133500
H	-6.60862400	2.86010100	0.05506900
I	-0.42159300	-0.65962700	-2.91901900
I	-3.87700300	-2.54145400	-0.10188300
**38**			
C	3.03986900	0.90630800	0.10946600
C	2.13965600	2.01837500	-0.38643100
H	2.74868000	-0.07763900	-0.27884400
C	0.70522300	1.86828000	0.13917700
H	2.55555700	2.99241300	-0.06441700
H	2.13107900	2.02332100	-1.48741800
C	-0.19372800	2.98770300	-0.38364500
H	0.73926700	1.95269000	1.23966800
C	-1.67384700	2.82771600	-0.01208100
H	-0.11128500	3.05160500	-1.47991300
H	0.16498900	3.96071300	0.00898500
H	-2.01930500	1.86199900	-0.41744000
H	-1.77607800	2.77132100	1.08412100
H	3.11309300	0.86776000	1.20036400
C	-2.54908700	3.92875200	-0.55450600
C	-2.99695600	4.97944300	0.25938100
C	-2.90584700	3.93825200	-1.91363500
C	-3.78180200	6.01132400	-0.26481000
H	-2.72757400	4.98662000	1.31972800
C	-3.68914800	4.96660200	-2.44100800
H	-2.56023400	3.12530700	-2.55878100
C	-4.13056700	6.00842000	-1.61782000
H	-4.12241000	6.82046900	0.38691900
H	-3.95795700	4.95538800	-3.50065800
H	-4.74424700	6.81358200	-2.02991500
Ni	0.01513500	0.03556900	-0.28773600
P	0.09629300	-0.68863400	1.77608000
P	-0.67846900	-2.17150800	-0.35657200
C	0.13779000	-2.47843800	1.29267400
C	-1.43779600	-0.52669000	2.75491300

C	1.44976900	-0.35087100	2.94498200
C	-2.46673000	-2.41284700	-0.09946200
C	-0.11738500	-3.49642900	-1.47512100
H	1.17552200	-2.76046500	1.05845600
H	-0.28615300	-3.21137000	1.99437600
C	-1.52353300	-1.03641300	4.06097300
C	-2.57270600	0.02955200	2.15039200
C	2.60294400	-1.14876500	2.98557700
C	1.39113200	0.81679800	3.72567100
C	-3.00180700	-3.18998900	0.93757100
C	-3.33140900	-1.70629200	-0.95324900
C	-0.84427100	-4.68114300	-1.66455300
C	1.09184100	-3.30228900	-2.16258100
C	-2.73778700	-0.99161100	4.74722800
H	-0.64123000	-1.46684800	4.54082800
C	-3.78662100	0.07148300	2.83937000
H	-2.50488300	0.41569400	1.13470500
C	3.68163600	-0.78451000	3.79436700
H	2.67470200	-2.05185900	2.37704000
C	2.47287700	1.17839200	4.52994300
H	0.49731400	1.44414700	3.70439800
C	-4.38437500	-3.25700100	1.12249900
H	-2.34435300	-3.73476800	1.61693400
C	-4.71281900	-1.78263300	-0.76978400
H	-2.91496000	-1.08551000	-1.75111800
C	-0.35339800	-5.67088600	-2.51912400
H	-1.79563000	-4.82830600	-1.14913800
C	1.58132000	-4.29820300	-3.00944500
H	1.63548400	-2.36118000	-2.05311400
C	-3.87021600	-0.43947400	4.13656100
H	-2.80219900	-1.38874000	5.76320900
H	-4.66675700	0.50032900	2.35564800
C	3.62064000	0.38038100	4.56408100
H	4.57592100	-1.41132600	3.81721600
H	2.42014000	2.08967100	5.13012400
C	-5.24083900	-2.55328600	0.27112600
H	-4.79302900	-3.85545100	1.94006200
H	-5.37848500	-1.22797900	-1.43531200
C	0.85995100	-5.48256300	-3.18822900
H	-0.92333100	-6.59156500	-2.66614100
H	2.52056300	-4.14025400	-3.54463300
H	-4.81950300	-0.40780300	4.67683900
H	4.46865200	0.66740200	5.19007900
H	-6.32201400	-2.60158200	0.42201900
H	1.23918600	-6.25687100	-3.85963200
I	-0.16555200	0.38684000	-2.84818100
I	5.12835300	1.12449200	-0.54095200

39

C	1.78147100	2.40838700	-0.41810500
C	1.40403500	1.55662000	-1.60989400
H	1.67682200	1.87834500	0.53193800
C	-0.09701100	1.22871500	-1.63542800
H	1.66515500	2.09711600	-2.53953100
H	2.00087300	0.63021100	-1.62399900
C	-0.46170000	0.52765700	-2.94645600
H	-0.65680500	2.18130500	-1.59339400
C	-1.92848200	0.09421400	-3.06429900
H	0.18279800	-0.35271300	-3.08958100
H	-0.23183300	1.20814500	-3.79025900
H	-2.17490800	-0.52848100	-2.18906300
H	-2.57577000	0.98415800	-3.01853200
H	1.21812700	3.34438000	-0.38147600
C	-2.22662200	-0.68693600	-4.31897700
C	-2.79775500	-0.07357900	-5.44354300
C	-1.90082400	-2.05134600	-4.39630200
C	-3.04061900	-0.79994100	-6.61325500
H	-3.05576100	0.98865600	-5.40011500
C	-2.14245700	-2.78073800	-5.56217300
H	-1.44627200	-2.54037400	-3.53005500
C	-2.71404900	-2.15713300	-6.67627600
H	-3.48717300	-0.30328800	-7.47880500
H	-1.88175300	-3.84162500	-5.60260300
H	-2.90308100	-2.72696500	-7.58956300
Ni	-0.64676900	0.16619100	-0.03051700
P	-1.62800100	1.83435800	1.01540500

P	-1.71076000	-0.75011900	1.85122900
C	-1.80388700	0.92932400	2.62811100
C	-3.34879900	2.25579500	0.57432400
C	-0.80664200	3.42560300	1.34038000
C	-3.42521500	-1.16653800	1.36494200
C	-1.28442100	-1.96408700	3.14401500
H	-0.87906600	1.07598400	3.20336500
H	-2.67042100	1.19042200	3.25191300
C	-4.07227800	3.18544000	1.34059300
C	-3.98604000	1.57142800	-0.46676100
C	0.23039800	3.51160600	2.28309600
C	-1.09480600	4.53368800	0.52644200
C	-4.53712200	-0.79536100	2.13485300
C	-3.62001100	-1.82920400	0.14237700
C	-1.56894500	-3.32010700	2.91049400
C	-0.61686000	-1.59385100	4.32139200
C	-5.41900900	3.42017800	1.06253400
H	-3.58242600	3.72630000	2.15386200
C	-5.33508000	1.80735300	-0.74210600
H	-3.42917700	0.84303300	-1.05110500
C	0.96623400	4.68993600	2.40962500
H	0.50175300	2.64837600	2.89316800
C	-0.35486300	5.71078900	0.65816800
H	-1.89285900	4.47629300	-0.21654500
C	-5.82711500	-1.06674000	1.67772800
H	-4.39979000	-0.28190200	3.08874700
C	-4.91247700	-2.11038600	-0.30705600
H	-2.75717800	-2.11745100	-0.46171000
C	-1.19558700	-4.28715200	3.84445000
H	-2.08366400	-3.62019300	1.99548800
C	-0.24713900	-2.56577600	5.25456100
H	-0.37227100	-0.54898100	4.51573500
C	-6.05226500	2.73030600	0.02179200
H	-5.97816700	4.14384500	1.66052300
H	-5.82344500	1.26145600	-1.55207600
C	0.67769700	5.79006300	1.59661100
H	1.78118800	4.74138300	3.13474200
H	-0.58221800	6.56659400	0.01837900
C	-6.01650200	-1.72151000	0.45583400
H	-6.68964000	-0.76196200	2.27504800
H	-5.05384300	-2.62172800	-1.26209500
C	-0.53337600	-3.91312500	5.01839000
H	-1.42191100	-5.33883400	3.65328700
H	0.27147800	-2.26622600	6.16826100
H	-7.10797600	2.91482300	-0.19130700
H	1.26403200	6.70694600	1.68947000
H	-7.02832900	-1.92694100	0.09812000
H	-0.24103400	-4.67150300	5.74844800
I	0.43404700	-2.13207300	-0.76910600
I	3.86632000	3.07321800	-0.41337400
Mg	2.78160000	-1.32148600	0.82648400
I	4.83239200	-1.17843800	-0.95131300
O	2.94021800	-3.26491900	1.57357100
C	3.52941900	-4.34608200	0.85164200
H	3.97240300	-5.06902600	1.55714100
H	4.31116000	-3.92824500	0.20547800
H	2.76950600	-4.85224300	0.23183700
C	1.91864200	-3.68858200	2.47453900
H	1.50691100	-2.79495600	2.95603100
H	2.34491400	-4.35724600	3.24071500
H	1.11966800	-4.21004300	1.92589700
C	2.07500100	0.01578900	2.32785600
H	2.08806000	-0.40534200	3.35028900
H	1.02892900	0.29834500	2.10898000
H	2.66710100	0.94861800	2.35985900
**TS40**			
C	-1.19536200	3.45491400	-0.54605400
C	-0.84936900	2.72825000	0.73661300
H	-1.21117100	2.80597100	-1.42369600
C	0.49162400	1.98572100	0.59983600
H	-0.78095500	3.47457800	1.55000200
H	-1.65928300	2.04704200	1.02487600
C	1.26925300	2.02793200	1.91678300
H	1.09683600	2.54222300	-0.13335200
C	2.65652200	1.37986200	1.86972400

H	0.68403000	1.56747700	2.72816300
H	1.39920700	3.09039600	2.20600600
H	2.53360500	0.31029500	1.64340600
H	3.23071100	1.81928300	1.03796300
H	-0.53668000	4.30950400	-0.74006400
C	3.42875900	1.53421000	3.15544700
C	4.37611700	2.55662600	3.31558300
C	3.18400400	0.67722200	4.24118000
C	5.06180900	2.72069000	4.52265200
H	4.57969000	3.23206200	2.47935500
C	3.86645200	0.83758900	5.44919500
H	2.44689000	-0.12313700	4.13222100
C	4.80869900	1.86096800	5.59503700
H	5.79781800	3.52263900	4.62535400
H	3.66278400	0.15870100	6.28154700
H	5.34419000	1.98630500	6.53955100
I	-3.21250000	4.32623800	-0.51989100
Mg	-2.31994600	-0.72005300	0.79313500
I	-4.26215300	-2.58735900	0.53580100
O	-3.37689400	0.69743100	1.89347900
C	-4.66677000	1.10051600	1.42629700
H	-4.74228100	0.84415000	0.36283900
H	-5.45653700	0.56857000	1.98096700
H	-4.78030600	2.18905300	1.54650000
C	-3.14017700	1.06329000	3.25211600
H	-3.22560300	2.15647500	3.36557700
H	-3.86688700	0.56211800	3.91344000
H	-2.12516300	0.74251200	3.51497600
Ni	0.29667400	0.13578800	-0.10437100
P	0.59412900	-1.91276300	-1.17618700
P	1.59436300	0.52099700	-1.85436300
C	0.92072300	-0.96760400	-2.73840000
C	-0.59477500	-3.24746100	-1.53726800
C	2.16607800	-2.81722400	-0.88519100
C	1.20968200	2.00834800	-2.84292700
C	3.41064500	0.34227700	-2.01966600
H	1.55900200	-1.41956300	-3.51069900
H	-0.05153800	-0.69929300	-3.17193800
C	-1.34347400	-3.29764200	-2.72167000
C	-0.78935900	-4.23110700	-0.55247600
C	2.74001500	-2.83168400	0.39339500
C	2.81641700	-3.47955400	-1.93857800
C	0.12365900	2.05485000	-3.73070200
C	1.93227200	3.18720100	-2.58485800
C	4.17401700	-0.11663600	-0.93969400
C	4.03525500	0.58036800	-3.25647100
C	-2.26889400	-4.32529600	-2.92113000
H	-1.20831100	-2.54116800	-3.49693300
C	-1.71187200	-5.25524000	-0.75892800
H	-0.22324900	-4.18908500	0.38083400
C	3.95510500	-3.48672100	0.61287800
H	2.23754300	-2.32442800	1.21778000
C	4.03524600	-4.12172900	-1.72051900
H	2.37230000	-3.49246400	-2.93668500
C	-0.23798000	3.25825300	-4.34157500
H	-0.45955900	1.15866200	-3.94469200
C	1.56774000	4.38713200	-3.19682300
H	2.78585000	3.16672600	-1.90350400
C	5.54478500	-0.34162100	-1.09026400
H	3.69380300	-0.31179300	0.01587300
C	5.40474200	0.35863500	-3.40331300
H	3.44901200	0.93982900	-4.10526800
C	-2.45225500	-5.30535400	-1.94346400
H	-2.85170600	-4.35591500	-3.84478600
H	-1.86529900	-6.00862500	0.01664700
C	4.60874900	-4.12339900	-0.44400500
H	4.39377400	-3.48935200	1.61358900
H	4.53996400	-4.62462200	-2.54898000
C	0.47815800	4.42711500	-4.07249800
H	-1.08786600	3.28034400	-5.02744300
H	2.13541800	5.29639900	-2.98542000
C	6.16135900	-0.10369000	-2.32017100
H	6.12727200	-0.70631300	-0.24149500
H	5.88431200	0.54543800	-4.36730000
H	-3.18297400	-6.10246500	-2.09805300

H	5.56444300	-4.62575900	-0.27521200
H	0.18843700	5.36825400	-4.54536600
H	7.23359100	-0.27820600	-2.43831700
C	-1.86804800	0.21348600	-1.10531700
H	-2.75393700	0.86882600	-0.98045200
H	-1.18574300	0.83491100	-1.69687200
H	-2.18368400	-0.61502800	-1.76389100
I	-0.14117300	-1.23900900	2.41559100
**41**			
C	2.99992500	0.91683200	-0.31222900
C	2.16532600	2.08646600	-0.78941900
H	2.69247200	-0.03153400	-0.76957900
C	0.71082200	1.93887800	-0.32505000
H	2.60633800	3.02929000	-0.41147400
H	2.21009000	2.14315800	-1.88918900
C	-0.16071000	3.06972500	-0.86616300
H	0.71238800	2.02846100	0.77867100
C	-1.61952900	2.99248900	-0.38975600
H	-0.14834400	3.07147400	-1.96873300
H	0.25158200	4.05460800	-0.56347000
H	-2.03498600	2.02592500	-0.71875800
H	-1.63859500	2.98607300	0.71294800
H	3.01507100	0.81711900	0.77763900
C	-2.48418800	4.11408600	-0.90661900
C	-2.71552100	5.26804000	-0.14234800
C	-3.04508700	4.04752500	-2.19271200
C	-3.48489600	6.32226100	-0.64290900
H	-2.28615000	5.33773800	0.86138000
C	-3.81444200	5.09789000	-2.69818800
H	-2.87314200	3.15635200	-2.80323900
C	-4.03786600	6.24098400	-1.92397500
H	-3.65430500	7.21098200	-0.02902500
H	-4.24315400	5.02403400	-3.70122200
H	-4.64084600	7.06346400	-2.31707100
Ni	-0.00771600	0.10217800	-0.68097800
P	0.10022800	-0.70792800	1.45127200
P	-0.76349700	-2.04302200	-0.74625200
C	0.10279600	-2.46612500	0.84719200
C	-1.40119400	-0.62092900	2.49551600
C	1.48786600	-0.48690300	2.61453000
C	-2.54819800	-2.23621100	-0.40934400
C	-0.32226700	-3.35077900	-1.94297900
H	1.12880700	-2.74472800	0.56310900
H	-0.31043400	-3.24316500	1.50709700
C	-1.50121300	-1.32180100	3.70824200
C	-2.50651300	0.09668800	2.01814800
C	2.64338700	-1.28119600	2.55502200
C	1.45984900	0.61683100	3.48513800
C	-3.06764200	-3.07467600	0.58696400
C	-3.42194000	-1.42123700	-1.14883700
C	-1.15998400	-4.43381100	-2.24785900
C	0.92016600	-3.23253800	-2.58941800
C	-2.69800100	-1.31116300	4.42578000
H	-0.64139000	-1.87833700	4.08987900
C	-3.70429000	0.10473400	2.73749800
H	-2.42940700	0.63646100	1.07396400
C	3.74930200	-0.97930100	3.35333400
H	2.69456900	-2.13349000	1.87485900
C	2.56719400	0.91585300	4.28045500
H	0.56783600	1.24560300	3.53891100
C	-4.43989000	-3.09174200	0.84593100
H	-2.40312100	-3.70668900	1.17813600
C	-4.79444600	-1.44909700	-0.89682000
H	-3.01686100	-0.75205100	-1.91209900
C	-0.74836400	-5.39811100	-3.17141300
H	-2.13527500	-4.52385000	-1.76501500
C	1.33088700	-4.20260900	-3.50522800
H	1.55974700	-2.37058500	-2.38065000
C	-3.80170300	-0.60018500	3.93918200
H	-2.77233600	-1.85996300	5.36796600
H	-4.56271500	0.65916300	2.35186900
C	3.71506500	0.11983300	4.21568000
H	4.64412200	-1.60341100	3.29557200
H	2.53472600	1.77769000	4.95135100
C	-5.30423300	-2.27964600	0.10618700

H	-4.83428200	-3.73643300	1.63485800
H	-5.46602700	-0.80967700	-1.47471500
C	0.49697100	-5.28684400	-3.79703700
H	-1.40496100	-6.24010200	-3.40431600
H	2.29882100	-4.10448300	-4.00252800
H	-4.73891900	-0.59668700	4.50119600
H	4.58338000	0.35827500	4.83428200
H	-6.37666500	-2.28964900	0.31556600
H	0.81498300	-6.04177400	-4.52023000
I	5.13628200	1.08460200	-0.83723800
C	-0.11389100	0.46349800	-2.60123200
H	-0.12327800	-0.45939100	-3.20859800
H	-1.06198900	0.99415500	-2.81373900
H	0.70310800	1.09904300	-2.98144700
**42**			
C	1.71790200	0.18324300	2.84927900
C	0.51761700	-0.28972600	2.06485400
H	1.54405800	0.25446000	3.92969300
C	-0.59849900	0.76585900	2.11546500
H	0.80648800	-0.48297500	1.01821400
H	0.16758600	-1.24668200	2.48283900
C	-0.22516200	2.06119500	1.38732800
H	-0.81385600	0.99866300	3.17278000
C	-1.41131800	3.00069000	1.18562900
H	0.21108200	1.82142900	0.40412300
H	0.56720200	2.61201600	1.92834200
H	-2.22423900	2.43646200	0.69126300
H	-1.82753500	3.27954400	2.17142800
H	2.18615200	1.09584400	2.46402500
C	-1.14839600	4.25516500	0.38186600
C	0.13688600	4.63602900	-0.02845900
C	-2.23255300	5.06216900	-0.00677900
C	0.33298900	5.78814200	-0.79667200
H	0.99873400	4.01934100	0.22858600
C	-2.04194200	6.21079000	-0.77573800
H	-3.24450900	4.77426100	0.29560700
C	-0.75208500	6.58090100	-1.17464600
H	1.34505400	6.05744200	-1.10977600
H	-2.90194900	6.82006200	-1.06712700
H	-0.59786300	7.47913700	-1.77808700
Ni	-2.25134900	-0.04866800	1.32663300
P	-1.65348800	-0.11554300	-0.90763300
P	-4.11810500	-0.65118400	0.21119900
C	-3.44107100	-0.13438100	-1.44483300
C	-1.07048600	-1.77430100	-1.41281500
C	-0.78749400	1.06423300	-2.00212700
C	-4.70112900	-2.37359500	0.04906900
C	-5.63581100	0.33913400	0.45474300
H	-3.78198900	0.89700500	-1.61577500
H	-3.69320300	-0.73980600	-2.32806000
C	-0.98455100	-2.15355000	-2.76227700
C	-0.81649100	-2.72165400	-0.41182100
C	-1.38945500	2.28802500	-2.33869900
C	0.53673400	0.81984100	-2.40118400
C	-4.21943700	-3.23708000	-0.94738100
C	-5.56615900	-2.88402300	1.03454600
C	-6.83906500	0.05822000	-0.21225600
C	-5.55526500	1.45554500	1.30102600
C	-0.65513600	-3.46588200	-3.10030900
H	-1.17319700	-1.41957700	-3.54937800
C	-0.49013000	-4.03647100	-0.75286700
H	-0.88602100	-2.42663800	0.63670200
C	-0.68544600	3.24212700	-3.07350600
H	-2.40468500	2.51794500	-2.01038300
C	1.23843300	1.77932300	-3.13302000
H	1.02752200	-0.11989300	-2.14121700
C	-4.60279400	-4.58062200	-0.96294000
H	-3.53081300	-2.87584400	-1.71085200
C	-5.94857900	-4.22560800	1.01315000
H	-5.94698400	-2.22651700	1.81976400
C	-7.94618200	0.88949300	-0.03473600
H	-6.90955500	-0.81358000	-0.86687900
C	-6.66569400	2.28629700	1.47496400
H	-4.61913200	1.66118300	1.82465000
C	-0.41216400	-4.41008200	-2.09534200

H	-0.58337300	-3.75450200	-4.15175600
H	-0.29247900	-4.76719700	0.03435500
C	0.62989900	2.99025500	-3.47266200
H	-1.16013600	4.19630900	-3.31042800
H	2.27032400	1.57720600	-3.42775900
C	-5.46749200	-5.07884300	0.01429400
H	-4.21587400	-5.23883300	-1.74449100
H	-6.62439100	-4.60729400	1.78243700
C	-7.86054900	2.00404400	0.80784100
H	-8.88129100	0.66751600	-0.55503800
H	-6.59761100	3.15150000	2.13887500
H	-0.14985700	-5.43634500	-2.36342000
H	1.18478700	3.74526500	-4.03452700
H	-5.76543400	-6.13002800	0.00031400
H	-8.73036500	2.65093900	0.94649300
I	3.42491300	-1.27735400	2.73902000
Mg	4.04280900	-0.52028100	-0.00910500
I	3.02290700	-2.33593300	-1.66820100
I	3.75625500	2.14615800	-0.01955500
O	6.07002400	-0.83014800	0.12561000
C	6.82674300	-1.37900800	-0.95919400
H	7.23792700	-0.56990100	-1.58551300
H	7.64771100	-1.99786400	-0.56264800
H	6.14705700	-2.00289300	-1.55381900
C	6.84939600	-0.02187400	1.01387400
H	7.70258800	-0.60779400	1.39211400
H	7.20872300	0.88067800	0.49435400
H	6.20689200	0.27218900	1.85338900
C	-2.95736700	-0.29993900	3.13408900
H	-3.90797700	-0.86314800	3.16725300
H	-2.22016900	-0.86166900	3.73813100
H	-3.12576600	0.66405000	3.64882200

TS43

C	-1.51492100	-0.07897200	-2.79048500
C	-0.60902900	-0.56906800	-1.77476100
H	-1.33782500	-0.34706200	-3.83302300
C	0.40735900	0.58170400	-2.05186500
H	-0.98176300	-0.50824300	-0.74519500
H	-0.21699800	-1.57209900	-1.97962600
C	0.10282100	1.92386400	-1.39092800
H	0.65578800	0.68861900	-3.11749900
C	1.33969700	2.81737100	-1.31177900
H	-0.28069600	1.74996100	-0.37507300
H	-0.69541700	2.47689800	-1.91322800
H	2.17295100	2.22930000	-0.88514000
H	1.67291200	3.07357500	-2.33463800
H	-2.08260100	0.83465300	-2.60951400
C	1.19338800	4.08441400	-0.49875000
C	-0.04590900	4.55013100	-0.03866100
C	2.34514000	4.81797800	-0.16341100
C	-0.13085300	5.71718700	0.72758700
H	-0.95864900	3.99440300	-0.25753400
C	2.26402400	5.98135200	0.60214000
H	3.32184700	4.46150300	-0.50622900
C	1.01970600	6.43767300	1.05206200
H	-1.10825400	6.05492700	1.08135500
H	3.17402100	6.53370000	0.85195100
H	0.95102800	7.34697400	1.65462600
Ni	2.14447200	-0.21271900	-1.27699900
P	1.72596200	-0.00586100	1.00300700
P	4.07382700	-0.64085800	-0.28773900
C	3.55658700	0.07106400	1.34815500
C	1.22378200	-1.61240600	1.71031500
C	0.92268000	1.26446400	2.03625600
C	4.68033500	-2.33433600	-0.00085100
C	5.53059200	0.32011600	-0.82169300
H	3.88377000	1.12090300	1.33769800
H	3.91954000	-0.40498800	2.27094100
C	1.10413500	-1.81322200	3.09430400
C	1.02396400	-2.68749700	0.83291600
C	1.60894800	2.40950800	2.47067800
C	-0.45076100	1.14059400	2.30766200
C	4.37195400	-3.05230300	1.16472800
C	5.39006800	-2.97275800	-1.03452600
C	6.81078100	0.09057800	-0.29295900

C	5.33145100	1.36407600	-1.73775700
C	0.79264100	-3.07964000	3.58931700
H	1.24413000	-0.97637800	3.78243300
C	0.71581000	-3.95436100	1.33180600
H	1.11501900	-2.52812100	-0.24360400
C	0.93456500	3.40715000	3.17667000
H	2.67010000	2.54098600	2.25481300
C	-1.11977500	2.14201600	3.01131100
H	-1.00622700	0.26172600	1.97292300
C	4.77266800	-4.38427400	1.29607300
H	3.80741000	-2.58677300	1.97203600
C	5.78934000	-4.30219100	-0.89699500
H	5.63748100	-2.42562900	-1.94729800
C	7.87807600	0.90493600	-0.67515400
H	6.97253600	-0.72642000	0.41394700
C	6.40233900	2.17858100	-2.11469100
H	4.33574400	1.52600500	-2.15504200
C	0.60046600	-4.15104600	2.70894500
H	0.69060100	-3.23172600	4.66649900
H	0.55470300	-4.78460900	0.64110500
C	-0.42931800	3.27577400	3.44837700
H	1.47601000	4.29934600	3.49695800
H	-2.18902800	2.03494500	3.20291200
C	5.48022400	-5.01192600	0.26834900
H	4.52255600	-4.93221200	2.20744100
H	6.34263300	-4.78711100	-1.70477000
C	7.67466900	1.94973800	-1.58387800
H	8.87372000	0.72419300	-0.26277200
H	6.24277800	2.98865700	-2.83025700
H	0.34633300	-5.13893500	3.10036100
H	-0.95729000	4.06474000	3.98909300
H	5.79025900	-6.05436900	0.37314300
H	8.51317800	2.58396900	-1.88178900
I	-3.85686700	-1.54738500	-2.78682400
Mg	-4.08724200	-0.60535700	-0.17822500
I	-2.81189900	-2.11998500	1.65014700
I	-3.70927500	2.08790900	-0.10604500
O	-6.10216700	-0.70251300	0.22236400
C	-6.53222500	-0.60936600	1.58379900
H	-6.60405800	0.44757600	1.89020600
H	-7.51135300	-1.10189000	1.69842800
H	-5.78584800	-1.12607500	2.20126800
C	-7.02358600	-0.11959800	-0.70400000
H	-7.99455200	-0.63645000	-0.63455700
H	-7.15182000	0.95512300	-0.49493300
H	-6.61052200	-0.25140600	-1.71261900
C	2.74338400	-0.71981700	-3.05865800
H	3.67407700	-1.31175800	-3.08397000
H	1.94591600	-1.33625200	-3.51381900
H	2.89292800	0.16362100	-3.70494300
**2**			
C	0.46418700	-0.20084200	-0.85913500
C	1.42135100	-0.36087900	0.33437100
H	0.57826000	-1.07083000	-1.52765300
H	0.76604800	0.68514200	-1.44164700
C	2.87198800	-0.47485100	-0.08058200
H	1.30002200	0.50095300	1.01349500
H	1.12584100	-1.25146000	0.91708000
C	3.68433000	0.76935900	-0.33635500
C	3.97212800	-0.08758300	0.87153600
H	3.08536000	-1.32579400	-0.73849200
H	4.40446000	0.76727800	-1.15958800
H	3.19493600	1.73475000	-0.17119800
H	4.88942300	-0.68338900	0.87868000
H	3.68050000	0.29758100	1.85376200
C	-0.98023400	-0.06808800	-0.44091900
C	-1.54861600	1.19363800	-0.20683700
C	-1.77473200	-1.20496800	-0.22412400
C	-2.87094500	1.31829200	0.22736100
H	-0.94500200	2.09089500	-0.37226000
C	-3.09708600	-1.08606200	0.21074000
H	-1.34989500	-2.19703500	-0.40321900
C	-3.65053900	0.17743700	0.43842700
H	-3.29541500	2.31101000	0.39881300
H	-3.69980700	-1.98414800	0.36962600

H	-4.68572900	0.27233200	0.77570300

44

Mg	-2.66369500	-0.56360800	-0.84603900
I	-4.69293700	0.63069800	0.47303600
O	-3.51566500	-1.42760700	-2.51679200
C	-2.67671400	-1.97921200	-3.53418100
H	-1.73315500	-1.41893500	-3.53090000
H	-2.47511500	-3.04390200	-3.33276100
H	-3.16407500	-1.86656100	-4.51659700
C	-4.79026200	-2.06901700	-2.42643300
H	-5.30951300	-2.00381900	-3.39652100
H	-4.66717900	-3.12653700	-2.13799100
H	-5.37356500	-1.53974600	-1.66191800
C	1.70412500	-0.72952300	-2.53085200
H	1.87145100	0.00105200	-3.34181900
H	2.60910900	-1.35406600	-2.45038500
H	0.85797000	-1.38164600	-2.80578900
I	-0.99336600	1.29503300	-2.11470200
Ni	1.15660100	0.25262000	-0.94664000
P	0.94485600	1.26872600	1.09450200
P	3.03414400	-0.13218600	0.00781300
C	2.77303500	1.05315700	1.41582800
C	0.05999000	0.32742800	2.37266400
C	0.59116200	3.03163900	1.39234700
C	3.34166000	-1.75536900	0.76390600
C	4.55861000	0.35774400	-0.86377200
H	3.27230600	2.00721200	1.19114700
H	3.07751500	0.72106300	2.41923900
C	0.66617700	-0.72744600	3.07132900
C	-1.31529500	0.56515000	2.53688500
C	0.62623700	3.90444900	0.29378500
C	0.34584700	3.53992600	2.67790300
C	4.31407200	-1.90740700	1.76834700
C	2.54026500	-2.84317400	0.39588300
C	4.62339900	1.65824200	-1.39373400
C	5.61178100	-0.53731000	-1.09634400
C	-0.09193200	-1.52097000	3.93457500
H	1.72331100	-0.95521400	2.93210600
C	-2.06711200	-0.23195600	3.39964300
H	-1.81052000	1.36456100	1.98094300
C	0.42692600	5.27429100	0.48080000
H	0.79120400	3.50608900	-0.70942700
C	0.14197100	4.90889900	2.85856100
H	0.30656300	2.86428900	3.53500700
C	4.47781700	-3.14196000	2.39605800
H	4.93930700	-1.06004300	2.06199000
C	2.70649800	-4.07712200	1.03072400
H	1.76606200	-2.71720500	-0.36161200
C	5.74283700	2.06475600	-2.11854800
H	3.79151600	2.35286900	-1.24794300
C	6.72897500	-0.12674700	-1.83007500
H	5.56221300	-1.55562700	-0.70771700
C	-1.45689300	-1.27555000	4.10111100
H	0.38825900	-2.34496800	4.46750100
H	-3.13799400	-0.04497400	3.50064700
C	0.18376100	5.77668900	1.76181800
H	0.44858600	5.94789000	-0.37911300
H	-0.05342400	5.30055500	3.85972900
C	3.67133800	-4.22685400	2.02871300
H	5.23154400	-3.25882000	3.17849500
H	2.06464300	-4.91538500	0.75135100
C	6.79860700	1.17229000	-2.33739400
H	5.78842400	3.07848900	-2.52326500
H	7.54656800	-0.82968700	-2.00669500
H	-2.04965200	-1.90660700	4.76736900
H	0.01844200	6.84711800	1.90663000
H	3.79525100	-5.19032100	2.52922900
H	7.67264900	1.48990200	-2.91091300
I	-1.24105900	-2.67694900	0.05488700

TS45

C	1.46025000	0.63994700	0.37793400
C	1.23878300	1.59819000	-0.75125100
H	0.93613300	-0.31566300	0.37123400
C	-0.02409500	1.82793300	-0.03835200
H	1.95080100	2.43154400	-0.76556900

H	1.15347400	1.11251500	-1.73092200
C	-1.30409600	1.14885000	-0.38514700
H	-0.04801900	2.60545300	0.73173000
C	-2.14981800	0.72395600	0.83105500
H	-1.11157400	0.27942600	-1.03558700
H	-1.90397700	1.85134900	-0.99977600
H	-1.56502800	0.01594600	1.43994600
H	-2.33537500	1.60836300	1.46258000
H	1.74741600	1.02731500	1.35505200
C	-3.46228500	0.09475200	0.43027700
C	-4.59585700	0.88734800	0.19253300
C	-3.56380200	-1.29109000	0.23537400
C	-5.79795700	0.31239900	-0.22721500
H	-4.53633800	1.96916600	0.34411100
C	-4.76412100	-1.87059900	-0.18411700
H	-2.69115200	-1.92442100	0.41963500
C	-5.88560800	-1.06977400	-0.41776500
H	-6.67132800	0.94575700	-0.40266900
H	-4.82443100	-2.95262400	-0.32622200
H	-6.82584500	-1.52154500	-0.74332300
I	3.77013000	-0.47441600	-0.00943700

I·

I	0.00000000	0.00000000	0.00000000

TS46

C	-3.31493000	-0.95218600	-0.47632900
C	-3.17874100	-2.05254900	-1.52207600
H	-2.44729600	-0.27856000	-0.48930300
C	-2.00842200	-2.90732200	-1.15684600
H	-4.11411400	-2.64412300	-1.54807500
H	-3.04215700	-1.60647700	-2.51841000
C	-0.81411700	-3.12524200	-2.02239300
H	-2.06074900	-3.44917600	-0.20461100
C	0.49181300	-3.22221200	-1.20664200
H	-0.73012800	-2.30196600	-2.74718800
H	-0.92304100	-4.05061200	-2.62836500
H	0.47411000	-2.39919800	-0.47648500
H	0.50391300	-4.16112300	-0.62952800
H	-3.47536800	-1.34431900	0.53340900
C	1.74612300	-3.09463600	-2.03209400
C	2.53543900	-4.20406800	-2.36528000
C	2.14773900	-1.82717500	-2.48922800
C	3.69592700	-4.05481800	-3.13189900
H	2.23997000	-5.19697200	-2.01421600
C	3.30479800	-1.67401800	-3.25372200
H	1.53727800	-0.95247900	-2.23298200
C	4.08509800	-2.78923800	-3.57839500
H	4.29959500	-4.93202900	-3.37940200
H	3.59882500	-0.67965700	-3.59984300
H	4.99207700	-2.67158800	-4.17681500
Ni	-0.14676500	0.12269900	-0.90435100
P	-0.00001900	-0.27144700	1.32611300
P	1.09686900	1.86910100	-0.03730700
C	0.24679000	1.55295800	1.59286300
C	1.50736500	-1.00170000	2.07582300
C	-1.36887400	-0.82369800	2.40414500
C	2.88535300	1.70428600	0.30758800
C	0.82714900	3.64397200	-0.39404900
H	-0.72681000	2.06074400	1.53034900
H	0.74983400	1.86039800	2.52191100
C	1.70108700	-1.07139300	3.46481700
C	2.53384200	-1.41683000	1.21545100
C	-2.41807400	0.03869600	2.75639000
C	-1.46697700	-2.18733000	2.73237800
C	3.46203600	1.87341900	1.57501300
C	3.69669400	1.29075100	-0.76211400
C	1.78170100	4.64232300	-0.14940000
C	-0.40991100	3.99318000	-0.96310100
C	2.90761100	-1.54853900	3.98022000
H	0.90711600	-0.75147100	4.14406400
C	3.74048600	-1.89274700	1.73228100
H	2.39460100	-1.34686000	0.13737800
C	-3.54454200	-0.45215400	3.42142400
H	-2.37575600	1.09769800	2.49568500
C	-2.59272100	-2.67484100	3.39801400

H	-0.66091900	-2.87245600	2.45883600
C	4.82248100	1.62547000	1.76975100
H	2.84890800	2.18200300	2.42331700
C	5.05790200	1.05186900	-0.56926900
H	3.24856500	1.13360900	-1.74614700
C	1.49340400	5.97580100	-0.45212600
H	2.75172900	4.37849900	0.27696300
C	-0.69712500	5.32769400	-1.25457200
H	-1.14023300	3.21084200	-1.18774600
C	3.92853700	-1.95896000	3.11470400
H	3.05414500	-1.59931300	5.06213800
H	4.53247200	-2.20561600	1.04857500
C	-3.63694600	-1.80918900	3.74121200
H	-4.35708500	0.22994800	3.68202000
H	-2.65725100	-3.73712400	3.64627700
C	5.62217300	1.21335400	0.69984300
H	5.25713000	1.74477500	2.76504600
H	5.67493300	0.72046600	-1.40789900
C	0.25409000	6.32113700	-0.99942200
H	2.24143900	6.74902900	-0.25928200
H	-1.66188300	5.59089100	-1.69506300
H	4.87238300	-2.33005200	3.52193400
H	-4.52107600	-2.19267300	4.25584600
H	6.68430900	1.01020000	0.85701400
H	0.03221300	7.36484300	-1.23557200
I	-5.03110200	0.38479300	-0.80804200
C	-0.82289300	0.57243100	-2.71113500
H	-1.78653900	1.12139900	-2.68367100
H	-0.12113800	1.23230200	-3.25921000
H	-0.99709700	-0.29721500	-3.37592100
**47**			
Ni	-0.04965100	-1.02462500	1.95016300
P	-1.33111900	-0.37664600	0.12836200
P	1.42019700	-0.48999500	0.29385300
C	0.10472700	-0.78536900	-0.98901400
C	-1.73198400	1.37913300	-0.18423700
C	-2.75392100	-1.32073900	-0.53019400
C	1.74294400	1.31118800	0.13148500
C	2.95931500	-1.25402200	-0.33618500
H	0.04717100	-1.86874100	-1.17640600
H	0.19380100	-0.25896400	-1.95088400
C	-1.31546600	2.09854600	-1.31343100
C	-2.43768500	2.04830900	0.83064500
C	-2.95247300	-2.60659600	-0.00135800
C	-3.63232600	-0.82915700	-1.50716000
C	2.43782400	1.86732300	-0.95289800
C	1.18419200	2.15599000	1.10142200
C	3.07771300	-1.79771900	-1.62386300
C	4.06570700	-1.29634100	0.52926300
C	-1.59431900	3.46291400	-1.42344500
H	-0.75567100	1.60423500	-2.10847000
C	-2.72777200	3.40814700	0.71301300
H	-2.74417900	1.49818900	1.72468900
C	-4.00395000	-3.40109900	-0.46307500
H	-2.28247700	-2.96810200	0.78362800
C	-4.68757200	-1.62413000	-1.96094400
H	-3.49445600	0.17696800	-1.90944900
C	2.55462700	3.25349700	-1.07165200
H	2.88648400	1.21572200	-1.70695500
C	1.29766000	3.54223600	0.97874100
H	0.64268800	1.71284800	1.94102200
C	4.28408500	-2.36997800	-2.03985900
H	2.22967000	-1.77605000	-2.31137700
C	5.27065500	-1.86020800	0.10961600
H	3.97834100	-0.88493500	1.53901500
C	-2.30071500	4.12002000	-0.41263900
H	-1.25122600	4.01557600	-2.30130000
H	-3.27619200	3.91736100	1.50934100
C	-4.87236500	-2.91047800	-1.44320400
H	-4.15320700	-4.40062200	-0.04726700
H	-5.37088000	-1.23706200	-2.72114300
C	1.98046100	4.09197700	-0.10908600
H	3.09446300	3.68330600	-1.91931500
H	0.84565400	4.19264000	1.73098900
C	5.38192400	-2.39986900	-1.17662200

H	4.36540600	-2.79101400	-3.04512400
H	6.12511200	-1.88508700	0.79041800
H	-2.51333400	5.18844700	-0.49851700
H	-5.70062400	-3.52846800	-1.79885000
H	2.06868400	5.17675100	-0.20813200
H	6.32398900	-2.84665600	-1.50358500
C	-0.95540100	-1.72433700	3.56007600
H	-1.58507200	-2.61867700	3.37912600
H	-0.24495000	-2.01309100	4.36238000
H	-1.63044800	-0.97088200	4.01600400

S1

C	-7.06435000	-2.12271600	-0.64485100
C	-5.68978200	-1.90646200	-0.52915700
C	-4.88407300	-2.79102000	0.20594900
C	-5.49018600	-3.89387500	0.82418000
C	-6.86614700	-4.11360200	0.70973900
C	-7.65769700	-3.22770100	-0.02545600
H	-7.67558600	-1.42520500	-1.22320900
H	-5.23276000	-1.03860900	-1.01310800
H	-4.87496200	-4.59079300	1.40086500
H	-7.32039100	-4.98091000	1.19588100
H	-8.73328800	-3.39800900	-0.11757000
C	-3.40570900	-2.52586800	0.36282100
C	-3.12088500	-1.43319300	1.39927300
H	-2.98166000	-2.21170100	-0.60583300
H	-2.89677700	-3.45569000	0.65829200
C	-1.65604700	-1.05288900	1.51472800
H	-3.66403800	-0.51859200	1.11607300
H	-3.49529300	-1.73730400	2.39048800
C	-1.43858800	0.15084100	2.42049200
H	-1.23768500	-0.84873500	0.51655000
H	-2.07099400	0.95196200	2.00519300
H	-1.82174200	-0.06226300	3.43048600
C	-0.00019100	0.63981300	2.48750500
H	0.47637600	0.67862900	1.50251000
H	0.62998100	0.02918900	3.14348400
O	0.07596000	1.96719600	3.08479800
S	-0.27486800	3.25982900	2.19142900
O	-1.52773600	3.85130200	2.61934000
O	-0.15402000	2.85733200	0.75751800
C	1.10678000	4.30020100	2.56236500
H	2.01735900	3.77707500	2.23774800
H	0.95453200	5.23395300	2.00310500
H	1.09846800	4.48690100	3.64440600
O	-0.94082200	-2.22796600	2.07126400
S	0.41055200	-2.77964900	1.41338400
O	0.98506600	-3.71308500	2.36114600
O	1.21708600	-1.57601400	1.05576200
C	-0.06279600	-3.58546400	-0.09172200
H	-0.68684400	-4.44702600	0.17553300
H	-0.58684700	-2.86713200	-0.73450700
H	0.87725400	-3.89665400	-0.57324300
Mg	2.79095300	-0.91192500	-0.01311800
I	3.43124700	1.72088500	0.48651100
O	4.07435900	-1.58967300	1.52073200
C	5.47728800	-1.63878600	1.25471700
H	5.94090000	-2.43990500	1.85355200
H	5.60446700	-1.86787800	0.18885600
H	5.95222000	-0.67204100	1.49178700
C	3.77856700	-1.33584700	2.89154400
H	4.07132900	-0.30892900	3.16787200
H	2.70038800	-1.46696400	3.02960100
H	4.30940700	-2.06201100	3.52850800
Mg	-0.95434400	2.19860700	-0.94285700
I	-3.61407000	2.05210500	-0.71942600
I	0.30942800	0.01914300	-1.95034600
O	-0.31960400	3.72375400	-2.11964400
C	1.08831400	3.92502800	-2.29920200
H	1.30629200	5.00513300	-2.32381700
H	1.61164900	3.46067400	-1.45222700
H	1.42570900	3.45099000	-3.23480400
C	-1.12693500	4.28508200	-3.15896500
H	-2.17741900	4.09536200	-2.90114300
H	-0.95181300	5.37128900	-3.22101400
H	-0.88827800	3.81204400	-4.12573200

I	3.43758500	-2.80277400	-1.84449400
**S2**			
C	6.70893400	-1.24361400	-0.12093900
C	5.35780100	-0.97348200	0.11187700
C	4.66196600	-0.04273900	-0.67520500
C	5.35828600	0.61161600	-1.70370900
C	6.70911600	0.34510600	-1.94037000
C	7.38993700	-0.58439800	-1.14842100
H	7.23152900	-1.97501700	0.50103400
H	4.82961500	-1.49503600	0.91537400
H	4.83127800	1.33791200	-2.32973100
H	7.23201100	0.86256700	-2.74899800
H	8.44582400	-0.79664300	-1.33383600
C	3.21553400	0.27954300	-0.39315600
C	3.02403800	1.46840000	0.56068700
H	2.71702800	-0.60027500	0.04386900
H	2.69349000	0.50066600	-1.33909700
C	1.54538600	1.74551400	0.81782700
H	3.53783800	1.25320700	1.51381100
H	3.51575000	2.36335400	0.14002100
C	1.28394000	2.89279300	1.79342300
H	1.05854700	1.96046800	-0.14730500
H	1.83675500	2.70979800	2.73221500
H	1.66392700	3.84639400	1.38840300
O	-1.01161200	3.55526700	1.09709400
S	-1.68023600	2.56978400	0.02996000
O	-1.53457100	1.18660300	0.56830700
O	-1.15314200	2.83804800	-1.29944100
C	-3.38941800	3.01953800	0.12596600
H	-3.73094700	2.85810200	1.15636400
H	-3.47807000	4.07162900	-0.17531700
H	-3.91766800	2.35356800	-0.57274300
Mg	-1.45213800	-0.75250700	0.03690800
I	-3.74093100	-0.95816100	-1.38014200
O	0.00232500	-0.85684700	-1.42158400
C	0.70447100	-2.08975100	-1.62332900
H	0.82751200	-2.56942700	-0.64396300
H	0.12798000	-2.74897200	-2.29370600
H	1.69361700	-1.88728600	-2.06204700
C	-0.13701400	-0.09259500	-2.62552700
H	-0.60535100	0.86490600	-2.37087200
H	0.85900900	0.09316900	-3.05818900
H	-0.76678000	-0.63285900	-3.35012000
I	-0.41965200	-1.97511800	2.18282300
C	-0.16577200	3.06567700	2.19935900
H	-0.60080700	2.13152500	2.58234500
H	-0.28448300	3.85345000	2.95463700
H	1.07551100	0.82789300	1.20764200
**Me$_2$O**			
O	0.00000000	0.58215700	-0.00001800
C	1.16781500	-0.18974600	-0.00000100
H	1.23990600	-0.84213000	-0.89580200
H	2.03079300	0.49404000	-0.00003800
H	1.23992700	-0.84206100	0.89584800
C	-1.16781500	-0.18974600	0.00001300
H	-2.03079300	0.49404000	-0.00000300
H	-1.23992200	-0.84212300	-0.89579100
H	-1.23991100	-0.84206800	0.89585800
**TS-S3**			
C	1.16956800	7.12666100	-1.89092500
C	1.57439900	5.80376400	-1.68692400
C	0.74095000	4.88732500	-1.02995900
C	-0.51243000	5.33337100	-0.57630900
C	-0.92139000	6.65289700	-0.77892500
C	-0.08043200	7.55564400	-1.43825800
H	1.83471200	7.82449600	-2.40621700
H	2.55353800	5.47355400	-2.04519400
H	-1.17518800	4.63769400	-0.05396100
H	-1.90117000	6.97892000	-0.42032500
H	-0.39923000	8.58873000	-1.59761800
C	1.17884500	3.46261500	-0.79045200
C	1.44011700	3.13442500	0.68550700
H	2.08550700	3.24858400	-1.37886100
H	0.40056000	2.77372000	-1.16146700
C	1.71880100	1.65074900	0.88945200

H	2.28697400	3.74004400	1.05255100
H	0.56322800	3.41695300	1.29060100
C	2.00093600	1.26575400	2.33614600
H	0.84616300	1.07809400	0.54037000
H	2.95423300	1.68382300	2.69749600
H	1.22875500	1.69402700	2.99797100
O	-0.01455200	-0.44990300	3.29979800
S	-1.22652300	0.04096800	2.55507300
O	-1.41005900	-0.78049500	1.27820300
O	-1.24521700	1.48954200	2.28018000
C	-2.59576800	-0.36590000	3.62001700
H	-2.57479800	-1.44604000	3.81342600
H	-2.45915900	0.20438400	4.54824800
H	-3.52368000	-0.07818500	3.10565900
Mg	-2.74519900	-0.98410300	-0.15763400
I	-5.23807500	-0.32180500	0.65717800
O	-2.26901000	0.62546300	-1.36855100
C	-2.53493900	0.57681100	-2.77346200
H	-2.41594700	-0.46346900	-3.10268200
H	-3.56503500	0.91219200	-2.97883400
H	-1.82059800	1.22440600	-3.30808400
C	-2.40997300	1.94546900	-0.82126200
H	-2.08272200	1.91432900	0.22588300
H	-1.78077300	2.64435300	-1.39275100
H	-3.46197400	2.26790000	-0.87499800
I	-2.20804900	-3.18719400	-1.63637500
I	4.51790300	-0.96213300	1.70443400
Mg	3.86639000	-0.62876700	-1.00365500
O	4.47206400	-2.41577700	-1.85949600
O	1.89619100	-1.21842100	-1.03121400
C	4.31014800	1.03928200	-2.21993100
C	4.84980900	-3.63851900	-1.22750000
C	4.08668800	-2.57006200	-3.22683700
C	0.91551200	-0.56049800	-1.84046700
C	1.40967300	-2.39275800	-0.37109600
H	5.25062300	0.88213300	-2.78400800
H	3.54021700	1.28244100	-2.97724500
H	4.46569200	1.96267600	-1.63067100
H	4.01345600	-4.35769700	-1.24729000
H	5.72109800	-4.07576600	-1.74244200
H	5.11412000	-3.40831000	-0.18772700
H	3.86665800	-1.57041500	-3.62672400
H	4.91226700	-3.01514700	-3.80575700
H	3.19186200	-3.21002300	-3.30711200
H	0.09446000	-0.18529700	-1.21399700
H	1.41384000	0.27871900	-2.34126300
H	0.51107600	-1.26071800	-2.58784000
H	2.21299300	-2.76375300	0.27875700
H	0.52235900	-2.14862800	0.23277200
H	1.13167600	-3.16049400	-1.10919600
C	1.97605800	-0.19803700	2.58702600
H	1.65023300	-0.90426000	1.83341000
H	2.25304900	-0.60343700	3.55724300
H	2.57193800	1.35122400	0.25816200

S4

C	7.57229300	0.44669100	1.75641800
C	6.19122300	0.52259300	1.55948600
C	5.57539900	-0.15959400	0.49887300
C	6.38114300	-0.92026600	-0.36187000
C	7.76289100	-0.99885100	-0.16946000
C	8.36352200	-0.31435300	0.89098800
H	8.03258500	0.98115000	2.59144800
H	5.57811500	1.11683000	2.24350500
H	5.91620800	-1.46217800	-1.19060000
H	8.37282400	-1.60074500	-0.84805800
H	9.44355300	-0.37714400	1.04515900
C	4.08995500	-0.03830000	0.25799600
C	3.74486400	1.16336200	-0.63190400
H	3.56185600	0.06211500	1.22055100
H	3.71131400	-0.95875000	-0.20855000
C	2.26067000	1.36759300	-0.88237100
H	4.12327300	2.08656900	-0.16303800
H	4.26023600	1.07448700	-1.60271600
C	1.93762500	2.60326000	-1.70827100
H	1.70600800	1.39088000	0.06872600

H	2.36348700	3.46281900	-1.16390700
H	2.49109000	2.53432500	-2.66059800
O	1.76691400	0.18896500	-1.65108400
S	0.68927100	-0.79191000	-1.00319400
O	-0.41232700	0.06962900	-0.47418800
O	1.28611000	-1.70922700	-0.04047100
C	0.14204300	-1.64069700	-2.45490000
H	-0.25011400	-0.90312300	-3.16597200
H	1.00373200	-2.18650500	-2.86088400
H	-0.65193100	-2.32545600	-2.11963900
Mg	-2.23831400	-0.00642700	0.36458500
I	-3.17014800	-2.42925500	-0.39122100
O	-1.70243000	-0.30878600	2.32573200
C	-2.59724400	0.08125400	3.37341100
H	-3.14396400	0.96885200	3.02875000
H	-3.30562300	-0.73408700	3.59617300
H	-2.02108500	0.33265700	4.27846000
C	-0.87726700	-1.42740100	2.67064000
H	-0.16227400	-1.58765900	1.85482700
H	-0.32581400	-1.20423000	3.59842500
H	-1.49248200	-2.33145200	2.80540200
I	-3.37842700	2.40402000	0.18669900
C	0.45247900	2.83530900	-1.96547900
H	0.00660000	2.00817600	-2.53760300
H	-0.11767500	2.93116300	-1.02905200
H	0.30224400	3.75820200	-2.54629900
**TS-S5**			
C	-6.85215600	3.76474700	0.24372100
C	-5.88764400	2.75402900	0.21267200
C	-4.51964300	3.06519300	0.21663400
C	-4.14027300	4.41583500	0.25014600
C	-5.10123800	5.42941600	0.28050500
C	-6.46165300	5.10649300	0.27704400
H	-7.91353800	3.50328200	0.24493300
H	-6.19762500	1.70537700	0.18914100
H	-3.07667600	4.67159000	0.25603800
H	-4.78719200	6.47598000	0.31079900
H	-7.21475800	5.89798200	0.30360000
C	-3.47869700	1.97774300	0.13221800
C	-3.07500200	1.67899300	-1.31507600
H	-3.86014100	1.05416100	0.59444300
H	-2.57420000	2.26554400	0.68735200
C	-2.00174700	0.66261400	-1.44848400
H	-3.94174600	1.37349300	-1.92320900
H	-2.69296100	2.59262500	-1.80319600
C	-1.39775200	0.30358200	-2.75394100
H	-1.58142000	0.23325300	-0.53991900
H	-2.20562200	0.09366000	-3.47508200
H	-0.94024600	1.24556200	-3.10871800
O	-0.40887000	2.24516700	-0.97151900
S	0.55857700	1.99768400	0.14117700
O	1.63941600	1.03141900	-0.31578900
O	-0.06704000	1.55255900	1.40576700
C	1.38497900	3.55280900	0.42903500
H	1.85332100	3.87511500	-0.50964600
H	0.62397800	4.27123000	0.76162500
H	2.15163200	3.40310700	1.20057700
Mg	3.41055900	0.26302100	-0.06068900
I	5.24585200	2.22955100	0.18363600
O	3.31810400	-0.36364200	1.92367100
C	4.41049500	-1.16250900	2.38828600
H	4.69516200	-1.83855600	1.57096000
H	5.26736200	-0.52485200	2.66149800
H	4.09127900	-1.75579800	3.26074200
C	2.81575100	0.53822900	2.91550000
H	1.89943200	0.99579500	2.52415300
H	2.57094300	-0.02169700	3.83267700
H	3.56525600	1.31487900	3.14037600
I	3.59556400	-2.02151200	-1.49001500
C	-0.35167100	-0.80113400	-2.70060200
H	0.43419600	-0.55400100	-1.97301400
H	-0.80537400	-1.76135800	-2.41575100
H	0.13053800	-0.92892300	-3.68064400
I	-3.73529900	-1.70851400	-1.10976000
Mg	-2.46943800	-2.02429800	1.35271300

O	-2.87478100	-4.00589900	1.81950900
O	-0.56021500	-2.38926400	0.69282500
C	-2.49309700	-0.75530500	3.03296500
C	-3.40306900	-5.04181800	0.99199800
C	-2.42015900	-4.46600200	3.09311300
C	0.55680100	-1.79193700	1.36799600
C	-0.21884800	-3.54663000	-0.07071200
H	-3.48985000	-0.66652500	3.50594900
H	-1.80113700	-1.09024700	3.83093000
H	-2.17392000	0.27000900	2.77206700
H	-2.62772500	-5.79737700	0.78050700
H	-4.25880400	-5.52562800	1.49160700
H	-3.74060900	-4.58293300	0.05425700
H	-2.06875800	-3.59158300	3.65640800
H	-3.24799300	-4.94275000	3.64348900
H	-1.59564000	-5.18852300	2.96991200
H	1.39152200	-1.70044100	0.66270900
H	0.24406300	-0.79945400	1.71093300
H	0.86640800	-2.41846600	2.22213700
H	-1.10267300	-3.83561500	-0.65346800
H	0.61222500	-3.31485100	-0.75401200
H	0.08122500	-4.37432900	0.59527600
**S6**			
C	-5.74050800	4.66210700	0.99791200
C	-4.71044300	3.74840800	0.75809200
C	-3.36736200	4.12935800	0.89936700
C	-3.07974400	5.44820800	1.28465500
C	-4.10735500	6.36376400	1.52456900
C	-5.44237000	5.97303000	1.38112900
H	-6.78141800	4.34727100	0.88737300
H	-4.94678900	2.72277100	0.46098700
H	-2.03599400	5.75441700	1.40038600
H	-3.86570000	7.38584600	1.82818500
H	-6.24760100	6.68735600	1.57051400
C	-2.25187600	3.16075400	0.59272100
C	-1.81198700	3.24706500	-0.87748500
H	-2.59050100	2.13240400	0.78883900
H	-1.39867600	3.36715300	1.25113300
C	-0.63864900	2.34545800	-1.25412800
H	-2.66842700	2.96463700	-1.50792300
H	-1.55281900	4.28352700	-1.14720600
C	-0.65502300	1.92515500	-2.71853800
H	-0.58926300	1.46281800	-0.60477800
H	-1.65041100	1.51313300	-2.94182100
H	-0.53069300	2.82798600	-3.33922200
C	0.39270200	0.91838800	-3.15099700
H	1.38405700	1.10664800	-2.71538900
H	0.48757100	0.89636700	-4.24339400
O	-0.03848200	-0.45601800	-2.82464400
S	0.57834800	-1.26424700	-1.60527800
O	-0.47580500	-2.19444500	-1.16375000
O	1.06063700	-0.29086400	-0.60615500
C	1.89200900	-2.22991000	-2.27760900
H	2.65647500	-1.54019500	-2.66253700
H	2.28922300	-2.82710400	-1.44285700
H	1.45295600	-2.86325000	-3.05990500
O	0.65155800	3.07304400	-1.10087500
S	1.39359300	3.25726500	0.30628900
O	1.63891500	1.90045000	0.88081700
O	0.70930500	4.18114200	1.19772400
C	2.90845200	3.96831500	-0.27044000
H	3.40310300	3.26266900	-0.95300600
H	2.65267900	4.91676900	-0.76065500
H	3.51905600	4.14207200	0.62612800
I	-3.65966400	-0.24294200	-0.88495400
Mg	-2.17472000	-2.33022900	-0.03591500
Mg	2.76668100	0.23238700	0.53645700
I	4.31641800	0.70505200	-1.71862300
O	4.06939900	1.02218700	1.98977500
C	5.41686600	0.53984500	2.04041700
H	5.75349800	0.38352600	1.00795300
H	6.05895800	1.29309500	2.52569300
H	5.46673600	-0.41032400	2.59619200
C	3.53523100	1.29010500	3.28847400
H	2.54187900	1.73590600	3.15666800

H	3.45246300	0.35820000	3.87182500
H	4.18624200	2.00392500	3.81938500
I	2.70652900	-2.24876700	1.70962100
O	-1.38164100	-1.75551500	1.76087600
C	-1.24763900	-2.62789200	2.89164200
H	-1.56440700	-3.63034500	2.57684000
H	-0.19550500	-2.65314500	3.21436000
H	-1.89270000	-2.27471400	3.71298500
C	-1.00521200	-0.40604700	2.04405400
H	-1.04189000	0.15886500	1.10742700
H	-1.70947000	0.04219000	2.76399000
H	0.01691000	-0.38556600	2.45073300
I	-2.85588200	-4.90118500	0.03631900

TS-S7

C	4.23269400	6.49666200	-1.08806200
C	3.81743100	5.18488600	-0.84090000
C	2.45816400	4.84245200	-0.88544100
C	1.51782300	5.84310100	-1.18064200
C	1.93191900	7.15416800	-1.42830500
C	3.28996200	7.48566200	-1.38225900
H	5.29647000	6.74595600	-1.05438500
H	4.55848300	4.41248000	-0.61493400
H	0.45910600	5.57491700	-1.22186400
H	1.18993300	7.92184600	-1.66347200
H	3.61280900	8.51117300	-1.57892900
C	2.00251800	3.43669000	-0.57399600
C	1.63007100	3.28172300	0.90414700
H	2.79721300	2.71887000	-0.82351400
H	1.11807400	3.19599900	-1.18010900
C	0.98265500	2.00422900	1.29392700
H	2.49155000	3.47313700	1.56587100
H	0.88921100	4.05478700	1.17674400
C	0.70273600	1.72712100	2.73586000
H	0.62162700	1.31582200	0.53433100
H	1.65610400	1.57385400	3.26519500
H	0.27652100	2.65298600	3.15875800
C	-0.27439400	0.61403900	3.05111400
H	-1.22840600	0.75609500	2.52735900
H	-0.46353100	0.56251800	4.13033300
O	0.29057400	-0.70344800	2.71826300
S	-0.27572100	-1.54773100	1.49184500
O	0.87293300	-2.34217500	1.00101100
O	-0.88185000	-0.61651700	0.52574600
C	-1.43845300	-2.68004700	2.17768200
H	-2.26237700	-2.09348300	2.61007900
H	-1.79820300	-3.29178200	1.33633700
H	-0.89788700	-3.27840900	2.92282100
O	-1.14571100	2.76085800	1.03737800
S	-1.78809100	2.86753500	-0.32018200
O	-1.89858100	1.48901100	-0.96047600
O	-1.15917800	3.82370700	-1.24309800
C	-3.45889100	3.41536200	-0.00995700
H	-3.95475800	2.69556800	0.65519100
H	-3.39031400	4.40519300	0.46049500
H	-3.97300400	3.48303500	-0.97774200
I	3.39006900	0.36628400	0.93813400
Mg	2.52616900	-2.02596600	-0.12081700
Mg	-2.76839100	-0.21697100	-0.47798900
I	-4.14281600	-0.02426300	1.93927300
O	-4.39928400	0.32828300	-1.70180500
C	-5.60794600	-0.43068100	-1.64415200
H	-5.73539400	-0.77492200	-0.61028400
H	-6.46169900	0.20873600	-1.92420600
H	-5.54921500	-1.29860400	-2.32093900
C	-4.14609900	0.86460500	-2.99997200
H	-3.22361500	1.45385700	-2.94158300
H	-4.02453900	0.05328600	-3.73788600
H	-4.98293800	1.51471300	-3.30621300
I	-2.49239000	-2.67845900	-1.71936700
O	1.67357000	-1.59823100	-1.90745700
C	1.48019000	-2.61776900	-2.90306200
H	2.01043700	-3.51712500	-2.56339300
H	0.40562700	-2.82937300	-3.01120100
H	1.90567500	-2.28033900	-3.86169700
C	1.03887900	-0.35723300	-2.24025900

H	1.27285000	0.36073500	-1.44683400
H	1.44010500	0.02075100	-3.19425400
H	-0.04880800	-0.49911200	-2.31730000
I	4.07806600	-4.16783600	-0.20001200

S8

C	6.12560500	5.01591200	-0.79257600
C	5.52191700	3.79577800	-0.47677300
C	4.17442100	3.55873800	-0.78722800
C	3.44116500	4.57235900	-1.42484800
C	4.04382300	5.79248400	-1.74259800
C	5.38679600	6.01958600	-1.42616400
H	7.17779800	5.18232100	-0.54693000
H	6.10650900	3.01192600	0.01430300
H	2.39502200	4.38165800	-1.67818900
H	3.46231800	6.57017200	-2.24503100
H	5.85800700	6.97340400	-1.67669500
C	3.50214500	2.26205900	-0.40312900
C	2.98607400	2.29892100	1.03984000
H	4.20698200	1.42469300	-0.52700100
H	2.64641700	2.08907400	-1.07125500
C	2.06171600	1.16902800	1.44375300
H	3.81789800	2.39983300	1.75741200
H	2.35511600	3.19804800	1.15204300
C	1.51647600	1.29002700	2.86657000
H	1.25261900	1.07510900	0.71531100
H	2.17465500	0.80429300	3.60243700
H	1.51741400	2.36467400	3.11821500
C	0.07950500	0.85480100	3.04630100
H	-0.58015700	1.37151100	2.33701900
H	-0.27215600	1.02638100	4.07127800
O	-0.04386300	-0.60695100	2.85041800
S	-0.91025200	-1.21150300	1.66782700
O	-0.12615600	-2.37305900	1.16367500
O	-1.18215000	-0.15832100	0.68036300
C	-2.35739700	-1.86028600	2.43575300
H	-2.92275300	-1.00852800	2.84297900
H	-2.92297800	-2.36626500	1.63853200
H	-2.01780100	-2.56097900	3.21048300
O	-0.21035800	2.80106400	0.49462600
S	-0.58023600	2.90816100	-0.93703900
O	-1.66856500	1.90126500	-1.32232700
O	0.53979000	2.83098200	-1.89940900
C	-1.38106100	4.49249000	-1.14780600
H	-2.25532600	4.52615800	-0.48379500
H	-0.65700600	5.27197100	-0.87450100
H	-1.68120500	4.59466000	-2.19898300
I	3.14892100	-0.80203600	1.20058700
Mg	1.31466000	-2.60775400	-0.18528300
Mg	-2.88160700	0.66357500	-0.42556000
I	-3.93377200	1.60833500	1.96228500
O	-4.47267800	1.30232300	-1.64650900
C	-5.80732000	0.88794300	-1.36127000
H	-5.90594900	0.82401400	-0.27016300
H	-6.52044300	1.63527900	-1.74760900
H	-6.01369400	-0.09546800	-1.81554500
C	-4.22833100	1.47843000	-3.04068700
H	-3.19740800	1.83540700	-3.14923100
H	-4.35261600	0.52363900	-3.57899100
H	-4.92604700	2.22808300	-3.45061600
I	-3.13106100	-1.95519600	-1.45525400
O	0.84700000	-1.71495700	-1.91119400
C	0.60588300	-2.47102800	-3.10914300
H	0.80625000	-3.52733200	-2.88610400
H	-0.44228600	-2.34452300	-3.41847100
H	1.28747700	-2.12803400	-3.90349400
C	0.63849200	-0.30174800	-2.07898100
H	0.76279500	0.17239000	-1.10327400
H	1.37942700	0.10261700	-2.78377400
H	-0.38103900	-0.11517100	-2.43928600
I	2.65496300	-4.85580400	-0.43397200

S9

I	-0.94341400	3.43509100	1.27646300
Mg	-3.67055400	-1.13510600	0.35583000
O	-4.70190900	-0.01399900	1.68595200
O	-1.89986600	-0.85704100	1.27300000

C	-5.58136300	-0.52549600	2.68942300
C	-4.31301800	1.35123900	1.88396100
S	-0.44596200	-0.61392100	1.07360100
H	-6.51184100	0.06485800	2.70911800
H	-5.09544400	-0.48488100	3.67859400
H	-5.80947100	-1.56805200	2.43083800
H	-3.74778200	1.45490900	2.82390800
H	-5.20757700	1.99397100	1.91203800
H	-3.67616900	1.65042600	1.04325800
C	0.22151600	-0.16912900	2.64582300
O	0.27785600	-1.98773500	0.76442800
O	-0.09116300	0.36356200	0.03966100
H	1.29442700	0.02373600	2.52492800
H	-0.30457500	0.75026500	2.94121100
H	0.02410000	-0.99961000	3.33614600
C	0.70464900	-2.36297400	-0.61142100
Mg	-0.23067700	2.12931200	-1.11076400
C	2.05918600	-3.04638600	-0.53110400
H	-0.07153600	-3.04444500	-0.97760000
H	0.71446000	-1.45970400	-1.23424800
O	-0.45949900	3.93804600	-2.16039900
O	-1.76359200	1.35014000	-2.15465400
I	2.14347800	1.40108400	-2.32642600
C	3.25407400	-2.09454600	-0.44075700
H	2.14911600	-3.65525200	-1.44410000
H	2.05476100	-3.75091000	0.31589300
C	0.55754100	4.93177900	-2.03532100
C	-1.75895400	4.49193800	-2.35222700
S	-2.54985600	0.06785300	-2.14484100
C	3.18810400	-1.13737700	0.73124000
H	3.37938500	-1.53818200	-1.37879300
H	0.38927600	5.54450600	-1.13398000
H	0.56328200	5.57475500	-2.93095200
H	1.52043100	4.41024600	-1.96092400
H	-1.76606700	5.11858400	-3.25990300
H	-2.05713100	5.09640100	-1.47933400
H	-2.45855700	3.65743800	-2.47834400
O	-3.68757800	0.17003200	-1.13501100
C	4.33717300	-0.14418200	0.93438100
H	2.29532000	-0.52486700	0.54112600
H	3.00906200	-1.69784600	1.66385800
C	3.94434800	0.83104000	2.02018600
H	5.25860700	-0.68027100	1.20805900
H	4.52604300	0.38815500	-0.01030000
C	3.24899600	2.00997000	1.70631700
C	4.14903500	0.51473400	3.37289200
C	2.76826100	2.84798900	2.71606100
H	3.07119900	2.26111200	0.65711700
C	3.66986100	1.35101600	4.38560400
H	4.68897400	-0.40046600	3.63341700
C	2.97351000	2.51926100	4.05942200
H	2.21560100	3.75127600	2.45007900
H	3.84177400	1.08977500	5.43325000
H	2.59329100	3.17262800	4.84870300
I	5.09313300	-3.33185100	-0.37103800
O	-1.75995500	-1.15446100	-1.90012100
C	-3.31518100	-0.02565700	-3.74282700
H	-2.51173800	-0.07830800	-4.48995400
H	-3.93182000	-0.93409900	-3.76283000
H	-3.92770600	0.87378000	-3.88647000
I	-4.58988700	-3.61339900	0.22444400

S10

I	3.03604200	0.30704000	1.80801300
Mg	3.98926200	-0.29787600	-0.72239000
O	5.50299900	1.00506500	-0.98682100
O	2.62286300	0.68784200	-1.86139400
C	6.84803100	0.63443700	-1.31015100
C	5.31902500	2.40648300	-0.75120500
S	1.13847500	0.63806100	-1.89496000
H	7.52007100	0.88872000	-0.47453000
H	7.17021400	1.16048400	-2.22317000
H	6.86134100	-0.44950900	-1.48523600
H	5.62376300	2.97791600	-1.64246600
H	5.91229600	2.72401300	0.12133100
H	4.25447000	2.58766000	-0.55576300

C	0.56766100	1.67600000	-3.19708400
O	0.78131800	-0.83417900	-2.32045800
O	0.44950000	0.97095000	-0.63044600
H	-0.52578200	1.55098600	-3.26577500
H	0.84801000	2.70232300	-2.91577800
H	1.06855300	1.35087600	-4.11901200
C	-0.44537400	-1.54287200	-1.86010800
Mg	0.91136300	2.12884800	1.11594200
C	-0.02867500	-2.71193800	-0.98835400
H	-1.09027300	-0.81635500	-1.35285200
H	-0.92718600	-1.87134000	-2.78797600
O	1.11888400	3.08221500	2.97275000
O	-1.00361100	1.93483200	1.48833600
I	1.74468400	4.28115800	-0.38734400
C	0.29160200	-2.31932300	0.45345600
H	0.83295800	-3.19800200	-1.46855600
H	-0.86135900	-3.43184200	-0.99726400
C	2.36494800	3.67898100	3.33737100
C	0.03631700	3.54365300	3.78653000
S	-2.34439400	2.02143100	0.79368500
C	-0.94583500	-1.98285700	1.26707400
H	1.02879900	-1.50809800	0.49375800
H	2.60240400	3.44009700	4.38717800
H	2.31692700	4.77162100	3.20144600
H	3.14597900	3.26663100	2.68818700
H	-0.03820400	4.64259700	3.72715500
H	0.20261400	3.24270700	4.83399100
H	-0.88474400	3.09317300	3.40619000
C	-2.17318500	3.29288500	-0.44429600
O	-3.42519800	2.40776800	1.70455000
O	-2.58346200	0.72093300	0.05743000
C	-0.72282400	-1.50843600	2.70498600
H	-1.47391700	-1.17816400	0.73287400
H	-1.62440600	-2.84835400	1.25044800
H	-1.38994300	3.00268600	-1.15218300
H	-3.13575300	3.39650800	-0.96020400
H	-1.90130100	4.21936100	0.07807400
C	-2.01807000	-1.14419600	3.39671400
H	-0.20859600	-2.30077700	3.27475800
H	-0.04934800	-0.63749900	2.70917200
C	-2.21288800	0.13351600	3.93783500
C	-3.06570400	-2.07548700	3.50298100
C	-3.41476300	0.47875600	4.56195100
H	-1.41591000	0.87268900	3.85120800
C	-4.26821800	-1.73604900	4.12595100
H	-2.94096700	-3.08015400	3.09164900
C	-4.44926400	-0.45414600	4.65713300
H	-3.54649100	1.48730800	4.96141800
H	-5.06907500	-2.47680900	4.19579800
H	-5.39177800	-0.18614100	5.14141100
I	1.42711800	-3.97372500	1.39162400
I	-4.09849700	-2.77852600	-1.09990700
Mg	-3.96721400	-0.09858600	-1.09966100
O	-5.56235600	0.57399300	-0.02882700
C	-6.14252600	1.86681200	-0.21281000
H	-7.22557900	1.82198500	-0.01247000
H	-5.66559400	2.59515300	0.46173200
H	-5.97941400	2.15782300	-1.25979600
C	-5.69739000	0.06148600	1.30854900
H	-5.26310700	-0.94614800	1.31721000
H	-5.15749600	0.70898700	2.01331800
H	-6.76530600	-0.00076100	1.57212700
I	-3.50131700	1.20038000	-3.44271500
I	4.50465300	-2.72037300	-1.61993200

TS-S11

I	3.08771800	0.24692400	1.69333400
Mg	4.19305900	-0.00655500	-0.84829400
O	5.63780400	1.40962200	-0.81253000
O	2.86383400	1.03482100	-1.89838600
C	7.01845300	1.09684500	-0.60464300
C	5.29786900	2.77516600	-0.54092800
S	1.37501600	0.88501400	-2.06556400
H	7.29254800	1.26121200	0.45077400
H	7.64523400	1.72753100	-1.25535000
H	7.16265300	0.04042500	-0.86827900

H	5.90533500	3.44102700	-1.17447200
H	5.46980600	3.00783200	0.52251300
H	4.23638900	2.91520400	-0.77699600
C	0.89355800	2.11596100	-3.24501000
O	1.03753800	-0.46130100	-2.63165500
O	0.62951200	1.14302000	-0.79263800
H	-0.18703400	2.01014700	-3.41011600
H	1.14388900	3.10058800	-2.82551800
H	1.45034700	1.91725500	-4.17049100
C	-0.65460300	-1.52639000	-2.13566700
Mg	0.92945700	2.09896900	1.01423900
C	0.02947500	-2.60838900	-1.37589500
H	-1.08114500	-0.65061100	-1.64532100
H	-0.80598000	-1.63766500	-3.20567100
O	1.06620500	2.66182700	3.03068600
O	-1.02366800	1.87045400	1.29922200
I	1.67359600	4.49857700	-0.10433200
C	0.32121000	-2.25743700	0.08044300
H	0.96229100	-2.83110500	-1.91865000
H	-0.57406600	-3.52668300	-1.44784600
C	2.21165600	3.34566800	3.53820100
C	0.28636400	2.05059100	4.05881100
S	-2.31911900	1.94496700	0.52861600
C	-0.90761800	-2.13786300	0.96969000
H	0.92985400	-1.34651400	0.13276400
H	2.85571800	2.65323600	4.10484800
H	1.88926200	4.17365700	4.19099200
H	2.76600000	3.75468000	2.68497500
H	0.02476300	2.79984700	4.82393700
H	0.84701700	1.22512700	4.52996900
H	-0.63090300	1.66918800	3.60127500
C	-2.18125500	3.30494700	-0.60831300
O	-3.48187000	2.14661500	1.39990200
O	-2.45637100	0.70062900	-0.34571800
C	-0.62580000	-1.54943200	2.35734600
H	-1.62376600	-1.47724100	0.45539700
H	-1.39676000	-3.12117900	1.05714100
H	-1.31994700	3.12840500	-1.26302100
H	-3.12083900	3.33631500	-1.17836000
H	-2.03519400	4.22021300	-0.02040200
C	-1.81538300	-1.60695200	3.28561900
H	0.21381100	-2.09577700	2.81478700
H	-0.29834600	-0.50670300	2.23729900
C	-2.63225400	-0.48735500	3.50003600
C	-2.13087000	-2.80043300	3.95670900
C	-3.71332200	-0.54690000	4.38500100
H	-2.43022200	0.44158800	2.96833100
C	-3.21843100	-2.86813900	4.83042600
H	-1.50459200	-3.68343300	3.79974700
C	-4.01049300	-1.73653100	5.05533600
H	-4.32241500	0.34553600	4.54869700
H	-3.44198700	-3.80451600	5.34819700
H	-4.85314500	-1.78372000	5.74965900
I	1.70386000	-3.78982100	0.86399500
I	-3.42765500	-2.40625500	-2.18484200
Mg	-4.18337900	-0.14663500	-0.80956200
O	-5.02455000	-0.81257400	0.90929200
C	-6.00945300	-0.00239900	1.56525900
H	-7.02019800	-0.31136800	1.25249600
H	-5.90114800	-0.10739200	2.65421900
H	-5.83060100	1.04045000	1.27946800
C	-5.01000100	-2.17361200	1.35489900
H	-4.19233000	-2.68705600	0.83668700
H	-4.84131500	-2.20626200	2.44043100
H	-5.96531600	-2.66394300	1.10395700
I	5.03423000	-2.33528500	-1.78281600
I	-5.74087700	1.62989200	-2.06916500
**S12**			
Mg	2.24616400	-0.02317700	-0.23978800
O	1.56217900	-1.76308100	-1.05762500
C	0.22686300	-1.89058900	-1.56494100
H	0.22574600	-2.54688200	-2.45001100
H	-0.43344800	-2.30837600	-0.79102200
H	-0.11539400	-0.89160000	-1.85776600
C	2.13170100	-3.01104600	-0.64393300

H	2.14460300	-3.71253700	-1.49371700
H	3.16278300	-2.81155900	-0.32171900
H	1.54706600	-3.43059200	0.18911200
Mg	-2.32135000	0.42979400	0.15247600
O	-3.44921500	1.93502400	0.91598600
C	-4.84494200	1.81272900	1.20352000
H	-5.36755700	2.73955000	0.91631200
H	-5.23302200	0.97075800	0.61533100
H	-4.99825900	1.61722600	2.27805000
C	-2.80410500	3.01286000	1.60502000
H	-2.80537700	2.82513500	2.69144800
H	-1.77223500	3.08298300	1.23703800
H	-3.33248500	3.95634200	1.39177200
O	1.25381700	-0.00602800	1.45816200
S	0.04661400	-0.68589700	2.06781600
O	-1.19702300	0.11785600	1.72794800
O	-0.06472100	-2.10967900	1.73845500
C	0.24524400	-0.48042100	3.82078900
H	1.16013300	-1.01262700	4.11337400
H	-0.63470200	-0.91986500	4.30893900
H	0.32867600	0.59227900	4.03734200
S	0.08531700	2.16632000	-1.14827700
O	-1.17594900	1.31797400	-1.17620800
O	1.28488700	1.26753900	-1.37172200
C	-0.00506100	3.16175300	-2.61666800
H	-0.88579800	3.81090800	-2.52435500
H	0.91590200	3.75747600	-2.66856500
H	-0.09430400	2.49528400	-3.48393800
O	0.18952900	3.02614800	0.03454600
I	-3.80057700	-1.53446000	-0.85722500
I	4.89591200	-0.02587600	-0.06412500
**TS-S13**			
C	-4.24181800	6.29808100	-0.34072300
C	-3.67438700	5.03281700	-0.51297900
C	-2.28853200	4.84461500	-0.39834600
C	-1.48311700	5.95580200	-0.10511700
C	-2.04701500	7.22249600	0.06776900
C	-3.42914300	7.39749800	-0.04878200
H	-5.32282100	6.42715500	-0.43914000
H	-4.31365700	4.17618200	-0.74562600
H	-0.40086200	5.82420500	-0.01756500
H	-1.40356600	8.07758300	0.29042700
H	-3.87093500	8.38835900	0.08309800
C	-1.68291800	3.46893400	-0.52914900
C	-1.63174400	2.74088300	0.81769700
H	-2.26508500	2.86042800	-1.23811200
H	-0.65903600	3.53951600	-0.92711300
C	-1.01383300	1.38773700	0.77589400
H	-2.64189300	2.64972000	1.25455900
H	-1.07313000	3.33831400	1.55976800
C	-0.87011700	0.58624600	2.03098300
H	-0.60473600	1.00102900	-0.15898800
H	-1.86393100	0.47880900	2.49642000
H	-0.27960400	1.18703200	2.74285900
C	-0.21704500	-0.75767200	1.78854200
H	-0.81656900	-1.40685300	1.14335300
H	0.78909800	-0.65747500	1.37544600
I	1.85477600	2.37306000	0.65251500
I	0.07864300	-1.87586600	3.63686900
Mg	-2.54751500	-2.35325100	-1.69549800
I	-3.32244300	-0.01995000	-0.31493800
O	-1.64036400	-3.40423200	-0.13282900
C	-0.48576900	-4.21015500	-0.37800600
H	-0.77618500	-5.26669400	-0.50774700
H	-0.00782000	-3.83882300	-1.29210100
H	0.21693100	-4.12303000	0.46631300
C	-2.35763700	-3.77962100	1.04323200
H	-1.67190600	-3.82179700	1.90364600
H	-3.12198200	-3.01563900	1.23285000
H	-2.83622500	-4.76425400	0.90747400
Mg	2.85592900	0.11052300	-0.62286500
C	-2.06541000	-2.91749000	-3.67244500
H	-1.62271000	-2.08685200	-4.25142300
H	-1.33031900	-3.74423000	-3.72451500
H	-2.95443400	-3.25595700	-4.24053700

I	0.73987500	-0.88527200	-2.02788900
I	5.40162500	0.05693200	-1.45470700
O	3.00633500	-1.15970300	1.01628900
C	3.09874400	-2.56661200	0.77549600
H	2.49994700	-2.78955000	-0.11714700
H	4.14709000	-2.85654000	0.59708700
H	2.69844900	-3.11728800	1.64175100
C	3.73240400	-0.73925900	2.17785400
H	3.55967500	0.33710800	2.30256800
H	3.35467000	-1.27642500	3.06189700
H	4.80966700	-0.93066300	2.04613400
O	-4.33259300	-3.45424100	-1.31858400
C	-4.33197400	-4.78396100	-1.83196700
H	-3.33155900	-4.99423100	-2.23089400
H	-4.57418400	-5.50434700	-1.03187200
H	-5.06803500	-4.88416900	-2.64735100
C	-5.57979200	-3.07572100	-0.74575900
H	-5.48720000	-2.04218600	-0.39002300
H	-6.38289700	-3.13345000	-1.50012600
H	-5.82886400	-3.73650000	0.10270400

TS-S14

C	-8.37697900	-2.28410400	-0.30791400
C	-7.25601900	-1.48015800	-0.08377100
C	-6.04757000	-2.03620100	0.36262100
C	-5.99002400	-3.42221300	0.57777100
C	-7.10780800	-4.22986600	0.35456900
C	-8.30609800	-3.66295500	-0.09008500
H	-9.31091700	-1.83117700	-0.65081100
H	-7.31897400	-0.40116500	-0.25275500
H	-5.05620200	-3.87047200	0.92920100
H	-7.04441600	-5.30650900	0.53267000
H	-9.18235600	-4.29298700	-0.26209900
C	-4.82078300	-1.17856300	0.55690700
C	-3.95176600	-1.11487800	-0.70564600
H	-5.11741400	-0.15494200	0.83920300
H	-4.21831000	-1.57470600	1.38883300
C	-2.71054500	-0.24546100	-0.57844500
H	-4.55782200	-0.69289400	-1.52884900
H	-3.67002900	-2.13267100	-1.02302700
C	-2.00112000	-0.03962200	-1.91232700
H	-2.96669500	0.72275600	-0.12976600
H	-2.73112600	0.35892100	-2.63799200
H	-1.69406100	-1.01655200	-2.32145500
C	-0.81095900	0.86524400	-1.90033500
H	-0.47115100	1.33943800	-2.81623900
H	-0.26584500	1.06942800	-0.98466900
I	-1.28361000	-1.10482100	0.88135800
I	1.37964200	-0.80593700	-2.41558100
Mg	-1.21105200	3.13422800	1.24865800
I	-2.07351400	3.32620300	-1.42242600
O	0.67804100	2.39004700	0.91421200
C	1.30898700	1.53817600	1.88085200
H	2.04804000	2.11004100	2.46536500
H	0.52609800	1.14006700	2.53613500
H	1.81799500	0.70572400	1.37847600
C	1.59238000	2.96899000	-0.02340200
H	2.11636400	2.17911500	-0.58194300
H	1.01216400	3.58779400	-0.71987000
H	2.34017900	3.58561200	0.50140700
Mg	3.09403300	-1.35952700	-0.21579200
C	-2.31792400	2.39144200	2.87601500
H	-3.10826800	1.68033200	2.57198300
H	-1.70591500	1.85691700	3.62697600
H	-2.83335600	3.20787800	3.41955300
I	4.89344600	0.64934200	0.00047100
I	2.13235600	-2.49537600	2.04730200
O	4.00347400	-2.97603600	-1.10309400
C	5.39928400	-3.23263100	-0.92475200
H	5.89601800	-2.26472700	-0.77843500
H	5.56046300	-3.87246500	-0.04104500
H	5.80442900	-3.72536500	-1.82342700
C	3.23381800	-4.15232800	-1.36601600
H	2.18746900	-3.84743700	-1.49648800
H	3.59268100	-4.62988700	-2.29225800
H	3.30803500	-4.85772300	-0.52263100

O	-0.46070100	5.03518600	1.61795300
C	0.21384700	5.17456500	2.86918900
H	-0.03220200	4.29723100	3.48358000
H	1.30491800	5.22950000	2.71675800
H	-0.13154400	6.08337400	3.38877900
C	-0.27251000	6.13903900	0.73467600
H	-0.83390500	5.93049700	-0.18480500
H	-0.65401700	7.06369000	1.19863900
H	0.79712300	6.26369400	0.49505000

TS-S15

C	-2.64415300	5.45023700	-1.56285800
C	-1.92118600	4.36356900	-1.06603400
C	-2.01178400	3.10250700	-1.67637500
C	-2.84577400	2.95301900	-2.79325200
C	-3.57008000	4.03902600	-3.29301300
C	-3.47018300	5.29106200	-2.68012000
H	-2.56762900	6.42404800	-1.07230000
H	-1.28268800	4.48871000	-0.18666000
H	-2.93435900	1.97165100	-3.26688200
H	-4.22005800	3.90459600	-4.16143000
H	-4.03945900	6.13972600	-3.06761700
C	-1.20001100	1.94416300	-1.15543700
C	0.24855300	1.97234700	-1.64733700
H	-1.20385600	1.96224200	-0.05457100
H	-1.66818200	0.99735700	-1.45930500
C	1.13647700	0.95395100	-1.03120400
H	0.70569300	2.96089800	-1.46307400
H	0.29641100	1.85609700	-2.74461300
C	2.56960400	0.83978200	-1.42495200
H	0.74745600	0.28339100	-0.26605400
H	3.03947600	1.82825400	-1.28526900
H	2.61330700	0.65805700	-2.51232700
C	3.31220100	-0.23611100	-0.65668000
H	3.31724000	-0.03887900	0.42153500
H	2.90450600	-1.23455200	-0.85171900
I	5.40744700	-0.33455500	-1.24197000
I	0.11302100	-1.44756100	-2.51991900
Mg	-0.69386200	1.08277100	2.66950900
I	1.56220100	2.36948300	1.59659700
Mg	-1.60072100	-2.03396500	-0.42081300
C	-2.69708100	1.72936300	2.63380500
H	-2.98784100	2.22786300	1.69195500
H	-3.40411000	0.88902200	2.76366400
H	-2.90437100	2.45363300	3.44624500
I	0.01000400	-1.64450600	1.83839100
I	-4.02639000	-0.96218600	-0.39256100
O	-1.67292000	-4.07057700	-0.44164100
C	-0.46817100	-4.83971400	-0.38124800
H	0.36686700	-4.18066100	-0.65137300
H	-0.31698200	-5.23143900	0.63781800
H	-0.52120900	-5.67200400	-1.10133400
C	-2.85665900	-4.82643600	-0.16255800
H	-3.71467800	-4.14900700	-0.26169300
H	-2.94868500	-5.65276900	-0.88564400
H	-2.81637900	-5.22826200	0.86335400
O	-0.10414300	0.61077500	4.60109600
C	-1.11018100	0.02992200	5.43376900
H	-1.07953000	-1.07025600	5.36109500
H	-0.95714000	0.33971600	6.48044600
H	-2.08369100	0.39535300	5.08210600
C	1.22661200	0.27022300	4.99044700
H	1.37684000	-0.82139700	4.94251200
H	1.91435600	0.76800200	4.29483700
H	1.41812600	0.62606900	6.01610800

TS-S16

C	-8.59241000	2.11907600	-0.71279200
C	-7.30913500	1.95742700	-0.18315400
C	-6.78367300	0.67881100	0.05605200
C	-7.57727800	-0.43708900	-0.25333100
C	-8.85977600	-0.28055500	-0.78426400
C	-9.37227800	0.99959900	-1.01603100
H	-8.98580700	3.12405000	-0.88643600
H	-6.70509200	2.83787100	0.05465800
H	-7.18336100	-1.44100900	-0.07024700
H	-9.46388700	-1.16201300	-1.01456600

H	-10.37691400	1.12384000	-1.42787400
C	-5.38087100	0.49884500	0.58435100
C	-4.35920400	0.30723300	-0.54330200
H	-5.09097200	1.37516300	1.18708700
H	-5.34592900	-0.37414500	1.25430000
C	-2.91242700	0.17123500	-0.09475600
H	-4.40081100	1.19046800	-1.20736600
H	-4.64136700	-0.55750400	-1.16659200
C	-1.94306700	0.18770400	-1.27010500
H	-2.65009900	0.95402600	0.62809900
H	-2.13035800	1.11082000	-1.84633300
H	-2.18331000	-0.63460800	-1.96252300
C	-0.48369300	0.14636000	-0.94061300
H	0.24232900	0.52444300	-1.65370100
H	-0.13763800	-0.15836100	0.04461800
I	-2.63153900	-1.66900100	1.11447600
I	0.39749100	-2.32055100	-1.78209300
Mg	2.48377100	3.26047800	-0.50732000
I	-0.17706500	2.72082900	0.21974700
Mg	2.47602000	-1.50799400	-0.11036100
I	3.65616300	0.68650800	-1.32298100
I	1.77075200	-1.28829500	2.44851900
O	3.76430100	-3.04506600	-0.46500900
C	4.32024100	-3.23884300	-1.77029900
H	4.37488500	-2.25769900	-2.25960900
H	5.33377600	-3.66150900	-1.68233300
H	3.68092500	-3.91353700	-2.36237600
C	3.66013000	-4.25190100	0.30112100
H	3.25246000	-3.98561700	1.28540200
H	2.98560600	-4.96462200	-0.20139000
H	4.65864500	-4.70098000	0.42287900
C	3.14511500	4.81167500	-1.77289000
H	3.17427600	5.79518700	-1.26501700
H	4.16257500	4.62806300	-2.16817100
H	2.49000000	4.94097600	-2.65514000
O	3.46021200	3.25827400	1.30440200
C	3.15743600	2.37368300	2.38820300
H	2.11041200	2.06544900	2.28712700
H	3.80598000	1.48427600	2.35237600
H	3.29702700	2.89929600	3.34714800
C	4.81052100	3.72469000	1.31421800
H	4.92783800	4.42814200	0.47973600
H	5.02125000	4.24343500	2.26375300
H	5.50864400	2.87997700	1.18863200
**38 (triplet)**			
C	-2.75601300	1.32320200	-0.76519600
C	-1.75140300	2.40647300	-1.09735700
H	-2.58089500	0.88945600	0.22207400
C	-0.35615600	1.83068000	-1.33674200
H	-2.09410900	2.98091000	-1.97973100
H	-1.71328900	3.11870100	-0.25699600
C	0.70455100	2.92895700	-1.39967600
H	-0.35780600	1.26642800	-2.29450300
C	2.12631200	2.39254200	-1.64003500
H	0.69844500	3.49435800	-0.45155100
H	0.47076200	3.67147600	-2.19048900
H	2.34593000	1.61855300	-0.87988600
H	2.16607100	1.88662500	-2.61960800
H	-2.80838300	0.53100200	-1.52235600
C	3.19230200	3.45547400	-1.56113900
C	3.72373400	4.04714300	-2.71606600
C	3.64213400	3.90844500	-0.30987200
C	4.67999800	5.06333900	-2.62701300
H	3.38245000	3.70642700	-3.69793900
C	4.59713700	4.92257900	-0.21655200
H	3.23346100	3.45810100	0.59957000
C	5.12008800	5.50435700	-1.37633000
H	5.08317200	5.51187000	-3.53884500
H	4.93616700	5.26026300	0.76626700
H	5.86817300	6.29792100	-1.30458700
Ni	0.32803800	0.49105000	-0.02776900
P	-0.98983800	-1.51645600	0.13470800
P	1.81161100	-1.39978700	0.64146300
C	0.28673400	-2.28111000	1.24122900
C	-1.01110500	-2.61060100	-1.32708400

C	-2.61840100	-1.70893500	0.92719900
C	2.35398300	-2.32785200	-0.83845900
C	3.08852800	-1.70434300	1.91365500
H	0.11050900	-1.90724100	2.26086800
H	0.31580600	-3.38024500	1.26382100
C	-1.38453500	-3.96139700	-1.23114300
C	-0.57233400	-2.10061800	-2.55661700
C	-2.77188000	-1.42208800	2.29401700
C	-3.75682400	-1.97789300	0.14828100
C	2.18833900	-3.71281500	-0.99229100
C	2.92404400	-1.58454700	-1.88467600
C	4.25035300	-2.44611600	1.65085500
C	2.91323300	-1.11545900	3.17913100
C	-1.31337100	-4.78854600	-2.35187000
H	-1.73129000	-4.36525100	-0.27679600
C	-0.49594800	-2.93268400	-3.67642700
H	-0.27592400	-1.05273900	-2.63291800
C	-4.04212000	-1.42718900	2.87251200
H	-1.90511000	-1.16660600	2.90558800
C	-5.02474500	-1.97437100	0.73191500
H	-3.65573300	-2.18225100	-0.91913500
C	2.57563800	-4.33887500	-2.17756300
H	1.74640900	-4.30858700	-0.19204900
C	3.32496300	-2.21492900	-3.06475400
H	3.04657000	-0.50430800	-1.77639600
C	5.21559400	-2.61192600	2.64779200
H	4.40323200	-2.89554500	0.66809400
C	3.87823900	-1.29132600	4.17153000
H	2.02773400	-0.50708600	3.38184100
C	-0.86562200	-4.27532200	-3.57480300
H	-1.60483200	-5.83866500	-2.27226400
H	-0.14162300	-2.53012800	-4.62781400
C	-5.17038500	-1.70226200	2.09470000
H	-4.14970100	-1.20056900	3.93576700
H	-5.90289100	-2.17716200	0.11478100
C	3.14458200	-3.59210900	-3.21451400
H	2.42730300	-5.41470400	-2.29516600
H	3.76877600	-1.62671300	-3.87137300
C	5.03073400	-2.03950400	3.90893400
H	6.11673900	-3.19253400	2.43537500
H	3.73281000	-0.83207900	5.15228200
H	-0.80456100	-4.92672000	-4.44998000
H	-6.16409800	-1.69373600	2.54849200
H	3.44333200	-4.08588900	-4.14232100
H	5.78736000	-2.17083400	4.68634200
I	-0.10679500	1.53036600	2.35655800
I	-4.82065600	2.06245500	-0.63367000
**41 (triplet)**			
C	3.08153200	-1.33638400	-0.60694300
C	2.16649800	-2.48095600	-0.99218900
H	2.77691400	-0.85729800	0.32546500
C	0.78054800	-1.94621900	-1.34907200
H	2.61571000	-3.05689600	-1.82608800
H	2.08972600	-3.17995000	-0.14167300
C	-0.25556800	-3.05850900	-1.53149300
H	0.85837100	-1.37080500	-2.29470400
C	-1.59369700	-2.52243600	-2.10039700
H	-0.44355600	-3.54679000	-0.55752500
H	0.11227100	-3.87360200	-2.19031300
H	-1.64784900	-1.44060600	-1.88176700
H	-1.59804100	-2.60967600	-3.19843700
H	3.18016700	-0.58007600	-1.39571400
C	-2.82342500	-3.17595700	-1.52160600
C	-3.53664600	-4.16598000	-2.21187200
C	-3.26521600	-2.81324200	-0.23628600
C	-4.65754400	-4.77583200	-1.63864400
H	-3.20927100	-4.46172500	-3.21269800
C	-4.38581400	-3.41467900	0.33815400
H	-2.71768500	-2.04703400	0.31855200
C	-5.08727300	-4.40172700	-0.36277800
H	-5.19916500	-5.54634600	-2.19385200
H	-4.71297000	-3.10899400	1.33539000
H	-5.96529200	-4.87552800	0.08341400
Ni	-0.06846600	-0.78781500	0.06635300
P	0.89175300	1.35431400	0.32788400

P	-1.85234100	0.76432700	0.78536100
C	-0.49177400	1.83342000	1.47614800
C	0.76546000	2.58200600	-1.02156200
C	2.46894700	1.68993600	1.17966100
C	-2.65444200	1.77681000	-0.51179400
C	-3.11206800	0.62623300	2.10874900
H	-0.26444600	1.42752500	2.47235600
H	-0.69629100	2.90953700	1.57851000
C	0.94858000	3.95890600	-0.81091200
C	0.37738400	2.12385600	-2.28871000
C	2.65843800	1.20731700	2.48665300
C	3.55906600	2.24942800	0.49229800
C	-2.50932100	3.16618800	-0.63601100
C	-3.41778600	1.08202000	-1.46703200
C	-4.16222700	1.54443000	2.26206400
C	-3.01881300	-0.47069100	2.98224700
C	0.73677100	4.86174800	-1.85312500
H	1.25448800	4.32467300	0.17246500
C	0.16138900	3.03008300	-3.33024500
H	0.22749700	1.05428700	-2.45139200
C	3.90765600	1.30834400	3.10012500
H	1.83553300	0.73007100	3.02202100
C	4.80829800	2.34257000	1.10959600
H	3.43594000	2.60668900	-0.53167400
C	-3.10590500	3.84606300	-1.70046000
H	-1.91652000	3.72750200	0.08600800
C	-4.02292100	1.76442100	-2.52319600
H	-3.53567700	-0.00144900	-1.38273700
C	-5.09909900	1.37288200	3.28368900
H	-4.25214700	2.39017900	1.57711300
C	-3.95536900	-0.63542700	4.00554300
H	-2.21308200	-1.19630900	2.85412100
C	0.33843500	4.39798900	-3.11275400
H	0.87847300	5.93203000	-1.68343200
H	-0.15251700	2.66519500	-4.31077100
C	4.98571000	1.87593000	2.41418100
H	4.04206800	0.92965800	4.11611500
H	5.64911700	2.77604000	0.56291700
C	-3.86258900	3.14833100	-2.64561300
H	-2.97200100	4.92625900	-1.79328200
H	-4.61317800	1.21144900	-3.25782600
C	-4.99656800	0.28544100	4.15710000
H	-5.91559400	2.09054500	3.39639300
H	-3.87534400	-1.49067300	4.68092700
H	0.16604900	5.10773000	-3.92559500
H	5.96537600	1.94396000	2.89272800
H	-4.32451600	3.68198300	-3.47978200
H	-5.73311800	0.15247400	4.95335100
I	5.15627500	-1.95314200	-0.20348800
C	0.36363200	-1.57607500	1.86708900
H	0.08632100	-0.99865500	2.76830700
H	-0.17796200	-2.54081800	1.92846700
H	1.43984700	-1.81023600	1.94932000
**TS-S17**			
C	-2.51380400	1.47626700	1.06420700
C	-1.83609200	2.70361700	0.57291600
H	-3.58064900	1.34918800	0.88530400
C	-0.91249400	1.97007000	-0.33657900
H	-2.51394500	3.38442500	0.03934700
H	-1.30296700	3.24282200	1.37340900
C	0.42842900	1.49994500	0.10098100
H	-1.28040300	1.72345400	-1.33467400
C	1.19502500	0.71332700	-0.97297600
H	0.33162900	0.87236500	1.00745600
H	1.02615300	2.37446500	0.43181600
H	0.55483700	-0.11370300	-1.31754500
H	1.37446100	1.36820400	-1.84101000
H	-2.11029300	0.99513500	1.95482200
C	2.50385700	0.16601300	-0.45863400
C	3.70850500	0.85829300	-0.65021000
C	2.53063100	-1.03414500	0.27041600
C	4.90967200	0.36648400	-0.13086800
H	3.70510500	1.79271900	-1.21906100
C	3.72847100	-1.52849100	0.79141400
H	1.59882700	-1.58602800	0.42494900

C	4.92318600	-0.82900600	0.59254900
H	5.83915700	0.91790800	-0.29513600
H	3.73051900	-2.46665200	1.35243900
H	5.86151100	-1.21649200	0.99726800
I	-2.36276000	-0.95871800	-0.14186200
**35***			
Ni	-0.17945400	0.46764400	0.06818600
P	-0.85083300	-1.62628000	0.31211000
P	1.67562800	-0.69138300	0.13336900
C	0.86103700	-2.32020300	0.48838500
C	-1.66493300	-2.39015800	-1.11302700
C	-1.74101700	-2.03249000	1.83721100
C	2.42083700	-0.88240500	-1.50914800
C	2.97421800	-0.40798400	1.36117600
H	1.02221000	-2.65275300	1.52360600
H	1.12695600	-3.13988700	-0.19362500
C	-0.90114700	-2.69352400	-2.25391800
C	-3.06231600	-2.53491700	-1.14793800
C	-1.78102600	-1.06388600	2.85402400
C	-2.29769700	-3.30399700	2.05389600
C	3.36356100	-1.90279500	-1.72497800
C	2.00430500	-0.07122200	-2.57317500
C	2.70605800	-0.65910900	2.71703600
C	4.20677100	0.14181800	0.97777300
C	-1.53041200	-3.16350200	-3.40697200
H	0.18181600	-2.55304200	-2.25317100
C	-3.68248500	-3.00694600	-2.30668900
H	-3.66705300	-2.28640700	-0.27371100
C	-2.38031000	-1.36352200	4.07917700
H	-1.34254700	-0.07767400	2.68327500
C	-2.90140500	-3.59244000	3.27865400
H	-2.26425200	-4.06488400	1.27118000
C	3.88635000	-2.10089400	-3.00249800
H	3.69392800	-2.53541900	-0.89695700
C	2.52996600	-0.27840300	-3.85084200
H	1.28244800	0.72611300	-2.39159300
C	3.67874300	-0.38589100	3.67856000
H	1.73873900	-1.05885700	3.03130200
C	5.17500000	0.40975300	1.94695600
H	4.40953900	0.36194000	-0.07143400
C	-2.91957400	-3.32300700	-3.43440600
H	-0.93224400	-3.40341400	-4.28873600
H	-4.76768400	-3.12842400	-2.32691400
C	-2.94388900	-2.62506700	4.28895100
H	-2.41079500	-0.60876200	4.86805400
H	-3.34000100	-4.57865400	3.44640500
C	3.46810700	-1.29081300	-4.06538700
H	4.62415700	-2.88861700	-3.17045900
H	2.20680500	0.35632600	-4.67875300
C	4.91405800	0.14560500	3.29420900
H	3.47047300	-0.58466100	4.73215600
H	6.13624000	0.83235200	1.64658600
H	-3.40929100	-3.69159900	-4.33846200
H	-3.41953400	-2.85739400	5.24450200
H	3.88016300	-1.44975400	-5.06456200
H	5.67384900	0.35955600	4.04920100
I	1.17857100	2.60375900	0.06853300
H	-3.81332300	4.05411900	1.82983400
C	-3.80989400	3.21823400	1.10542200
H	-5.39585200	3.93226700	-0.21690900
H	-1.55575500	4.10728900	-0.88847500
C	-4.31952700	3.72056700	-0.21939600
H	-4.54988200	2.49955600	1.50180800
C	-3.67247600	3.95398900	-1.37139900
H	-1.96279700	4.41044000	-2.56234700
C	-2.20925400	3.76982900	-1.70172500
H	-4.28952700	4.30917600	-2.20538800
H	-1.64411200	3.29157100	0.85211700
C	-2.41553700	2.56749500	1.12856000
H	-2.19364900	2.27057100	2.16590200
C	-1.84980100	2.32852800	-2.09672900
H	-0.79693200	2.28853800	-2.42391300
C	-2.35073400	1.33944200	0.26069900
C	-2.09825700	1.24730300	-1.07984500
H	-2.43884400	2.04227100	-2.98598000

H	-2.75448200	0.43542300	0.73039600
H	-2.25417800	0.25799200	-1.52424600
**34***			
C	4.60052400	-0.42059900	0.05289700
C	3.69250900	0.56923300	-0.33725400
C	2.31487800	0.30759200	-0.39116100
C	1.86443200	-0.97635900	-0.03903300
C	2.76882000	-1.96575500	0.35219500
C	4.14059400	-1.69367600	0.40003100
H	5.67051000	-0.19580400	0.08449500
H	4.05895300	1.56431000	-0.60805700
H	0.79225200	-1.19498000	-0.07111300
H	2.39534400	-2.95772100	0.62052900
H	4.84714600	-2.47060600	0.70494400
C	1.32721900	1.38596500	-0.76749700
C	0.59180900	1.97772400	0.44703900
H	1.85297800	2.19357000	-1.30556300
H	0.57249200	0.97424100	-1.45697200
C	-0.31039000	3.13666000	0.08093300
H	1.33465900	2.30598000	1.19702500
H	-0.00683000	1.18149400	0.91888800
C	-1.54697300	3.43567600	0.89011400
H	0.22083500	4.01154500	-0.31601300
H	-1.81462300	4.48218600	1.06774200
H	-1.78367400	2.75988800	1.71789200
C	-1.67537000	2.88119200	-0.50616600
H	-2.03286200	3.54692100	-1.29798100
H	-1.99342900	1.83758000	-0.60226200
I	-2.27777500	-1.31819800	-0.03294600
**36***			
Ni	-0.05037600	-0.49259700	1.13503300
P	-0.86582600	-0.10316200	-0.79604400
P	1.74925200	-0.29485600	-0.13091500
C	0.73710100	-0.37180000	-1.68175000
C	-1.43899400	1.58546700	-1.05959800
C	-2.08552100	-1.28489800	-1.40704500
C	2.38949800	1.39810500	-0.00469300
C	3.15631900	-1.42554800	-0.24438500
H	0.70777900	-1.40462400	-2.05951900
H	1.00660300	0.31087700	-2.49947000
C	-0.61160400	2.55351500	-1.65033200
C	-2.71951900	1.94034100	-0.59746600
C	-2.16869700	-2.54317000	-0.79196000
C	-2.88789000	-0.97720000	-2.51726000
C	3.47051100	1.82922600	-0.79014300
C	1.73613600	2.29563900	0.85420400
C	3.40154400	-2.21833200	-1.37519800
C	4.02443400	-1.48889900	0.86061500
C	-1.06970500	3.86466300	-1.79061700
H	0.39087300	2.29844200	-1.99312600
C	-3.16980500	3.25018800	-0.74744400
H	-3.35817800	1.19604700	-0.11721300
C	-3.05325300	-3.49778700	-1.29321900
H	-1.55589800	-2.75980200	0.08555000
C	-3.77381900	-1.93822700	-3.00701800
H	-2.82700600	0.00501000	-2.99035100
C	3.88342100	3.16001100	-0.72113600
H	3.98837100	1.12764400	-1.44862400
C	2.15608900	3.62545300	0.91843700
H	0.89708600	1.95251000	1.46542000
C	4.51391100	-3.06339800	-1.40095600
H	2.73933200	-2.17938300	-2.24206400
C	5.13133500	-2.33522800	0.82642800
H	3.83925300	-0.87079200	1.74343600
C	-2.34601100	4.21319900	-1.34186300
H	-0.42316400	4.61504700	-2.25021700
H	-4.16584700	3.52269500	-0.39237200
C	-3.85591700	-3.19451100	-2.39766900
H	-3.12615600	-4.47545500	-0.81225600
H	-4.40586400	-1.70273900	-3.86604700
C	3.22584200	4.05692900	0.12968800
H	4.72437400	3.49968200	-1.32988800
H	1.64719300	4.32379800	1.58611700
C	5.37689200	-3.12274000	-0.30395900
H	4.70729600	-3.67511200	-2.28481000

H	5.80610400	-2.38031700	1.68400000
H	-2.70091600	5.24042400	-1.45151200
H	-4.55565200	-3.93992800	-2.78236600
H	3.55570800	5.09704100	0.18132000
H	6.24461900	-3.78570500	-0.32812200
I	-2.05869000	-0.45506300	2.51757800
**a**			
Mg	2.24659000	-0.01844900	-0.23917300
O	1.56234300	-1.75886000	-1.05549900
C	0.22728100	-1.88806700	-1.56286600
H	0.22705500	-2.54426000	-2.44801500
H	-0.43249700	-2.30689200	-0.78903800
H	-0.11648700	-0.88955500	-1.85546000
C	2.13349800	-3.00634800	-0.64245000
H	2.14858200	-3.70683800	-1.49302300
H	3.16382200	-2.80545100	-0.31877100
H	1.54856500	-3.42769200	0.18945300
Mg	-2.32163700	0.42709100	0.15400100
O	-3.45127800	1.93169500	0.91544600
C	-4.84762200	1.80932300	1.20012500
H	-5.37000800	2.73534700	0.90996000
H	-5.23403400	0.96601700	0.61275200
H	-5.00315300	1.61586900	2.27470000
C	-2.80817900	3.01102000	1.60410000
H	-2.81096800	2.82462900	2.69074800
H	-1.77588800	3.08169100	1.23743800
H	-3.33715400	3.95371100	1.38885700
O	1.25515500	0.00111000	1.45949300
S	0.04936100	-0.68099600	2.06958200
O	-1.19598600	0.11998800	1.72959100
O	-0.05917300	-2.10512700	1.74075600
C	0.24785300	-0.47437600	3.82243400
H	1.16391000	-1.00452600	4.11508400
H	-0.63108200	-0.91545500	4.31093000
H	0.32906200	0.59858800	4.03851800
S	0.08195500	2.16654800	-1.14993900
O	-1.17526900	1.31215800	-1.17607400
O	1.28591200	1.27299100	-1.37079200
C	-0.01283400	3.15781800	-2.62088500
H	-0.89723000	3.80227700	-2.53080500
H	0.90479900	3.75855900	-2.67357900
H	-0.09776300	2.48880100	-3.48662300
O	0.18173000	3.02997500	0.03062100
I	-3.79963500	-1.53787700	-0.85622100
I	4.89685300	-0.02787900	-0.06559900
**39***			
C	-0.71566100	3.12207300	0.17329900
C	-0.07025100	2.61768100	1.44448900
H	-1.09851700	2.31566400	-0.45808700
C	1.18093000	1.77044500	1.17195900
H	0.20659300	3.48089600	2.08024400
H	-0.80008500	2.02628600	2.01940400
C	1.84122100	1.35798300	2.49018500
H	1.90002500	2.39070300	0.60684500
C	3.02489500	0.39475600	2.33312600
H	1.09031600	0.89176600	3.14682100
H	2.19248100	2.26280500	3.02544300
H	2.68273300	-0.46473500	1.73272900
H	3.82384000	0.88861300	1.75838800
H	-0.06372100	3.77762300	-0.41128400
C	3.57493800	-0.11420600	3.64083700
C	4.74401600	0.41967300	4.20216400
C	2.90063600	-1.12825000	4.34208000
C	5.23059900	-0.04544800	5.42782900
H	5.28104900	1.20953000	3.66872500
C	3.38358600	-1.59630900	5.56565500
H	1.98428400	-1.54989400	3.91892500
C	4.55201200	-1.05611100	6.11372400
H	6.14462400	0.38290700	5.84785800
H	2.84566800	-2.38741500	6.09474500
H	4.93143600	-1.42217300	7.07115100
I	-2.48367400	4.36851600	0.54949500
Mg	-3.14439400	-0.70990200	-0.03192900
O	-4.02283100	0.57997800	1.35809600
C	-5.05353700	1.42488400	0.84583200

H	-4.78187500	1.69739300	-0.18100900
H	-6.01801600	0.89320000	0.84209600
H	-5.12316900	2.33967700	1.45527200
C	-4.24968100	0.19074900	2.71330100
H	-4.35033900	1.08893500	3.34500300
H	-5.15701400	-0.43032500	2.78572400
H	-3.37834300	-0.38668600	3.04443100
Ni	0.77367200	0.16070900	0.03899000
P	0.74480300	-1.58044900	-1.51829000
P	1.77931400	0.92081400	-1.75219900
C	1.11316200	-0.38886900	-2.89205700
C	-0.53176200	-2.74470600	-2.09984500
C	2.25459200	-2.59814300	-1.32177900
C	1.36437000	2.56503700	-2.41384200
C	3.59106200	0.75762800	-1.93468300
H	1.77441000	-0.69584900	-3.71395600
H	0.14754700	-0.05389400	-3.29464900
C	-0.95003100	-2.79760300	-3.43762400
C	-1.11005800	-3.60826100	-1.15523800
C	2.63073700	-2.96079600	-0.01834400
C	3.05724200	-2.98280500	-2.40516800
C	0.16020100	2.78048100	-3.10198300
C	2.17220600	3.66369900	-2.07328800
C	4.31643100	0.03519500	-0.97902800
C	4.24553800	1.24319000	-3.08000700
C	-1.93129900	-3.71169500	-3.82744000
H	-0.51555200	-2.12641100	-4.18121600
C	-2.08609200	-4.52336300	-1.55158900
H	-0.80727100	-3.55608200	-0.10789800
C	3.80205400	-3.69039500	0.19784100
H	2.00903500	-2.66314200	0.82906600
C	4.23081800	-3.70554300	-2.18555500
H	2.77486500	-2.71200400	-3.42502100
C	-0.22591500	4.07625700	-3.44844500
H	-0.50475900	1.94720000	-3.33188200
C	1.77963000	4.95752000	-2.42124600
H	3.10726100	3.51004200	-1.53115400
C	5.68158200	-0.19958300	-1.15976800
H	3.80959900	-0.35703900	-0.10089500
C	5.60971700	1.00857900	-3.25661700
H	3.68979200	1.80495500	-3.83425500
C	-2.49672300	-4.57826000	-2.88704700
H	-2.25455800	-3.74771300	-4.87049500
H	-2.53372000	-5.18577900	-0.80787100
C	4.60661700	-4.05706800	-0.88453600
H	4.08970300	-3.96178200	1.21630500
H	4.85733200	-3.99328600	-3.03325600
C	0.57956000	5.16645400	-3.10726200
H	-1.16990100	4.23405000	-3.97456600
H	2.41169000	5.80606700	-2.14921800
C	6.32938400	0.28692600	-2.29712500
H	6.23367600	-0.76878600	-0.40883300
H	6.11401800	1.38915400	-4.14815700
H	-3.26096600	-5.29522100	-3.19707800
H	5.52893300	-4.61809600	-0.71531800
H	0.26875400	6.18005500	-3.37060500
H	7.39724500	0.10407400	-2.43973000
C	-2.28494800	0.29851400	-1.69385900
H	-2.80043300	1.25833900	-1.88893500
H	-1.22172800	0.54087600	-1.52676500
H	-2.35000000	-0.29205300	-2.62618000
I	-0.69586100	-1.06361100	1.82230900
S	-5.27235000	-2.37612000	0.22989400
O	-6.31858600	-2.28802400	1.25541500
O	-3.84927100	-2.50222000	0.77603000
C	-5.58563200	-3.84528200	-0.73436800
H	-6.59800300	-3.75246500	-1.15003600
H	-4.83820700	-3.90933200	-1.53335900
H	-5.52612400	-4.71195500	-0.06247200
O	-5.19186200	-1.21941100	-0.74174500

TS40*

C	-1.24864400	3.39381700	-0.65105000
C	-0.97795700	2.67073800	0.65208700
H	-1.24138000	2.73714500	-1.52347000
C	0.38637300	1.95691400	0.59243600

H	-0.97155400	3.41484000	1.46937200
H	-1.78958500	1.97285400	0.89324000
C	1.11879400	2.06983800	1.93049600
H	1.00422700	2.50536900	-0.13654500
C	2.53035600	1.47563300	1.93397000
H	0.52772300	1.60881800	2.73726200
H	1.19974200	3.14476400	2.19047100
H	2.44774100	0.39834100	1.72812900
H	3.10829300	1.91698100	1.10545600
H	-0.55423800	4.22512600	-0.82001100
C	3.26578800	1.68396800	3.23327800
C	4.20693300	2.71390600	3.38052900
C	2.99392400	0.86690500	4.34349200
C	4.86136200	2.92374300	4.59805500
H	4.43082300	3.35880900	2.52557600
C	3.64473900	1.07333200	5.56170800
H	2.26146400	0.06116500	4.24374000
C	4.58173700	2.10355100	5.69438600
H	5.59303800	3.73098500	4.69016900
H	3.42024300	0.42521100	6.41310300
H	5.09218900	2.26480500	6.64734700
I	-3.23246900	4.33712000	-0.73987100
Mg	-2.40173900	-0.83574600	0.69088100
O	-3.41394100	0.38870600	2.03826700
C	-4.44009800	1.25856600	1.55847000
H	-4.21538300	1.51135200	0.51724900
H	-5.41874700	0.75627900	1.60553800
H	-4.45463400	2.18223200	2.15941600
C	-3.64334000	-0.04595100	3.38114700
H	-3.74431400	0.83072700	4.04202300
H	-4.55392700	-0.66435600	3.42501300
H	-2.77441100	-0.63726700	3.69288500
Ni	0.27411300	0.08666900	-0.08852300
P	0.64943600	-1.94904300	-1.15120400
P	1.63592100	0.48835600	-1.79837100
C	1.00616100	-1.00617000	-2.70700600
C	-0.51383500	-3.30153100	-1.54634300
C	2.21936000	-2.84997700	-0.82968900
C	1.29636300	1.96693100	-2.81575500
C	3.45790500	0.30365500	-1.88945500
H	1.67410100	-1.44720000	-3.46015500
H	0.04183200	-0.75204200	-3.16677000
C	-1.02468800	-3.52075000	-2.83403100
C	-0.88807300	-4.15815700	-0.49653100
C	2.75783300	-2.87242800	0.46405800
C	2.90326500	-3.49727000	-1.87097900
C	0.24115800	2.00975300	-3.74032000
C	2.01490500	3.14518300	-2.54416100
C	4.17808100	-0.11881200	-0.76581700
C	4.13043200	0.50000200	-3.10822500
C	-1.88036100	-4.60022300	-3.07400200
H	-0.74826900	-2.86107400	-3.65907200
C	-1.73665700	-5.23697200	-0.74276900
H	-0.51611300	-3.97772400	0.51447600
C	3.97048300	-3.52212400	0.71034900
H	2.22924000	-2.37361200	1.27751600
C	4.11975600	-4.13426100	-1.62544800
H	2.48914600	-3.50263100	-2.88196200
C	-0.09187500	3.20823500	-4.37664200
H	-0.34029000	1.11425800	-3.96252500
C	1.67889100	4.34024500	-3.18153000
H	2.84328700	3.12788600	-1.83230200
C	5.55313000	-0.35064800	-0.85617100
H	3.66147300	-0.28021800	0.17753500
C	5.50379400	0.27106700	-3.19529000
H	3.57783500	0.83251000	-3.99018600
C	-2.22930300	-5.46426200	-2.03217300
H	-2.26958200	-4.76887700	-4.08103100
H	-2.02279500	-5.89539600	0.08045400
C	4.65738500	-4.14508000	-0.33359200
H	4.38185600	-3.53050500	1.72259500
H	4.65087400	-4.62501900	-2.44467600
C	0.62143700	4.37613600	-4.09592300
H	-0.91810900	3.22787100	-5.09094700
H	2.24342400	5.24895500	-2.95947000

C	6.21687200	-0.15623200	-2.06895300
H	6.10194800	-0.68699300	0.02612300
H	6.02069700	0.42482500	-4.14576200
H	-2.89426600	-6.31008500	-2.22311100
H	5.61149100	-4.64272000	-0.14306800
H	0.35375300	5.31352500	-4.58884300
H	7.29226200	-0.33698200	-2.13980100
C	-1.81580100	0.11803800	-1.17218900
H	-2.71065800	0.76209000	-1.07671200
H	-1.11410500	0.74512400	-1.73378200
H	-2.10652100	-0.70360600	-1.85112600
I	-0.12956800	-1.21841700	2.37462800
S	-4.63083300	-2.38097100	0.63875900
O	-5.73554200	-2.26495400	1.59863400
O	-3.26164500	-2.64206300	1.26833700
C	-4.98116400	-3.76118800	-0.43434200
H	-5.95008100	-3.56430900	-0.91273700
H	-4.18199000	-3.84116300	-1.17932500
H	-5.03585400	-4.66712100	0.18365100
O	-4.39609300	-1.17992100	-0.25173000

38 (geometric optimization was performed in gas phase)

C	3.03910700	0.87906400	0.06913200
C	2.15021100	2.04813100	-0.30471600
H	2.73840500	-0.04833700	-0.43549300
C	0.72169100	1.87142300	0.22752600
H	2.58591700	2.98224500	0.09818300
H	2.12404800	2.15297800	-1.40033000
C	-0.18529900	3.00841500	-0.23743000
H	0.76399200	1.89969900	1.33182600
C	-1.66344700	2.82658700	0.12968400
H	-0.10778500	3.11759700	-1.32987100
H	0.17107000	3.96483500	0.19524500
H	-1.99475700	1.86395100	-0.29517500
H	-1.76943400	2.75124200	1.22481500
H	3.09174600	0.71258800	1.14921500
C	-2.55075100	3.92234000	-0.40270900
C	-3.03744900	4.94379700	0.42438800
C	-2.88141300	3.95122400	-1.76788500
C	-3.83589400	5.96843700	-0.09313000
H	-2.78761100	4.93424200	1.48947100
C	-3.67816600	4.97225700	-2.28813800
H	-2.50337000	3.15964600	-2.42149800
C	-4.15892500	5.98583600	-1.45202800
H	-4.20697400	6.75587500	0.56820700
H	-3.92661200	4.97782500	-3.35263600
H	-4.78293100	6.78545400	-1.85899200
Ni	0.04030800	0.04689300	-0.27701600
P	0.12340800	-0.77553900	1.74722000
P	-0.65929100	-2.14261600	-0.44776000
C	0.15728000	-2.54668300	1.18444900
C	-1.41540700	-0.67472900	2.72860600
C	1.46858100	-0.49554700	2.94154200
C	-2.44987600	-2.35346300	-0.17399600
C	-0.14218900	-3.42537000	-1.63090200
H	1.19121500	-2.83028500	0.93811100
H	-0.27961000	-3.30289200	1.85258700
C	-1.52689400	-1.31836600	3.97187200
C	-2.53400900	-0.04076500	2.17311600
C	2.65759400	-1.23935500	2.89507300
C	1.37394300	0.59488500	3.82264500
C	-2.99248100	-3.15132500	0.84297900
C	-3.30466300	-1.58266900	-0.98131700
C	-0.84723000	-4.62724100	-1.79462500
C	1.01356300	-3.18231500	-2.38990500
C	-2.75020800	-1.33173700	4.64280200
H	-0.65630600	-1.80905600	4.41437900
C	-3.75750200	-0.05740900	2.84570900
H	-2.44700200	0.44944800	1.20518000
C	3.73402800	-0.89933200	3.71628000
H	2.75968700	-2.07807400	2.20402400
C	2.45325000	0.93328700	4.64048000
H	0.45282000	1.18003300	3.86823900
C	-4.37199100	-3.17510800	1.05651100
H	-2.34166900	-3.74166100	1.48976300
C	-4.68374600	-1.61667500	-0.76995500

H	-2.87899400	-0.94315100	-1.75991900
C	-0.38466300	-5.58789600	-2.69601300
H	-1.76023700	-4.80736300	-1.22286000
C	1.47445500	-4.14956500	-3.28515200
H	1.53021600	-2.22422500	-2.29838500
C	-3.86722100	-0.70410300	4.07829000
H	-2.83438800	-1.83394900	5.60953500
H	-4.62502800	0.43122100	2.39772900
C	3.63546400	0.18923700	4.58751800
H	4.65671100	-1.48145900	3.66704100
H	2.37160600	1.78559100	5.31879000
C	-5.21857700	-2.40750500	0.25219600
H	-4.78514500	-3.78782800	1.86086000
H	-5.34189600	-1.01175100	-1.39791100
C	0.77737500	-5.35156300	-3.43786600
H	-0.93588000	-6.52258600	-2.82379000
H	2.37145300	-3.95517100	-3.87740000
H	-4.82466000	-0.71995200	4.60446600
H	4.48178100	0.45967000	5.22276200
H	-6.29727500	-2.42137400	0.42548300
H	1.13431300	-6.10335600	-4.14591700
I	-0.19137700	0.52226800	-2.79031100
I	5.13592300	1.15057100	-0.51914900

34 (geometric optimization was performed in gas phase)

C	-5.77131500	0.20508600	-0.89201400
C	-4.65641500	0.90553200	-0.42563600
C	-3.71109700	0.28587000	0.40560900
C	-3.91210500	-1.05715100	0.75728600
C	-5.02539400	-1.76230600	0.29367000
C	-5.95908800	-1.13290100	-0.53407500
H	-6.49870600	0.70704100	-1.53491800
H	-4.51758500	1.95354300	-0.70684900
H	-3.18672000	-1.55495600	1.40739600
H	-5.16605000	-2.80687500	0.58254100
H	-6.83174600	-1.68182300	-0.89591300
C	-2.47772600	1.02742800	0.85998800
C	-1.31583300	0.91221000	-0.14184400
H	-2.71526200	2.09436500	1.00746900
H	-2.14583900	0.64067800	1.83753300
C	-0.08281500	1.64484000	0.26274800
H	-1.66737400	1.27682400	-1.13175900
H	-1.08561800	-0.15679500	-0.31523100
C	1.25885000	1.28835200	-0.28797400
H	-0.15582900	2.44328200	1.00886700
H	1.91140900	2.17388000	-0.35693000
H	1.16456200	0.87284800	-1.30578100
C	1.92366300	0.24639100	0.61602800
H	2.12289100	0.63344500	1.62252600
H	1.33449800	-0.67592700	0.68998500
I	3.88326400	-0.40188000	-0.13946500

36 (geometric optimization was performed in gas phase)

Ni	0.33486900	-1.29934700	-0.46222300
P	1.03923800	0.46687000	0.72992400
P	-1.55583400	-0.36857800	0.37719200
C	-0.56530600	0.53069300	1.67840600
C	1.15639900	2.04936500	-0.17323300
C	2.39414300	0.47273700	1.95052900
C	-2.08609400	1.00471700	-0.71966700
C	-3.10281300	-0.92238000	1.17633700
H	-0.44525500	-0.13575400	2.54629200
H	-0.94130700	1.50779400	2.01526500
C	0.46228900	3.21444200	0.17998100
C	1.92531500	2.03548600	-1.34952200
C	3.05740900	-0.74619600	2.15944100
C	2.78439900	1.61347600	2.66864800
C	-3.02188100	1.97087700	-0.32193200
C	-1.43544200	1.13478500	-1.95489600
C	-3.52393200	-0.47177900	2.43663000
C	-3.89510200	-1.85010500	0.47971800
C	0.53685700	4.35034900	-0.62904900
H	-0.16172600	3.23692400	1.07466100
C	2.00941000	3.17592400	-2.14836100
H	2.43296400	1.11460600	-1.64992400
C	4.09114700	-0.82722000	3.09627800
H	2.76619100	-1.61964000	1.56873900

C	3.81736400	1.52784200	3.60296700
H	2.28654500	2.56940000	2.48860700
C	-3.28568500	3.06688500	-1.14467700
H	-3.54309400	1.86723900	0.63329100
C	-1.69985700	2.23301300	-2.77532200
H	-0.70700300	0.37667500	-2.25496300
C	-4.72185800	-0.93637200	2.98723800
H	-2.91864100	0.24474500	2.99597700
C	-5.09399100	-2.30667000	1.02776900
H	-3.56473400	-2.21849300	-0.49557300
C	1.31136500	4.33411700	-1.79170400
H	-0.02108700	5.24832600	-0.35401700
H	2.60768200	3.15461600	-3.06225300
C	4.46858800	0.30750900	3.81906500
H	4.60818100	-1.77660700	3.25378100
H	4.12123400	2.41634400	4.16180000
C	-2.62027700	3.20169300	-2.36864700
H	-4.01239700	3.82041800	-0.83110400
H	-1.17806800	2.33454100	-3.72922700
C	-5.50937900	-1.85079400	2.28340900
H	-5.04063600	-0.58081300	3.97006000
H	-5.70314500	-3.02726600	0.47710100
H	1.36347100	5.22198200	-2.42630100
H	5.28007300	0.24536200	4.54822000
H	-2.82389000	4.06435800	-3.00760000
H	-6.44515500	-2.21320900	2.71529200
I	1.61686800	-2.80789700	-1.94941600

41 (geometric optimization was performed in gas phase)

C	3.09741700	1.01251900	-0.42506800
C	2.19272300	2.18962800	-0.72393700
H	2.78734500	0.10697100	-0.96056300
C	0.78076200	1.93471700	-0.18796300
H	2.62311100	3.10718700	-0.27823400
H	2.16053900	2.36142800	-1.81197000
C	-0.17290600	3.07128100	-0.55429200
H	0.84598500	1.89916900	0.91806400
C	-1.57459300	2.88844100	0.04828900
H	-0.26556700	3.15222400	-1.64902800
H	0.23073200	4.04768900	-0.21619500
H	-1.90751300	1.86645700	-0.19460200
H	-1.50713300	2.94770400	1.14729900
H	3.17261000	0.79688100	0.64520900
C	-2.60150700	3.86896400	-0.45408200
C	-2.92574700	5.02793300	0.26560200
C	-3.24184800	3.65143400	-1.68530500
C	-3.86208200	5.94229800	-0.22537700
H	-2.43679400	5.21364000	1.22630500
C	-4.17898900	4.56048700	-2.18008000
H	-2.99278400	2.75705000	-2.26363100
C	-4.49340700	5.71130900	-1.45026600
H	-4.10052500	6.83913300	0.35239600
H	-4.66625400	4.37094700	-3.14004800
H	-5.22681400	6.42423200	-1.83499000
Ni	0.05399200	0.13432500	-0.68970300
P	0.34932000	-0.95068300	1.30869400
P	-0.99736500	-1.87613400	-0.86531000
C	-0.02982600	-2.61167400	0.54639200
C	-0.89279000	-0.83008900	2.64833900
C	1.95779000	-1.08001200	2.16148700
C	-2.70297800	-1.71496100	-0.22410200
C	-1.05546700	-3.13497000	-2.18602500
H	0.88869900	-3.03439500	0.11377600
H	-0.50341400	-3.36462700	1.19340400
C	-0.89002800	-1.70287300	3.74878100
C	-1.92994000	0.10058400	2.50807900
C	3.02129300	-1.78194600	1.57086000
C	2.20451300	-0.30690500	3.30791300
C	-3.25626100	-2.56915900	0.74076300
C	-3.43714500	-0.59550600	-0.65093800
C	-2.17980900	-3.92992400	-2.45222700
C	0.09225200	-3.26929800	-2.98626000
C	-1.91859000	-1.64549400	4.68992000
H	-0.07992900	-2.42673600	3.86898200
C	-2.95979300	0.15578500	3.45021100
H	-1.93640000	0.76724300	1.64758700

C	4.30289900	-1.71769200	2.11796000
H	2.86111500	-2.36321400	0.66027900
C	3.48922700	-0.24334800	3.85264500
H	1.38992400	0.25236700	3.77288100
C	-4.51776200	-2.30310600	1.27637500
H	-2.69552900	-3.43573300	1.09593900
C	-4.70047900	-0.33303700	-0.11903300
H	-3.00201600	0.08263500	-1.38761500
C	-2.14646000	-4.86323500	-3.49152700
H	-3.08181400	-3.81788600	-1.84723600
C	0.12504900	-4.20956900	-4.01688700
H	0.95609100	-2.62329800	-2.80506000
C	-2.95604800	-0.71720200	4.53989500
H	-1.91335800	-2.32725600	5.54393100
H	-3.76861700	0.87853900	3.32405300
C	4.54103100	-0.94607000	3.25926400
H	5.12266000	-2.25887000	1.64081500
H	3.66924500	0.36465300	4.74234100
C	-5.23928400	-1.18378500	0.85064600
H	-4.93270400	-2.96470800	2.04008500
H	-5.25345300	0.54892500	-0.45034100
C	-0.99504900	-5.00792800	-4.27074000
H	-3.02535300	-5.48011400	-3.69405500
H	1.02150800	-4.30968200	-4.63325400
H	-3.76212500	-0.67733000	5.27658700
H	5.54729400	-0.88664400	3.67964900
H	-6.22061900	-0.97056100	1.28107200
H	-0.97307200	-5.73765300	-5.08359100
I	5.18845800	1.31332000	-1.03624300
C	-0.14328000	0.68007100	-2.55301100
H	-0.01803300	-0.18686300	-3.22594300
H	-1.16643200	1.06805800	-2.71922300
H	0.56265600	1.46161400	-2.87108400

47 (geometric optimization was performed in gas phase)

Ni	-0.07784200	-1.12430000	1.90777100
P	-1.31939900	-0.35890300	0.11975300
P	1.42027500	-0.51261100	0.30730500
C	0.11699200	-0.76523300	-1.00159200
C	-1.70093600	1.40402600	-0.18159200
C	-2.75396900	-1.27469200	-0.54812800
C	1.73555200	1.29446800	0.18978700
C	2.96830000	-1.24407800	-0.34092900
H	0.04699700	-1.84108800	-1.22115500
H	0.22447000	-0.21042700	-1.94567600
C	-1.23322200	2.14585500	-1.27528900
C	-2.43921000	2.05892500	0.81964500
C	-3.00079800	-2.54181900	0.00434000
C	-3.59328900	-0.77894600	-1.55708500
C	2.43111300	1.87951100	-0.87838400
C	1.16142800	2.11402800	1.17211400
C	3.10108300	-1.73195000	-1.64915300
C	4.06708500	-1.32062600	0.53138100
C	-1.49246600	3.51530000	-1.36417300
H	-0.64320000	1.66611900	-2.05682700
C	-2.71016000	3.42390800	0.72292600
H	-2.78609600	1.49220300	1.68824000
C	-4.06458800	-3.31466900	-0.46640800
H	-2.36033400	-2.89969000	0.81493400
C	-4.65933600	-1.55304800	-2.02007000
H	-3.41521900	0.21451700	-1.97514100
C	2.53140100	3.26884700	-0.97071600
H	2.89303500	1.24629400	-1.64011900
C	1.25892200	3.50336200	1.07671700
H	0.61645600	1.64809500	1.99676600
C	4.31266300	-2.28244500	-2.07864400
H	2.25842500	-1.68390000	-2.34177600
C	5.27763600	-1.86226300	0.09937400
H	3.96794700	-0.95480200	1.55732100
C	-2.23158700	4.15718600	-0.36774100
H	-1.10721400	4.08376600	-2.21375200
H	-3.28483800	3.92055000	1.50845500
C	-4.89361000	-2.82164800	-1.47850900
H	-4.25413900	-4.29929500	-0.03227700
H	-5.31235500	-1.16445900	-2.80552500
C	1.94083300	4.08169900	0.00352700

H	3.07133900	3.72118000	-1.80637600
H	0.79326800	4.13380200	1.83725500
C	5.40257600	-2.34599700	-1.20755800
H	4.40422300	-2.66070600	-3.09974700
H	6.12569700	-1.91444500	0.78634200
H	-2.42929300	5.22952700	-0.43670300
H	-5.73050200	-3.42344600	-1.84137600
H	2.01535200	5.16904500	-0.07495300
H	6.34879900	-2.77599100	-1.54434400
C	-1.03942500	-1.96440800	3.40249500
H	-1.66645100	-2.83026600	3.11373100
H	-0.34786400	-2.33535800	4.18485600
H	-1.71998400	-1.24988000	3.90638100

S18

Mg	2.92588300	0.81984100	-0.02721500
O	3.66731000	2.25389600	1.29802800
C	4.07510700	3.48649300	0.71216700
H	3.39705700	3.69479300	-0.12586100
H	5.10840100	3.41709600	0.33598900
H	3.99685500	4.29883100	1.45447100
C	4.45760600	1.86966900	2.42317500
H	4.44645700	2.67310900	3.17847000
H	5.49215500	1.65319200	2.11135800
H	4.00347200	0.96697400	2.84944300
Ni	-0.73211800	0.65526400	0.76965600
P	-0.80447900	-1.13599100	-0.68125000
P	-2.46278800	0.98362800	-0.47428400
C	-1.91755100	-0.17149300	-1.82202900
C	0.51573800	-1.89052300	-1.67966900
C	-1.84438500	-2.52713800	-0.11235400
C	-2.93838200	2.57499500	-1.22583800
C	-3.98027400	0.26747000	0.24036100
H	-2.67890000	-0.71107100	-2.40301700
H	-1.28061200	0.42000600	-2.49718800
C	0.50827500	-1.88432200	-3.08226200
C	1.59327400	-2.47337800	-0.99173400
C	-2.26390500	-2.54064400	1.22489100
C	-2.27951400	-3.53270600	-0.98966900
C	-1.91630300	3.38869900	-1.74430500
C	-4.26929400	3.01505800	-1.26999100
C	-4.19797100	0.42046700	1.61898200
C	-4.89852700	-0.46194600	-0.52892900
C	1.56485500	-2.46377100	-3.78928900
H	-0.31676300	-1.42314200	-3.62927800
C	2.64419900	-3.05269700	-1.70312500
H	1.61780800	-2.45812500	0.10019200
C	-3.12670500	-3.54183100	1.67773400
H	-1.91212500	-1.76335200	1.90581400
C	-3.14185300	-4.53050200	-0.53437000
H	-1.94194800	-3.53600000	-2.02928300
C	-2.23103400	4.61829000	-2.32305000
H	-0.87594800	3.05853400	-1.68964200
C	-4.57624000	4.25098300	-1.84581300
H	-5.06641200	2.39576500	-0.85497600
C	-5.32646600	-0.14317500	2.21649800
H	-3.47302100	0.96960400	2.22313900
C	-6.01985500	-1.03444700	0.07467900
H	-4.73856400	-0.59379600	-1.60069900
C	2.63160800	-3.05031800	-3.10152900
H	1.55480400	-2.45536900	-4.88189900
H	3.48057600	-3.49366200	-1.15757100
C	-3.56962600	-4.53279200	0.79866600
H	-3.45494900	-3.54319700	2.71955400
H	-3.48111600	-5.31120700	-1.21971000
C	-3.56042400	5.05136700	-2.37461900
H	-1.43258500	5.24492700	-2.72734000
H	-5.61468700	4.58938300	-1.87831200
C	-6.23532700	-0.87523600	1.44679400
H	-5.48893600	-0.02129800	3.28994900
H	-6.72489500	-1.61139100	-0.52833600
H	3.45615200	-3.50290800	-3.65798200
H	-4.24632200	-5.31458200	1.15245700
H	-3.80350900	6.01787900	-2.82278400
H	-7.11104300	-1.32797600	1.91807600
C	1.48192900	1.55205000	-1.40590500

H	1.77815300	2.53829000	-1.81464600
H	0.48862100	1.69326300	-0.93992000
H	1.34676100	0.88336700	-2.27592400
I	1.21343000	-0.24300300	2.29976900
S	5.43857800	-0.13058900	-0.48369200
O	6.65323100	0.09909300	0.30669400
O	4.27082000	-0.73537900	0.29880400
C	5.84786900	-1.29746800	-1.77267500
H	6.65901300	-0.85491400	-2.36643300
H	4.95975700	-1.47100100	-2.39185600
H	6.18807900	-2.22612100	-1.29526800
O	4.84197400	1.08187800	-1.16073900
C	-0.98682100	2.29435700	1.79046000
H	-1.04981800	2.05714000	2.86564600
H	-1.85121600	2.92096100	1.51583800
H	-0.06878600	2.88560700	1.62226500

TS-S19

Mg	2.52038900	0.84980800	0.19450100
O	3.05352300	2.33879800	1.54412200
C	3.08117200	3.66735900	1.03335700
H	2.22851700	3.77613500	0.35136600
H	4.01953100	3.85509200	0.48602500
H	2.97659500	4.38905900	1.86058700
C	4.05996100	2.07224300	2.52010300
H	3.98919200	2.80690800	3.33920800
H	5.06311200	2.10716400	2.06441700
H	3.87128800	1.06781400	2.91962500
Ni	-0.39878500	0.66173400	0.58548000
P	-0.62432000	-1.16563700	-0.76958600
P	-2.25111700	0.97263500	-0.52082800
C	-1.79240700	-0.21757400	-1.86757900
C	0.66804100	-1.91980300	-1.80858700
C	-1.62995500	-2.58336700	-0.19580700
C	-2.82830400	2.52693400	-1.28368200
C	-3.72651600	0.26480400	0.28829700
H	-2.59816100	-0.76957200	-2.37286200
H	-1.19550000	0.33634200	-2.60615100
C	0.65451000	-1.88192600	-3.21016700
C	1.73739300	-2.53885200	-1.13955600
C	-2.01924100	-2.64886500	1.14798500
C	-2.06313100	-3.57252100	-1.09392400
C	-2.14800800	3.07654100	-2.38399800
C	-3.89919300	3.23509100	-0.71407300
C	-3.75551400	0.18375800	1.68752800
C	-4.80576900	-0.22995100	-0.45993400
C	1.69637500	-2.46731900	-3.93542600
H	-0.16660600	-1.39762300	-3.74304100
C	2.77169200	-3.12575500	-1.86821800
H	1.76503000	-2.54789500	-0.04740100
C	-2.84774400	-3.68506100	1.58719600
H	-1.66846000	-1.88888100	1.84666300
C	-2.88933800	-4.60599200	-0.65222200
H	-1.75045900	-3.53598900	-2.14062100
C	-2.54864400	4.30329500	-2.91576400
H	-1.29355200	2.55728700	-2.82179500
C	-4.29192400	4.46482000	-1.24701400
H	-4.43095200	2.82239100	0.14538000
C	-4.85658900	-0.38431400	2.33298000
H	-2.90484700	0.55095200	2.26473700
C	-5.89716500	-0.80999900	0.18723400
H	-4.79134500	-0.16652700	-1.55075600
C	2.75309500	-3.09209500	-3.26652400
H	1.68076300	-2.43556200	-5.02761700
H	3.60109000	-3.59811200	-1.33766400
C	-3.28569000	-4.66073200	0.68939500
H	-3.15134900	-3.72610600	2.63578900
H	-3.22477900	-5.37289300	-1.35481400
C	-3.62111200	4.99967300	-2.35001200
H	-2.01569300	4.71886700	-3.77427400
H	-5.12823700	5.00573100	-0.79765100
C	-5.92347100	-0.88707200	1.58429800
H	-4.87286000	-0.44620900	3.42359100
H	-6.73015000	-1.20520200	-0.39912700
H	3.56628100	-3.54857400	-3.83622400
H	-3.93391100	-5.47030600	1.03404400

H	-3.93128600	5.96070000	-2.76726300
H	-6.77836100	-1.34392500	2.08881600
C	1.22013500	1.61020600	-1.33670700
H	1.89747600	2.43660800	-1.63190300
H	0.23205000	2.08023300	-1.23573300
H	1.18718700	0.93012400	-2.20656600
I	1.08291100	-0.50493300	2.43263700
S	5.09094800	0.19621300	-0.45967100
O	6.32907300	0.38973600	0.30144200
O	4.00004800	-0.58421600	0.28049600
C	5.48864800	-0.75812200	-1.91588400
H	6.22532600	-0.18268700	-2.49271700
H	4.57320700	-0.91957500	-2.49735400
H	5.92199200	-1.71235300	-1.58860800
O	4.39230000	1.44924500	-0.93115900
C	-0.55465600	2.32346200	1.57373000
H	-1.28594300	2.24797100	2.39697800
H	-0.85123100	3.16700300	0.92733300
H	0.42900700	2.54076300	2.01793600
**S20**			
Ni	-0.00612300	-0.89636600	1.75561600
P	-1.33713300	-0.39040300	0.00943100
P	1.37116500	-0.35289900	0.04012300
C	0.03635900	-0.65955500	-1.22198300
C	-1.79421500	1.37389300	-0.13542100
C	-2.78096900	-1.33589700	-0.59338300
C	1.71226200	1.43484100	-0.15630400
C	2.89585100	-1.19165800	-0.51605400
H	0.01464800	-1.73149700	-1.46911500
H	0.06150300	-0.07673000	-2.15485600
C	-1.56510200	2.14769000	-1.28192300
C	-2.32881300	1.98574100	1.01101600
C	-2.89994200	-2.66419100	-0.15219600
C	-3.75031300	-0.80271500	-1.45613600
C	2.26685700	1.96769300	-1.33110500
C	1.30349600	2.29762600	0.87012800
C	2.83960300	-2.43848400	-1.15972700
C	4.14988500	-0.65316700	-0.18094000
C	-1.86172800	3.51274400	-1.28186300
H	-1.13840400	1.69319400	-2.17738600
C	-2.63668400	3.34698500	1.00478200
H	-2.48486700	1.39264000	1.91571200
C	-3.96303500	-3.45693000	-0.59043000
H	-2.16144300	-3.06657200	0.54670100
C	-4.81484700	-1.59726100	-1.88771300
H	-3.67549300	0.23507000	-1.78808000
C	2.40146300	3.34871500	-1.47739300
H	2.59217500	1.29962600	-2.13267600
C	1.43568100	3.68053900	0.71963700
H	0.86761300	1.87765500	1.77917400
C	4.01422500	-3.12153200	-1.48165800
H	1.87638600	-2.88759200	-1.41113200
C	5.32218600	-1.34077700	-0.50084100
H	4.21066500	0.31003500	0.33023300
C	-2.39777300	4.11408100	-0.13989300
H	-1.66525700	4.10957100	-2.17571600
H	-3.05054500	3.81400300	1.90179300
C	-4.92070700	-2.92440700	-1.45880900
H	-4.05051200	-4.48913300	-0.24266800
H	-5.56750500	-1.17760200	-2.55990400
C	1.98174300	4.20635200	-0.45332900
H	2.83198900	3.75994100	-2.39394100
H	1.10368100	4.34695800	1.51889500
C	5.25838600	-2.57464400	-1.15464500
H	3.95576600	-4.08789100	-1.98849000
H	6.29076800	-0.90915800	-0.23677900
H	-2.62345700	5.18330100	-0.13948400
H	-5.75699700	-3.54208900	-1.79555600
H	2.08215600	5.28800700	-0.57296900
H	6.17652400	-3.11119200	-1.40572500
C	-1.36497400	-1.34464200	3.07048100
H	-1.18280700	-2.34667100	3.50046500
H	-1.32235400	-0.62748900	3.91215400
H	-2.39806000	-1.33558600	2.67701400
C	1.38816300	-1.25654100	3.05951600

H	1.03922800	-1.49098100	4.07940200
H	1.99358400	-2.11531200	2.70734000
H	2.07964200	-0.39371100	3.13214100

TS-S21

Ni	-0.02781700	-1.04573800	1.81367700
P	-1.37776600	-0.40537200	0.15374800
P	1.39190500	-0.37371800	0.19844000
C	0.03035200	-0.69516200	-1.04208600
C	-1.76280000	1.38311300	-0.01145800
C	-2.82090000	-1.22502500	-0.64796600
C	1.70048400	1.42994900	-0.04934600
C	2.90911400	-1.10098700	-0.54466800
H	0.02540300	-1.77124300	-1.27160000
H	0.05054400	-0.12960300	-1.98738900
C	-1.56211700	2.11333300	-1.19185500
C	-2.22965200	2.05164500	1.13092000
C	-2.79870400	-2.62815400	-0.75015200
C	-3.96137800	-0.53404000	-1.08557100
C	2.32961000	1.94682800	-1.19251700
C	1.21978300	2.31429900	0.92570600
C	2.93029800	-1.78153500	-1.77166200
C	4.10059100	-1.00610600	0.19704900
C	-1.80766900	3.48732500	-1.22455900
H	-1.19920300	1.61194900	-2.09067700
C	-2.49221200	3.42277400	1.09502800
H	-2.36850600	1.48981100	2.05877400
C	-3.87805600	-3.31935800	-1.30165800
H	-1.93056900	-3.18643000	-0.38790300
C	-5.04616800	-1.22872400	-1.62904300
H	-4.00246700	0.55381200	-1.00598600
C	2.45980800	3.32677800	-1.36097600
H	2.71707400	1.26664300	-1.95506800
C	1.34514500	3.69502800	0.75609200
H	0.72302000	1.90590600	1.80785700
C	4.11725000	-2.34719600	-2.24965100
H	2.01931600	-1.86984300	-2.36720400
C	5.28545100	-1.56232500	-0.28393900
H	4.09390500	-0.48921900	1.16133200
C	-2.27317800	4.14479700	-0.08165700
H	-1.62833200	4.04854100	-2.14481600
H	-2.85450600	3.93159400	1.99188000
C	-5.00712800	-2.62067900	-1.74328700
H	-3.84216300	-4.40901500	-1.37965800
H	-5.92617600	-0.67599300	-1.96832300
C	1.96449500	4.20308500	-0.38849500
H	2.94885100	3.72194300	-2.25527400
H	0.95315400	4.37401500	1.51683700
C	5.29717700	-2.23724200	-1.51045300
H	4.11825500	-2.87276100	-3.20818000
H	6.20405500	-1.47502100	0.30199200
H	-2.46031500	5.22120500	-0.10738200
H	-5.85521500	-3.16140500	-2.17055900
H	2.06358100	5.28302500	-0.52459200
H	6.22381800	-2.67848100	-1.88563800
C	-0.57289800	-2.70642600	2.71083200
H	0.00542400	-3.49670300	3.20993800
H	-1.58202500	-2.66407800	3.14778800
H	-0.63933800	-3.00043000	1.63610600
C	0.59155400	-1.36286400	3.60000500
H	0.04231400	-1.51705100	4.54011600
H	1.60635600	-1.77718500	3.70253500
H	0.65102000	-0.24575900	3.44151000

S22

C	0.65942400	-2.20469500	3.35762600
H	0.05667000	-2.60303500	2.48211200
H	0.04155100	-2.44230800	4.23825400
H	0.76136400	-1.08797900	3.32212500
C	2.03485800	-2.85413100	3.43396800
H	1.96019700	-3.95375700	3.44380300
H	2.64396300	-2.56249700	2.56491200
H	2.57120800	-2.54831800	4.34754200
Ni	0.01049500	-1.06211900	1.61690800
P	-1.38199400	-0.46086800	0.15908200
P	1.37521500	-0.31466400	0.09261700
C	-0.01998600	-0.61232800	-1.12672000

C	-1.96757300	1.27986100	0.03178600
C	-2.78747700	-1.40179100	-0.58657800
C	1.51503000	1.52532600	0.08413200
C	2.90790100	-0.78197800	-0.82098000
H	-0.00148700	-1.67471400	-1.41352700
H	-0.05641900	-0.00199800	-2.04432000
C	-1.60895300	2.15935600	-0.99971500
C	-2.76122400	1.76342500	1.08594100
C	-2.93837200	-2.74055600	-0.19583600
C	-3.68203500	-0.85857900	-1.52163600
C	2.05227000	2.25746000	-0.98553800
C	0.98301400	2.21411200	1.18379600
C	2.90771200	-1.41519800	-2.07350500
C	4.14154900	-0.55998000	-0.18059700
C	-2.02605000	3.49284100	-0.97393100
H	-0.98166000	1.81587500	-1.82195000
C	-3.19157100	3.09058100	1.10484300
H	-3.03023900	1.09035800	1.90531500
C	-3.95180500	-3.53177000	-0.74192600
H	-2.25408500	-3.15010500	0.55361700
C	-4.70162100	-1.64672100	-2.06169800
H	-3.58456000	0.18576900	-1.82682700
C	2.04170500	3.65394800	-0.95836100
H	2.47600800	1.73353300	-1.84596800
C	0.96999200	3.60968300	1.21189800
H	0.55219100	1.63179100	2.00191900
C	4.10920500	-1.80906100	-2.67340700
H	1.96605300	-1.60068200	-2.59431600
C	5.33941000	-0.94357300	-0.78162400
H	4.15664100	-0.07802300	0.80194400
C	-2.81898300	3.96229100	0.07577400
H	-1.72062200	4.16901600	-1.77615300
H	-3.80985600	3.45024000	1.93144600
C	-4.83682400	-2.98454300	-1.67600900
H	-4.05827400	-4.57419000	-0.43044000
H	-5.39541600	-1.21431100	-2.78756900
C	1.49762500	4.33214500	0.13824000
H	2.45759800	4.21738600	-1.79789200
H	0.53733700	4.13330800	2.06773000
C	5.32741700	-1.57315900	-2.03279400
H	4.09047100	-2.29876900	-3.65072900
H	6.28825100	-0.75548200	-0.27223600
H	-3.14095800	5.00643900	0.09687500
H	-5.63646000	-3.59813200	-2.09875400
H	1.48555000	5.42495200	0.15431100
H	6.26497200	-1.87985500	-2.50303100
**Me–Me**			
C	0.00000000	0.00000000	0.76286800
H	0.51302000	0.88862100	1.16715400
H	-1.02607800	-0.00002200	1.16715400
H	0.51305800	-0.88859900	1.16715400
C	0.00000000	0.00000000	-0.76286800
H	-0.51302000	0.88862100	-1.16715400
H	1.02607800	-0.00002200	-1.16715400
H	-0.51305800	-0.88859900	-1.16715400

VIII. ^{1}H, ^{13}C NMR Spectra

1H spectrum

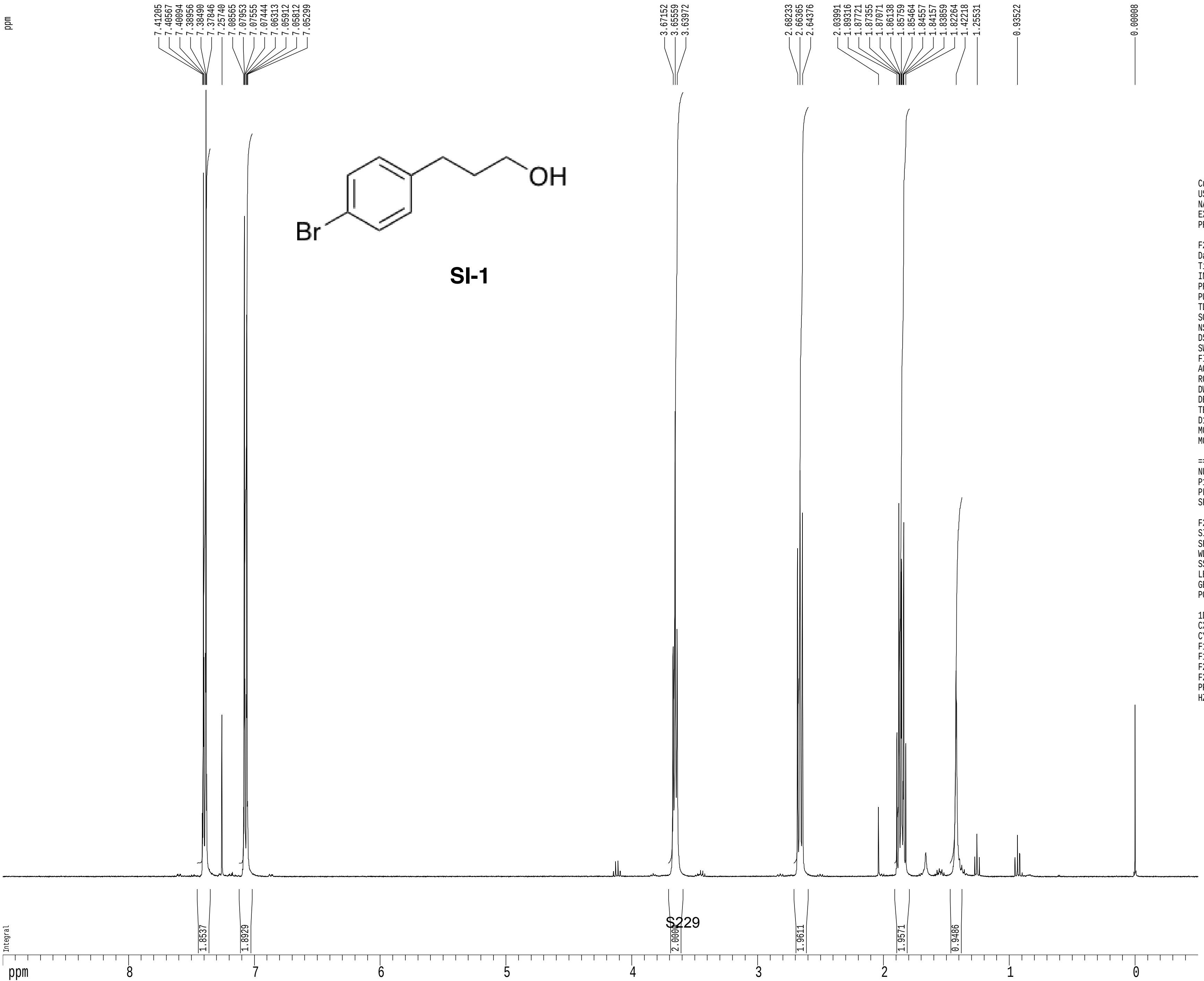


```
Current Data Parameters
USER          sanforda
NAME     ABS-2-049-pure
EXPNO                1
PROCNO               1

F2 - Acquisition Parameters
Date_         20181009
Time             11.25
INSTRUM         drx400
PROBHD   5 mm QNP H/F/P
PULPROG           zg30
TD               65536
SOLVENT          CDCl3
NS                   8
DS                   2
SWH           6410.256 Hz
FIDRES        0.097813 Hz
AQ           5.1118579 sec
RG                 181
DW              78.000 usec
DE                4.50 usec
TE               298.0 K
D1          0.10000000 sec
MCREST      0.00000000 sec
MCWRK       0.01500000 sec

======== CHANNEL f1 ========
NUC1                1H
P1               12.00 usec
PL1              -1.10 dB
SFO1       400.1328009 MHz

F2 - Processing parameters
SI               65536
SF         400.1300221 MHz
WDW                 no
SSB                  0
LB                0.00 Hz
GB                   0
PC                2.00

1D NMR plot parameters
CX               22.80 cm
CY               15.00 cm
F1P              9.000 ppm
F1             3601.17 Hz
F2P             -0.500 ppm
F2             -200.06 Hz
PPMCM          0.41667 ppm/cm
HZCM         166.72086 Hz/cm
```

1H spectrum

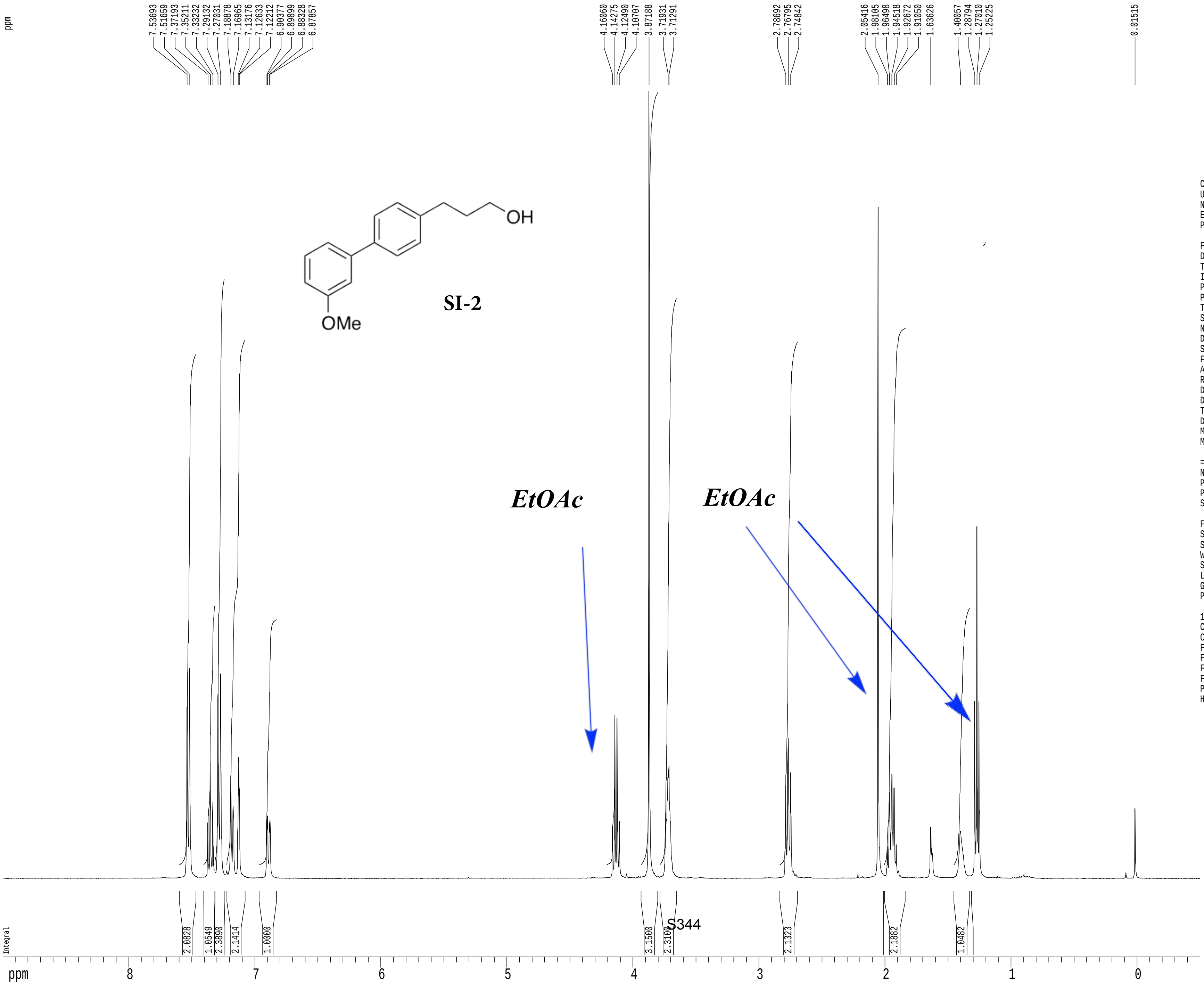

Current Data Parameters
USER mcginnit
NAME tmm-1-148-24
EXPNO 1
PROCNO 1

F2 - Acquisition Parameters
Date_ 20190801
Time 9.46
INSTRUM drx400
PROBHD 5 mm QNP H/F/P
PULPROG zg30
TD 65536
SOLVENT CDCl3
NS 8
DS 2
SWH 6410.256 Hz
FIDRES 0.097813 Hz
AQ 5.1118579 sec
RG 256
DW 78.000 usec
DE 4.50 usec
TE 298.0 K
D1 0.10000000 sec
MCREST 0.00000000 sec
MCWRK 0.01500000 sec

======== CHANNEL f1 ========
NUC1 1H
P1 12.00 usec
PL1 -1.10 dB
SFO1 400.1328009 MHz

F2 - Processing parameters
SI 65536
SF 400.1300175 MHz
WDW EM
SSB 0
LB 0.30 Hz
GB 0
PC 2.00

1D NMR plot parameters
CX 22.80 cm
CY 15.00 cm
F1P 9.000 ppm
F1 3601.17 Hz
F2P -0.500 ppm
F2 -200.06 Hz
PPMCM 0.41667 ppm/cm
HZCM 166.72084 Hz/cm

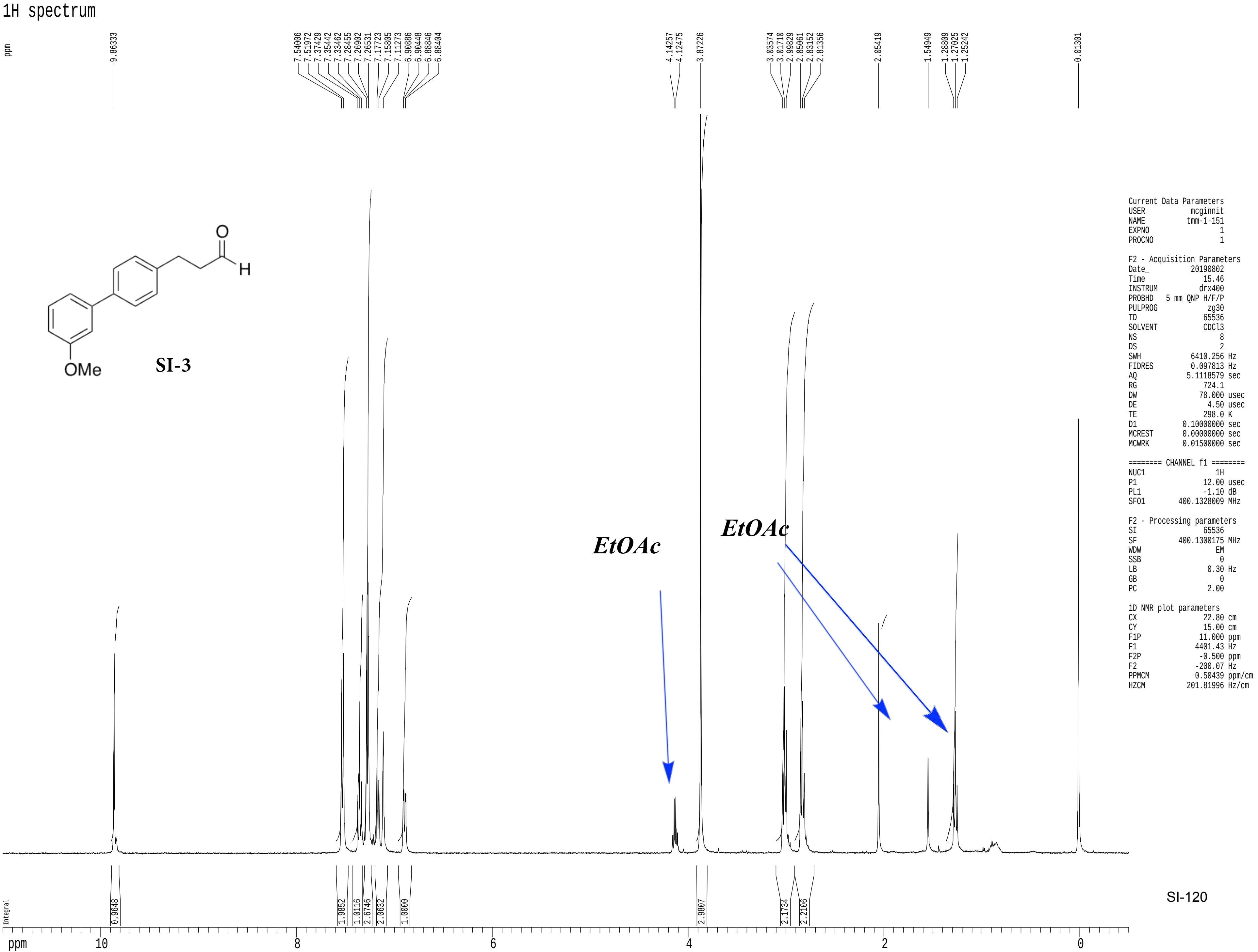
1H spectrum
9.86333
7.54006
7.51972
7.37429
7.35442
7.33462
7.28455
7.26902
7.26531
7.17723
7.15805
7.11273
6.90886
6.90448
6.88846
6.88404
4.14257
4.12475
3.87226
3.03574
3.01710
2.99829
2.85061
2.83152
2.81356
2.05419
1.54949
1.28809
1.27025
1.25242
0.01301
OMe
O
H
SI-3
EtOAc
EtOAc
Current Data Parameters
USER mcginnit
NAME tmm-1-151
EXPNO 1
PROCNO 1
F2 - Acquisition Parameters
Date_ 20190802
Time 15.46
INSTRUM drx400
PROBHD 5 mm QNP H/F/P
PULPROG zg30
TD 65536
SOLVENT CDCl3
NS 8
DS 2
SWH 6410.256 Hz
FIDRES 0.097813 Hz
AQ 5.1118579 sec
RG 724.1
DW 78.000 usec
DE 4.50 usec
TE 298.0 K
D1 0.10000000 sec
MCREST 0.00000000 sec
MCWRK 0.01500000 sec
======== CHANNEL f1 ========
NUC1 1H
P1 12.00 usec
PL1 -1.10 dB
SFO1 400.1328009 MHz
F2 - Processing parameters
SI 65536
SF 400.1300175 MHz
WDW EM
SSB 0
LB 0.30 Hz
GB 0
PC 2.00
1D NMR plot parameters
CX 22.80 cm
CY 15.00 cm
F1P 11.000 ppm
F1 4401.43 Hz
F2P -0.500 ppm
F2 -200.07 Hz
PPMCM 0.50439 ppm/cm
HZCM 201.81996 Hz/cm
Integral
0.9648
1.9852
1.0116
2.6746
2.0632
1.0000
2.9807
2.1734
2.2106
ppm
10
8
6
4
2
0

1H spectrum

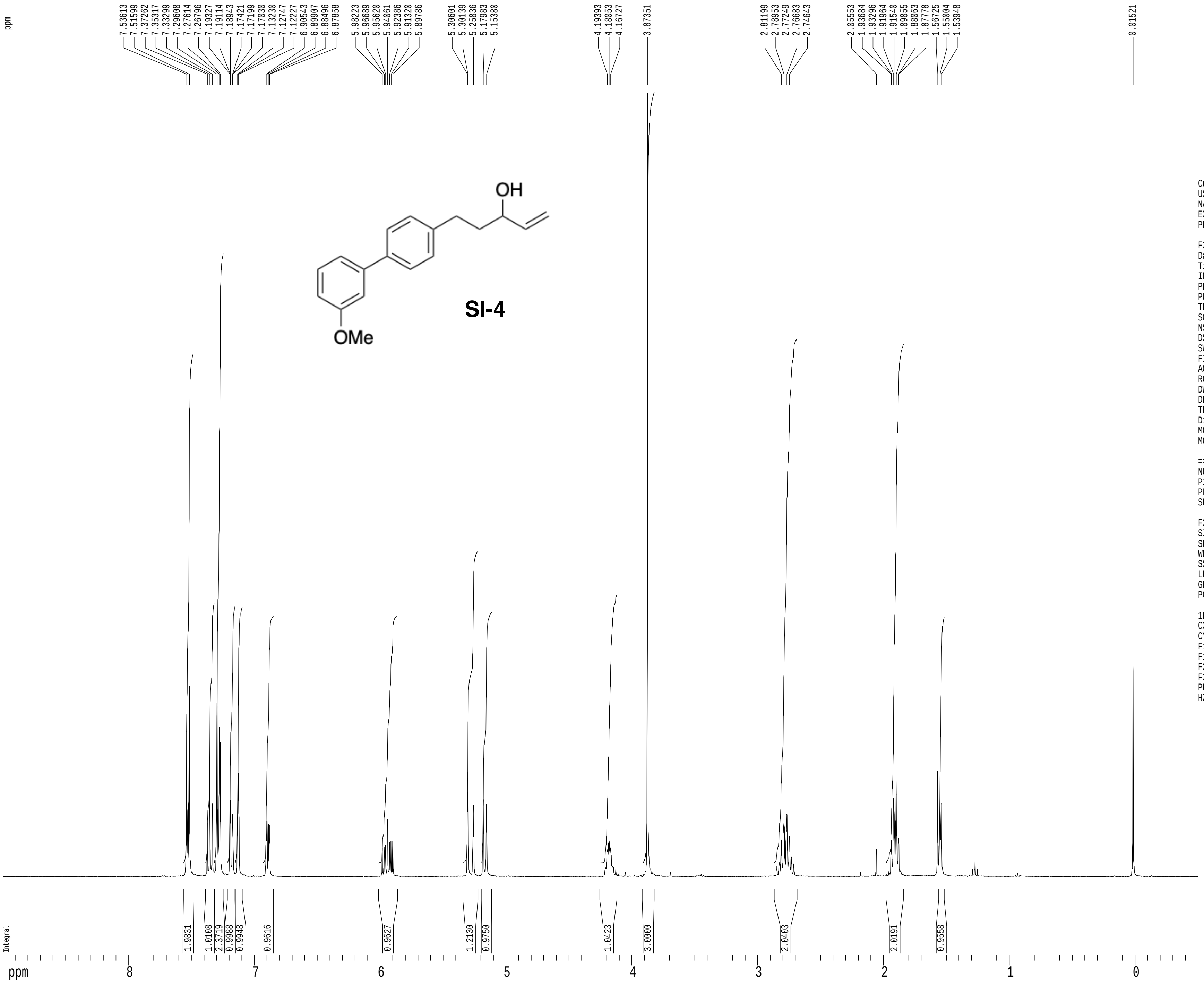

Current Data Parameters
USER mcginnit
NAME tmm-1-072
EXPNO 1
PROCNO 1

F2 - Acquisition Parameters
Date_ 20190416
Time 17.40
INSTRUM drx400
PROBHD 5 mm QNP H/F/P
PULPROG zg30
TD 65536
SOLVENT CDCl3
NS 8
DS 2
SWH 6410.256 Hz
FIDRES 0.097813 Hz
AQ 5.1118579 sec
RG 322.5
DW 78.000 usec
DE 4.50 usec
TE 298.0 K
D1 0.10000000 sec
MCREST 0.00000000 sec
MCWRK 0.01500000 sec

======== CHANNEL f1 ========
NUC1 1H
P1 12.00 usec
PL1 -1.10 dB
SFO1 400.1328009 MHz

F2 - Processing parameters
SI 65536
SF 400.1300175 MHz
WDW EM
SSB 0
LB 0.30 Hz
GB 0
PC 2.00

1D NMR plot parameters
CX 22.80 cm
CY 15.00 cm
F1P 9.000 ppm
F1 3601.17 Hz
F2P -0.500 ppm
F2 -200.06 Hz
PPMCM 0.41667 ppm/cm
HZCM 166.72084 Hz/cm

1H spectrum

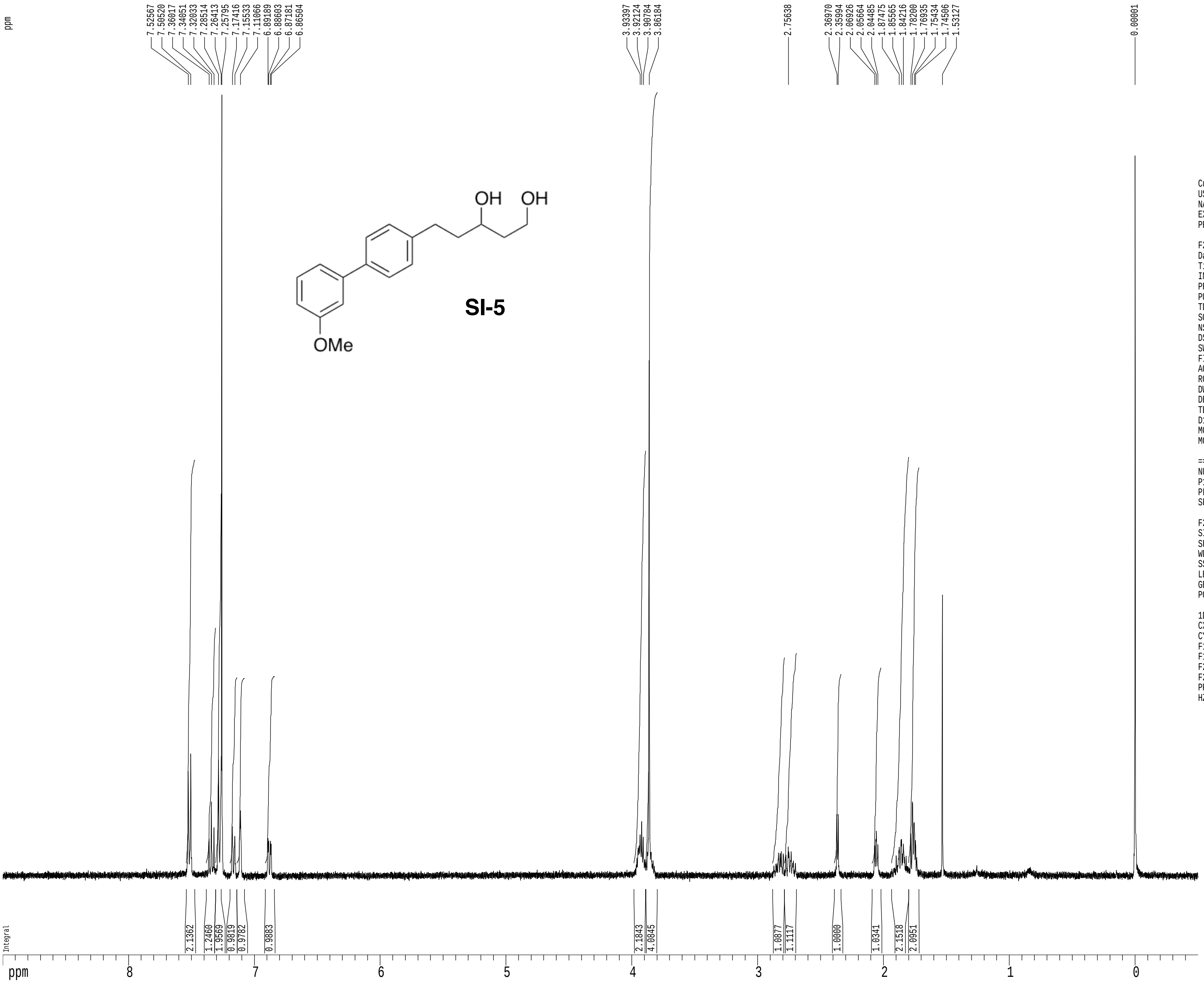


```
Current Data Parameters
USER          sanforda
NAME     ABS-1-279-pure
EXPNO                1
PROCNO               1

F2 - Acquisition Parameters
Date_          20180814
Time              16.05
INSTRUM          drx400
PROBHD   5 mm QNP H/F/P
PULPROG            zg30
TD                65536
SOLVENT           CDCl3
NS                    8
DS                    2
SWH            6410.256 Hz
FIDRES         0.097813 Hz
AQ            5.1118579 sec
RG               1290.2
DW               78.000 usec
DE                 4.50 usec
TE                298.0 K
D1           0.10000000 sec
MCREST       0.00000000 sec
MCWRK        0.01500000 sec

======== CHANNEL f1 ========
NUC1                 1H
P1                12.00 usec
PL1               -1.10 dB
SFO1        400.1328009 MHz

F2 - Processing parameters
SI                65536
SF          400.1300222 MHz
WDW                  no
SSB                   0
LB                 0.00 Hz
GB                    0
PC                 2.00

1D NMR plot parameters
CX                22.80 cm
CY                15.00 cm
F1P               9.000 ppm
F1              3601.17 Hz
F2P              -0.500 ppm
F2              -200.06 Hz
PPMCM           0.41667 ppm/cm
HZCM          166.72086 Hz/cm
```

1H spectrum

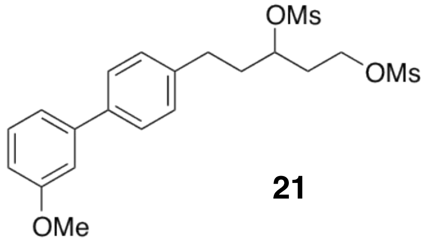

21

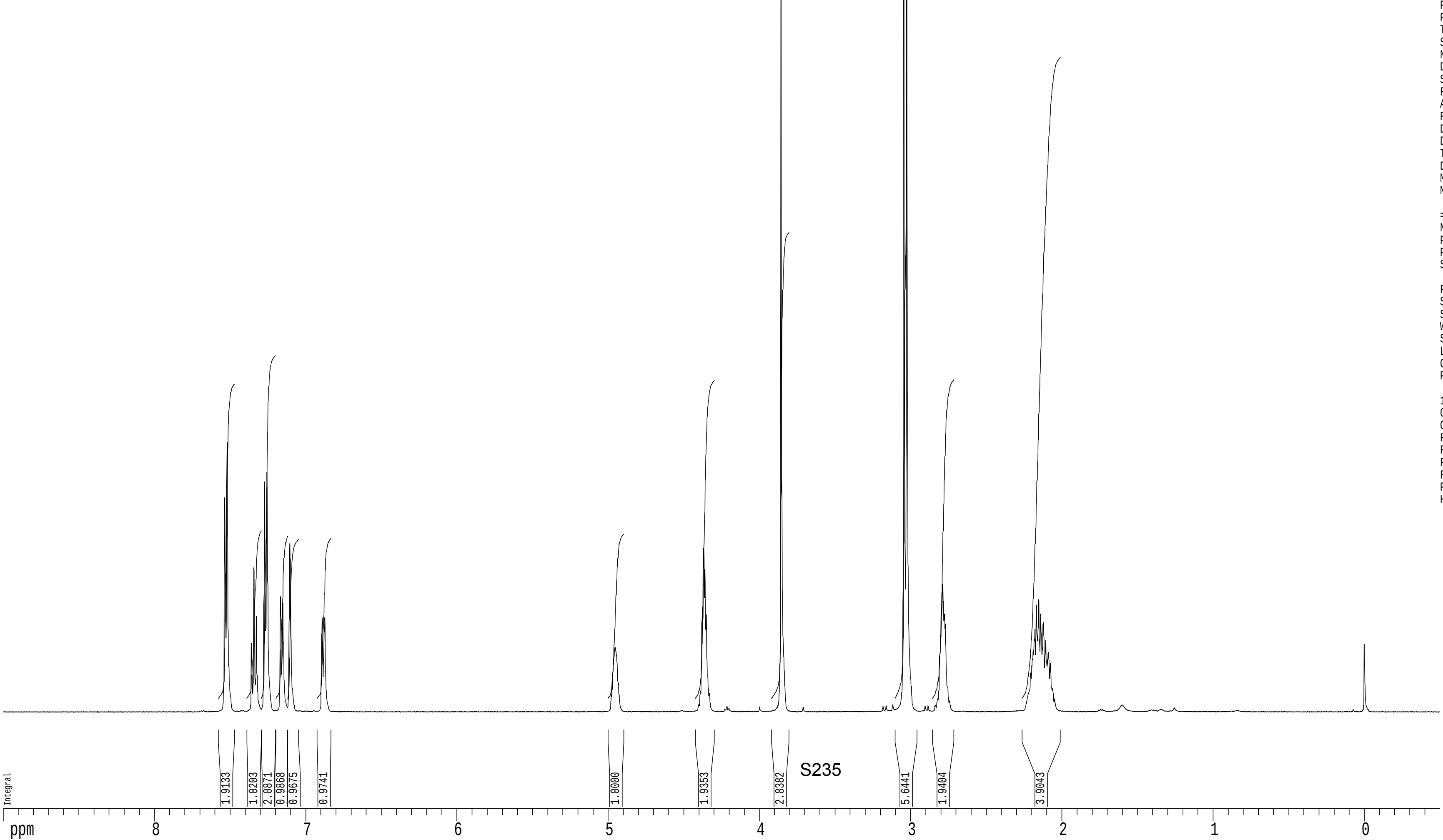


```
Current Data Parameters
USER            sanforda
NAME     ABS-2-025-proton
EXPNO                  1
PROCNO                 1

F2 - Acquisition Parameters
Date_           20181017
Time               15.15
INSTRUM         cryo500
PROBHD   5 mm CPTCI 1H-
PULPROG             zg30
TD                 81728
SOLVENT            CDCl3
NS                     8
DS                     2
SWH            8012.820 Hz
FIDRES         0.098043 Hz
AQ            5.0998774 sec
RG                   6.3
DW                62.400 usec
DE                  6.00 usec
TE                 298.0 K
D1            0.10000000 sec
MCREST        0.00000000 sec
MCWRK         0.01500000 sec

======== CHANNEL f1 ========
NUC1                  1H
P1                  7.50 usec
PL1                 1.60 dB
SFO1         500.2235015 MHz

F2 - Processing parameters
SI                 65536
SF           500.2200371 MHz
WDW                   no
SSB                    0
LB                  0.00 Hz
GB                     0
PC                  1.00

1D NMR plot parameters
CX                 22.80 cm
CY                 15.00 cm
F1P                9.000 ppm
F1               4501.98 Hz
F2P               -0.500 ppm
F2               -250.11 Hz
PPMCM            0.41667 ppm/cm
HZCM           208.42502 Hz/cm
```

1H spectrum

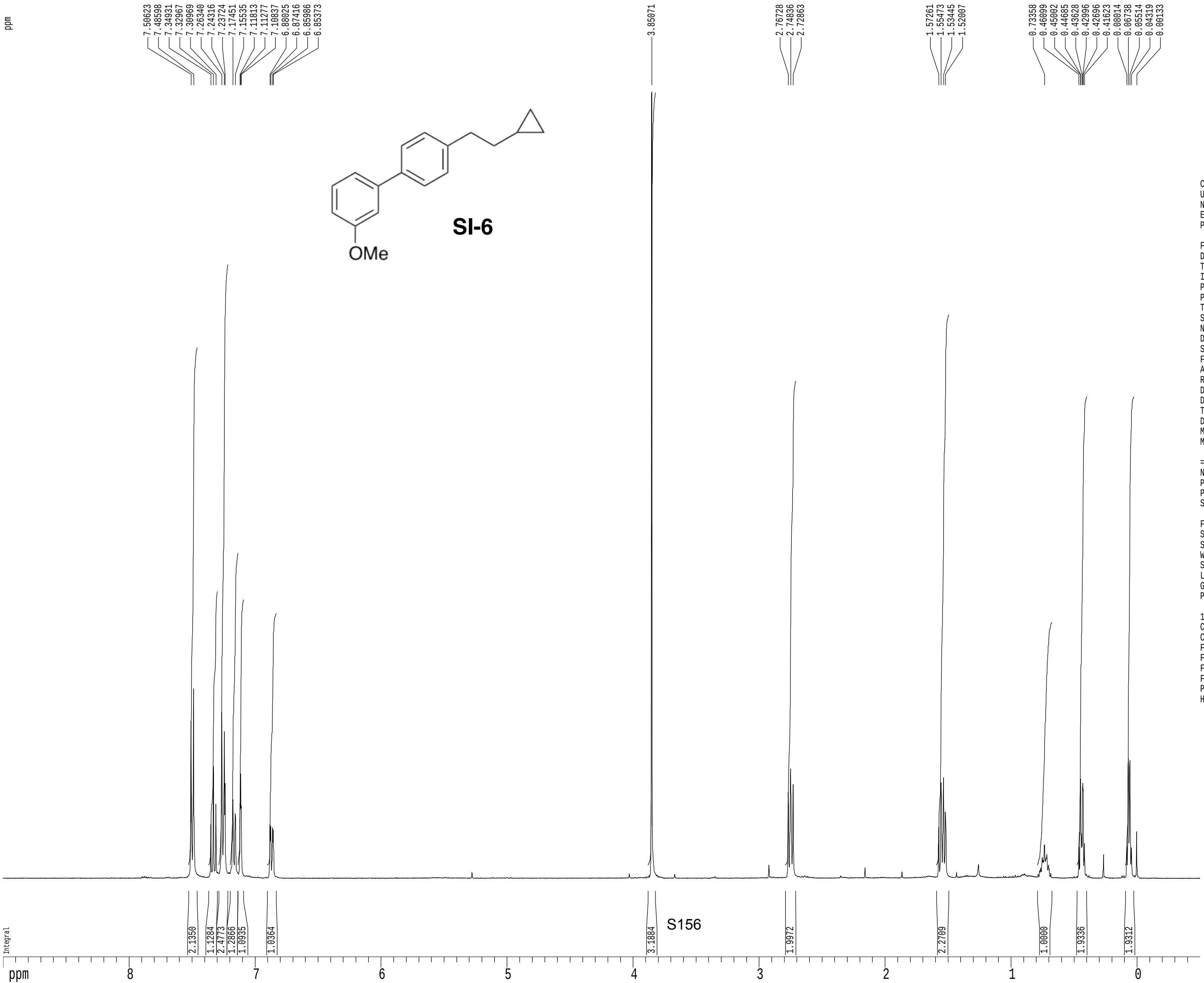

Current Data Parameters
USER sanforda
NAME ABS-2-065-pure
EXPNO 1
PROCNO 1

F2 - Acquisition Parameters
Date_ 20181025
Time 19.24
INSTRUM drx400
PROBHD 5 mm QNP H/F/P
PULPROG zg30
TD 65536
SOLVENT CDCl3
NS 8
DS 2
SWH 6410.256 Hz
FIDRES 0.097813 Hz
AQ 5.1118579 sec
RG 161.3
DW 78.000 usec
DE 4.50 usec
TE 298.0 K
D1 0.10000000 sec
MCREST 0.00000000 sec
MCWRK 0.01500000 sec

======== CHANNEL f1 ========
NUC1 1H
P1 12.00 usec
PL1 -1.10 dB
SFO1 400.1328009 MHz

F2 - Processing parameters
SI 65536
SF 400.1300304 MHz
WDW no
SSB 0
LB 0.00 Hz
GB 0
PC 2.00

1D NMR plot parameters
CX 22.80 cm
CY 15.00 cm
F1P 9.000 ppm
F1 3601.17 Hz
F2P -0.500 ppm
F2 -200.06 Hz
PPMCM 0.41667 ppm/cm
HZCM 166.72086 Hz/cm

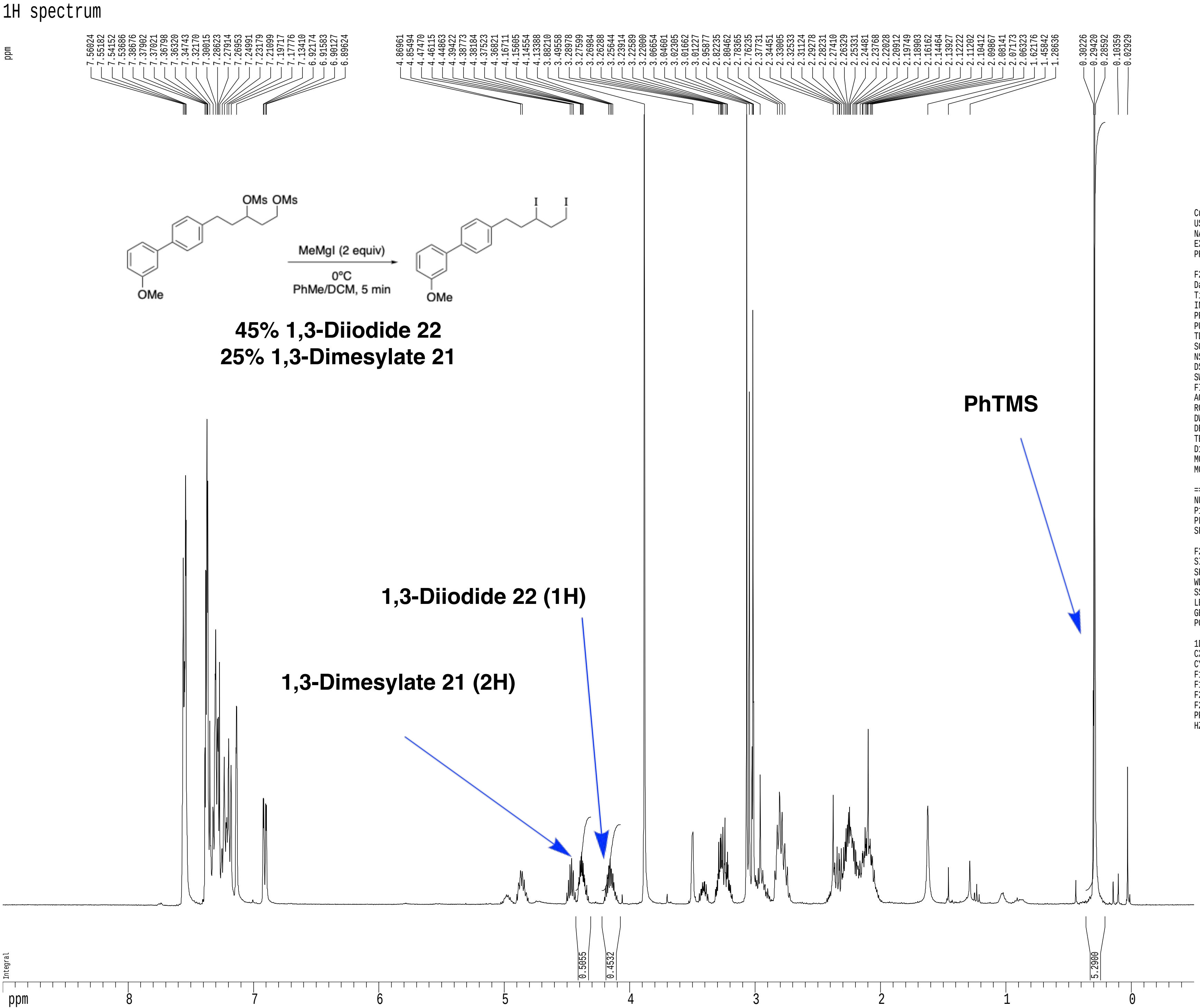


```
Current Data Parameters
USER            mcginnit
NAME          tmm-4-259b
EXPNO                  1
PROCNO                 1

F2 - Acquisition Parameters
Date_           20220802
Time               16.32
INSTRUM           drx400
PROBHD   5 mm QNP H/F/P
PULPROG             zg30
TD                 65536
SOLVENT            CDCl3
NS                     8
DS                     2
SWH             6410.256 Hz
FIDRES          0.097813 Hz
AQ             5.1118579 sec
RG                 101.6
DW                78.000 usec
DE                  4.50 usec
TE                 298.0 K
D1            0.10000000 sec
MCREST        0.00000000 sec
MCWRK         0.01500000 sec

======== CHANNEL f1 ========
NUC1                  1H
P1                 12.00 usec
PL1                -0.90 dB
SFO1         400.1328009 MHz

F2 - Processing parameters
SI                 65536
SF           400.1300175 MHz
WDW                   EM
SSB                    0
LB                  0.30 Hz
GB                     0
PC                  2.00

1D NMR plot parameters
CX                 22.80 cm
CY                100.00 cm
F1P                9.000 ppm
F1               3601.17 Hz
F2P               -0.500 ppm
F2               -200.06 Hz
PPMCM            0.41667 ppm/cm
HZCM           166.72084 Hz/cm
```

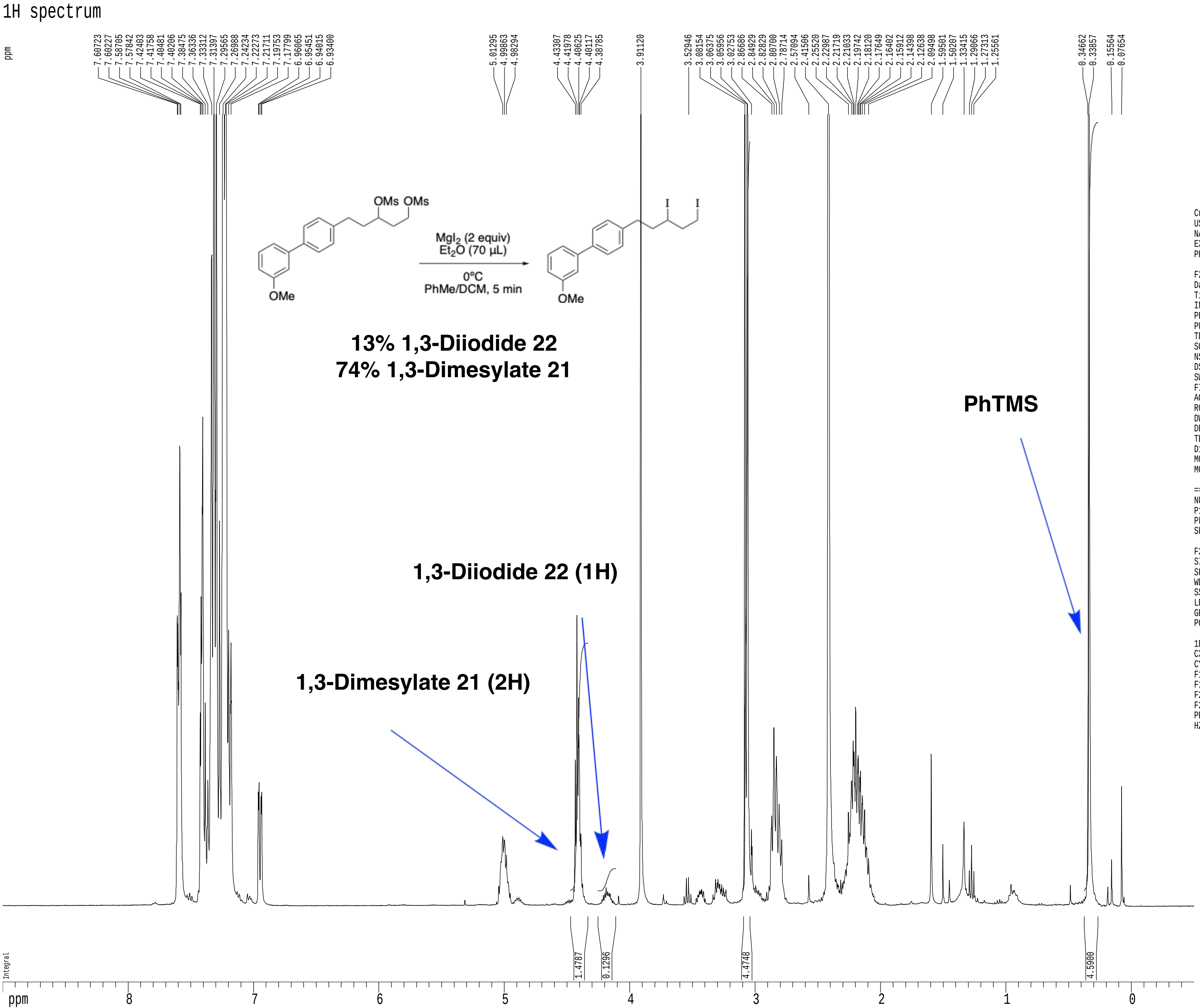

Current Data Parameters
USER mcginnit
NAME tmm-4-259a
EXPNO 1
PROCNO 1

F2 - Acquisition Parameters
Date_ 20220802
Time 16.27
INSTRUM drx400
PROBHD 5 mm QNP H/F/P
PULPROG zg30
TD 65536
SOLVENT CDCl3
NS 8
DS 2
SWH 6410.256 Hz
FIDRES 0.097813 Hz
AQ 5.1118579 sec
RG 64
DW 78.000 usec
DE 4.50 usec
TE 298.0 K
D1 0.10000000 sec
MCREST 0.00000000 sec
MCWRK 0.01500000 sec

======== CHANNEL f1 ========
NUC1 1H
P1 12.00 usec
PL1 -0.90 dB
SFO1 400.1328009 MHz

F2 - Processing parameters
SI 65536
SF 400.1300175 MHz
WDW EM
SSB 0
LB 0.30 Hz
GB 0
PC 2.00

1D NMR plot parameters
CX 22.80 cm
CY 100.00 cm
F1P 9.000 ppm
F1 3601.17 Hz
F2P -0.500 ppm
F2 -200.06 Hz
PPMCM 0.41667 ppm/cm
HZCM 166.72084 Hz/cm

1H spectrum

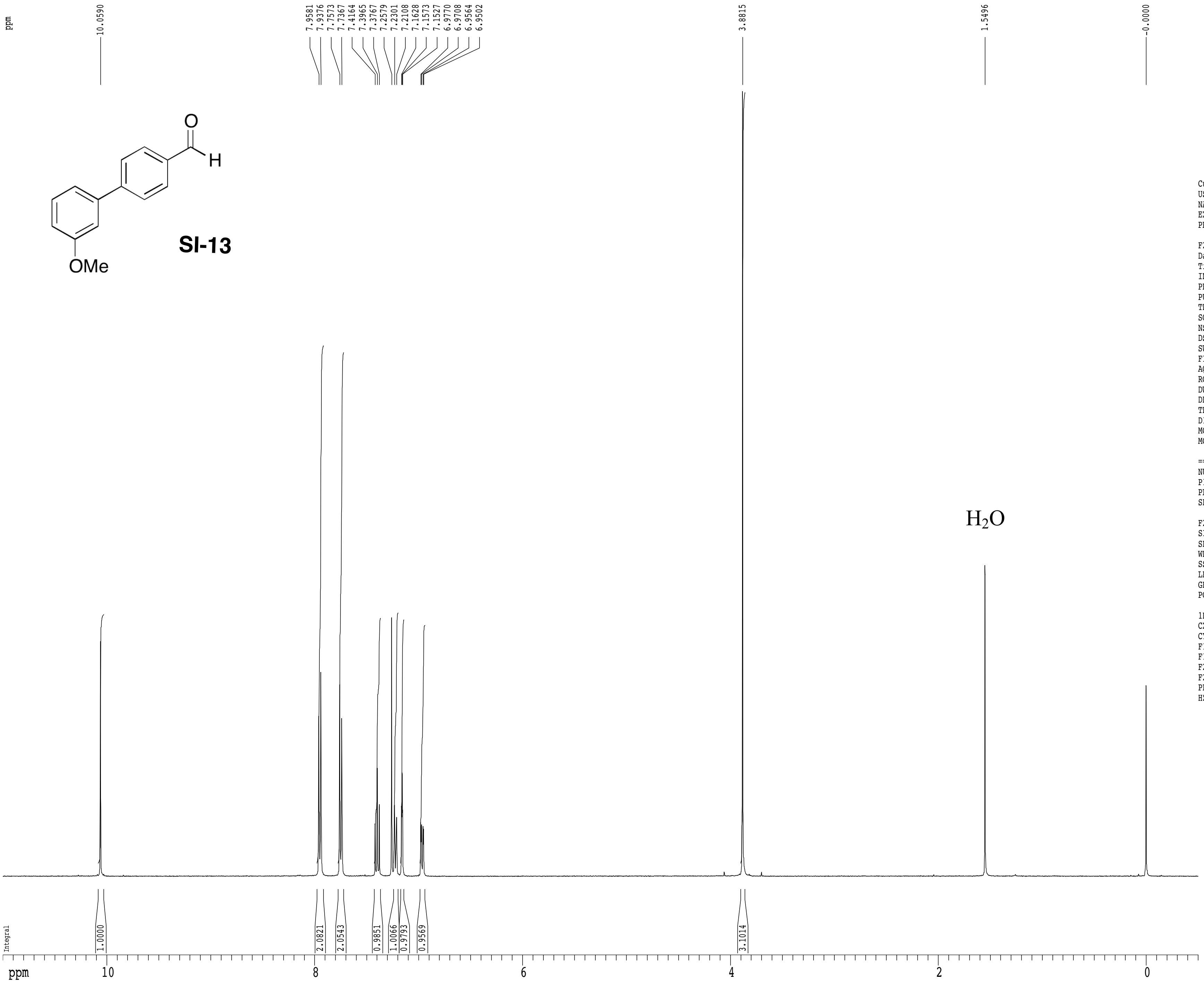

SI-13

```
Current Data Parameters
USER              linpc2
NAME           pcl-1-131
EXPNO                  1
PROCNO                 1

F2 - Acquisition Parameters
Date_           20200924
Time               18.15
INSTRUM           drx400
PROBHD   5 mm QNP H/F/P
PULPROG             zg30
TD                 65536
SOLVENT            CDCl3
NS                     8
DS                     2
SWH             6410.256 Hz
FIDRES          0.097813 Hz
AQ             5.1118579 sec
RG                 812.7
DW                78.000 usec
DE                  4.50 usec
TE                 298.0 K
D1            0.10000000 sec
MCREST        0.00000000 sec
MCWRK         0.01500000 sec

======== CHANNEL f1 ========
NUC1                  1H
P1                 12.00 usec
PL1                -1.10 dB
SFO1         400.1328009 MHz

F2 - Processing parameters
SI                 65536
SF           400.1300223 MHz
WDW                   EM
SSB                    0
LB                  0.30 Hz
GB                     0
PC                  2.00

1D NMR plot parameters
CX                 22.80 cm
CY                 15.00 cm
F1P               11.000 ppm
F1               4401.43 Hz
F2P               -0.500 ppm
F2               -200.07 Hz
PPMCM            0.50439 ppm/cm
HZCM           201.81998 Hz/cm
```

1H spectrum

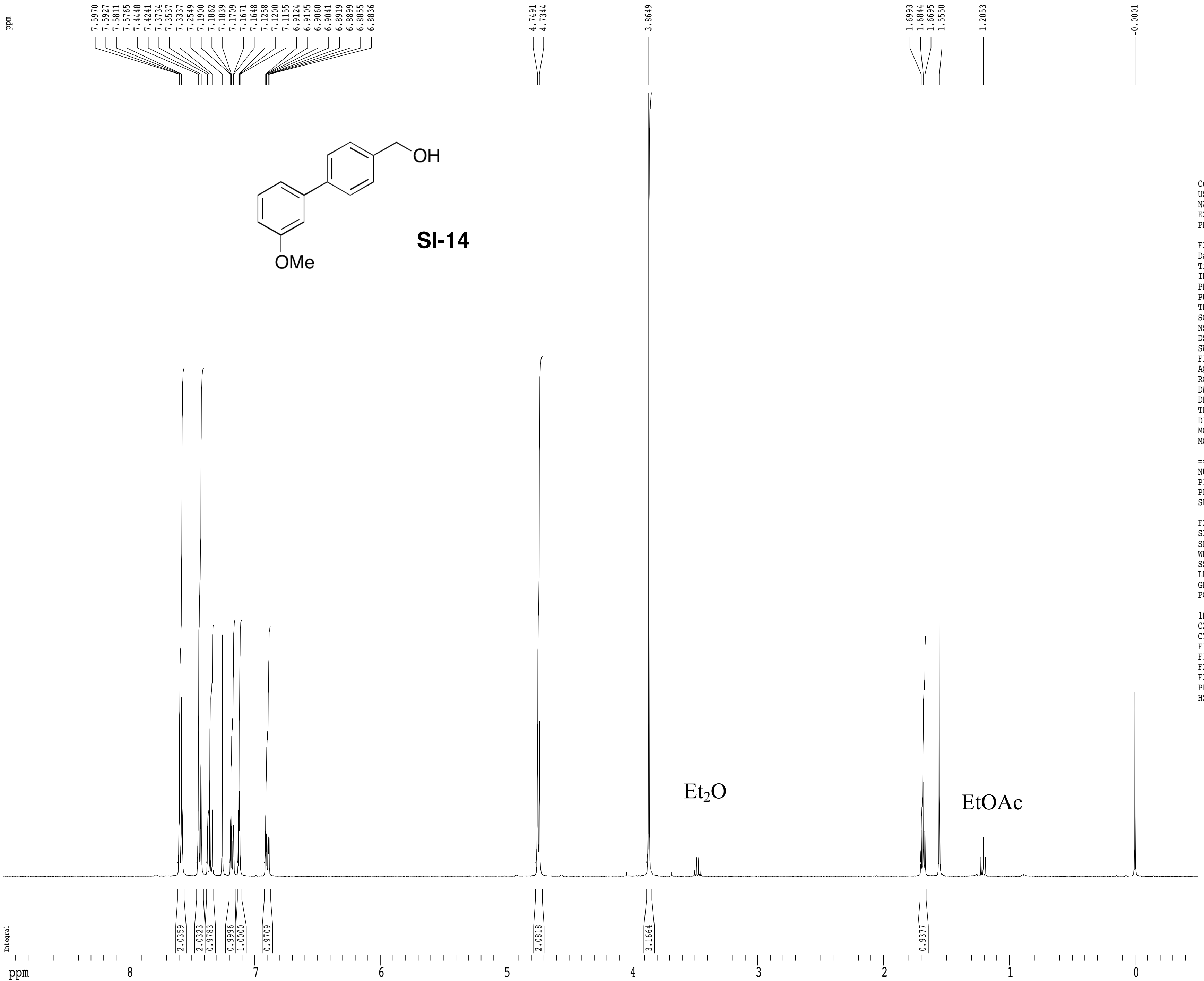

Current Data Parameters
USER linpc2
NAME pcl-1-134
EXPNO 1
PROCNO 1

F2 - Acquisition Parameters
Date_ 20200926
Time 14.15
INSTRUM drx400
PROBHD 5 mm QNP H/F/P
PULPROG zg30
TD 65536
SOLVENT CDCl3
NS 8
DS 2
SWH 6410.256 Hz
FIDRES 0.097813 Hz
AQ 5.1118579 sec
RG 724.1
DW 78.000 usec
DE 4.50 usec
TE 298.0 K
D1 0.10000000 sec
MCREST 0.00000000 sec
MCWRK 0.01500000 sec

======== CHANNEL f1 ========
NUC1 1H
P1 12.00 usec
PL1 -1.10 dB
SFO1 400.1328009 MHz

F2 - Processing parameters
SI 65536
SF 400.1300234 MHz
WDW EM
SSB 0
LB 0.30 Hz
GB 0
PC 2.00

1D NMR plot parameters
CX 22.80 cm
CY 15.00 cm
F1P 9.000 ppm
F1 3601.17 Hz
F2P -0.500 ppm
F2 -200.06 Hz
PPMCM 0.41667 ppm/cm
HZCM 166.72086 Hz/cm

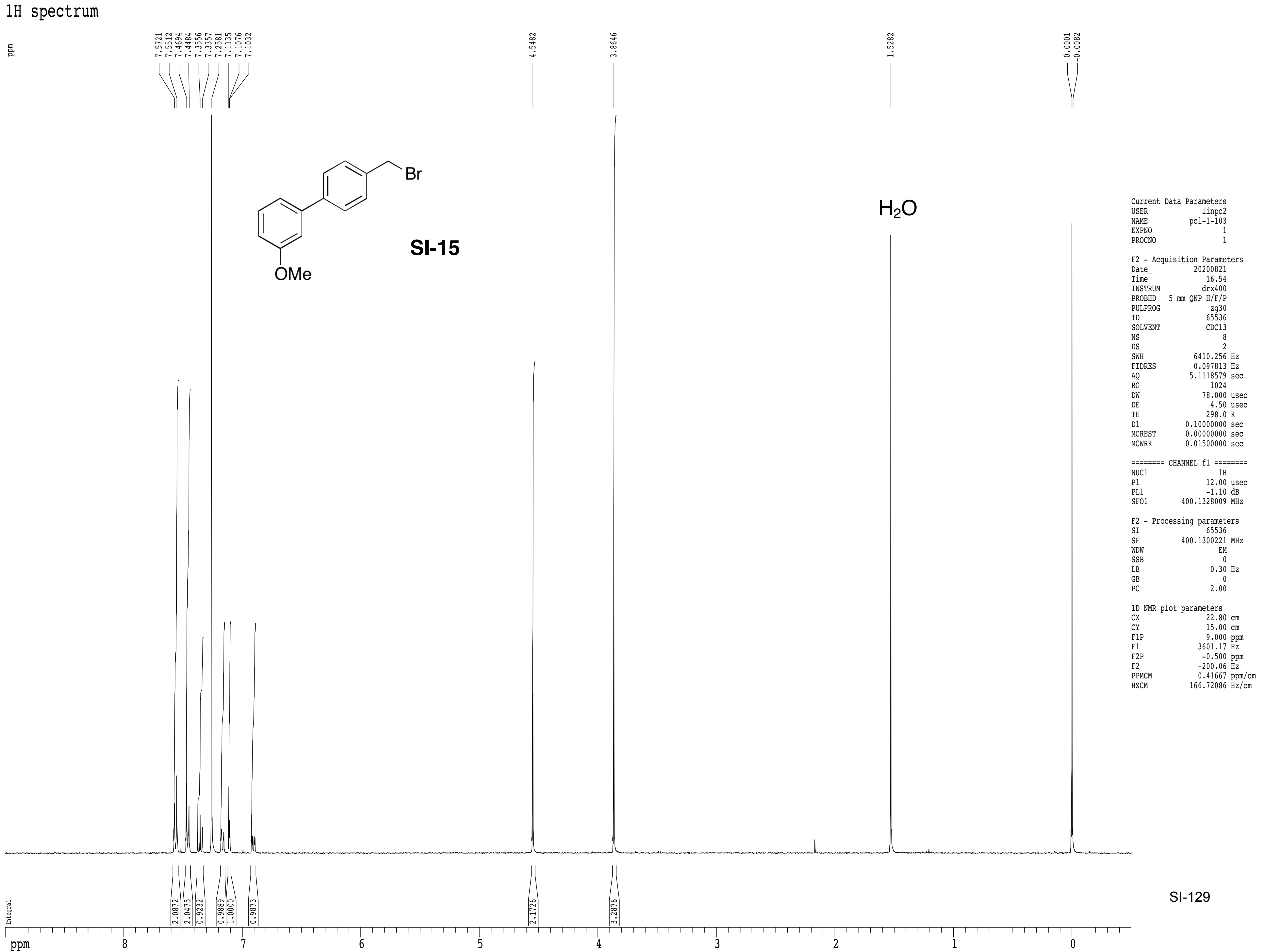
1H spectrum
ppm
7.5721
7.5512
7.4694
7.4484
7.3556
7.3357
7.2581
7.1135
7.1076
7.1032
4.5482
3.8646
1.5282
0.0001
-0.0082
Br
OMe
SI-15
H2O
Current Data Parameters
USER linpc2
NAME pcl-1-103
EXPNO 1
PROCNO 1
F2 - Acquisition Parameters
Date_ 20200821
Time 16.54
INSTRUM drx400
PROBHD 5 mm QNP H/F/P
PULPROG zg30
TD 65536
SOLVENT CDCl3
NS 8
DS 2
SWH 6410.256 Hz
FIDRES 0.097813 Hz
AQ 5.1118579 sec
RG 1024
DW 78.000 usec
DE 4.50 usec
TE 298.0 K
D1 0.10000000 sec
MCREST 0.00000000 sec
MCWRK 0.01500000 sec
======== CHANNEL f1 ========
NUC1 1H
P1 12.00 usec
PL1 -1.10 dB
SFO1 400.1328009 MHz
F2 - Processing parameters
SI 65536
SF 400.1300221 MHz
WDW EM
SSB 0
LB 0.30 Hz
GB 0
PC 2.00
1D NMR plot parameters
CX 22.80 cm
CY 15.00 cm
F1P 9.000 ppm
F1 3601.17 Hz
F2P -0.500 ppm
F2 -200.06 Hz
PPMCM 0.41667 ppm/cm
HZCM 166.72086 Hz/cm
Integral
2.0872
2.0475
0.9232
0.9889
1.0000
0.9873
2.1726
3.2876
ppm
8
7
6
5
4
3
2
1
0

13C spectrum with 1H decoupling

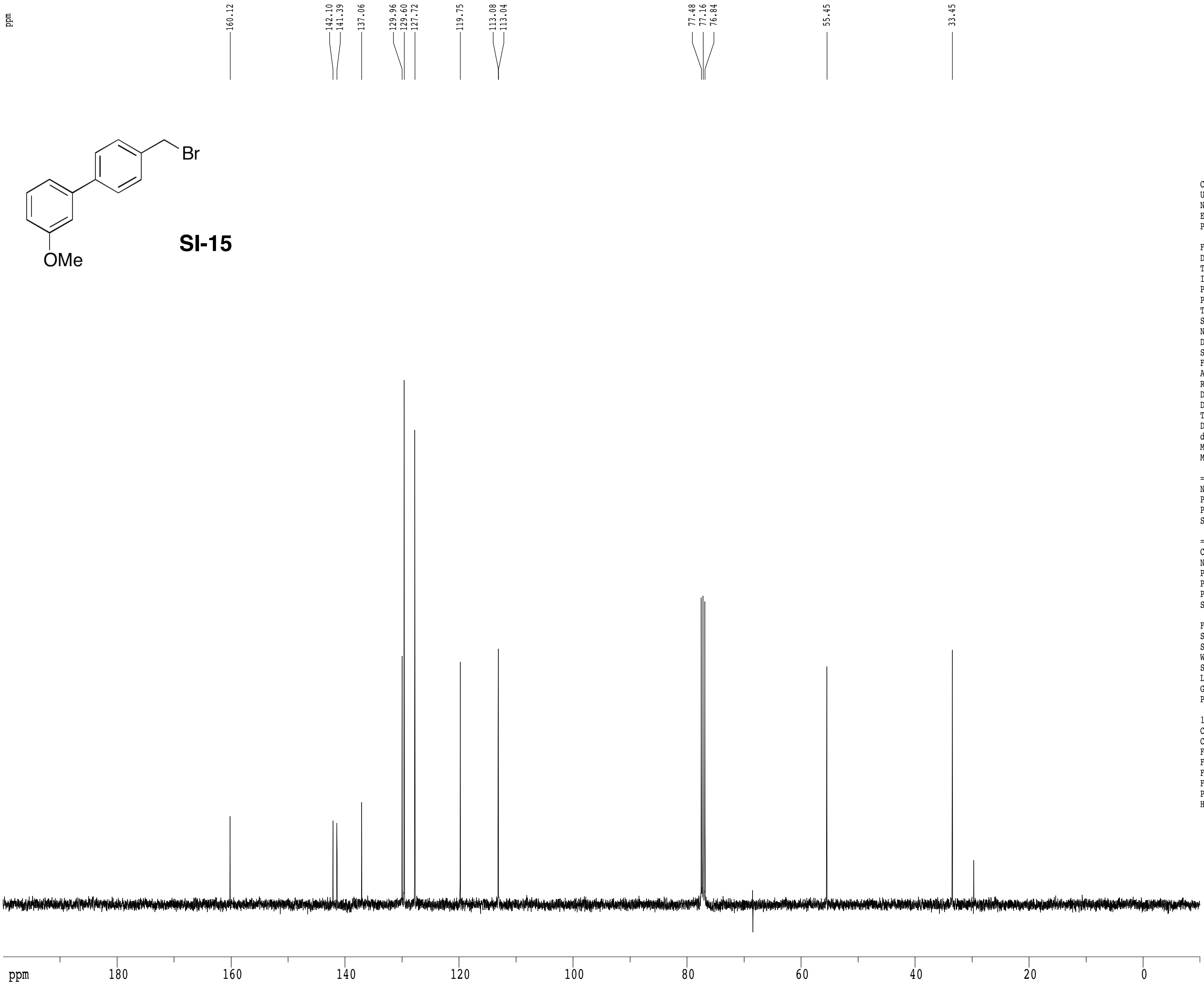

SI-15

Current Data Parameters
USER linpc2
NAME pcl-1-113
EXPNO 6
PROCNO 1

F2 - Acquisition Parameters
Date_ 20201107
Time 11.55
INSTRUM drx400
PROBHD 5 mm QNP H/F/P
PULPROG zgdc30
TD 65536
SOLVENT CDCl3
NS 248
DS 4
SWH 24154.590 Hz
FIDRES 0.368570 Hz
AQ 1.3566452 sec
RG 16384
DW 20.700 usec
DE 20.39 usec
TE 298.0 K
D1 0.10000000 sec
d11 0.03000000 sec
MCREST 0.00000000 sec
MCWRK 0.01500000 sec

======== CHANNEL f1 ========
NUC1 13C
P1 7.82 usec
PL1 -3.00 dB
SFO1 100.6237964 MHz

======== CHANNEL f2 ========
CPDPRG2 waltz16
NUC2 1H
PCPD2 90.00 usec
PL2 -1.10 dB
PL12 16.80 dB
SFO2 400.1328009 MHz

F2 - Processing parameters
SI 65536
SF 100.6127610 MHz
WDW EM
SSB 0
LB 1.00 Hz
GB 0
PC 1.00

1D NMR plot parameters
CX 22.80 cm
CY 10.00 cm
F1P 200.000 ppm
F1 20122.55 Hz
F2P -10.000 ppm
F2 -1006.13 Hz
PPMCM 9.21053 ppm/cm
HZCM 926.69647 Hz/cm

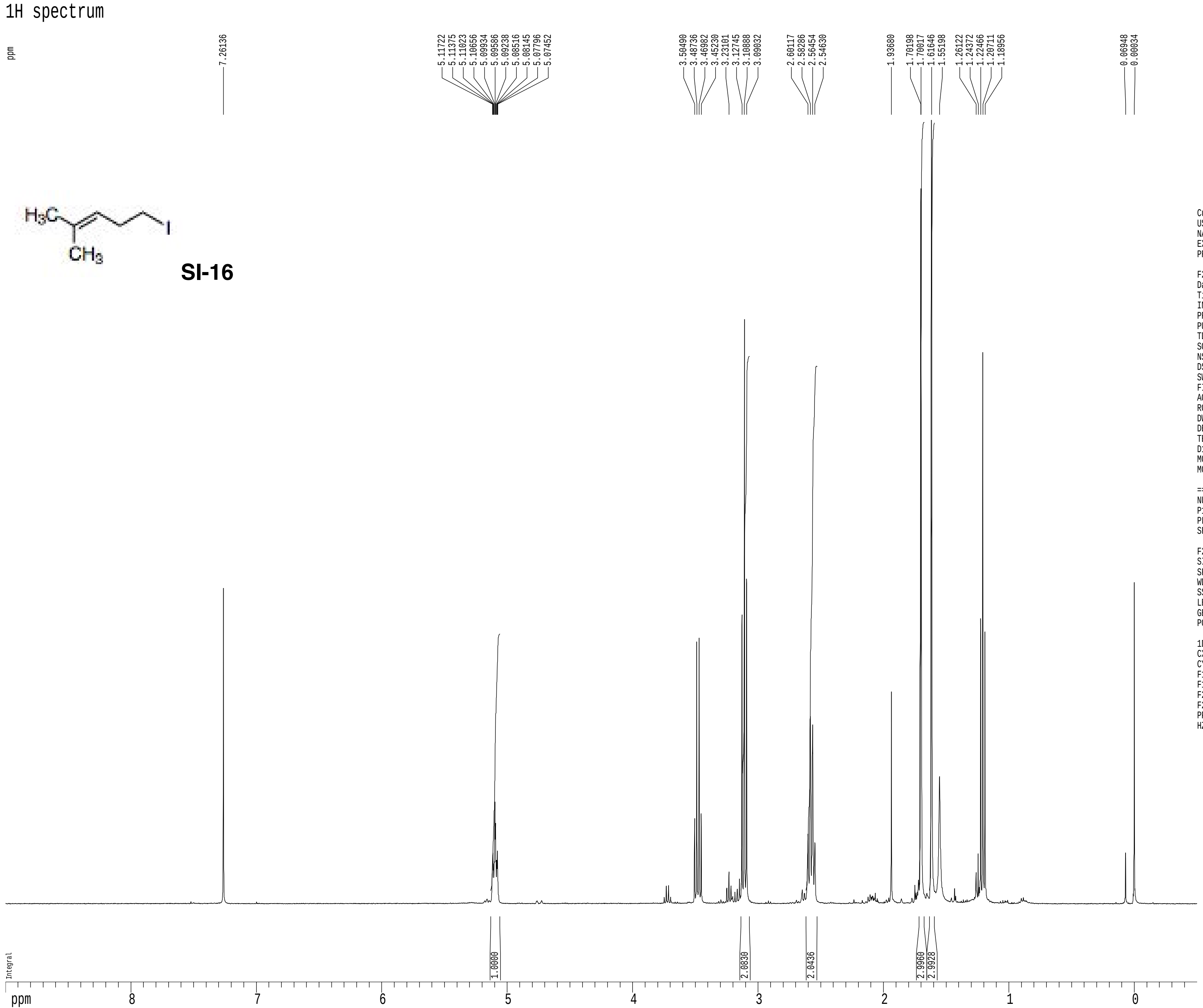

SI-16


```
Current Data Parameters
USER            linpc2
NAME         pcl-1-065
EXPNO                1
PROCNO               1

F2 - Acquisition Parameters
Date_         20200724
Time             17.23
INSTRUM         drx400
PROBHD   5 mm QNP H/F/P
PULPROG           zg30
TD               65536
SOLVENT          CDCl3
NS                   8
DS                   2
SWH           6410.256 Hz
FIDRES        0.097813 Hz
AQ           5.1118579 sec
RG               406.4
DW              78.000 usec
DE                4.50 usec
TE               298.1 K
D1          0.10000000 sec
MCREST      0.00000000 sec
MCWRK       0.01500000 sec

======== CHANNEL f1 ========
NUC1                1H
P1               12.00 usec
PL1              -1.10 dB
SFO1       400.1328009 MHz

F2 - Processing parameters
SI               65536
SF         400.1300210 MHz
WDW                 EM
SSB                  0
LB                0.30 Hz
GB                   0
PC                2.00

1D NMR plot parameters
CX               22.80 cm
CY               15.00 cm
F1P              9.000 ppm
F1             3601.17 Hz
F2P             -0.500 ppm
F2             -200.06 Hz
PPMCM          0.41667 ppm/cm
HZCM         166.72086 Hz/cm
```

1H spectrum

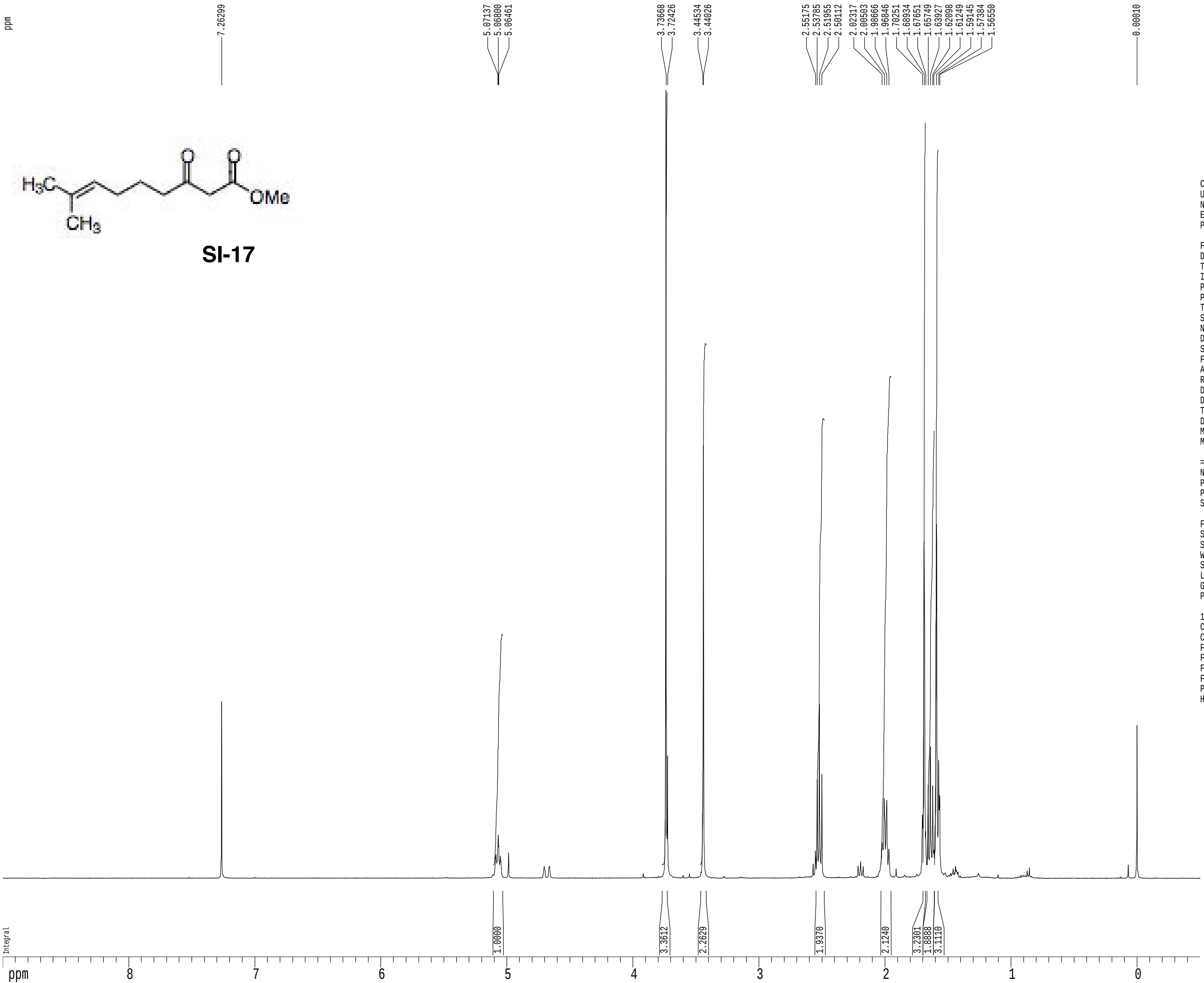

SI-17

Current Data Parameters
USER linpc2
NAME pcl-1-068
EXPNO 3
PROCNO 1

F2 - Acquisition Parameters
Date_ 20200808
Time 11.58
INSTRUM drx400
PROBHD 5 mm QNP H/F/P
PULPROG zg30
TD 65536
SOLVENT CDCl3
NS 8
DS 2
SWH 6410.256 Hz
FIDRES 0.097813 Hz
AQ 5.1118579 sec
RG 322.5
DW 78.000 usec
DE 4.50 usec
TE 297.9 K
D1 0.10000000 sec
MCREST 0.00000000 sec
MCWRK 0.01500000 sec

======== CHANNEL f1 ========
NUC1 1H
P1 12.00 usec
PL1 -1.10 dB
SFO1 400.1328009 MHz

F2 - Processing parameters
SI 65536
SF 400.1300201 MHz
WDW EM
SSB 0
LB 0.30 Hz
GB 0
PC 2.00

1D NMR plot parameters
CX 22.80 cm
CY 15.00 cm
F1P 9.000 ppm
F1 3601.17 Hz
F2P -0.500 ppm
F2 -200.06 Hz
PPMCM 0.41667 ppm/cm
HZCM 166.72084 Hz/cm

1H spectrum

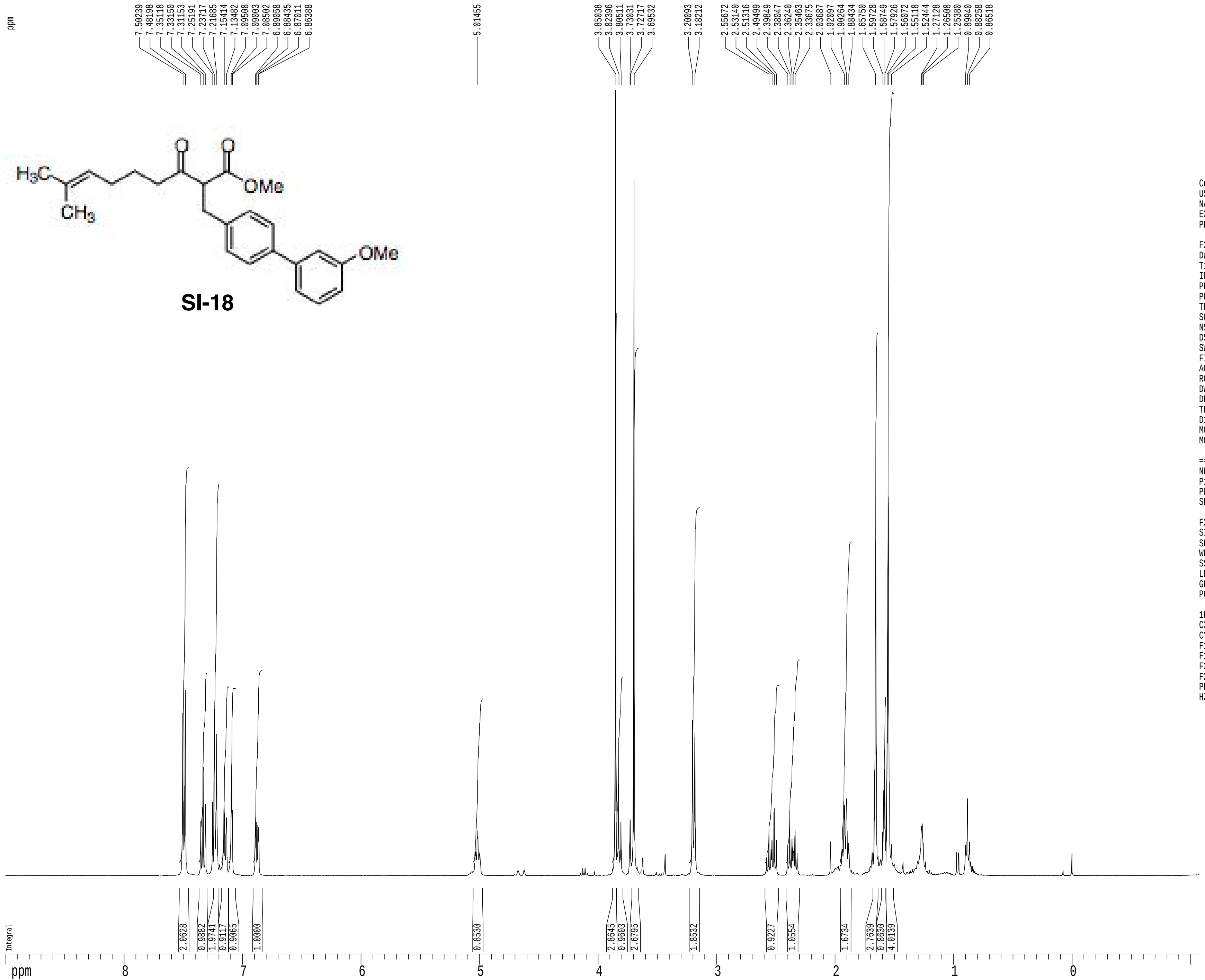

Current Data Parameters
USER linpc2
NAME pcl-1-088
EXPNO 2
PROCNO 1

F2 - Acquisition Parameters
Date_ 20200814
Time 12.11
INSTRUM drx400
PROBHD 5 mm QNP H/F/P
PULPROG zg30
TD 65536
SOLVENT CDCl3
NS 8
DS 2
SWH 6410.256 Hz
FIDRES 0.097813 Hz
AQ 5.1118579 sec
RG 128
DW 78.000 usec
DE 4.50 usec
TE 298.0 K
D1 0.10000000 sec
MCREST 0.00000000 sec
MCWRK 0.01500000 sec

======== CHANNEL f1 ========
NUC1 1H
P1 12.00 usec
PL1 -1.10 dB
SFO1 400.1328009 MHz

F2 - Processing parameters
SI 65536
SF 400.1300243 MHz
WDW EM
SSB 0
LB 0.30 Hz
GB 0
PC 2.00

1D NMR plot parameters
CX 22.80 cm
CY 15.00 cm
F1P 9.000 ppm
F1 3601.17 Hz
F2P -1.071 ppm
F2 -428.52 Hz
PPMCM 0.44171 ppm/cm
HZCM 176.74071 Hz/cm

13C spectrum with 1H decoupling

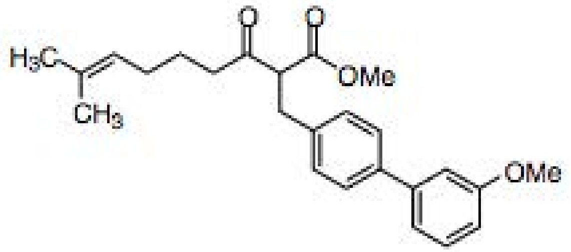

SI-18

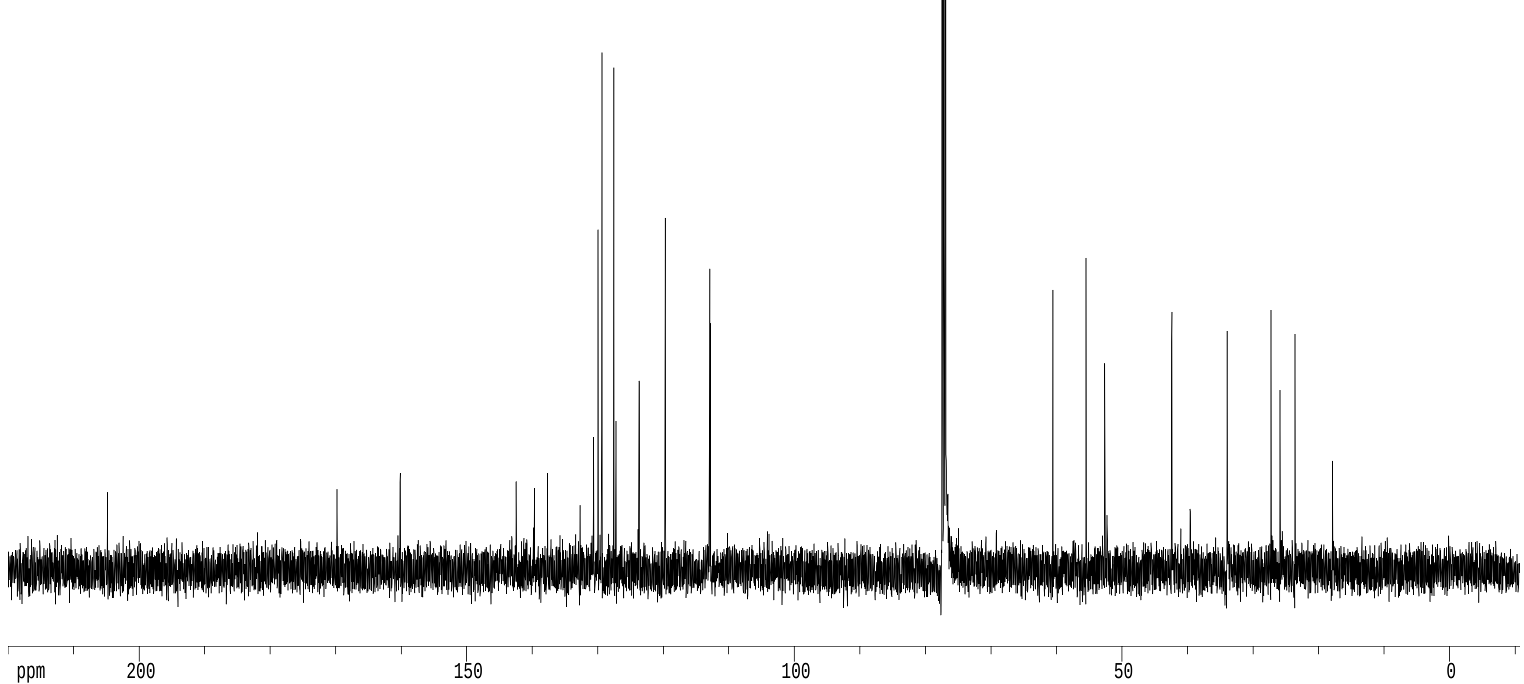

Current Data Parameters
USER linpc2
NAME pcl-1-137
EXPNO 6
PROCNO 1

F2 - Acquisition Parameters
Date_ 20201114
Time 15.27
INSTRUM gn500
PROBHD 5 mm broadband
PULPROG zgdc30
TD 65536
SOLVENT CDCl3
NS 824
DS 4
SWH 30303.031 Hz
FIDRES 0.462388 Hz
AQ 1.0813940 sec
RG 5792.6
DW 16.500 usec
DE 6.00 usec
TE 298.0 K
D1 0.25000000 sec
d11 0.03000000 sec
MCREST 0.00000000 sec
MCWRK 0.01500000 sec

======== CHANNEL f1 ========
NUC1 13C
P1 14.20 usec
PL1 -6.00 dB
SFO1 125.4245824 MHz

======== CHANNEL f2 ========
CPDPRG2 waltz16
NUC2 1H
PCPD2 80.00 usec
PL2 -6.00 dB
PL12 12.30 dB
SFO2 498.7524937 MHz

F2 - Processing parameters
SI 65536
SF 125.4107743 MHz
WDW EM
SSB 0
LB 1.00 Hz
GB 0
PC 2.00

1D NMR plot parameters
CX 20.00 cm
CY 35.00 cm
F1P 220.000 ppm
F1 27590.37 Hz
F2P -10.712 ppm
F2 -1343.39 Hz
PPMCM 11.53559 ppm/cm
HZCM 1446.68787 Hz/cm

1H spectrum

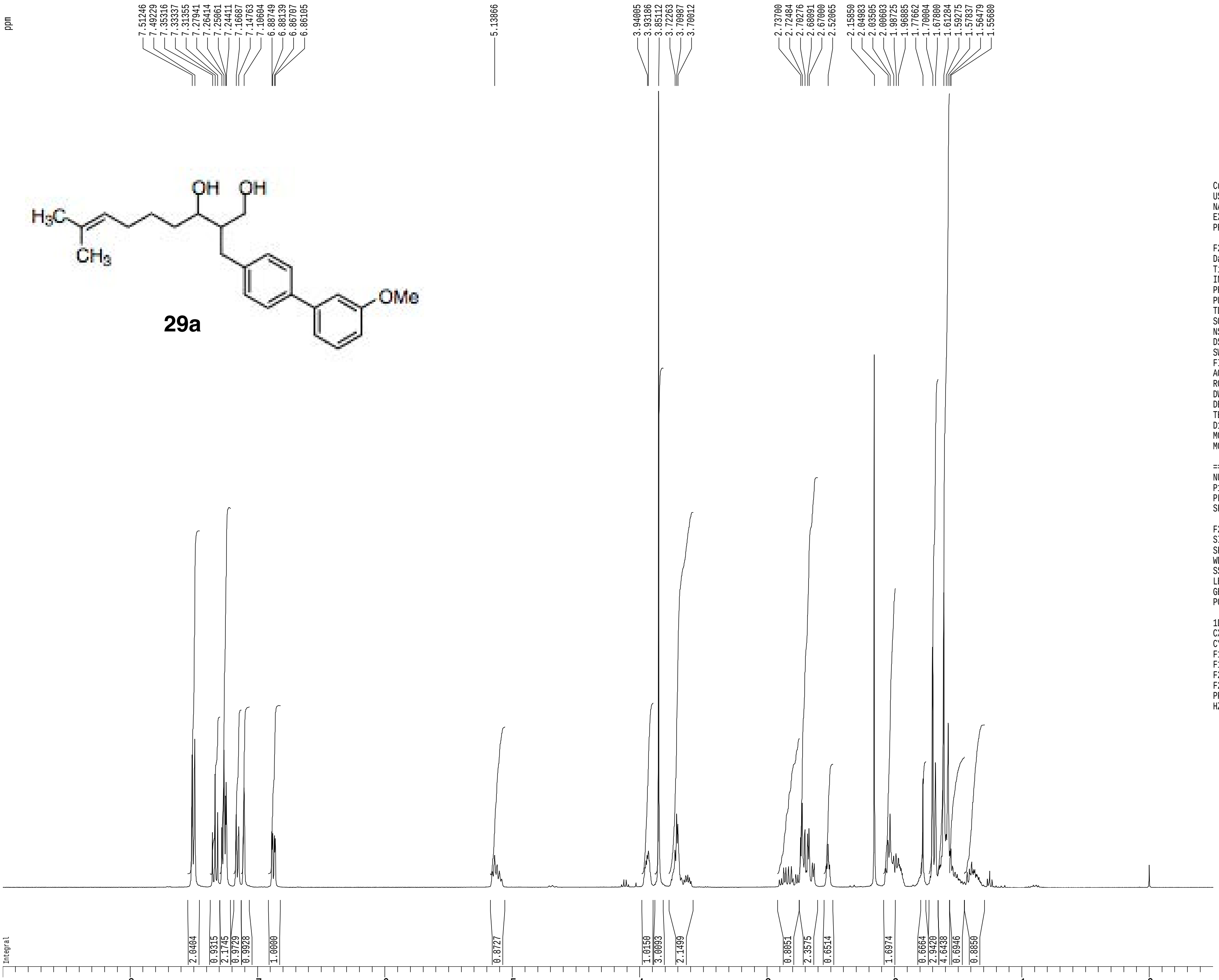


```
Current Data Parameters
USER              linpc2
NAME           pcl-1-138
EXPNO                  1
PROCNO                 1

F2 - Acquisition Parameters
Date_           20200928
Time               17.43
INSTRUM           drx400
PROBHD   5 mm QNP H/F/P
PULPROG             zg30
TD                 65536
SOLVENT            CDCl3
NS                     8
DS                     2
SWH             6410.256 Hz
FIDRES          0.097813 Hz
AQ             5.1118579 sec
RG                   114
DW                78.000 usec
DE                  4.50 usec
TE                 298.0 K
D1            0.10000000 sec
MCREST        0.00000000 sec
MCWRK         0.01500000 sec

======== CHANNEL f1 ========
NUC1                  1H
P1                 12.00 usec
PL1                -1.10 dB
SFO1         400.1328009 MHz

F2 - Processing parameters
SI                 65536
SF           400.1300250 MHz
WDW                   EM
SSB                    0
LB                  0.30 Hz
GB                     0
PC                  2.00

1D NMR plot parameters
CX                 22.80 cm
CY                 15.00 cm
F1P                9.000 ppm
F1               3601.17 Hz
F2P               -0.500 ppm
F2               -200.06 Hz
PPMCM            0.41667 ppm/cm
HZCM           166.72086 Hz/cm
```

13C spectrum with 1H decoupling

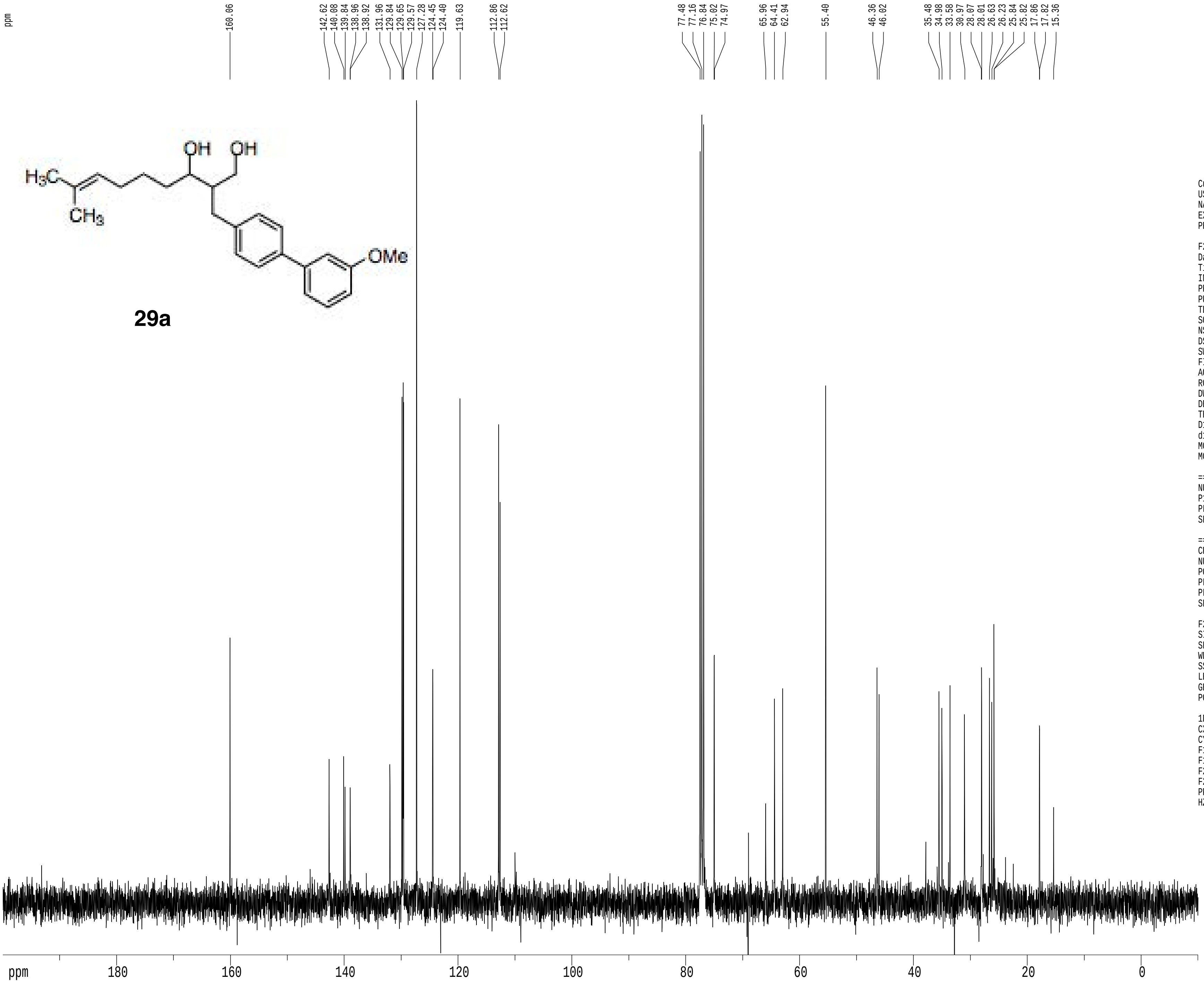

Current Data Parameters
USER linpc2
NAME pcl-1-117
EXPNO 5
PROCNO 1

F2 - Acquisition Parameters
Date_ 20200905
Time 11.49
INSTRUM drx400
PROBHD 5 mm QNP H/F/P
PULPROG zgdc30
TD 65536
SOLVENT CDCl3
NS 176
DS 4
SWH 24154.590 Hz
FIDRES 0.368570 Hz
AQ 1.3566452 sec
RG 14596.5
DW 20.700 usec
DE 20.39 usec
TE 298.0 K
D1 0.10000000 sec
d11 0.03000000 sec
MCREST 0.00000000 sec
MCWRK 0.01500000 sec

======== CHANNEL f1 ========
NUC1 13C
P1 7.82 usec
PL1 -3.00 dB
SFO1 100.6237964 MHz

======== CHANNEL f2 ========
CPDPRG2 waltz16
NUC2 1H
PCPD2 90.00 usec
PL2 -1.10 dB
PL12 16.80 dB
SFO2 400.1328009 MHz

F2 - Processing parameters
SI 65536
SF 100.6127621 MHz
WDW EM
SSB 0
LB 1.00 Hz
GB 0
PC 1.00

1D NMR plot parameters
CX 22.80 cm
CY 15.50 cm
F1P 200.000 ppm
F1 20122.55 Hz
F2P -10.000 ppm
F2 -1006.13 Hz
PPMCM 9.21053 ppm/cm
HZCM 926.69647 Hz/cm

1H spectrum

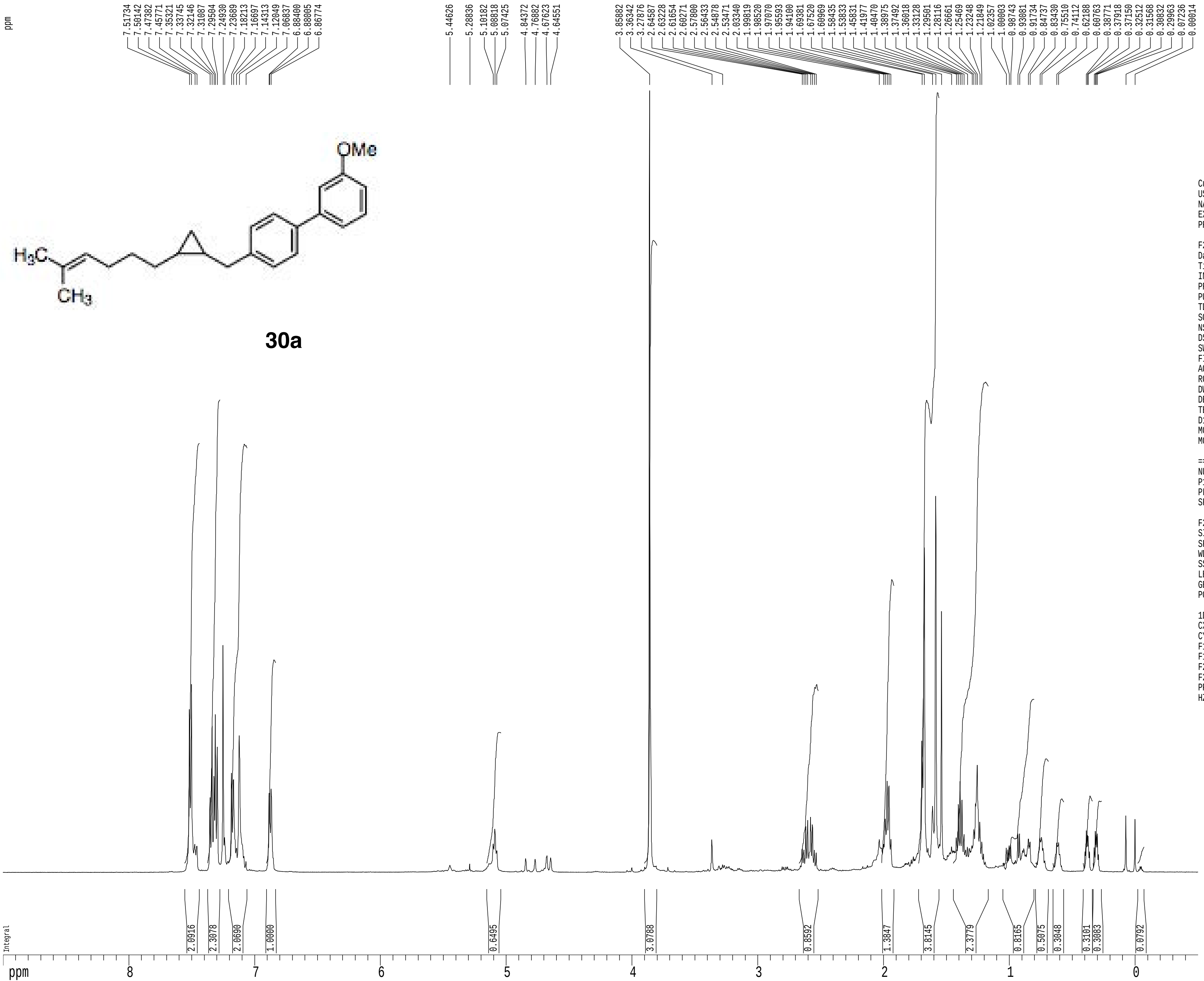


```
Current Data Parameters
USER              linpc2
NAME     pcl-1-118-cosy
EXPNO                  1
PROCNO                 1

F2 - Acquisition Parameters
Date_           20200910
Time               11.41
INSTRUM          cryo500
PROBHD   5 mm CPTCI 1H-
PULPROG             zg30
TD                 81728
SOLVENT            CDCl3
NS                     8
DS                     2
SWH            8012.820 Hz
FIDRES         0.098043 Hz
AQ            5.0998774 sec
RG                   7.1
DW                62.400 usec
DE                  6.00 usec
TE                 298.0 K
D1            0.10000000 sec
MCREST        0.00000000 sec
MCWRK         0.01500000 sec

======== CHANNEL f1 ========
NUC1                  1H
P1                  7.50 usec
PL1                 1.60 dB
SFO1        500.2235015 MHz

F2 - Processing parameters
SI                 65536
SF          500.2200374 MHz
WDW                   EM
SSB                    0
LB                  0.30 Hz
GB                     0
PC                  1.00

1D NMR plot parameters
CX                 22.80 cm
CY                 15.00 cm
F1P                9.000 ppm
F1               4501.98 Hz
F2P               -0.500 ppm
F2               -250.11 Hz
PPMCM            0.41667 ppm/cm
HZCM           208.42502 Hz/cm
```

13C spectrum with 1H decoupling

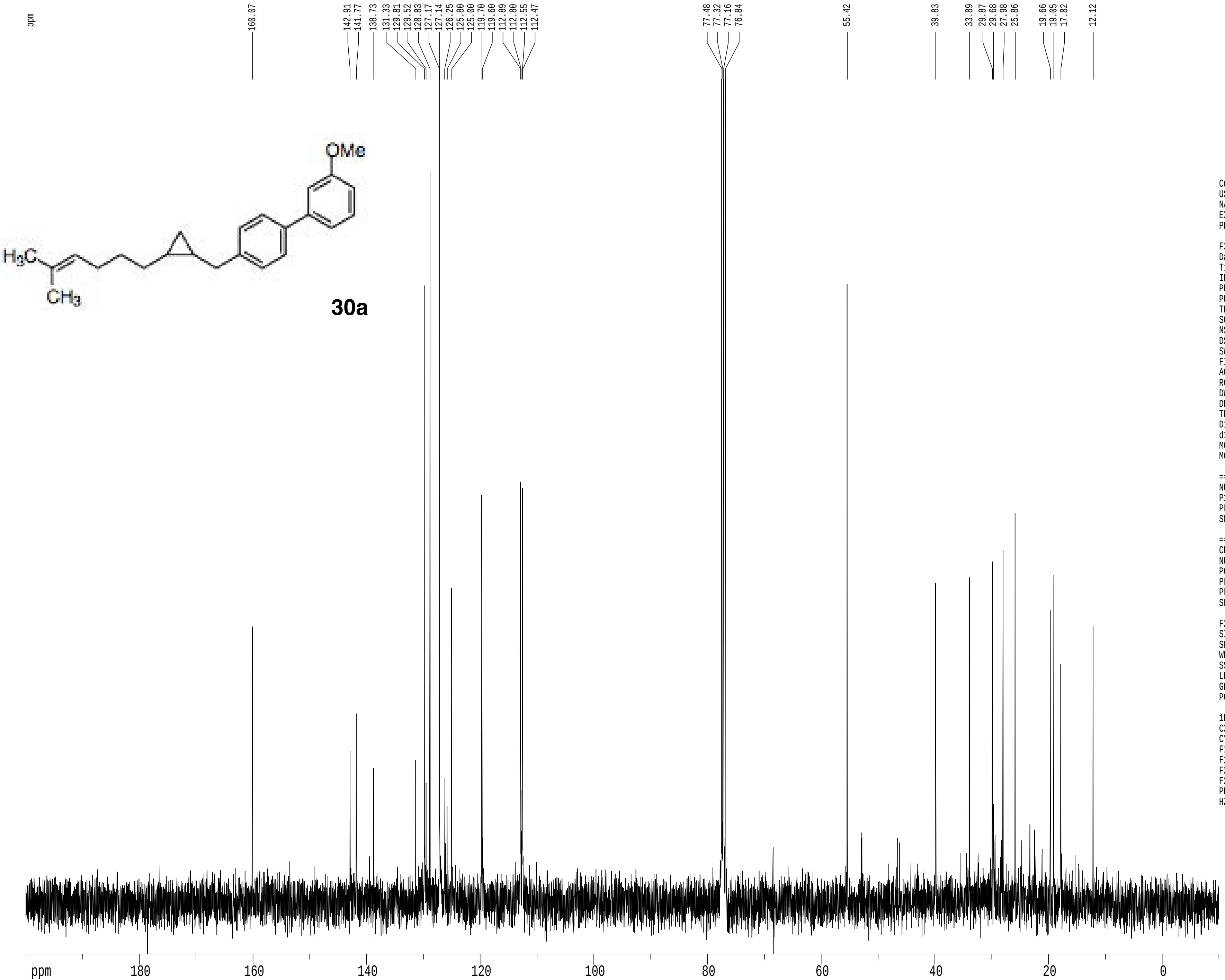

Current Data Parameters
USER linpc2
NAME pcl-1-139
EXPNO 5
PROCNO 1

F2 - Acquisition Parameters
Date_ 20201107
Time 13.04
INSTRUM drx400
PROBHD 5 mm QNP H/F/P
PULPROG zgdc30
TD 65536
SOLVENT CDCl3
NS 288
DS 4
SWH 24154.590 Hz
FIDRES 0.368570 Hz
AQ 1.3566452 sec
RG 9195.2
DW 20.700 usec
DE 20.39 usec
TE 298.0 K
D1 0.10000000 sec
d11 0.03000000 sec
MCREST 0.00000000 sec
MCWRK 0.01500000 sec

======== CHANNEL f1 ========
NUC1 13C
P1 7.82 usec
PL1 -3.00 dB
SFO1 100.6237964 MHz

======== CHANNEL f2 ========
CPDPRG2 waltz16
NUC2 1H
PCPD2 90.00 usec
PL2 -1.10 dB
PL12 16.80 dB
SFO2 400.1328009 MHz

F2 - Processing parameters
SI 65536
SF 100.6127595 MHz
WDW EM
SSB 0
LB 1.00 Hz
GB 0
PC 1.00

1D NMR plot parameters
CX 22.80 cm
CY 30.00 cm
F1P 200.000 ppm
F1 20122.55 Hz
F2P -10.000 ppm
F2 -1006.13 Hz
PPMCM 9.21053 ppm/cm
HZCM 926.69647 Hz/cm

gcosy60

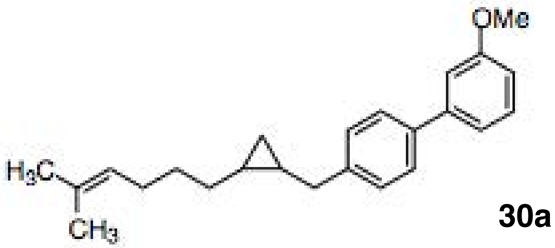

30a

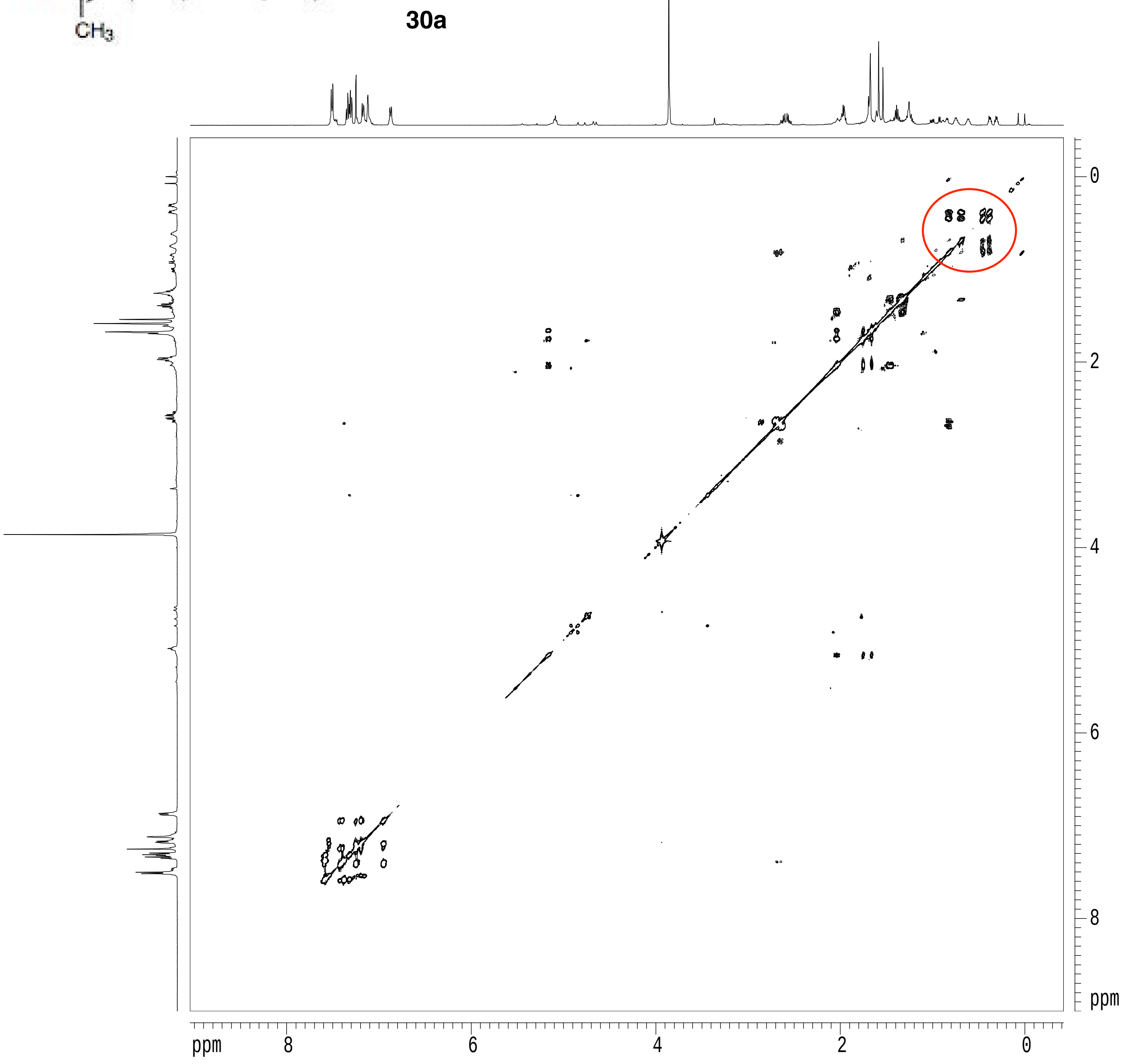

Current Data Parameters
USER linpc2
NAME pcl-1-118-cosy
EXPNO 2
PROCNO 1

F2 - Acquisition Parameters
Date_ 20200910
Time 11.45
INSTRUM cryo500
PROBHD 5 mm CPTCI 1H-
PULPROG cosygp60.prd
TD 2048
SOLVENT CDCl3
NS 1
DS 16
SWH 4734.849 Hz
FIDRES 2.311938 Hz
AQ 0.2163188 sec
RG 161.3
DW 105.600 usec
DE 6.00 usec
TE 298.0 K
d0 0.00000300 sec
D1 1.00000000 sec
d13 0.00000300 sec
D16 0.00020000 sec
IN0 0.00021120 sec

======== CHANNEL f1 ========
NUC1 1H
P1 7.50 usec
PL1 1.60 dB
SFO1 500.2221580 MHz

====== GRADIENT CHANNEL =====
GPNAM1 sine.100
GPNAM2 sine.100
GPX1 0.00 %
GPX2 0.00 %
GPY1 0.00 %
GPY2 0.00 %
GPZ1 17.00 %
GPZ2 17.00 %
P16 1000.00 usec

F1 - Acquisition parameters
ND0 1
TD 512
SFO1 500.2222 MHz
FIDRES 9.247751 Hz
SW 9.465 ppm
FnMODE QF

F2 - Processing parameters
SI 1024
SF 500.2200000 MHz
WDW SINE
SSB 0
LB 0.00 Hz
GB 0
PC 1.00

F1 - Processing parameters
SI 1024
MC2 QF
SF 500.2200000 MHz
WDW SINE
SSB 0
LB 0.00 Hz
GB 0

2D NMR plot parameters
CX2 15.00 cm
CX1 15.00 cm
F2PLO 9.047 ppm
F2LO 4525.43 Hz
F2PHI -0.419 ppm
F2HI -209.42 Hz
F1PLO 9.000 ppm
F1LO 4501.98 Hz
F1PHI -0.419 ppm
F1HI [illegible]
F2PPMCM 0.63104 ppm/cm
F2HZCM 315.65659 Hz/cm
F1PPMCM 0.62791 ppm/cm
F1HZCM 314.09351 Hz/cm

1H spectrum

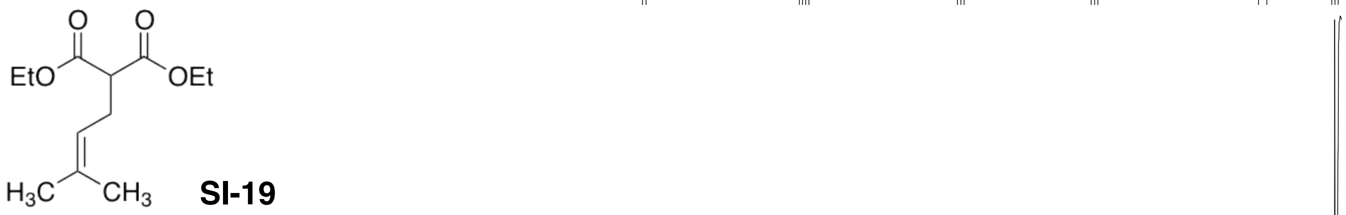

SI-19

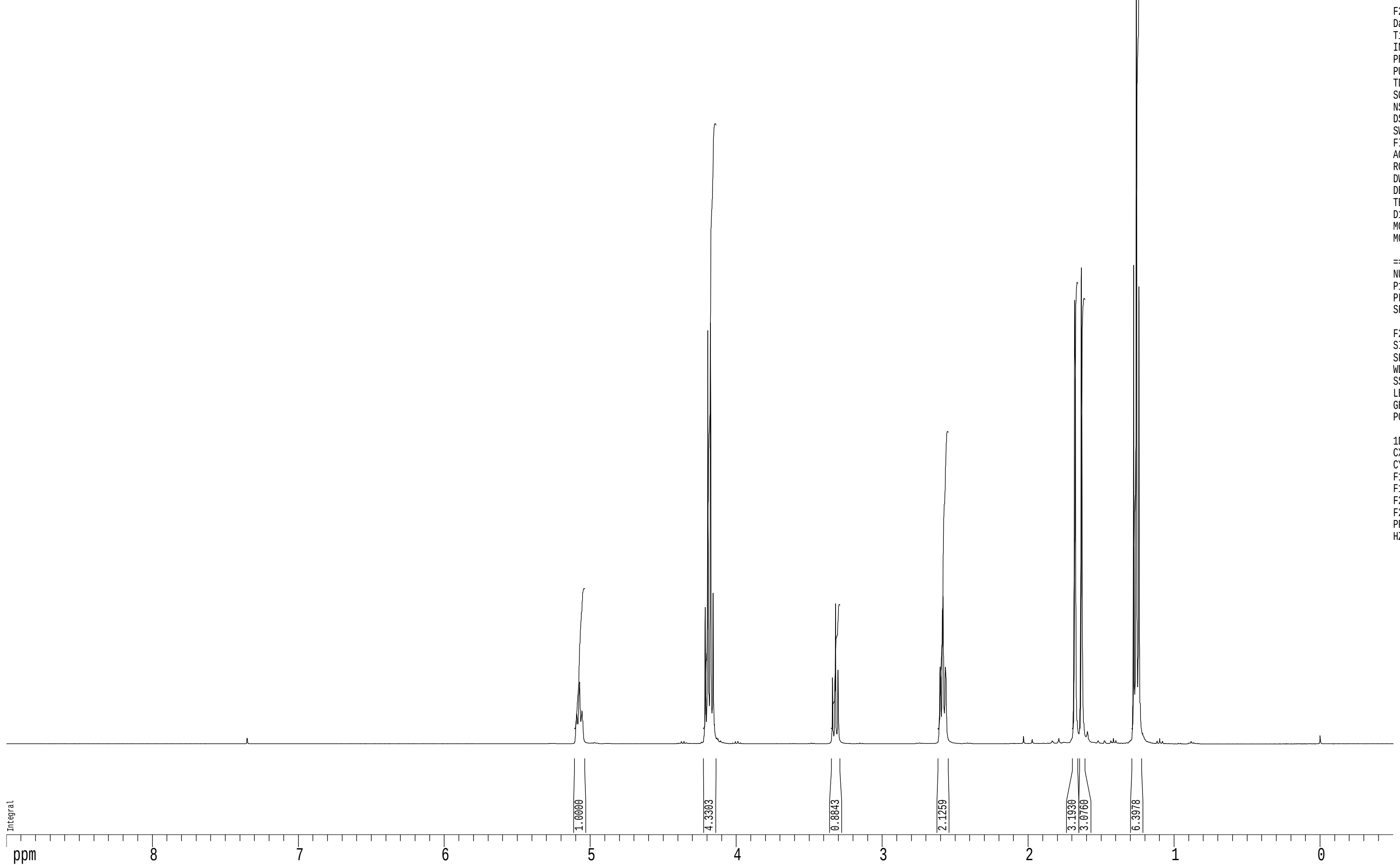

Current Data Parameters
USER linpc2
NAME pcl-1-219
EXPNO 2
PROCNO 1

F2 - Acquisition Parameters
Date_ 20210127
Time 11.33
INSTRUM drx400
PROBHD 5 mm QNP H/F/P
PULPROG zg30
TD 65536
SOLVENT CDCl3
NS 8
DS 2
SWH 6410.256 Hz
FIDRES 0.097813 Hz
AQ 5.1118579 sec
RG 18
DW 78.000 usec
DE 4.50 usec
TE 298.0 K
D1 0.10000000 sec
MCREST 0.00000000 sec
MCWRK 0.01500000 sec

======== CHANNEL f1 ========
NUC1 1H
P1 12.00 usec
PL1 -1.60 dB
SFO1 400.1328009 MHz

F2 - Processing parameters
SI 65536
SF 400.1299847 MHz
WDW EM
SSB 0
LB 0.30 Hz
GB 0
PC 2.00

1D NMR plot parameters
CX 22.80 cm
CY 15.00 cm
F1P 9.000 ppm
F1 3601.17 Hz
F2P -0.500 ppm
F2 -200.06 Hz
PPMCM 0.41667 ppm/cm
HZCM 166.72083 Hz/cm

1H spectrum

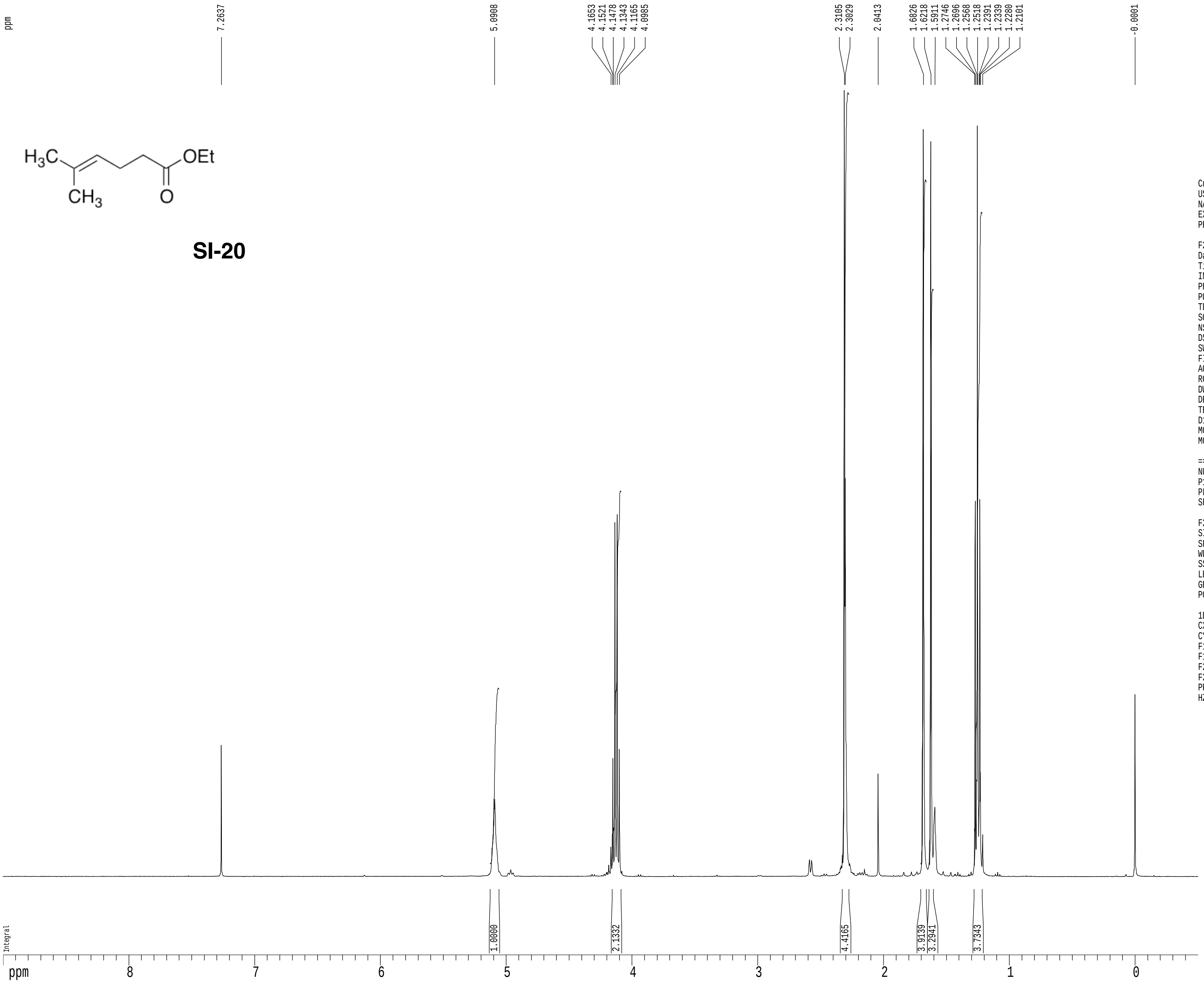

SI-20

Current Data Parameters
USER linpc2
NAME pcl-1-234
EXPNO 1
PROCNO 1

F2 - Acquisition Parameters
Date_ 20210206
Time 14.03
INSTRUM drx400
PROBHD 5 mm QNP H/F/P
PULPROG zg30
TD 65536
SOLVENT CDCl3
NS 8
DS 2
SWH 6410.256 Hz
FIDRES 0.097813 Hz
AQ 5.1118579 sec
RG 181
DW 78.000 usec
DE 4.50 usec
TE 298.0 K
D1 0.10000000 sec
MCREST 0.00000000 sec
MCWRK 0.01500000 sec

======== CHANNEL f1 ========
NUC1 1H
P1 12.00 usec
PL1 -1.60 dB
SFO1 400.1328009 MHz

F2 - Processing parameters
SI 65536
SF 400.1300199 MHz
WDW EM
SSB 0
LB 0.30 Hz
GB 0
PC 2.00

1D NMR plot parameters
CX 22.80 cm
CY 15.00 cm
F1P 9.000 ppm
F1 3601.17 Hz
F2P -0.500 ppm
F2 -200.06 Hz
PPMCM 0.41667 ppm/cm
HZCM 166.72084 Hz/cm

1H spectrum

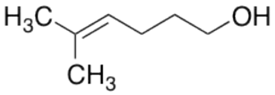

SI-21

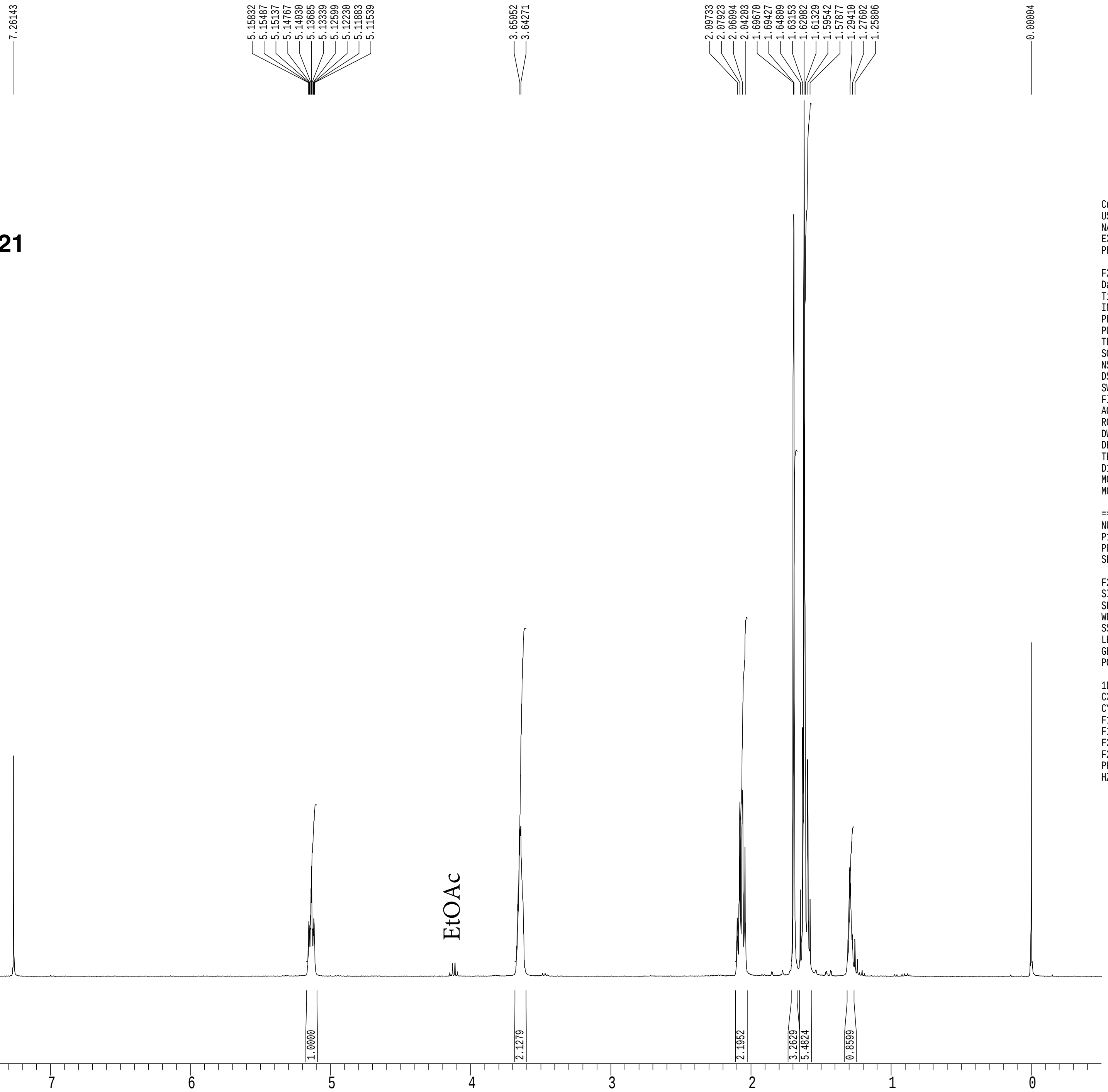


```
Current Data Parameters
USER            linpc2
NAME         pcl-1-235
EXPNO                2
PROCNO               1

F2 - Acquisition Parameters
Date_         20210209
Time             13.40
INSTRUM         drx400
PROBHD   5 mm QNP H/F/P
PULPROG           zg30
TD               65536
SOLVENT          CDCl3
NS                   8
DS                   2
SWH           6410.256 Hz
FIDRES        0.097813 Hz
AQ           5.1118579 sec
RG                 256
DW              78.000 usec
DE                4.50 usec
TE               298.0 K
D1          0.10000000 sec
MCREST      0.00000000 sec
MCWRK       0.01500000 sec

======== CHANNEL f1 ========
NUC1                1H
P1               12.00 usec
PL1              -1.60 dB
SFO1       400.1328009 MHz

F2 - Processing parameters
SI               65536
SF         400.1300208 MHz
WDW                 EM
SSB                  0
LB                0.30 Hz
GB                   0
PC                2.00

1D NMR plot parameters
CX               22.80 cm
CY               15.00 cm
F1P              9.000 ppm
F1             3601.17 Hz
F2P             -0.500 ppm
F2             -200.06 Hz
PPMCM          0.41667 ppm/cm
HZCM         166.72086 Hz/cm
```

1H spectrum

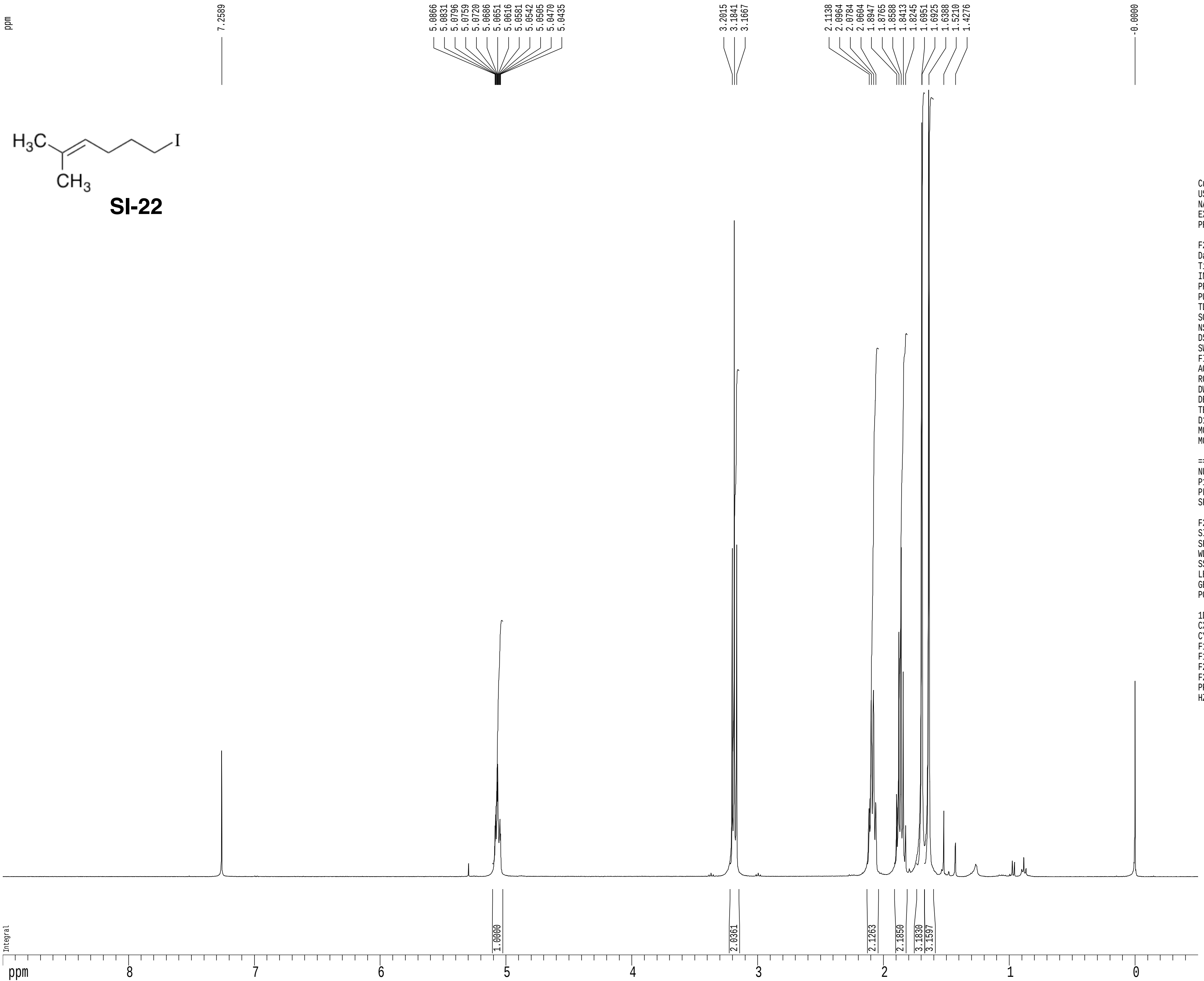

SI-22

Current Data Parameters
USER linpc2
NAME pcl-1-236
EXPNO 1
PROCNO 1

F2 - Acquisition Parameters
Date_ 20210210
Time 10.14
INSTRUM drx400
PROBHD 5 mm QNP H/F/P
PULPROG zg30
TD 65536
SOLVENT CDCl3
NS 8
DS 2
SWH 6410.256 Hz
FIDRES 0.097813 Hz
AQ 5.1118579 sec
RG 203.2
DW 78.000 usec
DE 4.50 usec
TE 298.0 K
D1 0.10000000 sec
MCREST 0.00000000 sec
MCWRK 0.01500000 sec

======== CHANNEL f1 ========
NUC1 1H
P1 12.00 usec
PL1 -1.60 dB
SFO1 400.1328009 MHz

F2 - Processing parameters
SI 65536
SF 400.1300217 MHz
WDW EM
SSB 0
LB 0.30 Hz
GB 0
PC 2.00

1D NMR plot parameters
CX 22.80 cm
CY 15.00 cm
F1P 9.000 ppm
F1 3601.17 Hz
F2P -0.500 ppm
F2 -200.06 Hz
PPMCM 0.41667 ppm/cm
HZCM 166.72086 Hz/cm

1H spectrum

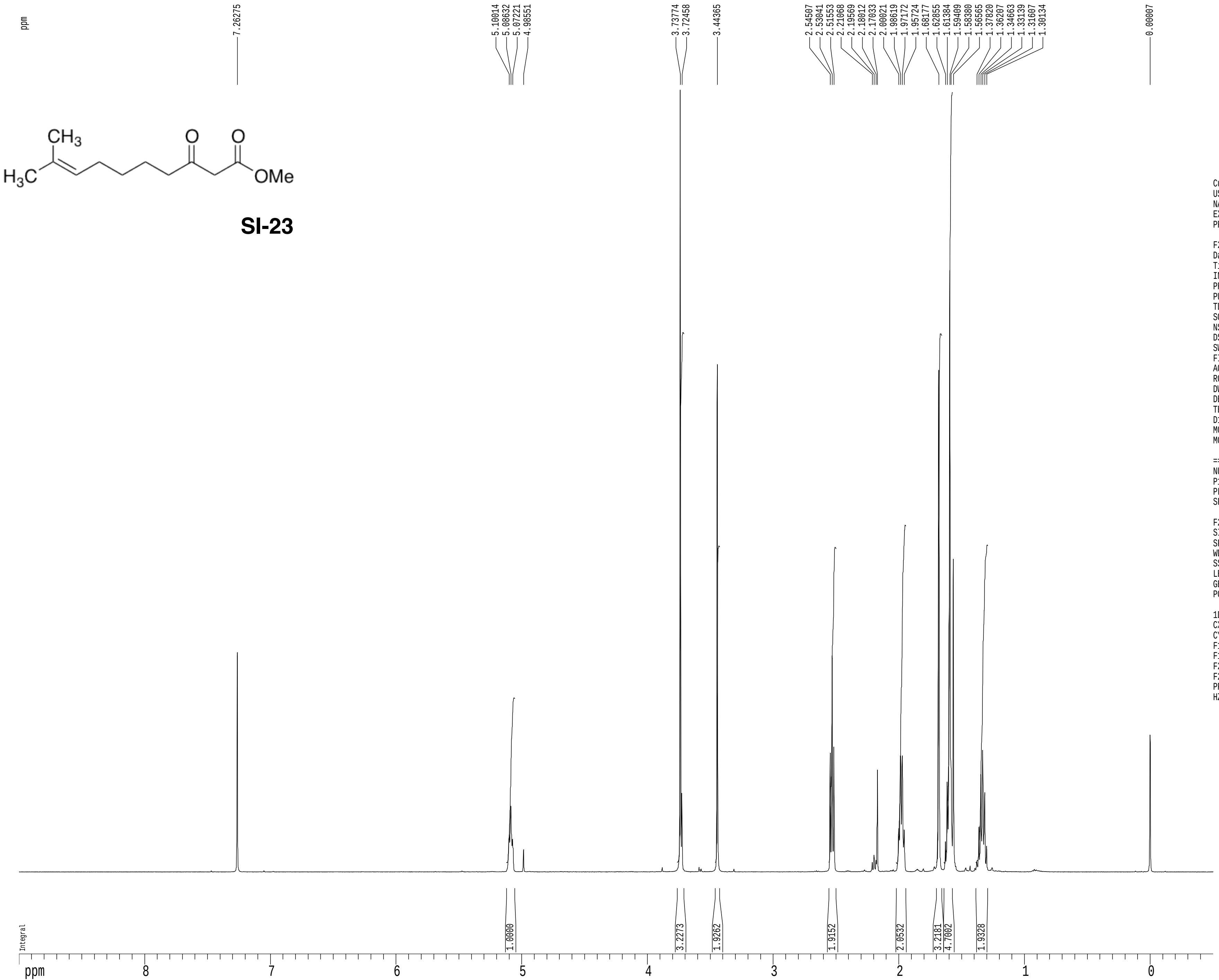

SI-23


```
Current Data Parameters
USER            linpc2
NAME         pcl-1-237
EXPNO                4
PROCNO               1

F2 - Acquisition Parameters
Date_         20210319
Time             15.41
INSTRUM        cryo500
PROBHD   5 mm CPTCI 1H-
PULPROG           zg30
TD               81728
SOLVENT          CDCl3
NS                   8
DS                   2
SWH           8012.820 Hz
FIDRES        0.098043 Hz
AQ           5.0998774 sec
RG                 7.1
DW              62.400 usec
DE                6.00 usec
TE               298.0 K
D1          0.10000000 sec
MCREST      0.00000000 sec
MCWRK       0.01500000 sec

======== CHANNEL f1 ========
NUC1                1H
P1                9.75 usec
PL1               1.60 dB
SFO1       500.2235015 MHz

F2 - Processing parameters
SI               65536
SF         500.2200299 MHz
WDW                 EM
SSB                  0
LB                0.30 Hz
GB                   0
PC                1.00

1D NMR plot parameters
CX               22.80 cm
CY               15.00 cm
F1P              9.000 ppm
F1             4501.98 Hz
F2P             -0.500 ppm
F2             -250.11 Hz
PPMCM          0.41667 ppm/cm
HZCM         208.42502 Hz/cm
```

Z-restored spin-echo 13C spectrum with 1H decoupling

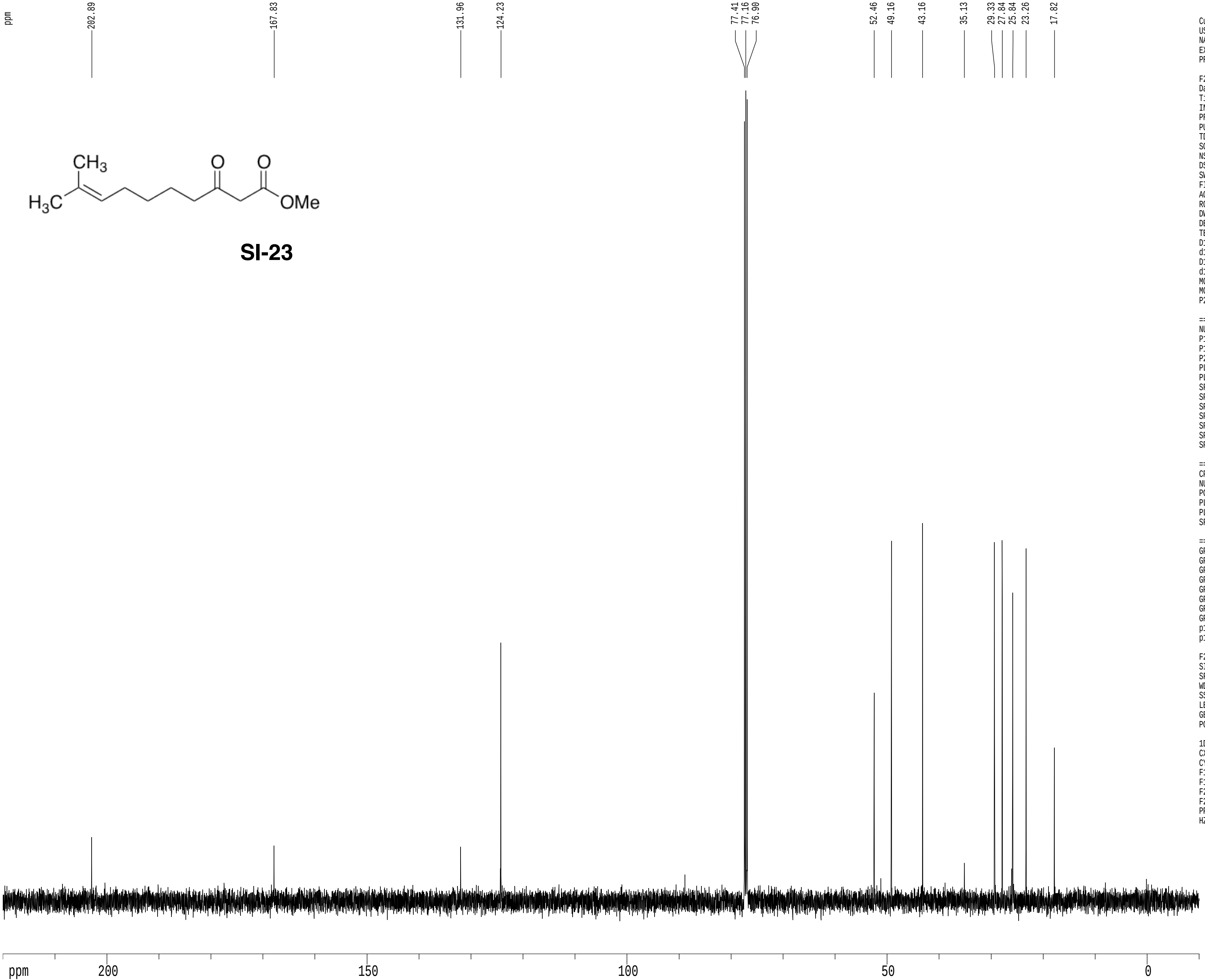

SI-23

Current Data Parameters
USER linpc2
NAME pcl-1-237
EXPNO 5
PROCNO 1

F2 - Acquisition Parameters
Date_ 20210319
Time 15.46
INSTRUM cryo500
PROBHD 5 mm CPTCI 1H-
PULPROG SpinEchopg30gp2.prd
TD 65536
SOLVENT CDCl3
NS 248
DS 16
SWH 30303.031 Hz
FIDRES 0.462388 Hz
AQ 1.0813940 sec
RG 9195.2
DW 16.500 usec
DE 6.00 usec
TE 298.0 K
D1 0.25000000 sec
d11 0.03000000 sec
D16 0.00020000 sec
d17 0.00019600 sec
MCREST 0.00000000 sec
MCWRK 0.01500000 sec
P2 37.70 usec

======== CHANNEL f1 ========
NUC1 13C
P1 18.85 usec
P12 2000.00 usec
P20 500.00 usec
PL0 120.00 dB
PL1 -1.00 dB
SFO1 125.7942548 MHz
SP2 1.55 dB
SP4 1.55 dB
SPNAM2 Crp60comp.4
SPNAM4 Crp60,0.5,20.1
SPOFF2 0.00 Hz
SPOFF4 0.00 Hz

======== CHANNEL f2 ========
CPDPRG2 waltz16
NUC2 1H
PCPD2 100.00 usec
PL2 1.60 dB
PL12 22.00 dB
SFO2 500.2225011 MHz

====== GRADIENT CHANNEL =====
GPNAM1 SINE.100
GPNAM2 SINE.100
GPX1 0.00 %
GPX2 0.00 %
GPY1 0.00 %
GPY2 0.00 %
GPZ1 30.00 %
GPZ2 50.00 %
p15 500.00 usec
p16 1000.00 usec

F2 - Processing parameters
SI 65536
SF 125.7804062 MHz
WDW EM
SSB 0
LB 1.00 Hz
GB 0
PC 2.00

1D NMR plot parameters
CX 22.80 cm
CY 15.65 cm
F1P 220.000 ppm
F1 27671.69 Hz
F2P -10.000 ppm
F2 -1257.80 Hz
PPMCM 10.08772 ppm/cm
HZCM 1268.83740 Hz/cm

1H spectrum

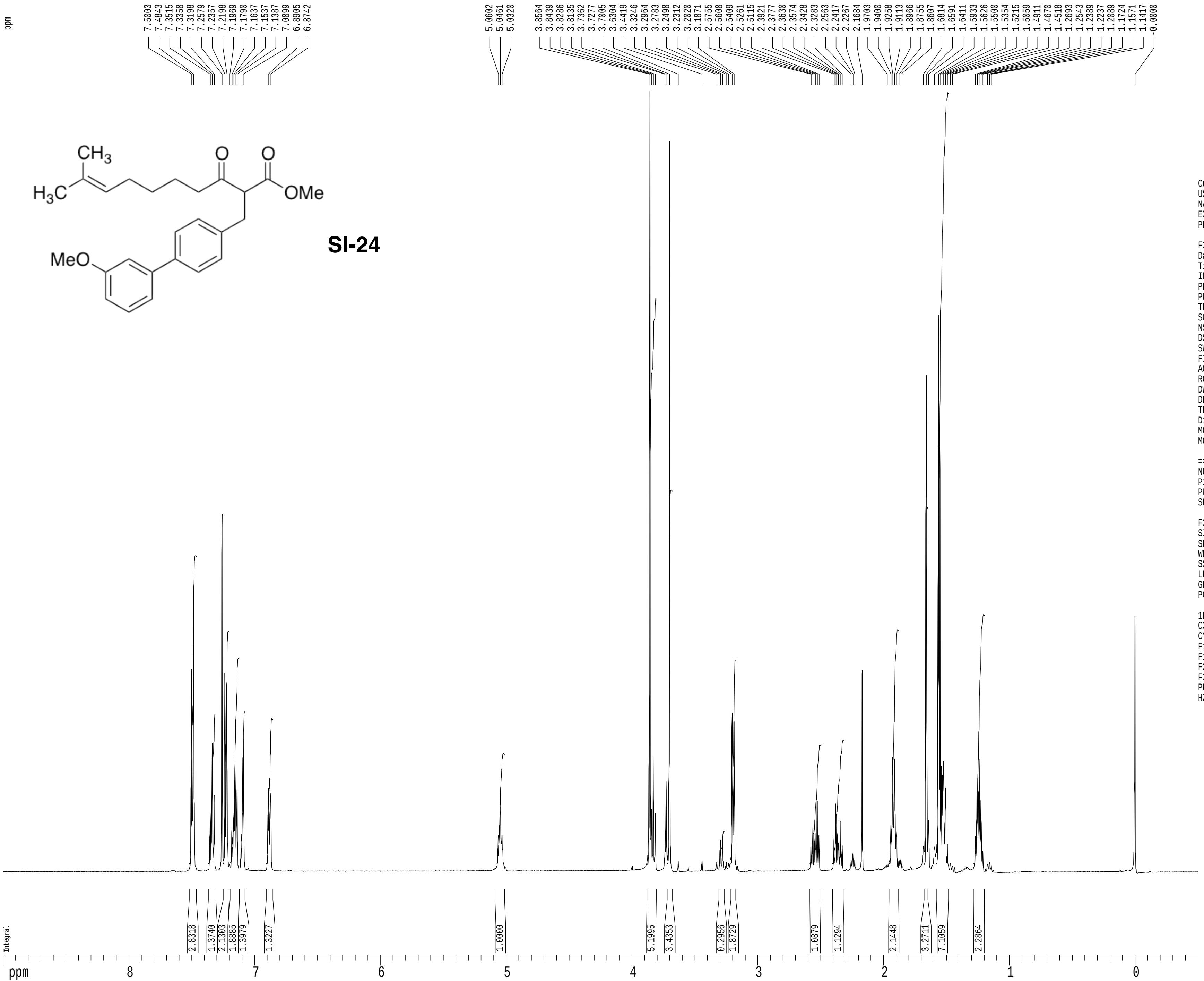

SI-24

Current Data Parameters
USER linpc2
NAME pcl-1-238
EXPNO 4
PROCNO 1

F2 - Acquisition Parameters
Date_ 20210319
Time 15.52
INSTRUM cryo500
PROBHD 5 mm CPTCI 1H-
PULPROG zg30
TD 81728
SOLVENT CDCl3
NS 8
DS 2
SWH 8012.820 Hz
FIDRES 0.098043 Hz
AQ 5.0998774 sec
RG 10.1
DW 62.400 usec
DE 6.00 usec
TE 298.0 K
D1 0.10000000 sec
MCREST 0.00000000 sec
MCWRK 0.01500000 sec

======== CHANNEL f1 ========
NUC1 1H
P1 9.75 usec
PL1 1.60 dB
SFO1 500.2235015 MHz

F2 - Processing parameters
SI 65536
SF 500.2200322 MHz
WDW EM
SSB 0
LB 0.30 Hz
GB 0
PC 1.00

1D NMR plot parameters
CX 22.80 cm
CY 15.00 cm
F1P 9.000 ppm
F1 4501.98 Hz
F2P -0.500 ppm
F2 -250.11 Hz
PPMCM 0.41667 ppm/cm
HZCM 208.42502 Hz/cm

Z-restored spin-echo 13C spectrum with 1H decoupling

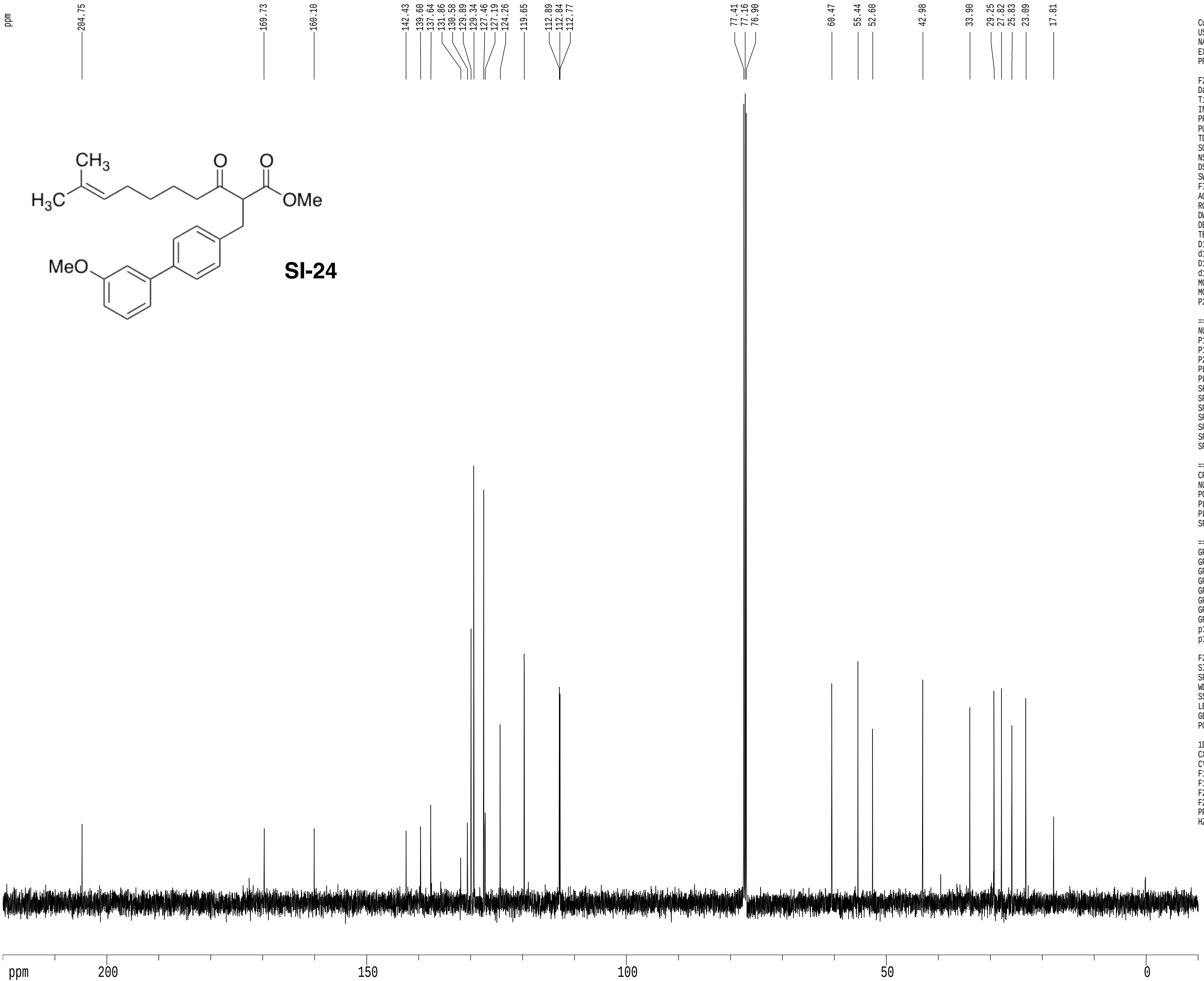


```
Current Data Parameters
USER              linpc2
NAME          pcl-1-238
EXPNO                  5
PROCNO                 1

F2 - Acquisition Parameters
Date_           20210319
Time               15.56
INSTRUM          cryo500
PROBHD   5 mm CPTCI 1H-
PULPROG  SpinEchopg30gp2.prd
TD                 65536
SOLVENT            CDCl3
NS                   184
DS                    16
SWH           30303.031 Hz
FIDRES         0.462388 Hz
AQ            1.0813940 sec
RG                2896.3
DW               16.500 usec
DE                 6.00 usec
TE                298.0 K
D1           0.25000000 sec
d11          0.03000000 sec
D16          0.00020000 sec
d17          0.00019600 sec
MCREST       0.00000000 sec
MCWRK        0.01500000 sec
P2                37.70 usec

======== CHANNEL f1 ========
NUC1                 13C
P1                18.85 usec
P12             2000.00 usec
P20              500.00 usec
PL0              120.00 dB
PL1               -1.00 dB
SFO1        125.7942548 MHz
SP2                1.55 dB
SP4                1.55 dB
SPNAM2      Crp60comp.4
SPNAM4   Crp60,0.5,20.1
SPOFF2             0.00 Hz
SPOFF4             0.00 Hz

======== CHANNEL f2 ========
CPDPRG2          waltz16
NUC2                  1H
PCPD2            100.00 usec
PL2                1.60 dB
PL12              22.00 dB
SFO2        500.2225011 MHz

====== GRADIENT CHANNEL =====
GPNAM1          SINE.100
GPNAM2          SINE.100
GPX1               0.00 %
GPX2               0.00 %
GPY1               0.00 %
GPY2               0.00 %
GPZ1              30.00 %
GPZ2              50.00 %
p15              500.00 usec
p16             1000.00 usec

F2 - Processing parameters
SI                 65536
SF          125.7804066 MHz
WDW                   EM
SSB                    0
LB                 1.00 Hz
GB                     0
PC                  2.00

1D NMR plot parameters
CX                 22.80 cm
CY                 15.65 cm
F1P              220.000 ppm
F1              27671.69 Hz
F2P              -10.000 ppm
F2              -1257.80 Hz
PPMCM           10.08772 ppm/cm
HZCM          1268.83740 Hz/cm
```

1H spectrum

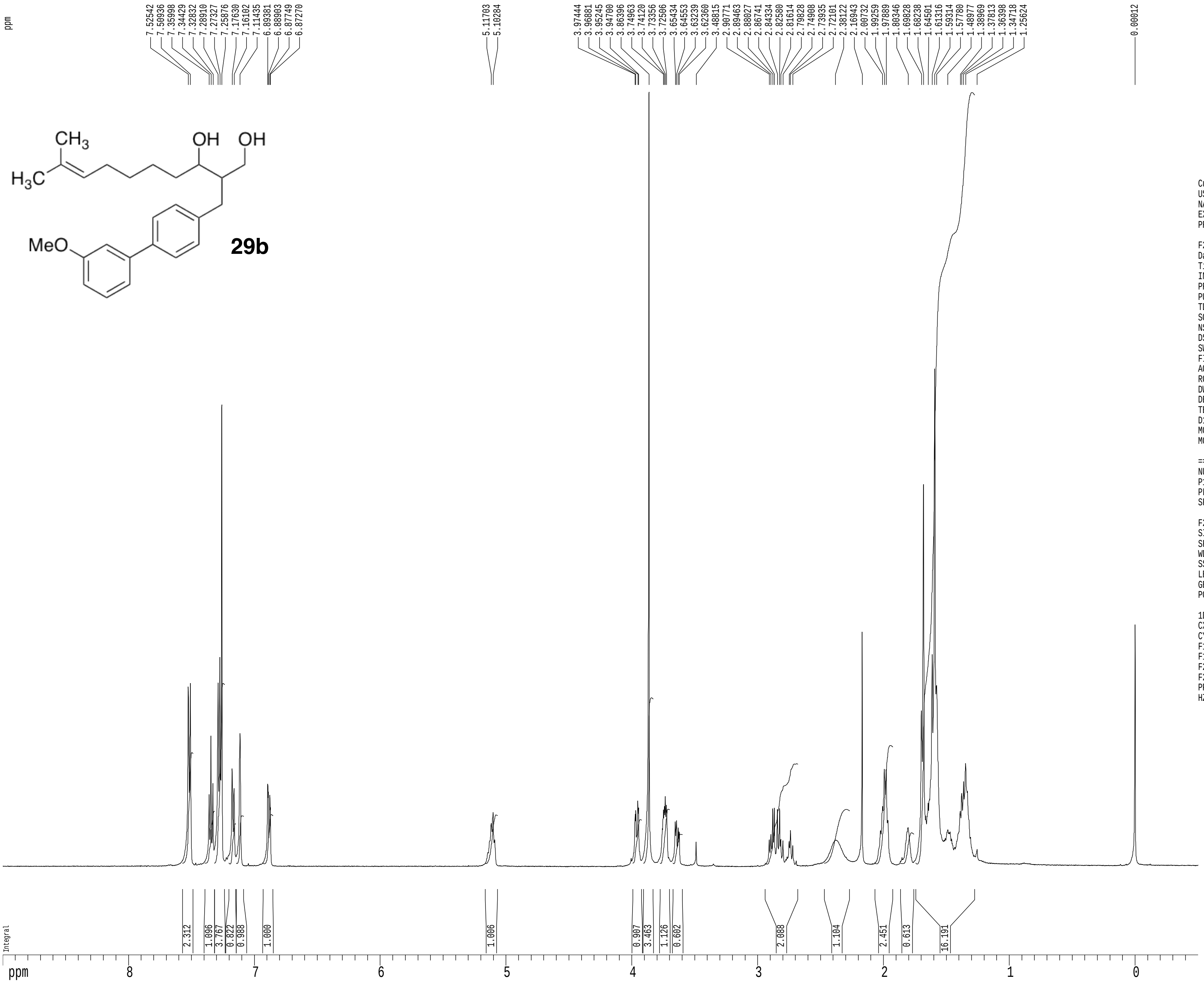

Current Data Parameters
USER linpc2
NAME pcl-1-239
EXPNO 4
PROCNO 1

F2 - Acquisition Parameters
Date_ 20210324
Time 8.15
INSTRUM cryo500
PROBHD 5 mm CPTCI 1H-
PULPROG zg30
TD 81728
SOLVENT CDCl3
NS 8
DS 2
SWH 8012.820 Hz
FIDRES 0.098043 Hz
AQ 5.0998774 sec
RG 4.5
DW 62.400 usec
DE 6.00 usec
TE 298.0 K
D1 0.10000000 sec
MCREST 0.00000000 sec
MCWRK 0.01500000 sec

======== CHANNEL f1 ========
NUC1 1H
P1 9.75 usec
PL1 1.60 dB
SFO1 500.2235015 MHz

F2 - Processing parameters
SI 65536
SF 500.2200315 MHz
WDW EM
SSB 0
LB 0.30 Hz
GB 0
PC 1.00

1D NMR plot parameters
CX 22.80 cm
CY 15.00 cm
F1P 9.000 ppm
F1 4501.98 Hz
F2P -0.500 ppm
F2 -250.11 Hz
PPMCM 0.41667 ppm/cm
HZCM 208.42502 Hz/cm

Z-restored spin-echo 13C spectrum with 1H decoupling

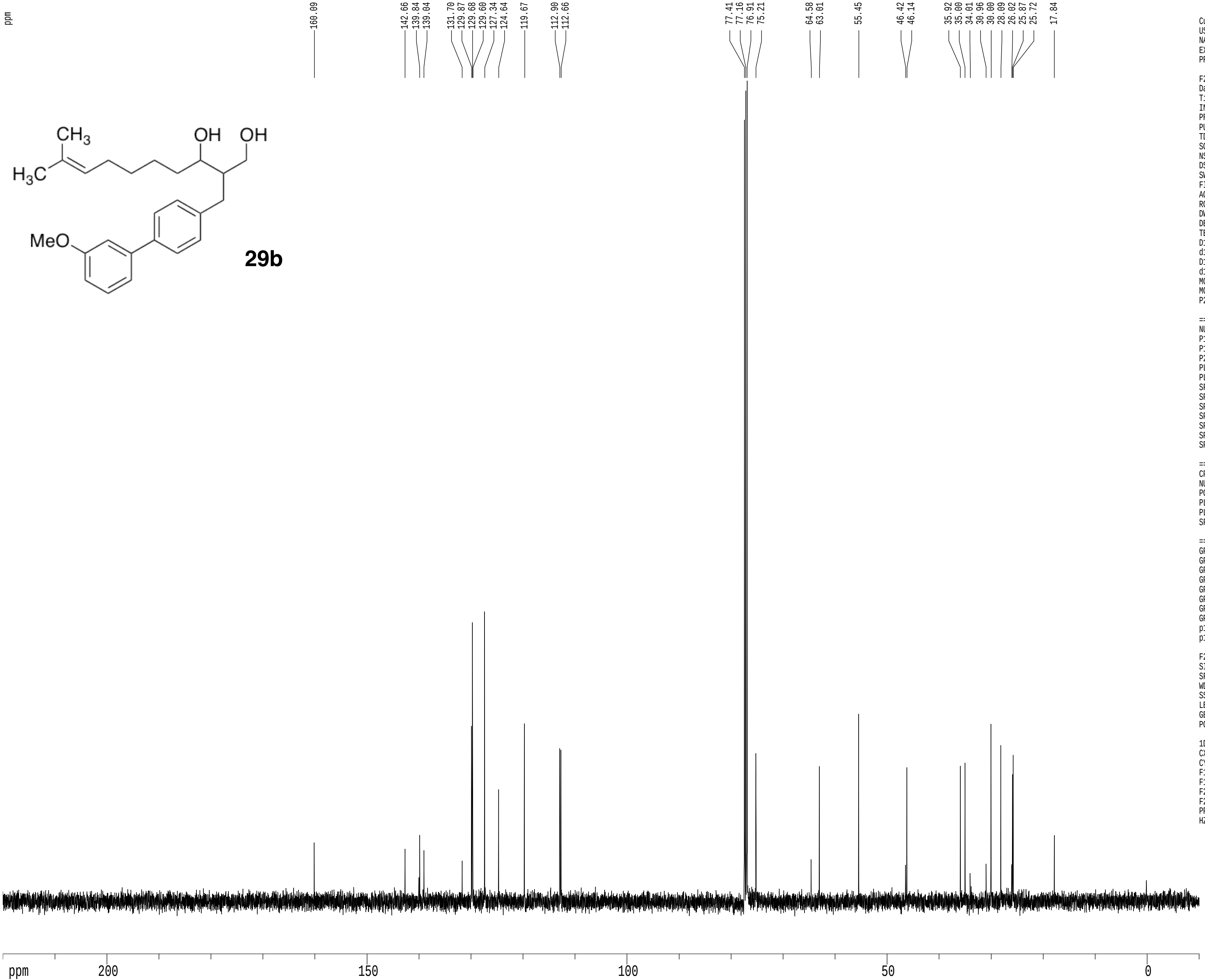

29b

Current Data Parameters
USER linpc2
NAME pcl-1-239
EXPNO 5
PROCNO 1

F2 - Acquisition Parameters
Date_ 20210324
Time 8.19
INSTRUM cryo500
PROBHD 5 mm CPTCI 1H-
PULPROG SpinEchopg30gp2.prd
TD 65536
SOLVENT CDCl3
NS 488
DS 16
SWH 30303.031 Hz
FIDRES 0.462388 Hz
AQ 1.0813940 sec
RG 4096
DW 16.500 usec
DE 6.00 usec
TE 298.0 K
D1 0.25000000 sec
d11 0.03000000 sec
D16 0.00020000 sec
d17 0.00019600 sec
MCREST 0.00000000 sec
MCWRK 0.01500000 sec
P2 37.70 usec

======== CHANNEL f1 ========
NUC1 13C
P1 18.85 usec
P12 2000.00 usec
P20 500.00 usec
PL0 120.00 dB
PL1 -1.00 dB
SFO1 125.7942548 MHz
SP2 1.55 dB
SP4 1.55 dB
SPNAM2 Crp60comp.4
SPNAM4 Crp60,0.5,20.1
SPOFF2 0.00 Hz
SPOFF4 0.00 Hz

======== CHANNEL f2 ========
CPDPRG2 waltz16
NUC2 1H
PCPD2 100.00 usec
PL2 1.60 dB
PL12 22.00 dB
SFO2 500.2225011 MHz

====== GRADIENT CHANNEL =====
GPNAM1 SINE.100
GPNAM2 SINE.100
GPX1 0.00 %
GPX2 0.00 %
GPY1 0.00 %
GPY2 0.00 %
GPZ1 30.00 %
GPZ2 50.00 %
p15 500.00 usec
p16 1000.00 usec

F2 - Processing parameters
SI 65536
SF 125.7804062 MHz
WDW EM
SSB 0
LB 1.00 Hz
GB 0
PC 2.00

1D NMR plot parameters
CX 22.80 cm
CY 15.65 cm
F1P 220.000 ppm
F1 27671.69 Hz
F2P -10.000 ppm
F2 -1257.80 Hz
PPMCM 10.08772 ppm/cm
HZCM 1268.83740 Hz/cm

1H spectrum

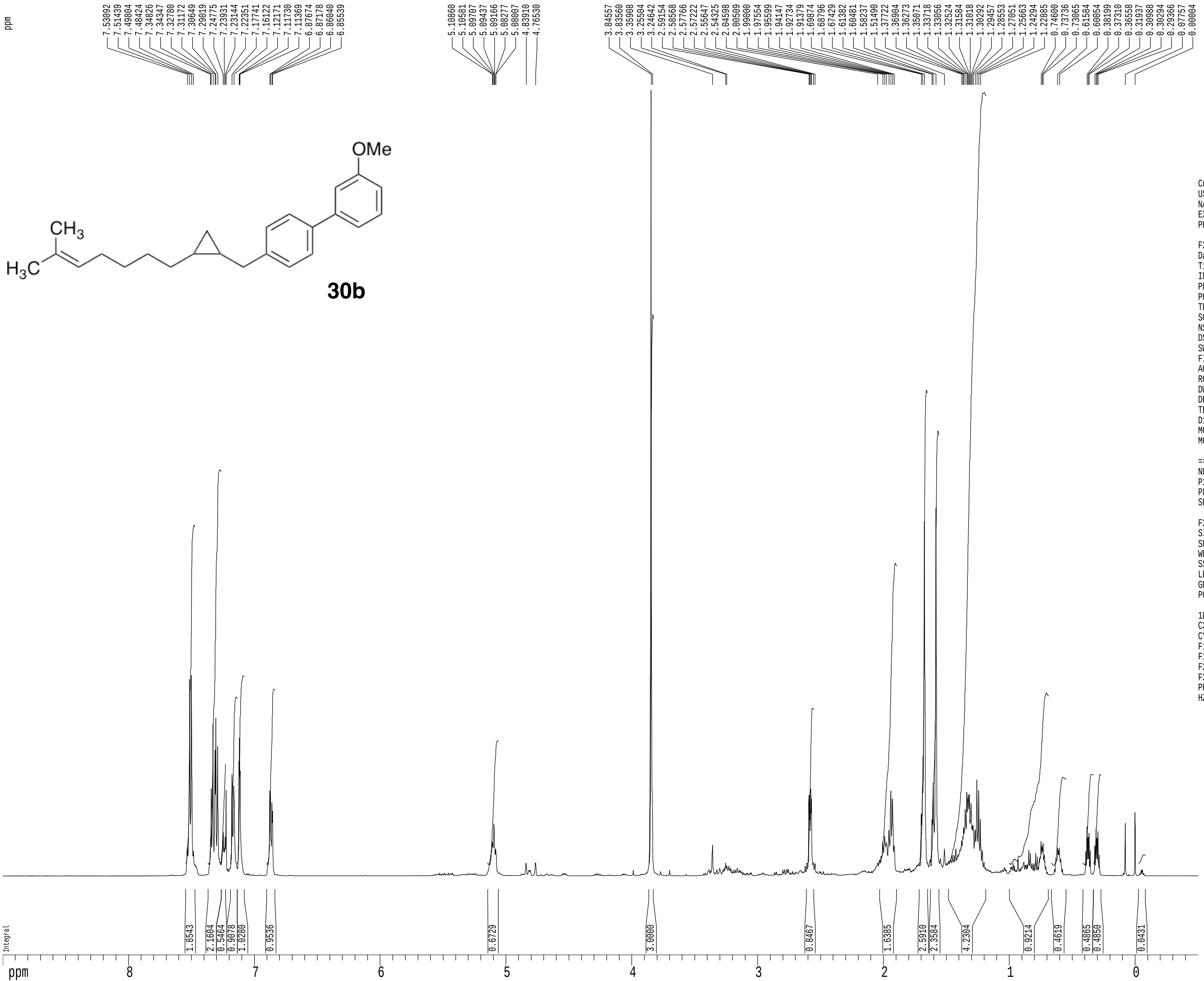

30b

Current Data Parameters
USER linpc2
NAME pcl-1-263
EXPNO 3
PROCNO 1

F2 - Acquisition Parameters
Date_ 20210312
Time 8.32
INSTRUM cryo500
PROBHD 5 mm CPTCI 1H-
PULPROG zg30
TD 81728
SOLVENT CDCl3
NS 8
DS 2
SWH 8012.820 Hz
FIDRES 0.098043 Hz
AQ 5.0998774 sec
RG 5
DW 62.400 usec
DE 6.00 usec
TE 298.0 K
D1 0.10000000 sec
MCREST 0.00000000 sec
MCWRK 0.01500000 sec

======== CHANNEL f1 ========
NUC1 1H
P1 9.75 usec
PL1 1.60 dB
SFO1 500.2235015 MHz

F2 - Processing parameters
SI 65536
SF 500.2200494 MHz
WDW EM
SSB 0
LB 0.30 Hz
GB 0
PC 1.00

1D NMR plot parameters
CX 22.80 cm
CY 15.00 cm
F1P 9.000 ppm
F1 4501.98 Hz
F2P -0.500 ppm
F2 -250.11 Hz
PPMCM 0.41667 ppm/cm
HZCM 208.42503 Hz/cm

Z-restored spin-echo 13C spectrum with 1H decoupling

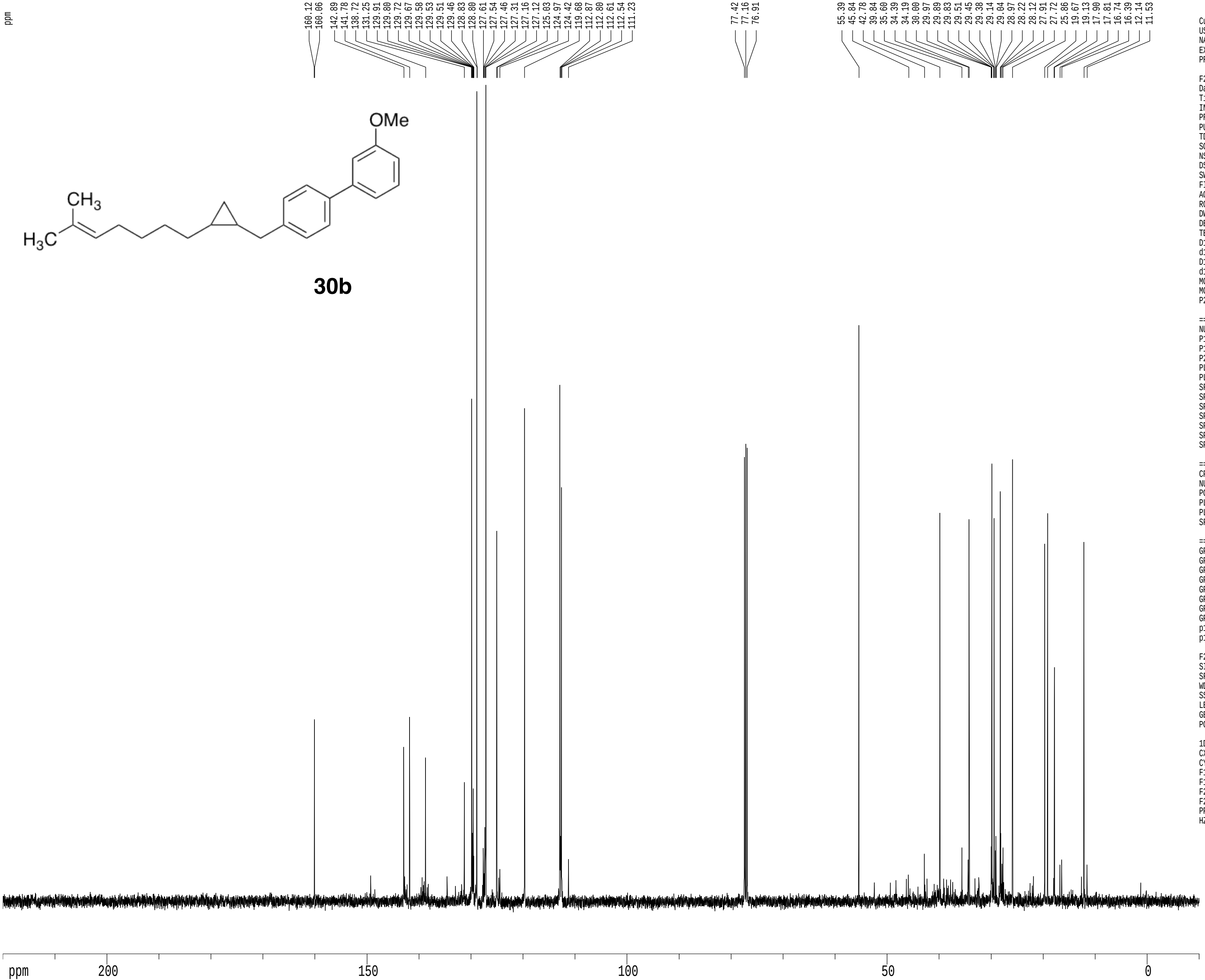

Current Data Parameters
USER linpc2
NAME pcl-1-263
EXPNO 4
PROCNO 1

F2 - Acquisition Parameters
Date_ 20210312
Time 8.37
INSTRUM cryo500
PROBHD 5 mm CPTCI 1H-
PULPROG SpinEchopg30gp2.prd
TD 65536
SOLVENT CDCl3
NS 120
DS 16
SWH 30303.031 Hz
FIDRES 0.462388 Hz
AQ 1.0813940 sec
RG 2896.3
DW 16.500 usec
DE 6.00 usec
TE 298.0 K
D1 0.25000000 sec
d11 0.03000000 sec
D16 0.00020000 sec
d17 0.00019600 sec
MCREST 0.00000000 sec
MCWRK 0.01500000 sec
P2 37.70 usec

======== CHANNEL f1 ========
NUC1 13C
P1 18.85 usec
P12 2000.00 usec
P20 500.00 usec
PL0 120.00 dB
PL1 -1.00 dB
SFO1 125.7942548 MHz
SP2 1.55 dB
SP4 1.55 dB
SPNAM2 Crp60comp.4
SPNAM4 Crp60,0.5,20.1
SPOFF2 0.00 Hz
SPOFF4 0.00 Hz

======== CHANNEL f2 ========
CPDPRG2 waltz16
NUC2 1H
PCPD2 100.00 usec
PL2 1.60 dB
PL12 22.00 dB
SFO2 500.2225011 MHz

====== GRADIENT CHANNEL =====
GPNAM1 SINE.100
GPNAM2 SINE.100
GPX1 0.00 %
GPX2 0.00 %
GPY1 0.00 %
GPY2 0.00 %
GPZ1 30.00 %
GPZ2 50.00 %
p15 500.00 usec
p16 1000.00 usec

F2 - Processing parameters
SI 65536
SF 125.7804117 MHz
WDW EM
SSB 0
LB 1.00 Hz
GB 0
PC 2.00

1D NMR plot parameters
CX 22.80 cm
CY 15.65 cm
F1P 220.000 ppm
F1 27671.69 Hz
F2P -10.000 ppm
F2 -1257.80 Hz
PPMCM 10.08772 ppm/cm
HZCM 1268.83752 Hz/cm
